# Regiodivergent construction of medium-sized heterocycles from vinylethylene carbonates and allylidenemalononitriles†

**DOI:** 10.1039/c9sc06377c

**Published:** 2020-02-10

**Authors:** Xiang Zhang, Xiang Li, Jun-Long Li, Qi-Wei Wang, Wen-Lin Zou, Yan-Qing Liu, Zhi-Qiang Jia, Fu Peng, Bo Han

**Affiliations:** West China School of Pharmacy, Sichuan University Chengdu 610041 China pengf@scu.edu.cn; State Key Laboratory of Southwestern Chinese Medicine Resources, School of Pharmacy, Chengdu University of Traditional Chinese Medicine Chengdu 611137 China hanbo@cdutcm.edu.cn; Chengdu Institute of Organic Chemistry, Chinese Academy of Sciences Chengdu 610041 China; Antibiotics Research and Re-evaluation Key Laboratory of Sichuan Province, Sichuan Industrial Institute of Antibiotics, Chengdu University Chengdu 610052 China

## Abstract

Medium-sized heterocycles exist in a broad spectrum of biologically active natural products and medicinally important synthetic compounds. The construction of medium-sized rings remains challenging, particularly the assembly of different ring sizes from the same type of substrate. Here we report palladium-catalyzed, regiodivergent [5 + 4] and [5 + 2] annulations of vinylethylene carbonates and allylidenemalononitriles. We describe the production of over 50 examples of nine- and seven-membered heterocycles in high isolated yields and excellent regioselectivities. We demonstrate the synthetic utility of this approach by converting a nine-membered ring product to an interesting polycyclic caged molecule *via* a [2 + 2] transannulation. Mechanistic studies suggest that the [5 + 2] annulation proceeds through palladium-catalyzed ring-opening/re-cyclization from the [5 + 4] adducts.

## Abstract 2

Here we report palladium-catalyzed, regiodivergent [5 + 4] and [5 + 2] annulations of vinylethylene carbonates and allylidenemalononitriles affording over 50 medium-sized heterocycles in high isolated yields with excellent regioselectivities.

## Graphical abstract

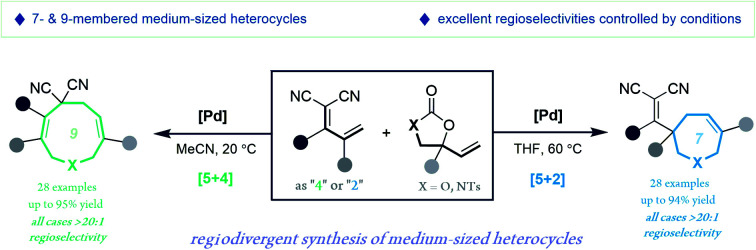

## Introduction

**Fig. 1:**
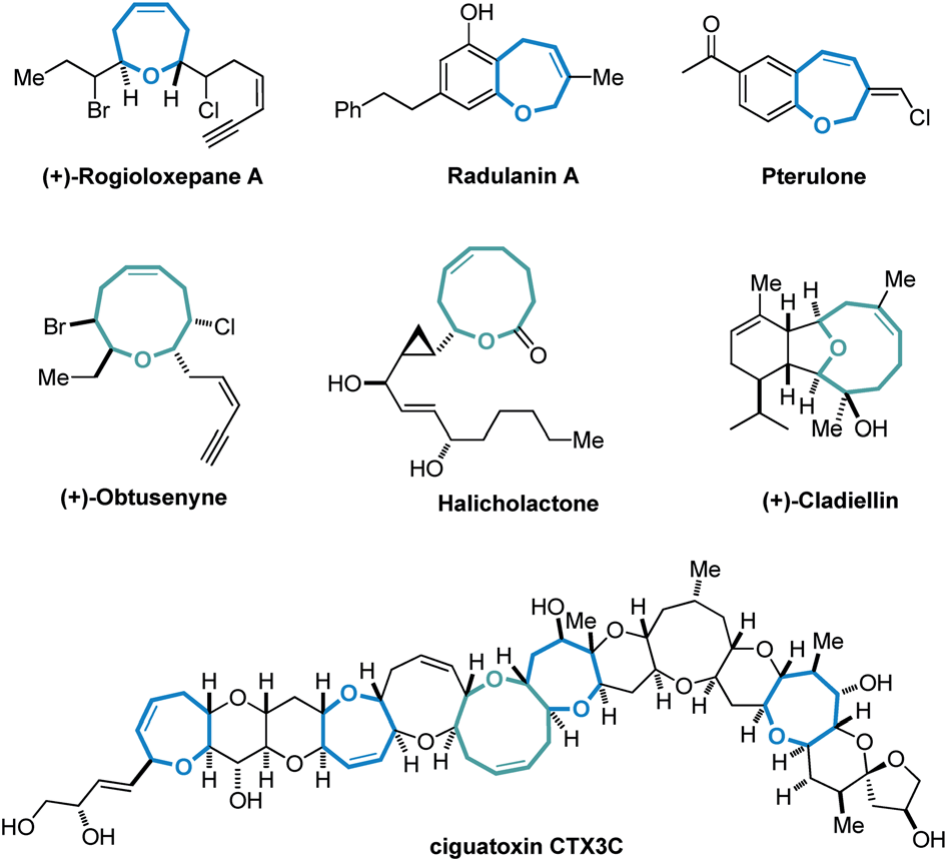
Selected natural products and synthetic bioactive compounds containing medium-sized oxo-heterocycles.

**Scheme 1:**
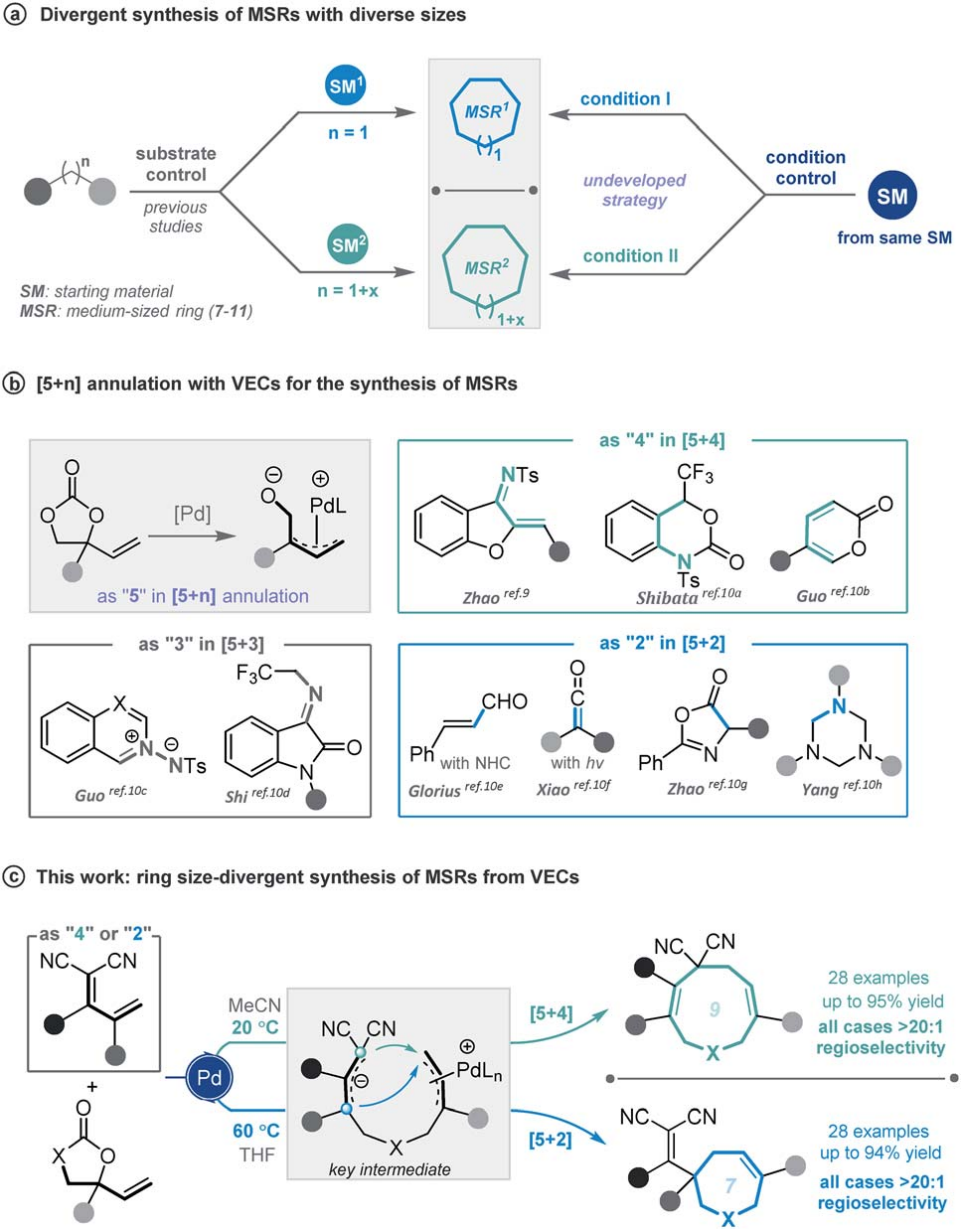
Divergent construction of medium-sized rings.

Cyclic molecular frameworks have special importance in chemical research and industry.^1^ Medium-sized rings (MSR, 7–11 members),^2^ particularly hetero-rings, exist in a large number of biologically active natural products and medicinally important synthetic molecules^3^ (Fig. 1). However, MSRs are challenging to prepare because of their inherent entropic factors and transannular interactions. Most established methods to generate MSRs are based on a fixed reaction site and suitable only for rings of the same size;^4^ changing the size of the ring usually requires changing the substrate design.^5^ Such a substrate-controlled strategy can be quite costly and inefficient because of the need to prepare the necessary substrate variants and optimize them in the ring-forming reactions. It could be much more efficient to develop a way to generate medium-sized rings of various sizes from the same set of substrates, simply by altering the reaction conditions. However, to our knowledge, controlling the regioselectivity of medium-sized ring cyclization is notoriously difficult and remains underdeveloped^6^ (Scheme 1a).

Vinylethylene carbonates (VECs) have recently emerged as versatile building blocks for various cyclizations, because of their inherent ability to undergo decarboxylation in the presence of a palladium catalyst to generate highly reactive zwitterionic π-allyl palladium intermediates.^7,8^ Recently, Zhao and co-workers disclosed that π-allyl palladium species can serve as 1,5-dipoles in a highly efficient [5 + 4] annulation with 1,3-azadienes to construct nine-membered hetero-rings.^9^ Since then, palladium-catalyzed [5 + *n*] annulations involving vinylethylene carbonates have been described for generating various medium-sized heterocycles^10^ (Scheme 1b). However, rarely have vinylethylene carbonates been used for divergent annulation,^10*h*,11^ and to our knowledge, they have never been applied to regioselective [5 + *n*] cyclization, which could generate multiple ring sizes.

Given our experience with the assembly of biologically interesting heterocycles by exploring novel catalytic reactions,^12^ we aimed to develop a convenient strategy for ring size-divergent construction of medium-sized rings. We found that by using the versatile, electron-deficient diene substrate allylidenemalononitriles,^13^ we could achieve smooth [5 + 4] annulation with vinylethylene carbonates in MeCN in the presence of a palladium catalyst at room temperature, generating a nine-membered product. More importantly, we could completely shift the regioselectivity to [5 + 2] cyclization by changing the solvent to THF and increasing the reaction temperature, generating a seven-membered product. In both cases, the regioselectivity was nearly perfect (Scheme 1c). In addition, the nine-membered cyclic ether adducts were able to undergo intramolecular transannular [2 + 2] cycloaddition^14^ to build a structurally interesting caged polycycle.

## Results and discussion

Our investigations began with a reaction between the easily accessible diene **1a** and vinylethylene carbonate **2a**. Different solvents were evaluated in the presence of Pd(PPh_3_)_4_ at 20 °C, and MeCN afforded the [5 + 4] adduct **3a** with a high yield and regioselectivity, while other solvents provided a mixture of nine- and seven-membered products (Table 1, entries 1–5) or **3a** in low yield (entry 6). The reaction in THF gave the highest ratio of [5 + 2] product **4a**, which encouraged us to screen the reaction conditions further in order to switch the regioselectivity. With THF as the solvent, phosphine ligands **L1–L7** were screened, but all reacted inefficiently (entry 7). To our gratification, conducting the reaction at 40 °C improved the relative amount of seven-membered cyclic ether **4a**, and increasing the temperature to 60 °C afforded **4a** as a single regioisomer in high yield (entries 8 and 9). Further increasing the temperature maintained the high regioselectivity but lowered the yield slightly (entry 10). Using other solvents at 60 °C did not improve the results in terms of yield and regioselectivity (entries 11–16).^15^

**Table 1:** Optimization studies for the annulation of allylidenemalononitril **1a** and VEC **2a**^a^

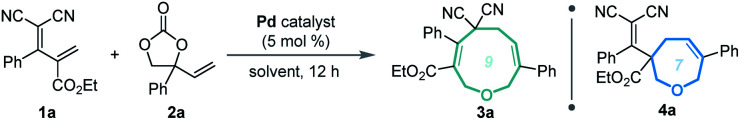					
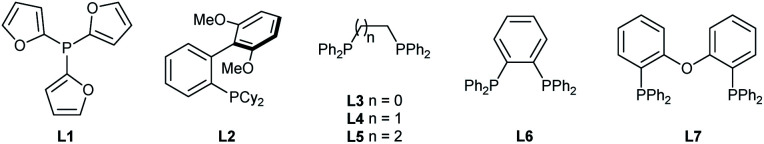					
Entry	Catalyst	Solvent	Temp. (°C)	Yield^b^ (%)	**3a** : **4a**^c^
1^d^	Pd(PPh_3_)_4_	Toluene	20	72	3.5 : 1
**2** e	**Pd(PPh**_**3**_ **)**_**4**_	**MeCN**	**20**	**96(90)**	**>20 : 1**
3^d^	Pd(PPh_3_)_4_	DCM	20	68	3.6 : 1
4^d^	Pd(PPh_3_)_4_	CHCl_3_	20	85	2.6 : 1
5	Pd(PPh_3_)_4_	THF	20	85	1.4 : 1
6^e^	Pd(PPh_3_)_4_	DMF	20	16	>20 : 1
7^f^	Pd/**L1–L7**	THF	20	<5	—
8	Pd(PPh_3_)_4_	THF	40	98	1 : 4.6
**9**	**Pd(PPh**_**3**_ **)**_**4**_	**THF**	**60**	**91(84)**	**<1 : 20**
10	Pd(PPh_3_)_4_	THF	80	89	<1 : 20
11	Pd(PPh_3_)_4_	1,4-Dioxane	60	83	16.0 : 1
12	Pd(PPh_3_)_4_	Toluene	60	88	1 : 1.3
13	Pd(PPh_3_)_4_	MeCN	60	87	8.6 : 1
14	Pd(PPh_3_)_4_	DMF	60	81	14.8 : 1
15	Pd(PPh_3_)_4_	DCM	60	76	1 : 1.4
16	Pd(PPh_3_)_4_	CHCl_3_	60	80	5.3 : 1

a Unless noted otherwise, the reactions were carried out with **1a** (0.10 mmol), **2a** (0.15 mmol) and the Pd catalyst (5 mol%) in solvent (1 mL) for 12 h.

b Yield was determined by ^1^H-NMR analysis with CH_2_Br_2_ as the internal standard; the data in parentheses refer to isolated yields.

c The ratio of **3a** : **4a** was determined by ^1^H-NMR analysis of the crude reaction mixture.

d For 48 h.

e For 24 h.

f The Pd/ligand complex was pre-prepared with Pd_2_(dba)_3_·CHCl_3_ and a ligand in THF at rt for 1 h.

Based on the optimized conditions for generating the seven- and nine-membered rings, we explored the generality of our method with various substituted allylidenemalononitriles **1** and vinylethylene carbonates **2**. Each substrate combination was tested under conditions A or B to generate, respectively, nine-membered products **3** or seven-membered products **4** (Table 2). First, we tested a range of electrophiles **1** with various aryl groups bearing different electronic and steric substituents, delivering the [5 + 4] adducts **3a–3h** or [5 + 2] adducts **4a–4h** in reasonable yields with excellent regioselectivities. Divergent annulations proceeded smoothly with a diene electrophile bearing a 2-naphthyl moiety, selectively affording the medium-sized rings **3i** and **4i** with satisfactory results. The reactions also worked well for thienyl-substituted **1**, generating the products **3j** and **4j** with impressive yields and regioselectivities. Different ester groups on **1** did not harm the reaction (**3k–3l** and **4k–4l**). We also tested three types of allylidenemalononitril substrates changing the ester group to hydrogen, but none of them could offer the desired products (see the ESI† for detailed experimental procedure). Next, we examined the reaction of **1a** with vinylethylene carbonates **2** featuring either an electron-donating or -withdrawing group on the benzene ring. The corresponding nine-membered products **3m–3y** and seven-membered products **4m–4y** were obtained with high isolated yields and regioselectivities. Naphthyl- and heteroarene-substituted **2** also performed well in the regiodivergent cyclizations (**3z–3aa** and **4z–4aa**). Moreover, this methodology is not tolerant to the VECs bearing aliphatic substituents (see the ESI† for more details).

**Table 2:** Substrate scope for the divergent annulation of allylidenemalononitrils **1** and VECs **2**^a^

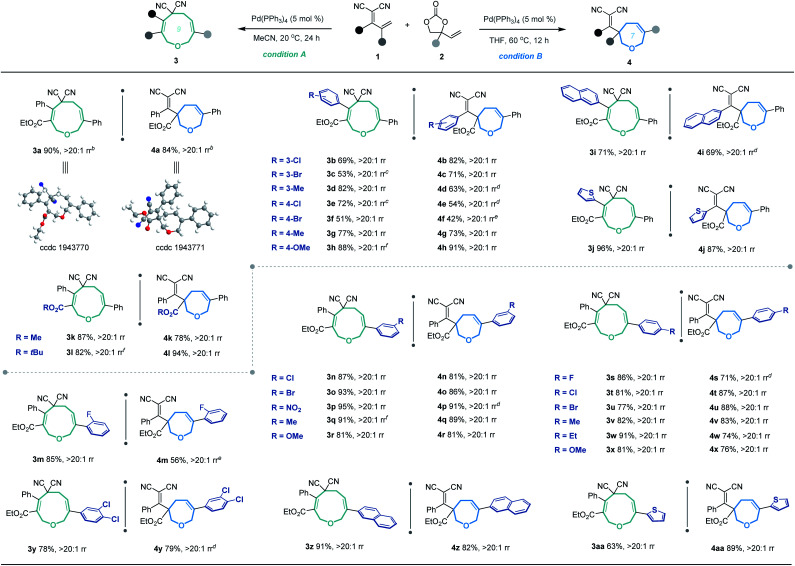

a Unless noted otherwise, the [5 + 4] annulation was performed under conditions A: **1** (0.1 mmol), **2** (0.15 mmol) and Pd(PPh_3_)_4_ (5 mol%) in MeCN (1.0 mL) at 20 °C for 24 h, and the rr (regioisomeric ratio) refers to the ratio of **3** : **4**; the [5 + 2] annulation was performed under conditions B: **1** (0.1 mmol), **2** (0.15 mmol) and Pd(PPh_3_)_4_ (5 mol%) in THF (1.0 mL) at 60 °C for 12 h, and the rr refers to the ratio of **4** : **3**; yield of the isolated product; rr was determined by ^1^H-NMR analysis of the crude reaction mixture.

b The structures of **3a** and **4a** were determined by X-ray diffraction analysis, and the structures of other products were assigned by analogy.

c For 48 h.

d At 80 °C.

e At 100 °C.

f With 0.3 mmol of **2**.

**Scheme 2:**
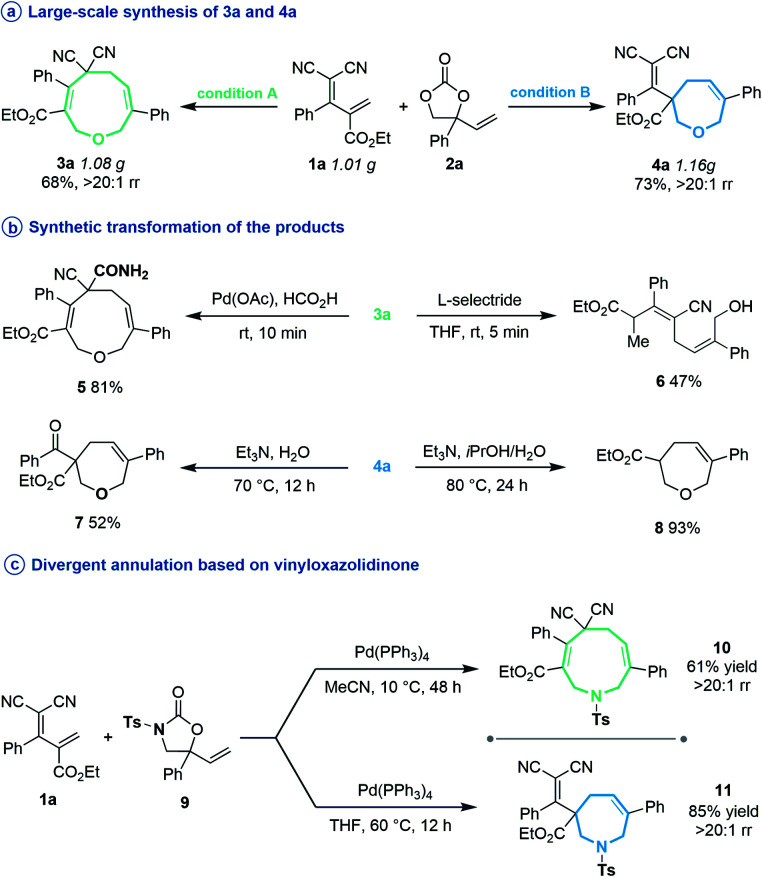
Large-scale reactions of regiodivergent cyclizations and further synthetic applications.

Subsequently, several experiments were performed to demonstrate the robustness and practicality of this synthetic method. Firstly, both [5 + 4] and [5 + 2] annulation of diene **1a** and vinylethylene carbonate **2a** could be scaled up to the 1 gram scale without drastic loss of yield (Scheme 2a). Then, the synthetic utility of our approach was explored, and we found that one of the two cyano groups on **3a** could be selectively hydrolyzed in formic acid in the presence of a Pd(OAc)_2_ catalyst, delivering **5** in 81% yield (Scheme 2b). Treating **3a** with l-selectride triggered reductive C–O bond cleavage that opened the nine-membered ring, offering linear 1,4-diene alcohol **6** in moderate yield. The product **4a** could undergo a retro-Knoevenagel reaction under aqueous basic conditions to release the malononitrile moiety and give the ketone-containing derivative **7** in 52% yield. It could also undergo sequential retro-Knoevenagel and retro-Claisen condensation in the presence of Et_3_N, iPrOH and water to afford product **8** in excellent yield. In addition, we extended this divergent cyclization strategy to a reaction between **1a** and vinyloxazolidinone **9**, assembling the nine- and seven-membered azacycles **10** and **11** in satisfying yields with excellent regioselectivities (Scheme 2c).

**Scheme 3:**
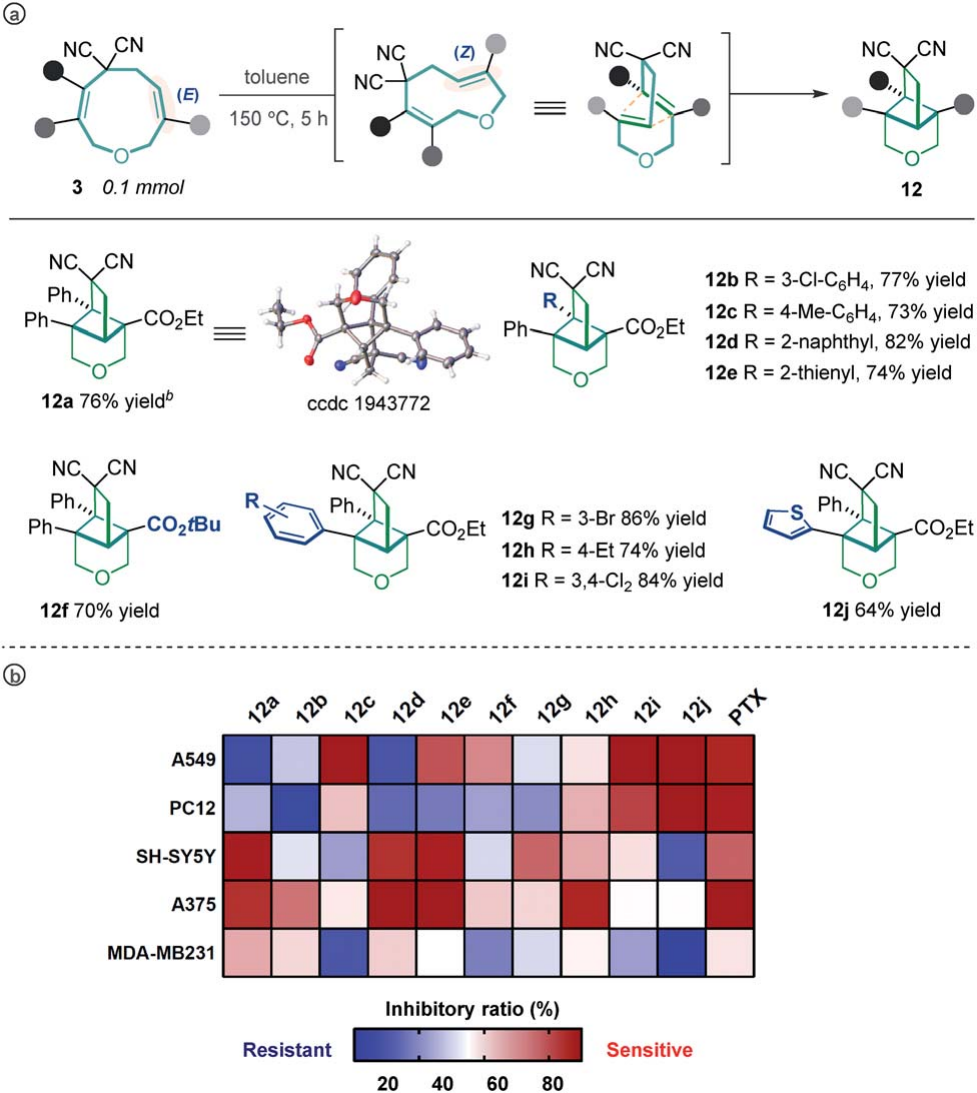
The transannular [2 + 2] cycloaddition of **3** (a) and heat map of the mean inhibitory ratio of compounds **12a–12j** against a panel of cancer cell lines (b).

Unexpectedly, heating the [5 + 4] adduct **3a** without the Pd catalyst in toluene generated a cage-like molecule **12a** in high yield. The structure of **12a** was confirmed by X-ray diffraction analysis. We attribute the formation of this product to heat-induced isomerization of the styrene moiety from the *E*- to *Z*-configuration, followed by transannular [2 + 2] cycloaddition (for the preliminary mechanism investigation, see the ESI†). This reaction proved tolerant of various functional groups, allowing the rapid synthesis of caged compounds **12a–12j** (Scheme 3a). With a series of synthesized molecule fused pharmacologically privileged frameworks in hand and motivated by the pharmaceutical properties of nitrile,^16*a*–*c*^ oxygen heterocycles^1*f*^ and caged-skeletons,^16*d*–*h*^ we preliminarily evaluated their ability to inhibit the proliferation of a panel of cancer cell lines (Scheme 3b). In these experiments, the concentrations of tested compounds and paclitaxel (PTX) were 20 μM and 5 μM, respectively. Compounds **12c**/**j**, **12j**, **12a** and **12d** showed promising cytotoxicity against A549, PC12, SH-SY5Y and A375 cells, respectively (for the details, see ESI, Table S3†).

**Scheme 4:**
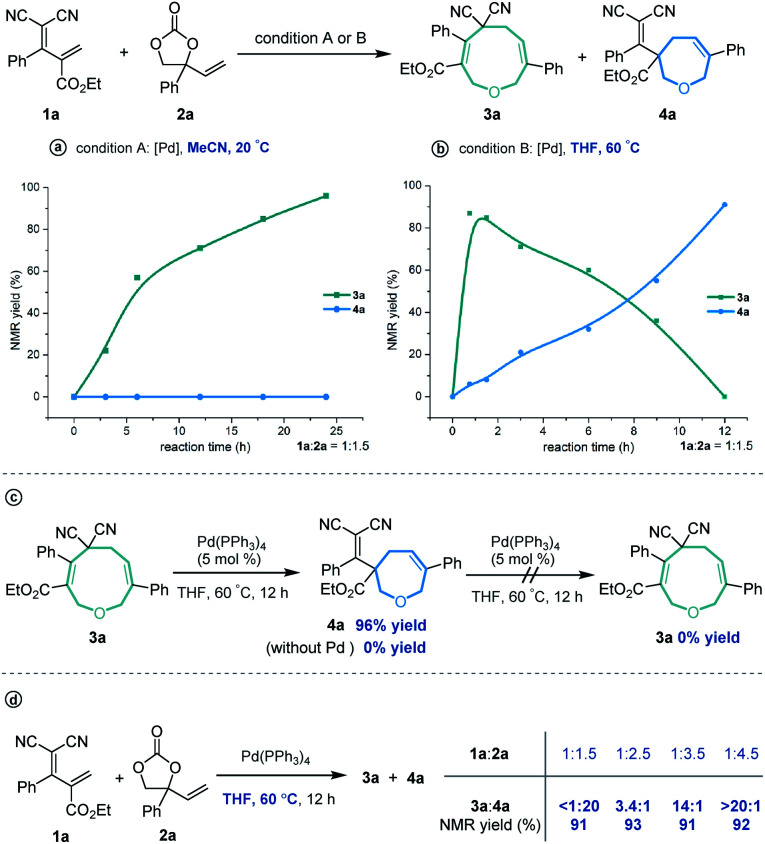
Control experiments. (a) Reaction progress was monitored in MeCN at 20 ^°^C; (b) Reaction progress was monitored in THF at 60 ^°^C; (c) Transformation from **3a** to **4a**; (d) Effect of the loading of VEC on the regioisomeric ratio.

In order to investigate the reaction mechanism, we performed several control experiments based on the reaction of allylidenemalononitril **1a** and vinylethylene carbonate **2a**. Firstly, the reaction progress was monitored by NMR analysis. As shown in Scheme 4a, under the [5 + 4] annulation reaction conditions, the nine-membered product **3a** formed gradually, without concomitant emergence of the [5 + 2] seven-membered product **4a**. In contrast, in the reaction meant to produce **4a**, the starting material **1a** was rapidly consumed and **3a** was initially generated in high NMR yield, together with trace amounts of **4a**. Subsequently, the ratio of **3a**/**4a** slowly decreased until **4a** was obtained as the sole regioisomer (Scheme 4b). Follow-up experiments showed that in the presence of a palladium catalyst in THF at 60 °C, **3a** converted to **4a**, but not *vice versa* (Scheme 4c). These results suggest that the nine-membered **3a** undergoes palladium-catalyzed ring-opening/re-cyclization to produce **4a**. In addition, we found that using excess vinylethylene carbonate inhibited the transformation from **3a** into **4a** under heating conditions in THF (Scheme 4d), probably because the palladium catalyst prefers to coordinate with a higher concentration of vinylethylene carbonate which blocks the palladium activation of **3a**.^17^

**Fig. 2:**
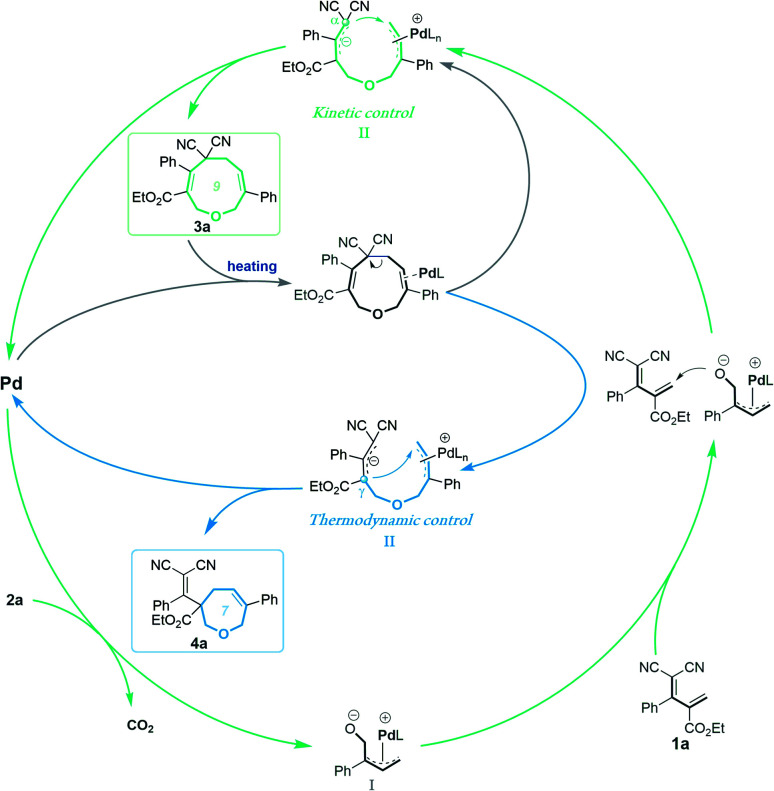
Proposed mechanism.

These experimental results suggest the following mechanism to rationalize the regioselectivity of the [5 + 4] and [5 + 2] annulations (Fig. 2). The palladium-catalyzed decarboxylation of vinylethylene carbonate **2a** generates an ambiphilic π-allyl palladium intermediate **I**, which undergoes vinylogous Michael addition with allylidenemalononitril **1a** to form intermediate **II**. At lower temperature and in MeCN solvent, the π-allylic anion is stabilized by dicyano electron-withdrawing groups, so the corresponding α terminal carbon attacks the electrophilic π-allyl palladium moiety to deliver **3a**in a kinetically controlled manner. At higher temperature and in THF solvent, the same pathway generates **3a**, which can revert to intermediate **II***via* palladium-catalyzed ring-opening, but en route it can undergo a different ring-closing reaction between an internal γ-carbon and the π-allyl palladium moiety, delivering **4a** in a thermodynamically controlled reaction.

## Conclusions

In summary, we have developed a regiodivergent cyclization of vinylethylene carbonates and allylidenemalononitriles for the synthesis of medium-sized heterocycles. [5 + 4] annulation proceeds smoothly in MeCN at lower temperature, delivering nine-membered oxo-heterocycles in high yields. Changing the solvent to THF and raising the temperature completely reverse the regioselectivity of the ring-closing step, giving rise to [5 + 2] annulation that generates seven-membered heterocycles. In this way, our strategy allows the selective assembly of two heterocycle sizes from the same set of substrates through simple manipulation of reaction conditions. The nine-membered products efficiently undergo a transannular [2 + 2] cycloaddition to afford intriguing caged ring systems. Mechanistic studies suggest that [5 + 2] cyclization may occur *via* palladium-catalyzed ring-opening/cyclization from [5 + 4] adducts. Further biological studies of these novel cyclic molecules are currently underway in our laboratory, and the results will be reported in due course.

## Conflicts of interest

The authors declare no conflict of interest.

## Supplementary Material

SC-011-C9SC06377C-s001

SC-011-C9SC06377C-s002
