# Supplementary material for: Regiodivergent construction of medium-sized heterocycles from vinylethylene carbonates and allylidenemalononitriles
Source: Chem Sci. 2020 Feb 10;11(11):2888–94. doi: 10.1039/c9sc06377c (PMC8157681; doi:10.1039/c9sc06377c)
Supplement: SC-011-C9SC06377C-s001 [file SC-011-C9SC06377C-s001.pdf]

Electronic Supplementary Material (ESI) for Chemical Science.
This journal is © The Royal Society of Chemistry 2020

Regiodivergent Construction of Medium-Sized Heterocycles from Vinylethylene Carbonates and Allylidenemalononitrils

Xiang Zhang,[a,c,d] Xiang Li,[b] Jun-Long Li,[d] Qi-Wei Wang,[c,d] Wen-Lin Zou,[d] Yan-Qing Liu,[b,d] Zhi-Qiang Jia,[d] Fu Peng*[,a] and Bo Han*[,b]

[a] *West China School of Pharmacy, Sichuan University, Chengdu 610041, China. E-mail: pengf@scu.edu.cn.*

[b] *State Key Laboratory of Southwestern Chinese Medicine Resources, School of Pharmacy, Chengdu University of Traditional Chinese Medicine, Chengdu 611137, China. E-mail: hanbo@cdutcm.edu.cn.*

[c] *Chengdu Institute of Organic Chemistry, Chinese Academy of Sciences, Chengdu 610041, China.*

[d] *Antibiotics Research and Re-evaluation Key Laboratory of Sichuan Province, Sichuan Industrial Institute of Antibiotics, Chengdu University, Chengdu 610052, China.*

Supporting Information

Table of Contents

1. **General Information.......S2**
2. **Further Optimization Studies.......S3**
3. **Further Substrate Scope Investigation.......S5**
4. **General Procedure for the Preparation of Nine-Membered Products 3.......S6**
5. **General Procedure for the Preparation of Seven-Membered Products 4.......S23**
6. **Synthetic Transformation of 3a and 4a.......S42**
7. **Procedure for the Synthesis of Medium-Sized Azacycles.......S46**
8. **General Procedure and Mechanism Studies for the [2+2] Cycloaddition.......S49**
9. **Preliminary Evaluation of Biological Activity for Compounds 12.......S56**
10. **Experiments for Mechanism Studies.......S58**
11. **Crystal Data and Structure Refinement.......S63**
12. **References and Notes.......S66**
13. **NMR Spectra.......S67**

1. General Information

General Procedures

- All reactions were performed in oven-dried or flame-dried reaction vessels, modified Schlenk flasks, or round-bottom flasks. The flasks were fitted with Teflon screw caps and reactions were conducted under an atmosphere of argon if needed. Gas-tight syringes with stainless steel needles were used to transfer air- and moisture-sensitive liquids. All moisture and/or air sensitive solid compounds were manipulated inside normal desiccators. Flash column chromatography was performed using silica gel (40–63 μm, 230–400 mesh).
- Analytical thin layer chromatography (TLC) was performed on silica gel 60 F_{254} aluminum plates (Merck) containing a 254 nm fluorescent indicator. TLC plates were visualized by exposure to short wave ultraviolet light (254 nm) and I_2.
- Organic solutions were concentrated at 30-50 °C on rotary evaporators at ~10 torr followed by drying on vacuum pump at ~1 torr. Reaction temperatures are reported as the temperature of the bath surrounding the vessel unless otherwise stated.

Materials

- Commercial reagents and solvents were were purchased from Adamas-beta, Aldrich Chemical Co., Alfa Aesar, Macklin and Energy Chemical and used as received with the following exceptions: THF, Et_2O and toluene were purified by refluxing over Na-benzophenone under positive argon pressure followed by distillation.[1] The allylidenemalononitriles **1**[2], vinylethylene carbonates **2**[3], and oxazolidinone **9**[4] were prepared according to literature procedure.

Instrumentation

- Proton nuclear magnetic resonance (^{1}H NMR) spectra were recorded with JEOL-600M. Proton chemical shifts are reported in parts per million (δ scale), and are referenced using residual protium in the NMR solvent ($CDCl_3$: δ 7.26 ($CHCl_3$)). Data are reported as follows: chemical shift [multiplicity (s = singlet, d = doublet, t = triplet, q = quartet, m = multiplet, br s = broad singlet), coupling constant(s) (Hz), integration].
- Carbon-13 nuclear magnetic resonance (^{13}C NMR) spectra were recorded with JEOL 150 MHz spectrometers. Carbon chemical shifts are reported in parts per million (δ scale), and are referenced using the carbon resonances of the solvent (δ 77.0 ($CHCl_3$)). Data are reported as follows: chemical shift [multiplicity (if not singlet), assignment (C_q = fully substituted carbon)].
- High resolution mass spectra (HRMS) were recorded on a Waters SYNAPT G2 using an electrospray (ESI) ionization source.
- Melting points were recorded on WRX-X-4A melting point apparatus.
- The reaction temperature of 20 °C and 10 °C were controlled with a DHJF-8002 Zhengzhou Changsheng cryogenic thermostatic reaction bath.

2. Further Optimization Studies

Table S1. Further screening of ligands and additives [a]

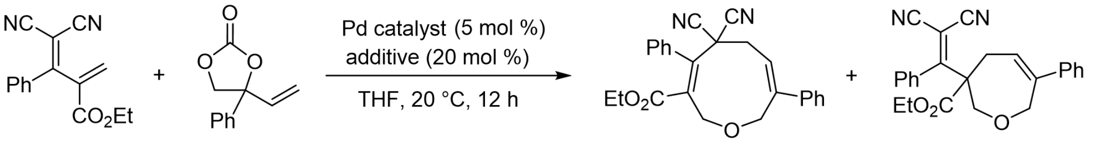

1a **2a** **3a** **4a**

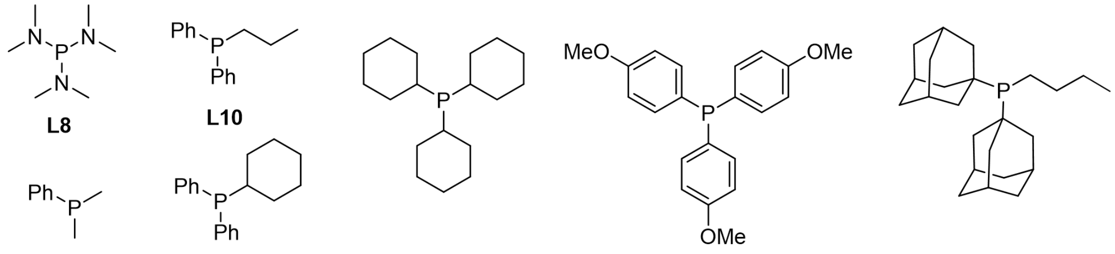

L8 **L9** **L10** **L11** **L12** **L13** **L14**

entry	catalyst	ligand	additive	yield (%)[b]	**3a**:**4a**[c]
1	$Pd_2(dba)_3 \cdot CHCl_3$	–	–	/	/
2[d]	$Pd_2(dba)_3 \cdot CHCl_3$	**L8**	–	<5	/
3[d]	$Pd_2(dba)_3 \cdot CHCl_3$	**L9**	–	/	/
4[d]	$Pd_2(dba)_3 \cdot CHCl_3$	**L10**	–	/	/
5[d]	$Pd_2(dba)_3 \cdot CHCl_3$	**L11**	–	/	/
6[d]	$Pd_2(dba)_3 \cdot CHCl_3$	**L12**	–	/	/
7[d]	$Pd_2(dba)_3 \cdot CHCl_3$	**L13**	–	<5	/
8[d]	$Pd_2(dba)_3 \cdot CHCl_3$	**L14**	–	/	/
9	$Pd(PPh_3)_4$	–	$Sc(OTf)_3$	<5	/
10	$Pd(PPh_3)_4$	–	$Al(OTf)_3$	81	2.0:1
11	$Pd(PPh_3)_4$	–	Ti(O*i*Pr)$_4$	75	9.6:1
12	$Pd(PPh_3)_4$	–	Mg(O*t*Bu)$_2$	89	2.8:1
13	$Pd(PPh_3)_4$	–	$PhB(OH)_2$	86	1.3:1
14[e]	$Pd(PPh_3)_4$	–	Cs_2CO_3	89	2.2:1
15[e]	$Pd(PPh_3)_4$	–	TMSCl	<5	/

[a] Unless noted otherwise, the reactions were carried out with **1a** (0.10 mmol), **2a** (0.15 mmol), Pd catalyst (5 mol %) and additive (0.02 mmol) in solvent (1.0 mL) for 12 h. [b] Yield was determined by ^{1}H-NMR analysis with CH_2Br_2 as the internal standard. [c] The ratio of **3a**:**4a** was determined by ^{1}H-NMR analysis of the crude reaction mixture. [d] The Pd/ligand complex was pre-prepared with $Pd_2(dba)_3 \cdot CHCl_3$ and ligand in THF at rt for 1 h. [e] The reactions were carried out with additive (0.12 mmol).

Table S2. Further screening of solvents and temperature [a]

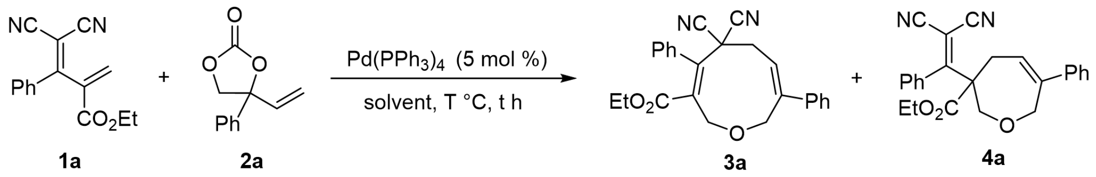

entry	solvent	T (°C)	t (h)	yield (%)[b]	**3a**:**4a**[c]
1	acetone	20	24	30	>20:1
2	ethyl acetate	20	24	84	3.1:1
3	DMSO	20	24	<5	/
4	acetone	60	12	64	12.8:1
5	ethyl acetate	60	12	77	1.8:1
6	DMSO	60	12	83	>20:1
7	DME	60	12	72	>20:1
8	MTBE	60	12	65	2.9:1

[a] Unless noted otherwise, the reactions were carried out with **1a** (0.10 mmol), **2a** (0.15 mmol), and $Pd(PPh_3)_4$ (5 mol %) in solvent (1.0 mL). [b] Yield was determined by ^{1}H-NMR analysis with CH_2Br_2 as the internal standard. [c] The ratio of **3a**:**4a** was determined by ^{1}H-NMR analysis of the crude reaction mixture.

3. Further Substrate Scope Investigation

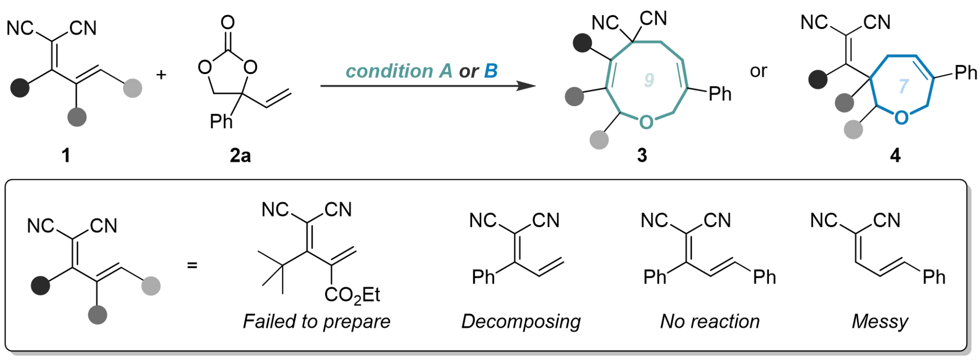

Scheme S1. Further substrate scope investigation of allylidenemalononitriles

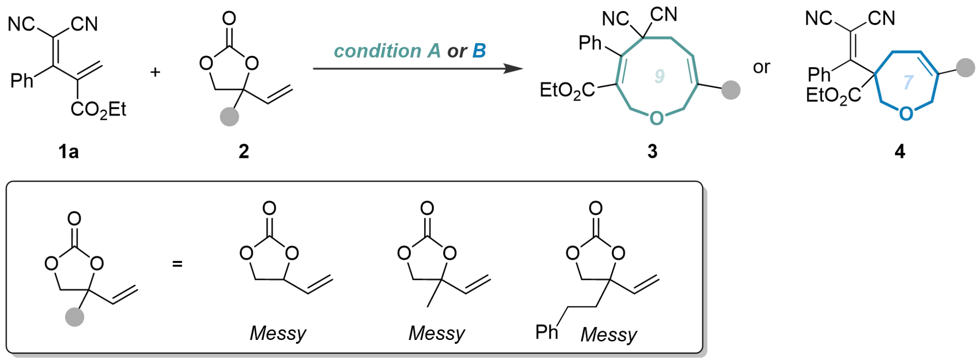

Scheme S2. Further substrate scope investigation of VECs

4. General Procedure for the Preparation of Nine-Membered Products 3

*General procedure for the synthesis of nine-membered products **3***

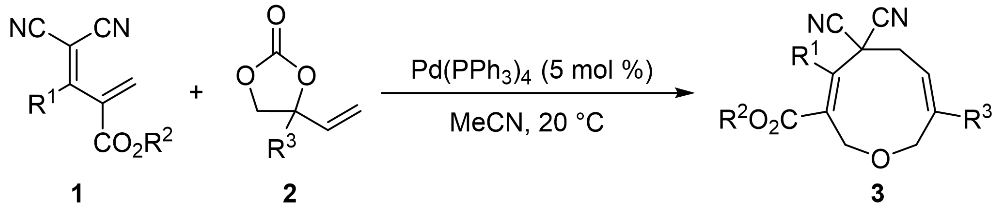

To an over-dried Schlenk tube was added $Pd(PPh_3)_4$ (5 mol %), after which the tube was evacuated and back-filled with argon three times. Subsequently, under the protection of argon, a solution of dried allylidenemalononitriles **1** (0.10 mmol) and vinylethylene carbonates **2** (0.15 mmol) in dry MeCN (1.0 mL) was added *via* syringe and the reaction mixture was stirred at 20 °C for 24 hours. Then the mixture was concentrated and purified by column chromatography on silica gel (petroleum ether/ dichloromethane = 3/1 to 1/1, then petroleum ether/ethyl acetate = 10/1) to afford the corresponding **3** in 51–96% yields, which were dried under vacuum and further analyzed by ^{1}H NMR, ^{13}C NMR, HRMS, *etc*.

*Gram-scale synthesis of the nine-membered product **3a***

To an over-dried 100 mL Schlenk flask, was added $Pd(PPh_3)_4$ (0.20 mmol, 0.23 g), after which the tube was evacuated and back-filled with argon three times. Subsequently, under the protection of argon, a solution of dried allylidenemalononitrile **1a** (4.00 mmol, 1.01 g) and vinylethylene carbonate **2a** (6.00 mmol, 1.14 g) in dry MeCN (40 mL) was added *via* syringe and the reaction mixture was stirred at 20 °C for 24 hours. Then the mixture was concentrated and purified by column chromatography on silica gel (petroleum ether/ dichloromethane = 3/1 to 1/1, then petroleum ether/ethyl acetate = 10/1) to afford **3a** (1.08 g) as white solid in 68% yields.

ethyl-(3*E*,7*Z*)-5,5-dicyano-4,8-diphenyl-2,5,6,9-tetrahydrooxonine-3-carboxylate 3a

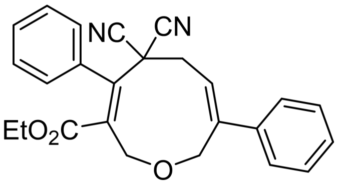

Prepared according to the general procedure to afford **3a** (35.8 mg, m. p. = 131 – 135 °C) in

90% yield as white solid. The regioisomeric ratio was determined to be >20:1 by crude ^{1}H NMR analysis.

NMR and HRMS data for the product **3a**:

^{1}H NMR (600 MHz, CDCl$_3$) δ (ppm): 7.55 (d, *J* = 6.6 Hz, 2H), 7.45 – 7.33 (m, 6H), 7.20 (d, *J* = 8.4 Hz, 2H), 6.25 (t, *J* = 9.0 Hz, 1H), 4.61 (s, 2H), 4.57 (s, 2H), 3.85 (q, *J* = 7.2 Hz, 2H), 3.63 (d, *J* = 9.0 Hz, 2H), 0.80 (t, *J* = 7.2 Hz, 3H).

^{13}C NMR (150 MHz, CDCl$_3$) δ (ppm): 167.6, 143.8, 139.4, 138.9, 137.4, 135.9, 129.7, 128.7, 128.6, 128.1, 126.6, 122.2, 114.2, 66.6, 63.4, 61.5, 40.2, 39.0, 13.4.

HRMS (ESI-TOF) m/z: **[M + Na]$^+$** calculated for $C_{25}H_{22}N_2O_3Na^+$: 421.1523, found: 421.1530.

<u>ethyl-(3*E*,7*Z*)-4-(3-chlorophenyl)-5,5-dicyano-8-phenyl-2,5,6,9-tetrahydrooxonine-3-carboxylate 3b</u>

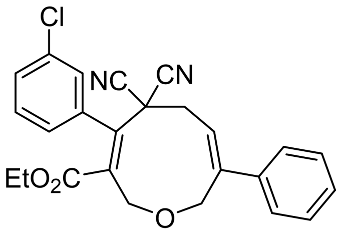

Prepared according to the general procedure to afford **3b** (29.9 mg, m. p. = 142 – 145 °C) in 69% yield as pale yellow solid. The regioisomeric ratio was determined to be >20:1 by crude ^{1}H NMR analysis.

NMR and HRMS data for the product **3b**:

^{1}H NMR (600 MHz, CDCl$_3$) δ (ppm): 7.54 (d, *J* = 7.8 Hz, 2H), 7.41 – 7.36 (m, 4H), 7.33 (t, *J* = 8.4 Hz, 1H), 7.20 (s, 1H), 7.10 (d, *J* = 7.2 Hz, 1H), 6.24 (t, *J* = 9.0 Hz, 1H), 4.61 (s, 2H), 4.56 (d, *J* = 4.8 Hz, 2H), 3.91 (q, *J* = 6.0 Hz, 2H), 3.62 (d, *J* = 11.4 Hz, 2H), 0.87 (t, *J* = 7.2 Hz, 3H).

^{13}C NMR (150 MHz, CDCl$_3$) δ (ppm): 167.2, 143.9, 139.2, 138.8, 137.5, 136.8, 134.6, 130.0, 129.8, 128.74, 128.68, 128.3, 126.6, 126.4, 122.0, 114.0, 66.8, 63.4, 61.7, 39.9, 39.1, 13.8.

HRMS (ESI-TOF) m/z: **[M + Na]$^+$** calculated for $C_{25}H_{21}{}^{35}ClN_2O_3Na^+$: 455.1133, found: 455.1131; calculated for $C_{25}H_{21}{}^{37}ClN_2O_3Na^+$: 457.1103, found: 457.1114.

ethyl-(3*E*,7*Z*)-4-(3-bromophenyl)-5,5-dicyano-8-phenyl-2,5,6,9-tetrahydrooxonine-3-carboxylate 3c

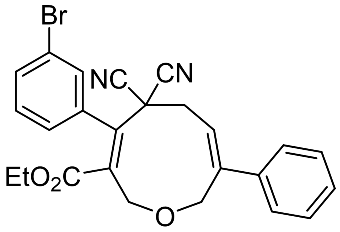

Prepared according to the general procedure for 48 h to afford **3c** (25.3 mg) in 53% yield as yellow semisolid. The regioisomeric ratio was determined to be >20:1 by crude ^{1}H NMR analysis.

NMR and HRMS data for the product **3c**:

^{1}H NMR (600 MHz, CDCl$_3$) δ (ppm): 7.59 – 7.51 (m, 3H), 7.44 – 7.36 (m, 3H), 7.36 – 7.32 (m, 1H), 7.28 (d, *J* = 8.4 Hz, 1H), 7.15 (d, *J* = 7.8 Hz, 1H), 6.23 (t, *J* = 9.0 Hz, 1H), 4.60 (s, 2H), 4.56 (d, *J* = 4.2 Hz, 2H), 3.91 (q, *J* = 7.2 Hz, 2H), 3.62 (dd, *J* = 9.0, 5.4 Hz, 2H), 0.87 (t, *J* = 7.2 Hz, 3H).

^{13}C NMR (150 MHz, CDCl$_3$) δ (ppm): 167.2, 143.9, 139.2, 139.1, 137.7, 136.8, 132.7, 131.1, 130.1, 128.7, 128.8, 126.9, 126.5, 122.5, 122.0, 114.0, 66.8, 63.4, 61.7, 39.9, 39.1, 13.4.

HRMS (ESI-TOF) m/z: **[M + Na]$^+$** calculated for $C_{25}H_{21}{}^{79}BrN_2O_3Na^+$: 499.0628, found: 499.0629; calculated for $C_{25}H_{21}{}^{81}BrN_2O_3Na^+$: 501.0607, found: 501.0607.

ethyl-(3*E*,7*Z*)-5,5-dicyano-8-phenyl-4-(m-tolyl)-2,5,6,9-tetrahydrooxonine-3-carboxylate 3d

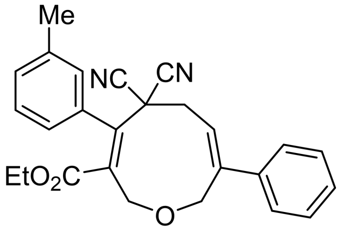

Prepared according to the general procedure to afford **3d** (33.8 mg) in 82% yield as colorless semisolid. The regioisomeric ratio was determined to be >20:1 by crude ^{1}H NMR analysis.

NMR and HRMS data for the product **3d**:

^{1}H NMR (600 MHz, CDCl$_3$) δ (ppm): 7.56 (d, *J* = 6.6 Hz, 2H), 7.43 – 7.34 (m, 3H), 7.27 (t, *J* = 7.2 Hz, 1H), 7.21 (d, *J* = 7.2 Hz, 1H), 7.00 (d, *J* = 5.4 Hz, 2H), 6.27 (t, *J* = 9.0 Hz, 1H), 4.59

(s, 2H), 4.56 (d, *J* = 3.0 Hz, 2H), 3.87 (q, *J* = 7.2 Hz, 2H), 3.61 (d, *J* = 9.0 Hz, 2H), 2.34 (s, 3H), 0.81 (t, *J* = 7.2 Hz, 3H).

^{13}C NMR (150 MHz, CDCl$_3$) δ (ppm): 167.7, 143.5, 139.4, 139.3, 138.4, 137.3, 135.5, 130.4, 128.7, 128.65, 128.58, 128.5, 126.6, 125.1, 122.4, 114.2, 66.2, 63.1, 61.4, 40.2, 39.0, 21.3, 13.4.

HRMS (ESI-TOF) m/z: **[M + Na]$^+$** calculated for $C_{26}H_{24}N_2O_3Na^+$: 435.1679, found: 435.1677.

ethyl-(3*E*,7*Z*)-4-(4-chlorophenyl)-5,5-dicyano-8-phenyl-2,5,6,9-tetrahydrooxonine-3-carboxylate 3e

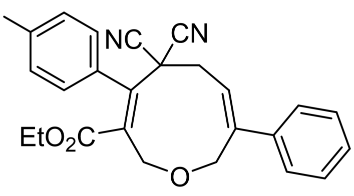

Prepared according to the general procedure for 48 h to afford **3e** (31.2 mg, m. p. = 86 – 89 °C) in 72% yield as pale yellow solid. The regioisomeric ratio was determined to be >20:1 by crude ^{1}H NMR analysis.

NMR and HRMS data for the product **3e**:

^{1}H NMR (600 MHz, CDCl$_3$) δ (ppm): 7.54 (d, *J* = 6.6 Hz, 2H), 7.43 – 7.36 (m, 4H), 7.34 (t, *J* = 7.2 Hz, 1H), 7.20 (brs, 1H), 7.10 (d, *J* = 7.2 Hz, 1H), 6.24 (t, *J* = 9.0 Hz, 1H), 4.61 (s, 2H), 4.56 (d, *J* = 4.2 Hz, 2H), 3.91 (q, *J* = 7.2 Hz, 2H), 3.62 (d, *J* = 9.0 Hz, 2H), 0.87 (t, *J* = 7.2 Hz, 3H).

^{13}C NMR (150 MHz, CDCl$_3$) δ (ppm): 167.2, 143.9, 139.2, 138.8, 136.8, 134.6, 129.8, 128.74, 128.68, 128.3, 126.6, 126.4, 122.0, 114.0, 66.8, 63.4, 61.7, 39.9, 39.1, 13.5.

HRMS (ESI-TOF) m/z: **[M + Na]$^+$** calculated for $C_{25}H_{21}{}^{35}ClN_2O_3Na^+$: 455.1133, found: 455.1128; calculated for $C_{25}H_{21}{}^{37}ClN_2O_3Na^+$: 457.1103, found: 457.1107.

ethyl-(3*E*,7*Z*)-4-(4-bromophenyl)-5,5-dicyano-8-phenyl-2,5,6,9-tetrahydrooxonine-3-carboxylate 3f

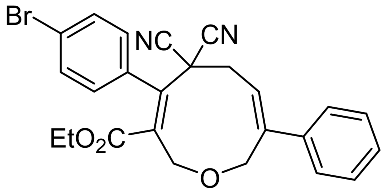

Prepared according to the general procedure to afford **3f** (24.3 mg, m. p. = 121 – 125 °C) in 51% yield as pale yellow solid. The regioisomeric ratio was determined to be >20:1 by crude ^{1}H NMR analysis.

NMR and HRMS data for the product **3f**:

^{1}H NMR (600 MHz, CDCl$_3$) δ (ppm): 7.57 – 7.48 (m, 4H), 7.44 – 7.33 (m, 3H), 7.06 (d, *J* = 8.4 Hz, 2H), 6.19 (t, *J* = 9.0 Hz, 1H), 4.61 (s, 2H), 4.57 (s, 2H), 3.90 (q, *J* = 7.2 Hz, 2H), 3.64 (d, *J* = 9.0 Hz, 2H), 0.88 (t, *J* = 7.2 Hz, 3H).

^{13}C NMR (150 MHz, CDCl$_3$) δ (ppm): 167.3, 144.3, 139.3, 137.7, 136.7, 136.2, 131.8, 129.9, 128.8, 128.7, 126.6, 124.1, 121.9, 114.1, 67.2, 63.8, 61.7, 39.9, 39.2, 13.5.

HRMS (ESI-TOF) m/z: **[M + Na]$^+$** calculated for $C_{25}H_{21}{}^{79}BrN_2O_3Na^+$: 499.0628, found: 499.0626; calculated for $C_{25}H_{21}{}^{81}BrN_2O_3Na^+$: 501.0607, found: 501.0608.

ethyl-(3*E*,7*Z*)-5,5-dicyano-8-phenyl-4-(p-tolyl)-2,5,6,9-tetrahydrooxonine-3-carboxylate 3g

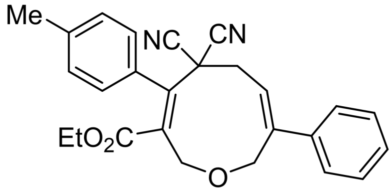

Prepared according to the general procedure to afford **3g** (31.7 mg, m. p. = 129 – 132 °C) in 77% yield as white solid. The regioisomeric ratio was determined to be >20:1 by crude ^{1}H NMR analysis.

NMR and HRMS data for the product **3g**:

^{1}H NMR (600 MHz, CDCl$_3$) δ (ppm): 7.55 (d, *J* = 7.8 Hz, 2H), 7.44 – 7.33 (m, 3H), 7.18 (d, *J* = 7.2 Hz, 2H), 7.08 (d, *J* = 7.8 Hz, 2H), 6.25 (t, *J* = 9.0 Hz, 1H), 4.59 (s, 2H), 4.55 (s, 2H), 3.87 (q, *J* = 7.2 Hz, 2H), 3.60 (d, *J* = 9.0 Hz, 2H), 2.36 (s, 3H), 0.84 (t, *J* = 7.2 Hz, 3H).

^{13}C NMR (150 MHz, CDCl$_3$) δ (ppm): 167.7, 143.6, 139.7, 139.4, 139.1, 135.6, 134.4, 129.2, 128.7, 128.6, 128.0, 126.6, 122.4, 114.3, 66.3, 63.3, 61.5, 40.3, 38.9, 21.4, 13.5.

HRMS (ESI-TOF) m/z: **[M + Na]$^+$** calculated for $C_{26}H_{24}N_2O_3Na^+$: 435.1679, found:

435.1680.

ethyl-(3*E*,7*Z*)-5,5-dicyano-4-(4-methoxyphenyl)-8-phenyl-2,5,6,9-tetrahydrooxonine-3-carboxylate 3h

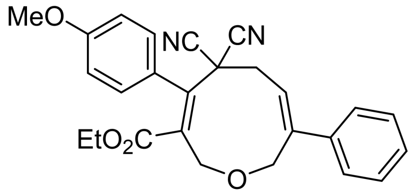

The reaction was performed according to the general procedure by using 0.30 mmol of the corresponding vinylethylene carbonates **2** to afford **3h** (37.7 mg) in 88% yield as pale yelow semisolid. The regioisomeric ratio was determined to be >20:1 by crude ^{1}H NMR analysis.

NMR and HRMS data for the product **3h**:

^{1}H NMR (600 MHz, CDCl$_3$) δ (ppm): 7.55 (d, *J* = 7.2 Hz, 2H), 7.43 – 7.32 (m, 3H), 7.12 (d, *J* = 9.0 Hz, 2H), 6.88 (d, *J* = 8.3 Hz, 2H), 6.24 (t, *J* = 8.4 Hz, 1H), 4.59 (s, 2H), 4.55 (s, 2H), 3.89 (q, *J* = 7.2 Hz, 2H), 3.81 (s, 3H), 3.60 (d, *J* = 9.0 Hz, 2H), 0.87 (t, *J* = 7.8 Hz, 3H).

^{13}C NMR (150 MHz, CDCl$_3$) δ (ppm): 167.8, 160.4, 143.7, 139.4, 138.7, 135.9, 129.6, 129.5, 128.7, 128.6, 126.6, 122.3, 114.3, 113.9, 66.5, 63.4, 61.5, 55.3, 40.4, 39.0, 13.6.

HRMS (ESI-TOF) m/z: **[M + Na]$^+$** calculated for $C_{26}H_{24}N_2O_4Na^+$: 451.1628, found: 451.1631.

ethyl-(3*E*,7*Z*)-5,5-dicyano-4-(naphthalen-2-yl)-8-phenyl-2,5,6,9-tetrahydrooxonine-3-carboxylate 3i

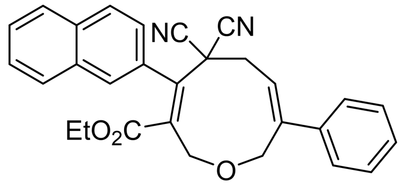

Prepared according to the general procedure to afford **3i** (31.9 mg, m. p. = 165 – 168 °C) in 71% yield as pale yellow solid. The regioisomeric ratio was determined to be >20:1 by crude ^{1}H NMR analysis.

NMR and HRMS data for the product **3i**:

^{1}H NMR (600 MHz, CDCl$_3$) δ (ppm): 7.87 (d, *J* = 7.8 Hz, 1H), 7.84 (d, *J* = 7.8 Hz, 1H), 7.74 (d, *J* = 8.4 Hz, 1H), 7.63 (s, 1H), 7.59 (d, *J* = 6.6 Hz, 2H), 7.56 – 7.48 (m, 2H), 7.46 – 7.37 (m,

3H), 7.34 (dd, J = 8.4, 2.4 Hz, 1H), 6.28 (t, J = 9.0 Hz, 1H), 4.83 – 4.52 (m, 4H), 3.85 – 3.69 (m, 3H), 3.69 – 3.58 (m, 1H), 0.60 (t, J = 7.2 Hz, 3H).

^{13}C NMR (150 MHz, CDCl$_3$) δ (ppm): 167.7, 143.9, 139.4, 139.1, 136.3, 134.8, 133.3, 132.6, 128.7, 128.6, 128.3, 128.2, 127.9, 127.8, 127.2, 126.9, 126.6, 125.2, 122.3, 114.3, 114.2, 66.8, 63.5, 61.5, 40.2, 39.2, 13.3.

HRMS (ESI-TOF) m/z: **[M + Na]$^+$** calculated for $C_{29}H_{24}N_2O_3Na^+$: 471.1679, found: 471.1680.

ethyl-(3*Z*,7*Z*)-5,5-dicyano-8-phenyl-4-(thiophen-2-yl)-2,5,6,9-tetrahydrooxonine-3-carboxylate 3j

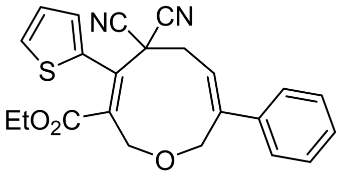

Prepared according to the general procedure to afford **3j** (38.8 mg) in 96% yield as yellow semisolid. The regioisomeric ratio was determined to be >20:1 by crude ^{1}H NMR analysis.

NMR and HRMS data for the product **3j**:

^{1}H NMR (600 MHz, CDCl$_3$) δ (ppm): 7.54 (d, J = 8.4 Hz, 2H), 7.42 (dd, J = 6.6, 1.8 Hz, 1H), 7.41 – 7.33 (m, 3H), 7.06 – 7.01 (m, 2H), 6.23 (t, J = 9.0 Hz, 1H), 4.61 (s, 2H), 4.54 (s, 2H), 3.96 (q, J = 7.2 Hz, 2H), 3.58 (d, J = 9.0 Hz, 2H), 0.94 (t, J = 7.2 Hz, 3H).

^{13}C NMR (150 MHz, CDCl$_3$) δ (ppm): 167.2, 143.9, 139.3, 139.2, 136.3, 131.8, 129.4, 128.65, 128.55, 127.9, 127.0, 126.6, 122.2, 114.0, 67.1, 63.7, 61.7, 40.7, 38.8, 13.6.

HRMS (ESI-TOF) m/z: **[M + Na]$^+$** calculated for $C_{23}H_{20}N_2O_3SNa^+$: 427.1087, found: 427.1086.

methyl-(3*E*,7*Z*)-5,5-dicyano-4,8-diphenyl-2,5,6,9-tetrahydrooxonine-3-carboxylate 3k

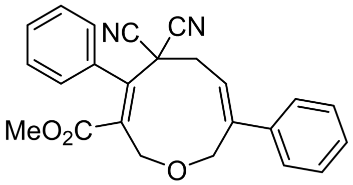

Prepared according to the general procedure to afford **3k** (33.4 mg) in 87% yield as pale yellow semisolid. The regioisomeric ratio was determined to be >20:1 by crude ^{1}H NMR analysis.

NMR and HRMS data for the product **3k**:

^{1}H NMR (600 MHz, CDCl$_3$) δ (ppm): 7.56 (d, J = 8.4 Hz, 2H), 7.45 – 7.35 (m, 6H), 7.20 (d, J = 7.8 Hz, 2H), 6.29 (t, J = 9.0 Hz, 1H), 4.60 (s, 2H), 4.55 (s, 2H), 3.60 (d, J = 9.0 Hz, 2H), 3.38 (s, 3H).

^{13}C NMR (150 MHz, CDCl$_3$) δ (ppm): 168.0, 143.5, 139.5, 139.3, 137.3, 135.6, 129.7, 128.7, 128.6, 127.9, 126.6, 122.4, 114.1, 66.2, 63.0, 52.3, 40.2, 38.9.

HRMS (ESI-TOF) m/z: **[M + Na]$^+$** calculated for $C_{24}H_{20}N_2O_3Na^+$: 407.1366, found: 407.1362.

<u>tert-butyl-(3*E*,7*Z*)-5,5-dicyano-4,8-diphenyl-2,5,6,9-tetrahydrooxonine-3-carboxylate 3l</u>

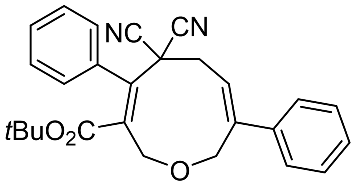

The reaction was performed according to the general procedure by using 0.30 mmol of the corresponding vinylethylene carbonate **2** to afford **3l** (35.0 mg, m. p. = 125 – 129 °C) in 82% yield as white solid. The regioisomeric ratio was determined to be >20:1 by crude ^{1}H NMR analysis.

NMR and HRMS data for the product **3l**:

^{1}H NMR (600 MHz, CDCl$_3$) δ (ppm): 7.56 (d, J = 7.2 Hz, 2H), 7.45 – 7.33 (m, 6H), 7.20 (d, J = 7.2 Hz, 2H), 6.23 (t, J = 9.0 Hz, 1H), 4.58 (s, 2H), 4.57 (s, 2H), 3.62 (d, J = 9.0 Hz, 2H), 1.07 (s, 9H).

^{13}C NMR (150 MHz, CDCl$_3$) δ (ppm): 166.7, 143.8, 139.5, 137.5, 137.3, 136.7, 129.5, 128.7, 128.6, 128.5, 126.6, 125.5, 122.2, 114.3, 82.7, 66.7, 63.7, 40.1, 39.0, 27.3.

HRMS (ESI-TOF) m/z: **[M + Na]$^+$** calculated for $C_{27}H_{26}N_2O_3Na^+$: 449.1836, found: 449.1836.

<u>ethyl-(3*E*,7*Z*)-5,5-dicyano-8-(2-fluorophenyl)-4-phenyl-2,5,6,9-tetrahydrooxonine-3-carboxylate 3m</u>

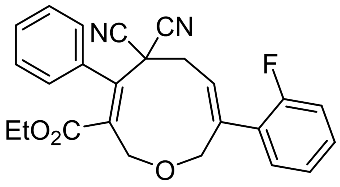

Prepared according to the general procedure to afford **3m** (35.4 mg, m. p. = 109 – 111 °C) in 85% yield as white solid. The regioisomeric ratio was determined to be >20:1 by crude ^{1}H NMR analysis.

NMR and HRMS data for the product **3m**:

^{1}H NMR (600 MHz, CDCl$_3$) δ (ppm): 7.50 – 7.37 (m, 4H), 7.36 – 7.30 (1H), 7.23 (d, J = 6.6 Hz, 2H), 7.17 (t, J = 7.2 Hz, 1H), 7.10 (dd, J = 10.2, 8.4 Hz, 1H), 6.16 (t, J = 9.0 Hz, 1H), 4.63 (s, 2H), 4.54 (s, 2H), 3.84 (q, J = 7.2 Hz, 2H), 3.67 (d, J = 9.0 Hz, 2H), 0.80 (t, J = 7.2 Hz, 3H).

^{13}C NMR (150 MHz, CDCl$_3$) δ (ppm): 167.5, 159.6 (d, $J_{C\text{-}F}$ = 247.1 Hz), 140.2, 138.6, 137.4, 135.9, 130.2 (d, $J_{C\text{-}F}$ = 2.9 Hz), 130.1 (d, $J_{C\text{-}F}$ = 8.7 Hz), 129.7, 128.6, 128.1, 127.4 (d, $J_{C\text{-}F}$ = 12.9 Hz), 125.8, 124.5 (d, $J_{C\text{-}F}$ = 3.0 Hz), 116.0 (d, $J_{C\text{-}F}$ = 21.5 Hz), 114.2, 67.4, 64.1, 61.5, 40.1, 38.7, 13.4.

^{19}F NMR (564 MHz, CDCl$_3$) δ (ppm): -115.0.

HRMS (ESI-TOF) m/z: **[M + Na]$^+$** calculated for $C_{25}H_{21}FN_2O_3Na^+$ 439.1428, found: 439.1427.

<u>ethyl-(3*E*,7*Z*)-8-(3-chlorophenyl)-5,5-dicyano-4-phenyl-2,5,6,9-tetrahydrooxonine-3-carboxylate 3n</u>

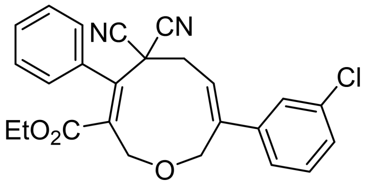

Prepared according to the general procedure to afford **3n** (37.7 mg, m. p. = 89 – 92 °C) in 87% yield as pale yellow solid. The regioisomeric ratio was determined to be >20:1 by crude ^{1}H NMR analysis.

NMR and HRMS data for the product **3n**:

^{1}H NMR (600 MHz, CDCl$_3$) δ (ppm): 7.55 (s, 1H), 7.47 – 7.36 (m, 4H), 7.36 – 7.30 (m, 2H), 7.19 (d, *J* = 8.4 Hz, 2H), 6.27 (t, *J* = 9.0 Hz, 1H), 4.60 (s, 2H), 4.52 (s, 2H), 3.86 (q, *J* = 7.2 Hz, 2H), 3.62 (d, *J* = 9.0 Hz, 2H), 0.80 (t, *J* = 7.2 Hz, 3H).

^{13}C NMR (150 MHz, CDCl$_3$) δ (ppm): 167.5, 142.6, 141.2, 138.8, 137.2, 135.8, 134.7, 130.0, 129.7, 128.6, 128.1, 126.8, 124.8, 123.5, 114.1, 66.4, 63.4, 61.6, 40.0, 38.9, 13.4.

HRMS (ESI-TOF) m/z: **[M + Na]$^+$** calculated for $C_{25}H_{21}{}^{35}ClN_2O_3Na^+$: 455.1133, found: 455.1135; calculated for $C_{25}H_{21}{}^{37}ClN_2O_3Na^+$: 457.1103, found: 457.1115.

ethyl-(3*E*,7*Z*)-8-(3-bromophenyl)-5,5-dicyano-4-phenyl-2,5,6,9-tetrahydrooxonine-3-carboxylate 3o

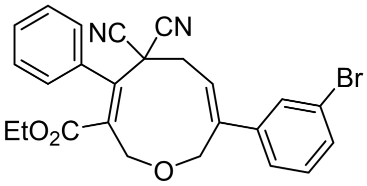

Prepared according to the general procedure to afford **3o** (44.4 mg, m. p. = 104 – 106 °C) in 93% yield as white solid. The regioisomeric ratio was determined to be >20:1 by crude ^{1}H NMR analysis.

NMR and HRMS data for the product **3o**:

^{1}H NMR (600 MHz, CDCl$_3$) δ (ppm): 7.70 (s, 1H), 7.53 – 7.46 (m, 2H), 7.45 – 7.35 (m, 3H), 7.27 (t, *J* = 8.4 Hz, 1H), 7.19 (d, *J* = 7.2 Hz, 2H), 6.26 (t, *J* = 9.0 Hz, 1H), 4.61 (s, 2H), 4.52 (s, 2H), 3.86 (q, *J* = 7.2 Hz, 2H), 3.62 (d, *J* = 9.0 Hz, 2H), 0.80 (t, *J* = 7.2 Hz, 3H).

^{13}C NMR (150 MHz, CDCl$_3$) δ (ppm): 167.5, 142.5, 141.5, 138.8, 137.2, 135.8, 131.6, 130.2, 129.73, 129.69, 128.6, 128.1, 125.2, 123.5, 122.8, 114.1, 66.4, 63.5, 61.6, 40.0, 38.9, 13.4.

HRMS (ESI-TOF) m/z: **[M + Na]$^+$** calculated for $C_{25}H_{21}{}^{79}BrN_2O_3Na^+$: 499.0628, found: 499.0628; calculated for $C_{25}H_{21}{}^{81}BrN_2O_3Na^+$: 501.0607, found: 501.0599.

ethyl-(3*E*,7*Z*)-5,5-dicyano-8-(3-nitrophenyl)-4-phenyl-2,5,6,9-tetrahydrooxonine-3-carboxylate 3p

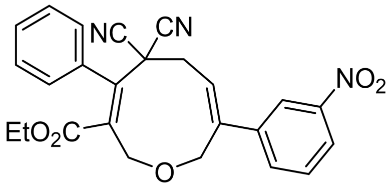

Prepared according to the general procedure to afford **3p** (42.1 mg, m. p. = 145 – 148 °C) in 95% yield as pale yellow solid. The regioisomeric ratio was determined to be >20:1 by crude ^{1}H NMR analysis.

NMR and HRMS data for the product **3p**:

^{1}H NMR (600 MHz, CDCl$_3$) δ (ppm): 8.43 (s, 1H), 8.23 (d, *J* = 8.4 Hz, 1H), 7.92 (d, *J* = 8.4 Hz, 1H), 7.59 (t, *J* = 8.4 Hz, 1H), 7.46 – 7.35 (m, 3H), 7.19 (d, *J* = 6.0 Hz, 2H), 6.39 (t, *J* = 9.0 Hz, 1H), 4.62 (s, 2H), 4.57 (s, 2H), 3.87 (q, *J* = 7.2 Hz, 2H), 3.65 (d, *J* = 8.4 Hz, 2H), 0.79 (t, *J* = 7.2 Hz, 3H).

^{13}C NMR (150 MHz, CDCl$_3$) δ (ppm): 167.4, 148.5, 141.6, 141.1, 138.8, 137.0, 135.8, 132.6, 129.83, 129.81, 128.7, 128.0, 125.0, 123.3, 121.5, 113.9, 66.0, 63.4, 61.7, 39.9, 38.8, 13.4.

HRMS (ESI-TOF) m/z: **[M + Na]$^+$** calculated for $C_{25}H_{21}N_3O_5Na^+$: 466.1373, found: 466.1374.

ethyl-(3*E*,7*Z*)-5,5-dicyano-4-phenyl-8-(m-tolyl)-2,5,6,9-tetrahydrooxonine-3-carboxylate 3q

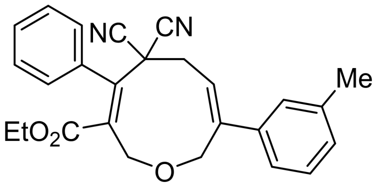

The reaction was performed according to the general procedure by using 0.30 mmol of the corresponding vinylethylene carbonates **2** to afford **3q** (37.5 mg) in 91% yield as colorless semisolid. The regioisomeric ratio was determined to be >20:1 by crude ^{1}H NMR analysis.

NMR and HRMS data for the product **3q**:

^{1}H NMR (600 MHz, CDCl$_3$) δ (ppm): 7.46 – 7.34 (m, 5H), 7.28 (t, *J* = 8.4 Hz, 1H), 7.23 – 7.16 (m, 3H), 6.26 (t, *J* = 8.4 Hz, 1H), 4.61 (s, 2H), 4.55 (s, 2H), 3.86 (q, *J* = 7.2 Hz, 2H), 3.61 (q, *J* = 9.0 Hz, 2H), 2.39 (s, 3H), 0.80 (t, *J* = 7.2 Hz, 3H).

^{13}C NMR (150 MHz, CDCl$_3$) δ (ppm): 167.6, 143.7, 139.4, 139.0, 138.3, 137.4, 135.8, 129.6, 129.3, 128.6, 128.1, 127.3, 123.7, 122.1, 114.2, 66.4, 63.2, 61.5, 40.2, 39.0, 21.5, 13.4.

HRMS (ESI-TOF) m/z: **[M + Na]$^+$** calculated for $C_{26}H_{24}N_2O_3Na^+$: 435.1679, found: 435.1674.

ethyl-(3*E*,7*Z*)-5,5-dicyano-8-(3-methoxyphenyl)-4-phenyl-2,5,6,9-tetrahydrooxonine-3-carboxylate 3r

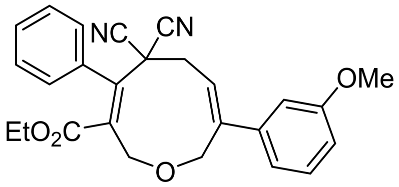

Prepared according to the general procedure to afford **3r** (34.7 mg, m. p. = 101 – 106 °C) in 81% yield as white solid. The regioisomeric ratio was determined to be >20:1 by crude ^{1}H NMR analysis.

NMR and HRMS data for the product **3r**:

^{1}H NMR (600 MHz, CDCl$_3$) δ (ppm): 7.44 – 7.35 (m, 3H), 7.31 (t, *J* = 8.4 Hz, 1H), 7.20 (d, *J* = 6.0 Hz, 2H), 7.13 (d, *J* = 8.4 Hz, 1H), 7.10 (s, 1H), 6.91 (d, *J* = 7.2 Hz, 1H), 6.24 (t, *J* = 9.0 Hz, 1H), 4.61 (s, 2H), 4.55 (s, 2H), 3.85 (q, *J* = 7.2 Hz, 2H), 3,84 (s, 3H), 3.62 (d, *J* = 9.0 Hz, 2H), 0.79 (t, *J* = 7.2 Hz, 3H).

^{13}C NMR (150 MHz, CDCl$_3$) δ (ppm): 167.6, 159.8, 143.8, 140.9, 138.9, 137.4, 135.9, 129.7, 129.6, 128.6, 128.1, 122.4, 119.0, 114.2, 114.0, 112.4, 66.7, 63.5, 61.5, 55.3, 40.2, 39.0, 13.4.

HRMS (ESI-TOF) m/z: **[M + Na]$^+$** calculated for $C_{26}H_{24}N_2O_4Na^+$: 451.1628, found: 451.1631.

ethyl-(3*E*,7*Z*)-5,5-dicyano-8-(4-fluorophenyl)-4-phenyl-2,5,6,9-tetrahydrooxonine-3-carboxylate 3s

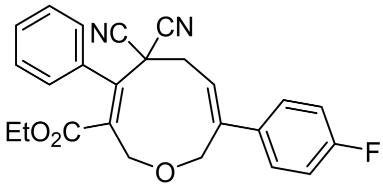

Prepared according to the general procedure to afford **3s** (35.8 mg, m. p. = 115 – 117 °C) in 86% yield as pale yellow solid. The regioisomeric ratio was determined to be >20:1 by crude ^{1}H NMR analysis.

NMR and HRMS data for the product **3s**:

^{1}H NMR (600 MHz, CDCl$_3$) δ (ppm): 7.57 – 7.50 (m, 2H), 7.44 – 7.35 (m, 3H), 7.19 (d, *J* = 7.2 Hz, 2H), 7.08 (t, *J* = 7.8 Hz, 2H), 6.20 (t, *J* = 8.4 Hz, 1H), 4.60 (s, 2H), 4.53 (s, 2H), 3.85 (q, *J* = 7.8 Hz, 2H), 3.61 (d, *J* = 9.0 Hz, 2H), 0.80 (t, *J* = 7.2 Hz, 3H).

^{13}**C NMR (150 MHz**, **CDCl$_3$)** δ (ppm): 167.6, 163.0 (d, J_{C-F} = 247.1 Hz), 142.8, 138.9, 137.3, 135.8, 135.5 (d, J_{C-F} = 2.9 Hz), 129.7, 128.6, 128.4 (d, J_{C-F} = 8.7 Hz), 128.1, 122.3, 115.6 (d, J_{C-F} = 21.6 Hz), 114.1, 66.5, 63.4, 61.5, 40.2, 39.0, 13.4.

^{19}**F NMR (564 MHz**, **CDCl$_3$)** δ (ppm): -112.9.

HRMS (ESI-TOF) m/z: **[M + Na]$^+$** calculated for $C_{25}H_{21}FN_2O_3Na^+$: 439.1428, found: 439.1427.

ethyl-(3*E*,7*Z*)-8-(4-chlorophenyl)-5,5-dicyano-4-phenyl-2,5,6,9-tetrahydrooxonine-3-carboxylate 3t

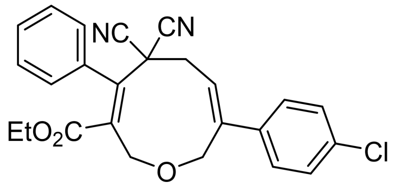

Prepared according to the general procedure to afford **3t** (35.1 mg) in 81% yield as colorless semisolid. The regioisomeric ratio was determined to be >20:1 by crude ^{1}H NMR analysis.

NMR and HRMS data for the product **3t**:

^{1}**H NMR (600 MHz**, **CDCl$_3$)** δ (ppm): 7.49 (d, J = 8.4 Hz, 2H), 7.44 – 7.34 (m, 5H), 7.18 (d, J = 7.2 Hz, 2H), 6.24 (t, J = 9.0 Hz, 1H), 4.59 (s, 2H), 4.53 (s, 2H), 3.85 (q, J = 7.2 Hz, 2H), 3.61 (d, J = 9.0 Hz, 2H), 0.79 (t, J = 7.2 Hz, 3H).

^{13}**C NMR (150 MHz**, **CDCl$_3$)** δ (ppm): 167.6, 142.7, 138.9, 137.8, 137.3, 135.8, 134.6, 129.7, 128.9, 128.6, 128.1, 127.9, 122.8, 114.1, 66.4, 63.4, 61.6, 40.1, 39.0, 13.4.

HRMS (ESI-TOF) m/z: **[M + Na]$^+$** calculated for $C_{25}H_{21}{}^{35}ClN_2O_3Na^+$: 455.1133, found: 455.1135; calculated for $C_{25}H_{21}{}^{37}ClN_2O_3Na^+$: 457.1103, found: 457.1112.

ethyl-(3*E*,7*Z*)-8-(4-bromophenyl)-5,5-dicyano-4-phenyl-2,5,6,9-tetrahydrooxonine-3-carboxylate 3u

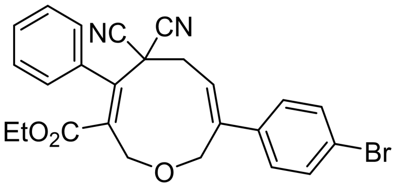

Prepared according to the general procedure to afford **3u** (36.7 mg, m. p. = 149 – 152 °C) in 77% yield as white solid. The regioisomeric ratio was determined to be >20:1 by crude ^{1}H

NMR analysis.

NMR and HRMS data for the product **3u**:

^{1}H NMR (600 MHz, CDCl$_3$) δ (ppm): 7.52 (d, *J* = 9.0 Hz, 2H), 7.46 – 7.36 (m, 5H), 7.18 (d, *J* = 7.2 Hz, 2H), 6.24 (t, *J* = 9.0 Hz, 1H), 4.59 (s, 2H), 4.52 (s, 2H), 3.85 (q, *J* = 7.2 Hz, 2H), 3.61 (d, *J* = 9.0 Hz, 2H), 0.79 (t, *J* = 7.2 Hz, 3H).

^{13}C NMR (150 MHz, CDCl$_3$) δ (ppm): 167.6, 142.7, 138.9, 138.3, 137.2, 135.8, 131.8, 129.7, 128.6, 128.2, 128.1, 122.84, 122.81, 114.1, 66.3, 63.4, 61.6, 40.0, 39.0, 13.4.

HRMS (ESI-TOF) m/z: **[M + Na]$^+$** calculated for $C_{25}H_{21}{}^{79}BrN_2O_3Na^+$: 499.0628, found: 499.0632; calculated for $C_{25}H_{21}{}^{81}BrN_2O_3Na^+$: 501.0607, found: 501.0609.

ethyl-(3*E*,7*Z*)-5,5-dicyano-4-phenyl-8-(p-tolyl)-2,5,6,9-tetrahydrooxonine-3-carboxylate 3v

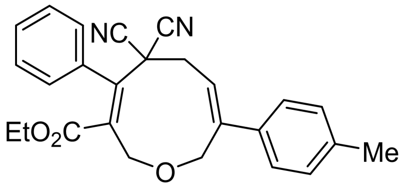

Prepared according to the general procedure to afford **3v** (33.8 mg, m. p. = 110 – 115 °C) in 82% yield as pale yellow solid. The regioisomeric ratio was determined to be >20:1 by crude ^{1}H NMR analysis.

NMR and HRMS data for the product **3v**:

^{1}H NMR (600 MHz, CDCl$_3$) δ (ppm): 7.45 (d, *J* = 8.4 Hz, 2H), 7.43 – 7.36 (m, 3H), 7.23 – 7.18 (m, 4H), 6.24 (t, *J* = 9.0 Hz, 1H), 4.59 (s, 2H), 4.54 (s, 2H), 3.85 (q, *J* = 7.8 Hz, 2H), 3.60 (d, *J* = 9.0 Hz, 2H), 2.38 (s, 3H), 0.80 (t, *J* = 7.2 Hz, 3H).

^{13}C NMR (150 MHz, CDCl$_3$) δ (ppm): 167.6, 143.4, 139.0, 138.6, 137.4, 136.4, 135.8, 129.6, 129.4, 128.6, 128.1, 126.5, 121.5, 114.2, 66.3, 63.2, 61.5, 40.2, 39.0, 21.2, 13.4.

HRMS (ESI-TOF) m/z: **[M + Na]$^+$** calculated for $C_{26}H_{24}N_2O_3Na^+$: 435.1679, found: 435.1682.

ethyl-(3*E*,7*Z*)-5,5-dicyano-8-(4-ethylphenyl)-4-phenyl-2,5,6,9-tetrahydrooxonine-3-carboxylate 3w

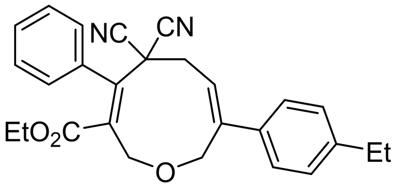

Prepared according to the general procedure to afford **3w** (38.9 mg, m. p. = 94 – 97 °C) in 91% yield as white solid. The regioisomeric ratio was determined to be >20:1 by crude ^{1}H NMR analysis.

NMR and HRMS data for the product **3w**:

^{1}H NMR (600 MHz, CDCl$_3$) δ (ppm): 7.48 (d, *J* = 7.8 Hz, 2H), 7.44 – 7.35 (m, 3H), 7.25 – 7.19 (m, 4H), 6.25 (t, *J* = 9.0 Hz, 1H), 4.60 (s, 2H), 4.55 (s, 2H), 3.85 (q, *J* = 7.2 Hz, 2H), 3.60 (d, *J* = 8.4 Hz, 2H), 2.67 (q, *J* = 7.8 Hz, 2H), 1.26 (t, *J* = 7.8 Hz, 3H), 0.80 (t, *J* = 7.2 Hz, 3H).

^{13}C NMR (150 MHz, CDCl$_3$) δ (ppm): 167.6, 144.9, 143.4, 139.0, 137.4, 136.7, 135.8, 129.6, 128.6, 128.2, 128.1, 126.6, 121.5, 114.2 66.3, 63.2, 61.5, 40.3, 39.0, 28.5, 15.4, 13.4.

HRMS (ESI-TOF) m/z: **[M + Na]$^+$** calculated for $C_{27}H_{26}N_2O_3Na^+$: 449.1836, found: 449.1836.

<u>ethyl-(3*E*,7*Z*)-5,5-dicyano-8-(4-methoxyphenyl)-4-phenyl-2,5,6,9-tetrahydrooxonine-3-carboxylate 3x</u>

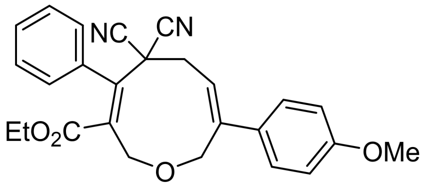

Prepared according to the general procedure to afford **3x** (34.7 mg) in 81% yield as pale yellow semisolid. The regioisomeric ratio was determined to be >20:1 by crude ^{1}H NMR analysis.

NMR and HRMS data for the product **3x**:

^{1}H NMR (600 MHz, CDCl$_3$) δ (ppm): 7.51 (d, *J* = 9.0 Hz, 2H), 7.44 – 7.35 (m, 3H), 7.20 (d, *J* = 6.6 Hz, 2H), 6.92 (d, *J* = 9.0 Hz, 2H), 6.21 (t, *J* = 9.0 Hz, 1H), 4.59 (s, 2H), 4.53 (s, 2H), 3.85 (q, *J* = 7.2 Hz, 2H), 3.84 (s, 3H), 3.59 (d, *J* = 9.0 Hz, 2H), 0.80 (t, *J* = 6.6 Hz, 3H).

^{13}C NMR (150 MHz, CDCl$_3$) δ (ppm): 167.7, 159.9, 142.8, 139.0, 137.4, 135.8, 131.7, 129.6, 128.6, 128.1, 127.9, 120.7, 114.2, 114.0, 66.2, 63.1, 61.5, 55.3, 40.3, 39.1, 13.4.

HRMS (ESI-TOF) m/z: **[M + Na]$^+$** calculated for $C_{26}H_{24}N_2O_4Na^+$: 451.1628, found: 451.1629.

ethyl-(3*E*,7*Z*)-5,5-dicyano-8-(3,4-dichlorophenyl)-4-phenyl-2,5,6,9-tetrahydrooxonine-3-carboxylate 3y

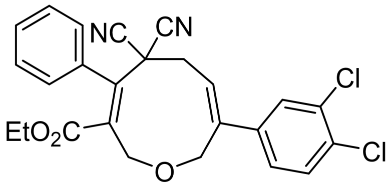

Prepared according to the general procedure to afford **3y** (36.4 mg, m. p. = 118 – 121 °C) in 78% yield as white solid. The regioisomeric ratio was determined to be >20:1 by crude ^{1}H NMR analysis.

NMR and HRMS data for the product **3y**:

^{1}H NMR (600 MHz, CDCl$_3$) δ (ppm): 7.66 (d, *J* = 2.4 Hz, 1H), 7.46 (d, *J* = 8.4 Hz, 1H), 7.44 – 7.36 (m, 4H), 7.18 (d, *J* = 6.6 Hz, 2H), 6.27 (t, *J* = 9.0 Hz, 1H), 4.59 (s, 2H), 4.50 (s, 2H), 3.86 (q, *J* = 7.2 Hz, 2H), 3.61 (d, *J* = 9.6 Hz, 2H), 0.80 (t, *J* = 7.2 Hz, 3H).

^{13}C NMR (150 MHz, CDCl$_3$) δ (ppm): 167.5, 141.6, 139.4, 138.8, 137.1, 135.8, 132.9, 132.8, 130.6, 129.8, 128.65, 128.59, 128.1, 125.9, 123.8, 114.0, 66.1, 63.4, 61.6, 40.0, 38.9, 13.4.

HRMS (ESI-TOF) m/z: **[M + Na]$^+$** calculated for $C_{25}H_{20}{}^{35}Cl_2N_2O_3Na^+$: 489.0743, found: 489.0740; calculated for $C_{25}H_{20}{}^{35}Cl^{37}ClN_2O_3Na^+$: 491.0714, found: 491.0717; calculated for $C_{25}H_{20}{}^{37}Cl_2N_2O_3Na^+$: 493.0684, found: 493.0689.

ethyl-(3*E*,7*Z*)-5,5-dicyano-8-(naphthalen-2-yl)-4-phenyl-2,5,6,9-tetrahydrooxonine-3-carboxylate 3z

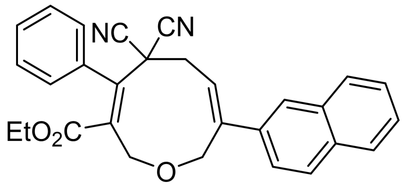

Prepared according to the general procedure to afford **3z** (40.9 mg, m. p. = 116 – 118 °C) in 91% yield as white solid. The regioisomeric ratio was determined to be >20:1 by crude ^{1}H NMR analysis.

NMR and HRMS data for the product **3z**:

^{1}H NMR (600 MHz, CDCl$_3$) δ (ppm): 8.04 (s, 1H), 7.92 – 7.82 (m, 3H), 7.68 (d, *J* = 10.2 Hz, 1H), 7.56 – 7.47 (m, 2H), 7.44 – 7.35 (m, 3H), 7.21 (d, *J* = 6.0 Hz, 2H), 6.41 (t, *J* = 9.0 Hz,

1H), 4.68 (s, 2H), 4.66 (s, 2H), 3.86 (q, *J* = 7.8 Hz, 2H), 3.68 (d, *J* = 8.4 Hz, 2H), 0.79 (t, *J* = 7.2 Hz, 3H).

^{13}C NMR (150 MHz, CDCl$_3$) δ (ppm): 167.6, 143.4, 138.9, 137.4, 136.5, 135.9, 133.3, 133.1, 129.7, 128.8, 128.6, 128.4, 128.1, 127.6, 126.6, 126.5, 126.0, 124.2, 122.7, 114.2, 66.4, 63.4, 61.5, 40.2, 39.2, 13.4.

HRMS (ESI-TOF) m/z: **[M + Na]$^+$** calculated for $C_{29}H_{24}N_2O_3Na^+$: 471.1679, found: 471.1680.

ethyl-(3*E*,7*E*)-5,5-dicyano-4-phenyl-8-(thiophen-2-yl)-2,5,6,9-tetrahydrooxonine-3-carboxylate 3aa

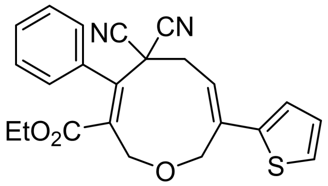

Prepared according to the general procedure to afford **3aa** (25.5 mg, m. p. = 146 – 150 °C) in 63% yield as pale yellow solid. The regioisomeric ratio was determined to be >20:1 by crude ^{1}H NMR analysis.

NMR and HRMS data for the product **3aa**:

^{1}H NMR (600 MHz, CDCl$_3$) δ (ppm): 7.44 – 7.36 (m, 3H), 7.29 (d, *J* = 4.2 Hz, 1H), 7.25 (d, *J* = 3.6 Hz, 1H), 7.21 (d, *J* = 6.6 Hz, 2H), 7.05 (dd, *J* = 4.8, 3.6 Hz, 1H), 6.36 (t, *J* = 9.0 Hz, 1H), 4.60 (s, 2H), 4.58 (s, 2H), 3.83 (q, *J* = 7.2 Hz, 2H), 3.60 (d, *J* = 9.0 Hz, 2H), 0.77 (t, *J* = 7.2 Hz, 3H).

^{13}C NMR (150 MHz, CDCl$_3$) δ (ppm): 167.5, 142.1, 139.2, 137.3, 137.2, 135.7, 129.7, 128.6, 128.1, 127.9, 125.9, 125.7, 120.0, 114.1, 65.8, 63.4, 61.5, 40.2, 39.0, 13.4.

HRMS (ESI-TOF) m/z: **[M + Na]$^+$** calculated for $C_{23}H_{20}N_2O_3SNa^+$: 427.1087, found: 427.1089.

5. General Procedure for the Preparation of Seven-Membered Products 4

*General procedure for the synthesis of seven-membered products **4***

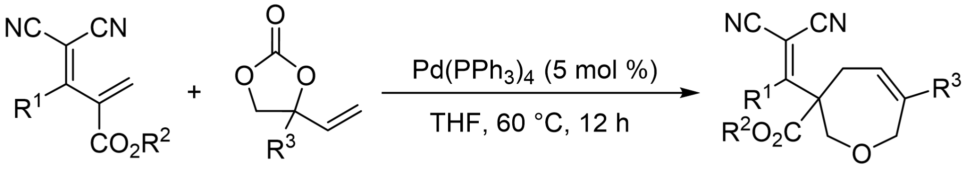

1 2 4

To an over-dried Schlenk tube was added $Pd(PPh_3)_4$ (5 mol %), after which the tube was evacuated and back-filled with argon three times. Subsequently, under the protection of argon, a solution of dried allylidenemalononitriles **1** (0.10 mmol) and vinylethylene carbonates **2** (0.15 mmol) in dry THF (1.0 mL) was added via syringe and the reaction mixture was stirred at 60 °C for 12 hours. Then the mixture was concentrated and purified by column chromatography on silica gel (petroleum ether/ dichloromethane = 3/1 to 1/1, then petroleum ether/ethyl acetate = 10/1) to afford the corresponding **4** in 71–94% yields, which were dried under vacuum and further analyzed by ^{1}H NMR, ^{13}C NMR, HRMS, *etc.*

*Gram-scale synthesis of the seven-membered product **4a***

To an over-dried 100 mL Schlenk flask, was added $Pd(PPh_3)_4$ (0.20 mmol, 231 mg), after which the tube was evacuated and back-filled with argon three times. Subsequently, under the protection of argon, a solution of dried allylidenemalononitrile **1a** (4.00 mmol, 1.01 g) and vinylethylene carbonate **2a** (6.00 mmol, 1.14 g) in dry THF (40 mL) was added via syringe and the reaction mixture was stirred at 60 °C for 12 hours. Then the mixture was concentrated and purified by column chromatography on silica gel (petroleum ether/ dichloromethane = 3/1 to 1/1, then petroleum ether/ethyl acetate = 10/1) to afford **4a** (1.16 g) as pale yellow solid in 73% yields.

ethyl-3-(2,2-dicyano-1-phenylvinyl)-6-phenyl-2,3,4,7-tetrahydrooxepine-3-carboxylate 4a

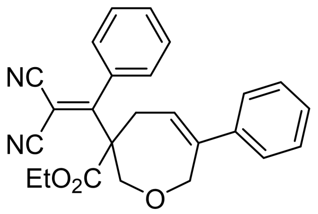

Prepared according to the general procedure to afford **4a** (33.4 mg, m. p. = 104 – 108 °C) in

84% yield as pale yellow solid. The regioisomeric ratio was determined to be >20:1 by crude ^{1}H NMR analysis.

NMR and HRMS data for the product **4a**:

^{1}H NMR (600 MHz, **CDCl$_3$)** δ (ppm): 7.55 – 7.44 (m, 3H), 7.34 – 7.20 (m, 5H), 7.09 (d, *J* = 6.6 Hz, 2H), 5.50 (t, *J* = 6.6 Hz, 1H), 4.69 (d, *J* = 16.2 Hz, 1H), 4.64 (d, *J* = 16.2 Hz, 1H), 4.55 (d, *J* = 13.2 Hz, 1H), 4.39 – 4.25 (m, 2H), 4.18 (d, *J* = 12.0 Hz, 1H), 3.01 (dd, *J* = 14.4, 6.0 Hz, 1H), 2.94 (dd, *J* = 15.0, 6.6 Hz, 1H), 1.35 (t, *J* = 6.6 Hz, 3H).

^{13}C NMR (150 MHz, **CDCl$_3$)** δ (ppm): 180.7, 170.5, 141.4, 139.1, 135.5, 130.4, 128.8, 128.5, 127.7, 126.7, 125.6, 122.2, 111.8, 111.3, 90.2, 75.0, 74.3, 62.7, 60.9, 32.5, 14.1.

HRMS (ESI-TOF) m/z: **[M + Na]$^+$** calculated for $C_{25}H_{22}N_2O_3Na^+$: 421.1523, found: 421.1521.

ethyl-3-(1-(3-chlorophenyl)-2,2-dicyanovinyl)-6-phenyl-2,3,4,7-tetrahydrooxepine-3-carboxylate 4b

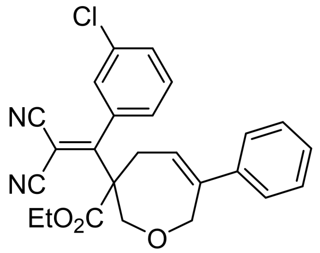

Prepared according to the general procedure to afford **4b** (35.5 mg) in 82% yield as pale yellow semisolid. The regioisomeric ratio was determined to be >20:1 by crude ^{1}H NMR analysis.

NMR and HRMS data for the product **4b**:

^{1}H NMR (600 MHz, **CDCl$_3$)** δ (ppm): 7.40 (d, *J* = 7.8 Hz, 2H), 7.30 – 7.19 (m, 3H), 7.13 (d, *J* = 8.4 Hz, 2H), 7.06 (d, *J* = 8.4 Hz, 2H), 5.53 (t, *J* = 6.6 Hz, 1H), 4.62 (d, *J* = 15.6 Hz, 1H), 4.58 (d, *J* = 15.6 Hz, 1H), 4.40 (d, *J* = 13.2 Hz, 1H), 4.34 – 4.22 (m, 2H), 4.17 (d, *J* = 13.2 Hz, 1H), 2.98 (dd, *J* = 14.4, 6.6 Hz, 1H), 2.88 (dd, *J* = 14.4, 6.0 Hz, 1H), 1.30 (t, *J* = 6.6 Hz, 3H).

^{13}C NMR (150 MHz, **CDCl$_3$)** δ (ppm): 179.7, 170.2, 142.0, 139.1, 136.8, 133.9, 129.2, 128.7, 128.6, 128.3, 127.9, 126.6, 125.6, 122.2, 111.6, 111.1, 90.7, 74.9, 74.7, 62.7, 60.5, 32.7, 14.1.

HRMS (ESI-TOF) m/z: **[M + Na]$^+$** calculated for $C_{25}H_{21}{}^{35}ClN_2O_3Na^+$: 455.1133, found: 455.1131; calculated for $C_{25}H_{21}{}^{37}ClN_2O_3Na^+$: 457.1103, found: 457.1108.

ethyl-3-(1-(3-bromophenyl)-2,2-dicyanovinyl)-6-phenyl-2,3,4,7-tetrahydrooxepine-3-carboxylate 4c

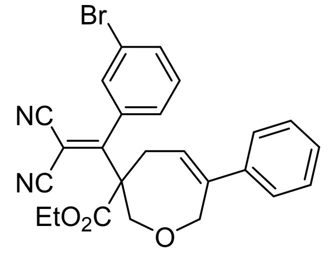

Prepared according to the general procedure to afford **4c** (33.9 mg, m. p. = 106 – 111 °C) in 71% yield as pale yellow solid. The regioisomeric ratio was determined to be >20:1 by crude ^{1}H NMR analysis.

NMR and HRMS data for the product **4c**:

^{1}H NMR (600 MHz, CDCl$_3$) δ (ppm): 7.64 (d, *J* = 8.4 Hz, 1H), 7.41 – 7.27 (m, 5H), 7.18 (d, *J* = 6.6 Hz, 1H), 7.12 (d, *J* = 6.6 Hz, 2H), 5.52 (brs, 1H), 4.66 (brs, 2H), 4.49 (d, *J* = 12.6 Hz, 1H), 4.39 – 4.27 (m, 2H), 4.22 (d, *J* = 13.2 Hz, 1H), 3.01 (dd, *J* = 14.4, 6.6 Hz, 1H), 2.95 (dd, *J* = 14.4, 6.6 Hz, 1H), 1.37 (t, *J* = 7.2 Hz, 3H).

^{13}C NMR (150 MHz, CDCl$_3$) δ (ppm): 179.0, 170.2, 142.1, 139.0, 137.3, 133.4, 130.3, 129.6, 128.6, 127.9, 125.7, 125.5, 122.8, 122.1, 111.4, 111.0, 91.0, 74.9, 74.7, 62.9, 60.4, 32.7, 14.1.

HRMS (ESI-TOF) m/z: **[M + Na]$^+$** calculated for $C_{25}H_{21}{}^{79}BrN_2O_3Na^+$: 499.0628, found: 499.0633; calculated for $C_{25}H_{21}{}^{81}BrN_2O_3Na^+$: 501.0607, found: 501.0607.

ethyl-3-(2,2-dicyano-1-(m-tolyl)vinyl)-6-phenyl-2,3,4,7-tetrahydrooxepine-3-carboxylate 4d

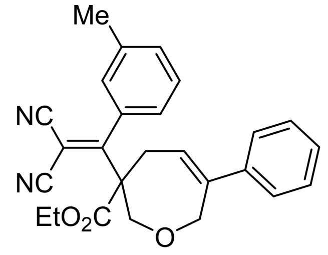

The reaction was performed according to the general procedure at 80 °C to afford **4d** (26.0 mg) in 63% yield as pale yellow semisolid. The regioisomeric ratio was determined to be >20:1 by crude ^{1}H NMR analysis.

NMR and HRMS data for the product **4d**:

^{1}H NMR (600 MHz, CDCl$_3$) δ (ppm): 7.37 (t, *J* = 7.8 Hz, 1H), 7.33 – 7.26 (m, 4H), 7.08 (d, *J* = 7.2 Hz, 2H), 7.05 – 6.94 (m, 2H), 5.46 (brs, 1H), 4.71 (d, *J* = 15.6 Hz, 1H), 4.63 (d, *J* = 16.8 Hz, 1H), 4.58 (d, *J* = 12.6 Hz, 1H), 4.37 – 4.27 (m, 2H), 4.15 (d, *J* = 12.0 Hz, 1H), 3.04 – 2.89 (m, 2H), 2.37 (s, 3H), 1.35 (t, *J* = 7.2 Hz, 3H).

^{13}C NMR (150 MHz, CDCl$_3$) δ (ppm): 181.0, 170.6, 141.2, 139.1, 138.7, 135.5, 131.1, 128.7, 128.5, 127.7, 127.2, 125.6, 123.7, 122.3, 111.8, 111.4, 89.9, 75.0, 74.2, 62.7, 61.0, 32.4, 21.4, 14.1.

HRMS (ESI-TOF) m/z: **[M + Na]$^+$** calculated for $C_{26}H_{24}N_2O_3Na^+$: 435.1679, found: 435.1677.

ethyl-3-(1-(4-chlorophenyl)-2,2-dicyanovinyl)-6-phenyl-2,3,4,7-tetrahydrooxepine-3-carboxylate 4e

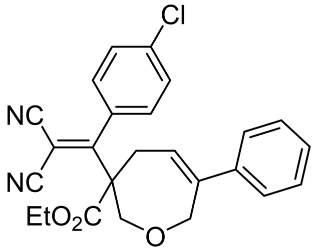

The reaction was performed according to the general procedure at 80 °C to afford **4e** (23.4 mg) in 54% yield as yellow semisolid. The regioisomeric ratio was determined to be >20:1 by crude ^{1}H NMR analysis.

NMR and HRMS data for the product **4e**:

^{1}H NMR (600 MHz, CDCl$_3$) δ (ppm): 7.46 (d, *J* = 8.4 Hz, 2H), 7.34 – 7.27 (m, 3H), 7.18 (d, *J* = 8.4 Hz, 2H), 7.11 (d, *J* = 6.6 Hz, 2H), 5.59 (t, *J* = 6.0 Hz, 1H), 4.67 (d, *J* = 16.2 Hz, 1H), 4.64 (d, *J* = 16.8 Hz, 1H), 4.46 (d, *J* = 13.2 Hz, 1H), 4.38 – 4.27 (m, 2H), 4.22 (d, *J* = 12.6 Hz, 1H), 3.03 (dd, *J* = 13.8, 6.6 Hz, 1H), 2.93 (dd, *J* = 14.4, 7.2 Hz, 1H), 1.35 (t, *J* = 7.2 Hz, 3H).

^{13}C NMR (150 MHz, CDCl$_3$) δ (ppm): 179.7, 170.2, 141.9, 139.0, 136.8, 133.9, 129.2, 128.6, 128.3, 127.9, 125.6, 122.2, 111.6, 111.1, 90.7, 74.9, 74.7, 62.9, 60.5, 32.7, 14.1.

HRMS (ESI-TOF) m/z: **[M + Na]$^+$** calculated for $C_{25}H_{21}{}^{35}ClN_2O_3Na^+$: 455.1133, found: 455.1130; calculated for $C_{25}H_{21}{}^{37}ClN_2O_3Na^+$: 457.1103, found: 457.1107.

ethyl-3-(1-(4-bromophenyl)-2,2-dicyanovinyl)-6-phenyl-2,3,4,7-tetrahydrooxepine-3-carboxylate 4f

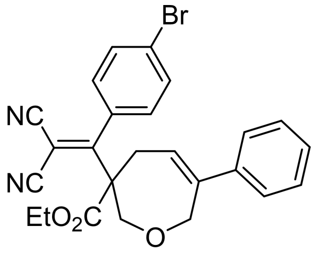

The reaction was performed according to the general procedure at 100 °C to afford **4f** (20.0 mg) in 42% yield as pale yellow semisolid. The regioisomeric ratio was determined to be >20:1 by crude ^{1}H NMR analysis.

NMR and HRMS data for the product **4f**:

^{1}H NMR (600 MHz, CDCl$_3$) δ (ppm): 7.62 (d, J = 8.4 Hz, 2H), 7.34 – 7.27 (m, 3H), 7.16 – 7.06 (m, 4H), 5.59 (t, J = 6.6 Hz, 1H), 4.67 (d, J = 16.8 Hz, 1H), 4.64 (d, J = 16.8 Hz, 1H), 4.45 (d, J = 12.0 Hz, 1H), 4.37 – 4.27 (m, 2H), 4.22 (d, J = 12.6 Hz, 1H), 3.03 (dd, J = 14.4, 6.0 Hz, 1H), 2.93 (dd, J = 14.4, 6.0 Hz, 1H), 1.35 (t, J = 7.2 Hz, 3H).

^{13}C NMR (150 MHz, CDCl$_3$) δ (ppm): 179.7, 170.2, 142.0, 139.0, 134.4, 132.1, 128.6, 128.4, 127.9, 125.6, 125.0, 122.2, 111.6, 111.1, 90.7, 74.9, 74.7, 62.9, 60.5, 32.7, 14.1.

HRMS (ESI-TOF) m/z: **[M + Na]$^+$** calculated for $C_{25}H_{21}{}^{79}BrN_2O_3Na^+$: 499.0628, found: 499.0622; calculated for $C_{25}H_{21}{}^{81}BrN_2O_3Na^+$: 501.0607, found: 501.0607.

ethyl-3-(2,2-dicyano-1-(p-tolyl)vinyl)-6-phenyl-2,3,4,7-tetrahydrooxepine-3-carboxylate 4g

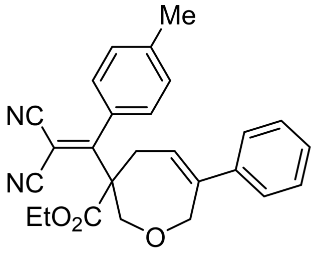

Prepared according to the general procedure to afford **4g** (30.1 mg, m. p. = 104 – 107 °C) in 73% yield as pale yellow solid. The regioisomeric ratio was determined to be >20:1 by crude ^{1}H NMR analysis.

NMR and HRMS data for the product **4g**:

^{1}H NMR (600 MHz, CDCl$_3$) δ (ppm): 7.35 – 7.26 (m, 5H), 7.12 (d, *J* = 8.4 Hz, 2H), 7.09 (d, *J* = 6.6 Hz, 2H), 5.55 (t, *J* = 7.2 Hz, 1H), 4.69 (d, *J* = 16.2 Hz, 1H), 4.64 (d, *J* = 16.2 Hz, 1H), 4.53 (d, *J* = 12.6 Hz, 1H), 4.37 – 4.24 (m, 2H), 4.16 (d, *J* = 12.6 Hz, 1H), 3.03 (dd, *J* = 14.4, 6.0 Hz, 1H), 2.93 (dd, *J* = 15.0, 6.6 Hz, 1H), 2.42 (s, 3H), 1.34 (t, *J* = 7.2 Hz, 3H).

^{13}C NMR (150 MHz, CDCl$_3$) δ (ppm): 181.0, 170.6, 141.3, 140.7, 139.1, 132.6, 129.5, 128.5, 127.7, 126.7, 125.6, 122.3, 112.0, 111.4, 89.8, 75.0, 74.2, 62.7, 61.0, 32.4, 21.4, 14.0.

HRMS (ESI-TOF) m/z: **[M + Na]$^+$** calculated for $C_{26}H_{24}N_2O_3Na^+$: 435.1679, found: 435.1677.

ethyl-3-(2,2-dicyano-1-(4-methoxyphenyl)vinyl)-6-phenyl-2,3,4,7-tetrahydrooxepine-3-carboxylate 4h

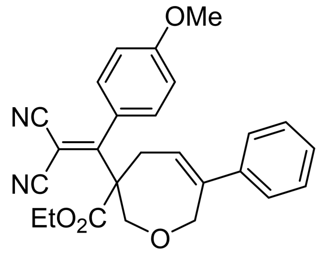

Prepared according to the general procedure to afford **4h** (38.9 mg) in 91% yield as yellow semisolid. The regioisomeric ratio was determined to be >20:1 by crude ^{1}H NMR analysis.

NMR and HRMS data for the product **4h**:

^{1}H NMR (600 MHz, CDCl$_3$) δ (ppm): 7.33 – 7.27 (m, 3H), 7.18 (d, *J* = 9.0 Hz, 2H), 7.11 (d, *J* = 8.4 Hz, 2H), 6.98 (d, *J* = 9.0 Hz, 2H), 5.56 (t, *J* = 7.2 Hz, 1H), 4.69 (d, *J* = 17.4 Hz, 1H), 4.65 (d, *J* = 15.6 Hz, 1H), 4.53 (d, *J* = 12.6 Hz, 1H), 4.37 – 4.24 (m, 2H), 4.18 (d, *J* = 12.0 Hz, 1H), 3.85 (s, 3H), 3.05 (dd, *J* = 14.4, 6.0 Hz, 1H), 2.93 (dd, *J* = 15.0, 6.6 Hz, 1H), 1.33 (t, *J* = 7.2 Hz, 3H).

^{13}C NMR (150 MHz, CDCl$_3$) δ (ppm): 180.8, 170.6, 161.1, 141.3, 139.1, 128.6, 128.5, 127.7, 127.6, 125.6, 122.4, 114.2, 112.2, 111.6, 89.7, 75.0, 74.3, 62.7, 61.2, 55.4, 32.4, 14.0.

HRMS (ESI-TOF) m/z: **[M + Na]$^+$** calculated for $C_{26}H_{24}N_2O_4Na^+$: 451.1628, found: 451.1624.

ethyl-3-(2,2-dicyano-1-(naphthalen-2-yl)vinyl)-6-phenyl-2,3,4,7-tetrahydrooxepine-3-carboxylate 4i

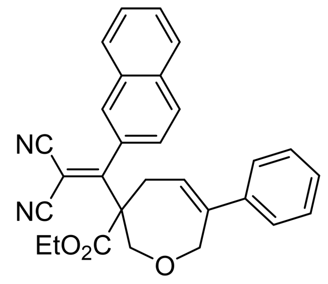

The reaction was performed according to the general procedure at 80 °C to afford **4i** (31.0 mg) in 69% yield as yellow semisolid. The regioisomeric ratio was determined to be >20:1 by crude ^{1}H NMR analysis.

NMR and HRMS data for the product **4i**:

^{1}H NMR (600 MHz, CDCl$_3$) δ (ppm): 7.96 (d, *J* = 9.0 Hz, 1H), 7.91 (d, *J* = 7.8 Hz, 1H), 7.86 (d, *J* = 8.4 Hz, 1H), 7.72 (s, 1H), 7.65 – 7.55 (m, 2H), 7.34 (d, *J* = 7.8 Hz, 1H), 7.24 (brs, 3H), 7.00 (brs, 2H), 5.46 (brs, 1H), 4.70 (d, *J* = 15.6 Hz, 1H), 4.64 (d, *J* = 16.8 Hz, 1H), 4.61 (d, *J* = 13.2 Hz, 1H), 4.42 – 4.30 (m, 2H), 4.26 (d, *J* = 12.6 Hz, 1H), 3.07 (brs, 1H), 2.99 (dd, *J* = 15.0, 7.2 Hz, 1H), 1.38 (t, *J* = 7.2 Hz, 3H).

^{13}C NMR (150 MHz, CDCl$_3$) δ (ppm): 180.8, 170.6, 141.4, 139.0, 133.6, 132.9, 132.4, 128.8, 128.5, 128.4, 128.0, 127.8, 127.7, 127.3, 126.9, 125.5, 123.4, 122.2, 111.9, 111.4, 90.3, 75.0, 74.4, 62.8, 61.1, 32.5, 14.1.

HRMS (ESI-TOF) m/z: **[M + Na]$^+$** calculated for $C_{29}H_{24}N_2O_3Na^+$: 471.1679, found: 471.1679.

ethyl-3-(2,2-dicyano-1-(thiophen-2-yl)vinyl)-6-phenyl-2,3,4,7-tetrahydrooxepine-3-carboxylate 4j

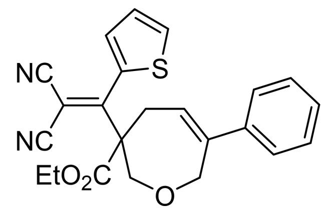

Prepared according to the general procedure to afford **4j** (35.1 mg) in 87% yield as dark yellow semisolid. The regioisomeric ratio was determined to be >20:1 by crude ^{1}H NMR analysis.

NMR and HRMS data for the product **4j**:

^{1}H NMR (600 MHz, CDCl$_3$) δ (ppm): 7.62 (d, *J* = 3.6 Hz, 1H), 7.36 – 7.26 (m, 3H), 7.19 (dd, *J* = 3.6, 1.2 Hz, 1H), 7.17 – 7.12 (m, 3H), 5.68 (t, *J* = 6.6 Hz, 1H), 4.69 (s, 2H), 4.48 (d, *J* =

12.0 Hz, 1H), 4.37 – 4.26 (m, 2H), 4.24 (d, J = 13.8 Hz, 1H), 3.16 (dd, J = 15.6, 7.2 Hz, 1H), 3.00 (dd, J = 14.4, 6.0 Hz, 1H), 1.34 (t, J = 7.2 Hz, 3H).

^{13}C NMR (150 MHz, CDCl$_3$) δ (ppm): 172.8, 170.3, 141.7, 139.2, 134.2, 130.4, 130.1, 128.5, 127.8, 127.7, 125.7, 122.2, 112.1, 111.3, 90.9, 75.0, 74.2, 62.8, 61.5, 32.5, 14.0.

HRMS (ESI-TOF) m/z: **[M + Na]$^+$** calculated for $C_{23}H_{20}N_2O_3SNa^+$: 427.1087, found: 427.1084.

methyl-3-(2,2-dicyano-1-phenylvinyl)-6-phenyl-2,3,4,7-tetrahydrooxepine-3-carboxylate 4k

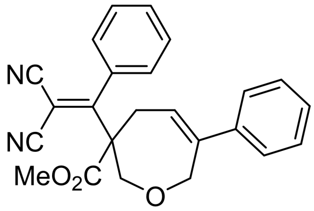

Prepared according to the general procedure to afford **4k** (30.0 mg, m. p. = 83 – 87 °C) in 78% yield as yellow solid. The regioisomeric ratio was determined to be >20:1 by crude ^{1}H NMR analysis.

NMR and HRMS data for the product **4k**:

^{1}H NMR (600 MHz, CDCl$_3$) δ (ppm): 7.54 – 7.45 (m, 3H), 7.33 – 7.20 (m, 5H), 7.07 (d, J = 8.4 Hz, 2H), 5.47 (t, J = 6.6 Hz, 1H), 4.70 (d, J = 18.0 Hz, 1H), 4.64 (d, J = 15.0 Hz, 1H), 4.56 (d, J = 12.0 Hz, 1H), 4.16 (d, J = 12.6 Hz, 1H), 3.86 (s, 3H), 2.99 (dd, J = 15.0, 6.6 Hz, 1H), 2.94 (q, J = 15.6, 7.2 Hz, 1H).

^{13}C NMR (150 MHz, CDCl$_3$) δ (ppm): 180.4, 171.2, 141.4, 139.0, 135.4, 130.4, 128.8, 128.5, 127.8, 126.7, 125.6, 122.0, 111.7, 111.2, 90.3, 75.1, 74.1, 61.0, 53.5, 32.4.

HRMS (ESI-TOF) m/z: **[M + Na]$^+$** calculated for $C_{24}H_{20}N_2O_3Na^+$: 407.1366, found: 407.1363.

***tert*-butyl-3-(2,2-dicyano-1-phenylvinyl)-6-phenyl-2,3,4,7-tetrahydrooxepine-3-carboxylate 4l**

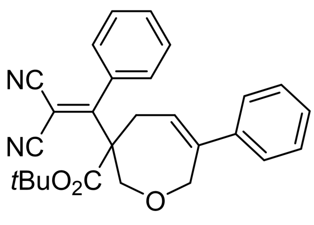

Prepared according to the general procedure to afford **4l** (40.1 mg, m. p. = 87 – 90 °C) in 94% yield as pale yellow solid. The regioisomeric ratio was determined to be >20:1 by crude ^{1}H NMR analysis.

NMR and HRMS data for the product **4l**:

^{1}H NMR (600 MHz, CDCl$_3$) δ (ppm): 7.54 – 7.43 (m, 3H), 7.33 – 7.26 (m, 3H), 7.22 (d, *J* = 6.6 Hz, 2H), 7.10 (d, *J* = 7.2 Hz, 2H), 5.51 (t, *J* = 6.6 Hz, 1H), 4.68 (d, *J* = 16.2 Hz, 1H), 4.64 (d, *J* = 15.6 Hz, 1H), 4.52 (d, *J* = 12.0 Hz, 1H), 4.19 (d, *J* = 12.0 Hz, 1H), 3.01 (dd, *J* = 15.6, 7.2 Hz, 1H), 2.90 (q, *J* = 15.0, 7.8 Hz, 1H), 1.53 (s, 9H).

^{13}C NMR (150 MHz, CDCl$_3$) δ (ppm): 181.6, 169.1, 141.4, 139.2, 135.9, 130.3, 128.7, 128.5, 127.7, 126.7, 125.5, 122.5, 111.9, 111.6, 89.8, 84.0, 74.9, 74.8, 61.4, 32.7, 27.8.

HRMS (ESI-TOF) m/z: **[M + Na]$^+$** calculated for $C_{27}H_{26}N_2O_3Na^+$: 449.1836, found: 449.1832.

ethyl-3-(2,2-dicyano-1-phenylvinyl)-6-(2-fluorophenyl)-2,3,4,7-tetrahydrooxepine-3-carboxylate 4m

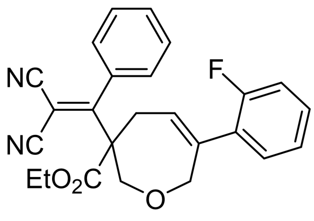

The reaction was performed according to the general procedure at 100 °C to afford **4m** (23.3 mg) in 56% yield as pale yellow semisolid. The regioisomeric ratio was determined to be >20:1 by crude ^{1}H NMR analysis.

NMR and HRMS data for the product **4m**:

^{1}H NMR (600 MHz, CDCl$_3$) δ (ppm): 7.52 – 7.43 (m, 3H), 7.31 – 7.21 (m, 3H), 7.06 (t, *J* = 7.2 Hz, 1H), 7.04 – 6.97 (m, 2H), 5.41 (t, *J* = 6.0 Hz, 1H), 4.56 (brs, 2H), 4.52 (d, *J* = 13.2 Hz, 1H), 4.38 – 4.29 (m, 2H), 4.27 (d, *J* = 12.6 Hz, 1H), 3.02 (dd, *J* = 14.4, 6.0 Hz, 1H), 2.92 (dd, *J* = 15.6, 7.2 Hz, 1H), 1.36 (t, *J* = 7.2 Hz, 3H).

^{13}C NMR (150 MHz, CDCl$_3$) δ (ppm): 180.6, 170.4, 159.5 (d, $J_{C\text{-}F}$ = 245.6 Hz), 138.4, 135.5, 130.4, 129.6 (d, $J_{C\text{-}F}$ = 4.4 Hz), 129.4 (d, $J_{C\text{-}F}$ = 8.7 Hz), 128.8, 127.3 (d, $J_{C\text{-}F}$ = 14.4 Hz), 126.8, 125.6, 124.2 (d, $J_{C\text{-}F}$ = 2.9 Hz), 115.7 (d, $J_{C\text{-}F}$ = 21.6 Hz), 111.8, 111.3, 90.3, 74.9 (d, $J_{C\text{-}F}$ = 5.7 Hz), 74.6, 62.8, 60.6, 32.9, 14.0.

^{19}F NMR (564 MHz, CDCl$_3$) δ (ppm): -114.5.

HRMS (ESI-TOF) m/z: **[M + Na]$^+$** calculated for $C_{25}H_{21}FN_2O_3Na^+$: 439.1428, found: 439.1433.

ethyl-6-(3-chlorophenyl)-3-(2,2-dicyano-1-phenylvinyl)-2,3,4,7-tetrahydrooxepine-3-carboxylate 4n

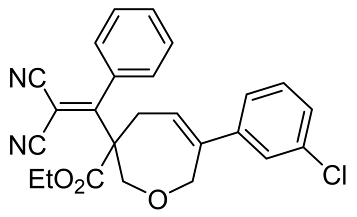

Prepared according to the general procedure to afford **4n** (35.1 mg, m. p. = 96 – 101 °C) in 81% yield as yellow solid. The regioisomeric ratio was determined to be >20:1 by crude ^{1}H NMR analysis.

NMR and HRMS data for the product **4n**:

^{1}H NMR (600 MHz, CDCl$_3$) δ (ppm): 7.56 – 7.47 (m, 3H), 7.25 – 7.17 (m, 4H), 7.03 (s, 1H), 6.95 (d, J = 7.2 Hz, 1H), 5.47 (t, J = 7.2 Hz, 1H), 4.66 (d, J = 16.2 Hz, 1H), 4.62 – 4.52 (m, 2H), 4.37 – 4.26 (m, 2H), 4.17 (d, J = 12.6 Hz, 1H), 2.99 (dd, J = 15.0, 6.6 Hz, 1H), 2.93 (dd, J = 14.4, 6.0 Hz, 1H), 1.35 (t, J = 7.2 Hz, 3H).

^{13}C NMR (150 MHz, CDCl$_3$) δ (ppm): 180.4, 170.4, 140.9, 140.2, 135.5, 134.4, 130.5, 129.7, 128.9, 127.8, 126.7, 125.9, 123.7, 123.5, 111.7, 111.3, 90.2, 74.8, 74.3, 62.8, 60.9, 32.4, 14.0.

HRMS (ESI-TOF) m/z: **[M + Na]$^+$** calculated for $C_{25}H_{21}{}^{35}ClN_2O_3Na^+$: 455.1133, found: 455.1135; calculated for $C_{25}H_{21}{}^{37}ClN_2O_3Na^+$: 457.1103, found: 457.1112.

ethyl-6-(3-bromophenyl)-3-(2,2-dicyano-1-phenylvinyl)-2,3,4,7-tetrahydrooxepine-3-carboxylate 4o

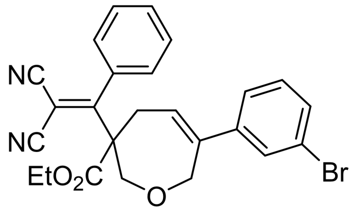

Prepared according to the general procedure to afford **4o** (41.0 mg, m. p. = 130 – 132 °C) in 86% yield as pale yellow solid. The regioisomeric ratio was determined to be >20:1 by crude ^{1}H NMR analysis.

NMR and HRMS data for the product **4o**:

^{1}H NMR (600 MHz, CDCl$_3$) δ (ppm): 7.57 – 7.47 (m, 3H), 7.38 (d, *J* = 7.2 Hz, 1H), 7.23 (d, *J* = 6.6 Hz, 2H), 7.18 (s, 1H), 7.15 (t, *J* = 8.4 Hz, 1H), 6.99 (d, *J* = 7.8 Hz, 1H), 5.45 (t, *J* = 6.6 Hz, 1H), 4.65 (d, *J* = 16.2 Hz, 1H),4.57 (d, *J* = 17.4 Hz, 1H), 4.56 (d, *J* = 12.0 Hz, 1H), 4.37 – 4.26 (m, 2H), 4.17 (d, *J* = 12.0 Hz, 1H), 2.99 (dd, *J* = 15.6, 6.6 Hz, 1H), 2.93 (dd, *J* = 14.4, 6.6 Hz, 1H), 1.35 (t, *J* = 7.2 Hz, 3H).

^{13}C NMR (150 MHz, CDCl$_3$) δ (ppm): 180.4, 170.4, 141.1, 140.1, 135.5, 130.7, 130.5, 130.0, 128.9, 128.8, 126.7, 124.1, 123.6, 122.6, 111.7, 111.3, 90.2, 74.7, 74.3, 62.8, 60.9, 32.4, 14.0.

HRMS (ESI-TOF) m/z: **[M + Na]$^+$** calculated for $C_{25}H_{21}{}^{79}BrN_2O_3Na^+$: 499.0628, found: 499.0622; calculated for $C_{25}H_{21}{}^{81}BrN_2O_3Na^+$: 501.0607, found: 501.0607.

ethyl-3-(2,2-dicyano-1-phenylvinyl)-6-(3-nitrophenyl)-2,3,4,7-tetrahydrooxepine-3-carboxylate 4p

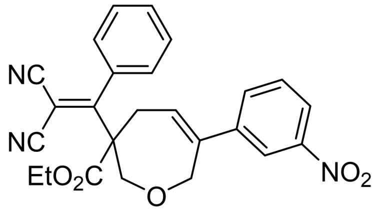

The reaction was performed according to the general procedure at 80 °C to afford **4p** (40.3 mg) in 91% yield as yellow semisolid. The regioisomeric ratio was determined to be >20:1 by crude ^{1}H NMR analysis.

NMR and HRMS data for the product **4p**:

^{1}H NMR (600 MHz, CDCl$_3$) δ (ppm): 8.11 (d, *J* = 8.4 Hz, 1H), 7.89 (s, 1H), 7.60 – 7.50 (m, 3H), 7.47 (t, *J* = 8.4 Hz, 1H), 7.39 (d, *J* = 8.4 Hz, 1H), 7.25 (d, *J* = 7.2 Hz, 2H), 5.55 (t, *J* = 6.6 Hz, 1H), 4.73 (d, *J* = 16.2 Hz, 1H), 4.63 (d, *J* = 16.2 Hz, 1H), 4.60 (d, *J* = 12.6 Hz, 1H), 4.37 –

4.28 (m, 2H), 4.21 (d, *J* = 12.6 Hz, 1H), 3.02 (dd, *J* = 14.4, 7.2 Hz, 1H), 2.97 (dd, *J* = 14.4, 6.0 Hz, 1H), 1.36 (t, *J* = 7.2 Hz, 3H).

^{13}C NMR (150 MHz, CDCl$_3$) δ (ppm): 180.0, 170.3, 148.3, 140.7, 139.4, 135.4, 131.4, 130.7, 129.5, 129.0, 126.7, 125.2, 122.5, 120.6, 111.6, 111.2, 90.3, 74.6, 74.5, 62.9, 60.9, 32.5, 14.0.

HRMS (ESI-TOF) m/z: **[M + Na]$^+$** calculated for $C_{25}H_{21}N_3O_5Na^+$: 466.1373, found: 466.1363.

ethyl-3-(2,2-dicyano-1-phenylvinyl)-6-(m-tolyl)-2,3,4,7-tetrahydrooxepine-3-carboxylate 4q

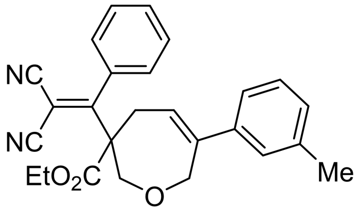

Prepared according to the general procedure to afford **4q** (36.7 mg, m. p. = 126 – 129 °C) in 89% yield as pale yellow solid. The regioisomeric ratio was determined to be >20:1 by crude ^{1}H NMR analysis.

NMR and HRMS data for the product **4q**:

^{1}H NMR (600 MHz, CDCl$_3$) δ (ppm): 7.56 – 7.46 (m, 3H), 7.24 (d, *J* = 6.6 Hz, 2H), 7.18 (t, *J* = 7.8 Hz, 1H), 7.08 (d, *J* = 7.2 Hz, 1H), 6.88 (d, *J* = 6.0 Hz, 2H), 5.48 (t, *J* = 6.0 Hz, 1H), 4.69 (d, *J* = 16.8 Hz, 1H), 4.62 (d, *J* = 15.0 Hz, 1H), 4.56 (d, *J* = 12.6 Hz, 1H), 4.38 – 4.26 (m, 2H), 4.17 (d, *J* = 12.0 Hz, 1H), 3.00 (dd, *J* = 14.4, 7.2 Hz, 1H), 2.95 (dd, *J* = 14.4, 6.6 Hz, 1H), 2.33 (s, 3H), 1.36 (t, *J* = 7.2 Hz, 3H).

^{13}C NMR (150 MHz, CDCl$_3$) δ (ppm): 180.7, 170.5, 141.5, 139.1, 138.0, 135.6, 130.3, 128.8, 128.4, 128.3, 126.7, 126.4, 122.6, 121.9, 111.8, 111.3, 90.1, 75.0, 74.2, 62.7, 60.9, 32.4, 21.4, 14.0.

HRMS (ESI-TOF) m/z: **[M + Na]$^+$** calculated for $C_{26}H_{24}N_2O_3Na^+$: 435.1679, found: 435.1677.

ethyl-3-(2,2-dicyano-1-phenylvinyl)-6-(3-methoxyphenyl)-2,3,4,7-tetrahydrooxepine-3-carboxylate 4r

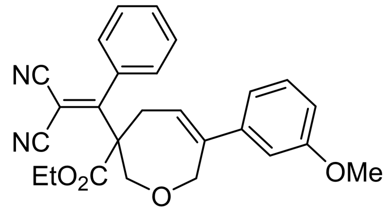

Prepared according to the general procedure to afford **4r** (34.7 mg, m. p. = 149 – 152 °C) in 81% yield as yellow solid. The regioisomeric ratio was determined to be >20:1 by crude ^{1}H NMR analysis.

NMR and HRMS data for the product **4r**:

^{1}H NMR (600 MHz, CDCl$_3$) δ (ppm): 7.54 – 7.45 (m, 3H), 7.25 – 7.17 (m, 3H), 6.81 (d, *J* = 8.4 Hz, 1H), 6.67 (d, *J* = 7.8 Hz, 1H), 6.60 (s, 1H), 5.50 (t, *J* = 6.0 Hz, 1H), 4.67 (d, *J* = 16.8 Hz, 1H), 4.62 (d, *J* = 15.6 Hz, 1H), 4.55 (d, *J* = 13.2 Hz, 1H), 4.38 – 4.26 (m, 2H), 4.18 (d, *J* = 12.6 Hz, 1H), 3.79 (s, 3H), 3.00 (dd, *J* = 14.4, 6.6 Hz, 1H), 2.93 (dd, *J* = 15.0, 6.6 Hz, 1H), 1.35 (t, *J* = 6.6 Hz, 3H).

^{13}C NMR (150 MHz, CDCl$_3$) δ (ppm): 180.7, 170.5, 159.6, 141.3, 140.6, 135.5, 130.4, 129.5, 128.8, 126.7, 122.3, 118.0, 112.8, 111.8, 111.7, 111.3, 90.2, 75.0, 74.3, 62.7, 60.9, 55.2, 32.5, 14.1.

HRMS (ESI-TOF) m/z: **[M + Na]$^+$** calculated for $C_{26}H_{24}N_2O_4Na^+$: 451.1628, found: 451.1628.

ethyl-3-(2,2-dicyano-1-phenylvinyl)-6-(4-fluorophenyl)-2,3,4,7-tetrahydrooxepine-3-carboxylate 4s

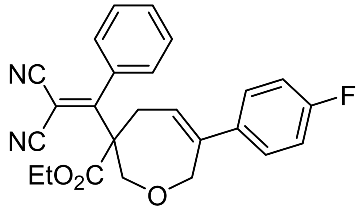

The reaction was performed according to the general procedure at 80 °C to afford **4s** (29.5 mg, m. p. = 107 – 112 °C) in 71% yield as pale yellow solid. The regioisomeric ratio was determined to be >20:1 by crude ^{1}H NMR analysis.

NMR and HRMS data for the product **4s**:

^{1}H NMR (600 MHz, CDCl$_3$) δ (ppm): 7.53 – 7.41 (m, 3H), 7.21 (d, *J* = 6.6 Hz, 2H), 7.07 – 7.01 (m, 2H), 6.96 (t, *J* = 9.0 Hz, 2H), 5.44 (t, *J* = 6.6 Hz, 1H), 4.63 (d, *J* = 16.8 Hz, 1H), 4.59

(d, J = 17.4 Hz, 1H), 4.53 (d, J = 12.6 Hz, 1H), 4.35 – 4.25 (m, 2H), 4.17 (d, J = 13.2 Hz, 1H), 2.99 (dd, J = 14.4, 6.6 Hz, 1H), 2.89 (dd, J = 15.0, 6.6 Hz, 1H), 1.33 (t, J = 7.2 Hz, 3H).

^{13}C NMR (150 MHz, $CDCl_3$) δ (ppm): 180.6, 170.4, 162.3 (d, J_{C-F} = 245.6 Hz), 140.6, 135.5, 135.2 (d, J_{C-F} = 2.9 Hz), 130.4, 128.8, 127.3 (d, J_{C-F} = 7.2 Hz), 126.7, 122.3, 115.4 (d, J_{C-F} = 21.5 Hz), 111.7, 111.3, 90.2, 75.0, 74.4, 62.8, 60.9, 32.4, 14.1.

^{19}F NMR (564 MHz, $CDCl_3$) δ (ppm): -114.1.

HRMS (ESI-TOF) m/z: **[M + Na]$^+$** calculated for $C_{25}H_{21}FN_2O_3Na^+$: 439.1428, found: 439.1434.

ethyl-6-(4-chlorophenyl)-3-(2,2-dicyano-1-phenylvinyl)-2,3,4,7-tetrahydrooxepine-3-carboxylate 4t

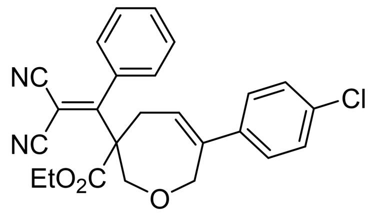

Prepared according to the general procedure to afford **4t** (37.7 mg, m. p. = 103 – 107 °C) in 87% yield as yellow solid. The regioisomeric ratio was determined to be >20:1 by crude ^{1}H NMR analysis.

NMR and HRMS data for the product **4t**:

^{1}H NMR (600 MHz, $CDCl_3$) δ (ppm): 7.53 – 7.44 (m, 3H), 7.25 (d, J = 7.8 Hz, 2H), 7.22 (d, J = 6.6 Hz, 2H), 7.01 (d, J = 8.4 Hz, 2H), 5.49 (t, J = 6.6 Hz, 1H), 4.64 (d, J = 15.6 Hz, 1H), 4.60 (d, J = 16.2 Hz, 1H), 4.54 (d, J = 12.6 Hz, 1H), 4.35 – 4.25 (m, 2H), 4.19 (d, J = 13.2 Hz, 1H), 3.00 (dd, J = 14.4, 6.6 Hz, 1H), 2.91 (dd, J = 14.4, 6.0 Hz, 1H), 1.34 (t, J = 7.2 Hz, 3H).

^{13}C NMR (150 MHz, $CDCl_3$) δ (ppm): 180.5, 170.4, 140.4, 137.5, 135.5, 133.6, 130.4, 128.8, 128.6, 126.9, 126.7, 122.9, 111.7, 111.3, 90.2, 74.8, 74.4, 62.8, 60.8, 32.5, 14.0.

HRMS (ESI-TOF) m/z: **[M + Na]$^+$** calculated for $C_{25}H_{21}{}^{35}ClN_2O_3Na^+$: 455.1133, found: 455.1124; calculated for $C_{25}H_{21}{}^{37}ClN_2O_3Na^+$: 457.1103, found: 457.1106.

ethyl-6-(4-bromophenyl)-3-(2,2-dicyano-1-phenylvinyl)-2,3,4,7-tetrahydrooxepine-3-carboxylate 4u

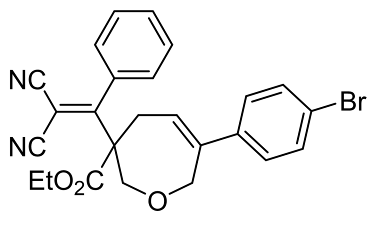

Prepared according to the general procedure to afford **4u** (42.0 mg, m. p. = 128 – 132 °C) in 88% yield as pale yellow solid. The regioisomeric ratio was determined to be >20:1 by crude ^{1}H NMR analysis.

NMR and HRMS data for the product **4u**:

^{1}H NMR (600 MHz, CDCl$_3$) δ (ppm): 7.55 – 7.44 (m, 3H), 7.41 (d, *J* = 8.4 Hz, 2H), 7.22 (d, *J* = 6.6 Hz, 2H), 6.95 (d, *J* = 8.4 Hz, 2H), 5.49 (t, *J* = 6.0 Hz, 1H), 4.64 (d, *J* = 15.6 Hz, 1H), 4.59 (d, *J* = 17.4 Hz, 1H), 4.54 (d, *J* = 13.2 Hz, 1H), 4.36 – 4.25 (m, 2H), 4.19 (d, *J* = 13.2 Hz, 1H), 3.00 (dd, *J* = 15.0, 6.6 Hz, 1H), 2.91 (dd, *J* = 14.4, 6.0 Hz, 1H), 1.34 (t, *J* = 7.2 Hz, 3H).

^{13}C NMR (150 MHz, CDCl$_3$) δ (ppm): 180.5, 170.4, 140.5, 137.9, 135.5, 131.6, 130.4, 128.8, 127.2, 126.7, 122.9, 121.7, 111.7, 111.3, 90.2, 74.7, 74.4, 62.8, 60.8, 32.5, 14.0.

HRMS (ESI-TOF) m/z: **[M + Na]$^+$** calculated for $C_{25}H_{21}{}^{79}BrN_2O_3Na^+$: 499.0628, found: 499.0619; calculated for $C_{25}H_{21}{}^{81}BrN_2O_3Na^+$: 501.0607, found: 501.0603.

ethyl-3-(2,2-dicyano-1-phenylvinyl)-6-(p-tolyl)-2,3,4,7-tetrahydrooxepine-3-carboxylate 4v

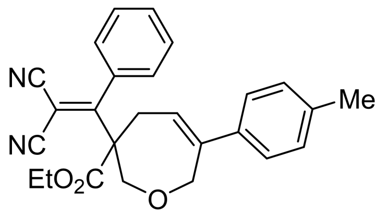

Prepared according to the general procedure to afford **4v** (34.2 mg, m. p. = 78 – 83 °C) in 83% yield as yellow solid. The regioisomeric ratio was determined to be >20:1 by crude ^{1}H NMR analysis.

NMR and HRMS data for the product **4v**:

^{1}H NMR (600 MHz, CDCl$_3$) δ (ppm): 7.55 – 7.44 (m, 3H), 7.23 (d, *J* = 6.6 Hz, 2H), 7.10 (d, *J* = 8.4 Hz, 2H), 6.98 (d, *J* = 8.4 Hz, 2H), 5.47 (t, *J* = 6.6 Hz, 1H), 4.67 (d, *J* = 15.6 Hz, 1H), 4.62 (d, *J* = 16.2 Hz, 1H), 4.55 (d, *J* = 12.6 Hz, 1H), 4.37 – 4.25 (m, 2H), 4.16 (d, *J* = 12.6 Hz, 1H),

2.99 (dd, J = 15.6, 7.2 Hz, 1H), 2.93 (dd, J = 14.4, 6.0 Hz, 1H), 2.33 (s, 3H), 1.35 (t, J = 7.2 Hz, 3H).

^{13}C NMR (150 MHz, CDCl$_3$) δ (ppm): 180.8, 170.5, 141.2, 137.6, 136.2, 135.6, 130.4, 129.1, 128.8, 126.7, 125.4, 121.4, 111.8, 111.3, 90.1, 75.0, 74.3, 62.7, 61.0, 32.4, 21.1, 14.1.

HRMS (ESI-TOF) m/z: **[M + Na]$^+$** calculated for $C_{26}H_{24}N_2O_3Na^+$: 435.1679, found: 435.1682.

ethyl-3-(2,2-dicyano-1-phenylvinyl)-6-(4-ethylphenyl)-2,3,4,7-tetrahydrooxepine-3-carboxylate 4w

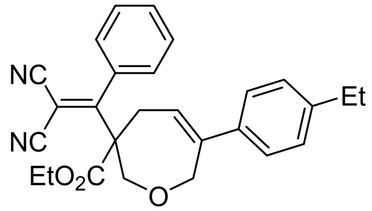

Prepared according to the general procedure to afford **4w** (31.6 mg) in 74% yield as yellow semisolid. The regioisomeric ratio was determined to be >20:1 by crude ^{1}H NMR analysis.

NMR and HRMS data for the product **4w**:

^{1}H NMR (600 MHz, CDCl$_3$) δ (ppm): 7.55 – 7.44 (m, 3H), 7.23 (d, J = 6.0 Hz, 2H), 7.13 (d, J = 8.4 Hz, 2H), 7.01 (d, J = 8.4 Hz, 2H), 5.48 (t, J = 6.6 Hz, 1H), 4.68 (d, J = 17.4 Hz, 1H), 4.63 (d, J = 15.0 Hz, 1H), 4.55 (d, J = 12.6 Hz, 1H), 4.37 – 4.26 (m, 2H), 4.16 (d, J = 12.0 Hz, 1H), 3.00 (dd, J = 15.0, 6.6 Hz, 1H), 2.94 (dd, J = 14.4, 6.0 Hz, 1H), 2.62 (q, J = 7.2 Hz, 2H), 1.35 (t, J = 7.2 Hz, 3H), 1.22 (t, J = 7.2 Hz, 3H).

^{13}C NMR (150 MHz, CDCl$_3$) δ (ppm): 180.8, 170.5, 143.9, 141.2, 136.4, 135.6, 130.4, 128.8, 127.9, 126.7, 125.5, 121.4, 111.8, 111.3, 90.1, 75.0, 74.2, 62.7, 61.0, 32.4, 28.4, 15.5, 14.0.

HRMS (ESI-TOF) m/z: **[M + Na]$^+$** calculated for $C_{27}H_{26}N_2O_3Na^+$: 449.1836, found: 449.1839.

ethyl-3-(2,2-dicyano-1-phenylvinyl)-6-(4-methoxyphenyl)-2,3,4,7-tetrahydrooxepine-3-carboxylate 4x

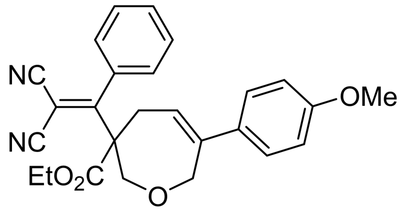

Prepared according to the general procedure to afford **4x** (32.5 mg) in 76% yield as yellow semisolid. The regioisomeric ratio was determined to be >20:1 by crude ^{1}H NMR analysis.

NMR and HRMS data for the product **4x**:

^{1}H NMR (600 MHz, CDCl$_3$) δ (ppm): 7.55 – 7.44 (m, 3H), 7.22 (d, *J* = 6.6 Hz, 2H), 7.02 (d, *J* = 9.0 Hz, 2H), 6.82 (d, *J* = 8.4 Hz, 2H), 5.42 (t, *J* = 6.6 Hz, 1H), 4.66 (d, *J* = 16.2 Hz, 1H), 4.61 (d, *J* = 16.2 Hz, 1H), 4.55 (d, *J* = 12.6 Hz, 1H), 4.37 – 4.25 (m, 2H), 4.15 (d, *J* = 13.2 Hz, 1H), 3.79 (s, 3H), 2.99 (dd, *J* = 15.0, 7.2 Hz, 1H), 2.92 (dd, *J* = 14.4, 6.6 Hz, 1H), 1.35 (t, *J* = 7.2 Hz, 3H).

^{13}C NMR (150 MHz, CDCl$_3$) δ (ppm): 180.8, 170.5, 159.2, 140.8, 135.6, 131.5, 130.3, 128.8, 126.7, 120.6, 113.8, 111.8, 111.3, 90.1, 75.0, 74.2, 62.7, 61.0, 55.3, 32.4, 14.1.

HRMS (ESI-TOF) m/z: **[M + Na]$^+$** calculated for $C_{26}H_{24}N_2O_4Na^+$: 451.1628, found: 451.1628.

ethyl-6-(3,4-dichlorophenyl)-3-(2,2-dicyano-1-phenylvinyl)-2,3,4,7-tetrahydrooxepine-3-carboxylate 4y

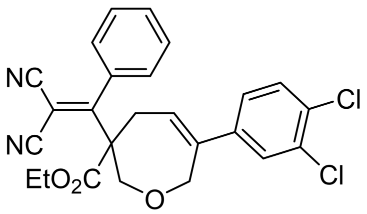

The reaction was performed according to the general procedure at 80 °C to afford **4y** (36.9 mg, m. p. = 125 – 129 °C) in 79% yield as pale yellow solid. The regioisomeric ratio was determined to be >20:1 by crude ^{1}H NMR analysis.

NMR and HRMS data for the product **4y**:

^{1}H NMR (600 MHz, CDCl$_3$) δ (ppm): 7.56 – 7.47 (m, 3H), 7.35 (d, *J* = 7.8 Hz, 1H), 7.22 (d, *J* = 7.2 Hz, 2H), 7.13 (s, 1H), 6.90 (d, *J* = 8.4 Hz, 1H), 5.48 (t, *J* = 6.6 Hz, 1H), 4.63 (d, *J* = 16.2 Hz, 1H), 4.56 (d, *J* = 15.6 Hz, 1H), 4.55 (d, *J* = 12.0 Hz, 1H), 4.37 – 4.25 (m, 2H), 4.18 (d, *J* =

13.2 Hz, 1H), 2.99 (dd, J = 14.4, 6.0 Hz, 1H), 2.92 (dd, J = 15.6, 7.2 Hz, 1H), 1.34 (t, J = 7.2 Hz, 3H).

^{13}C NMR (150 MHz, CDCl$_3$) δ (ppm): 180.2, 170.3, 139.4, 139.0, 135.4, 132.6, 131.8, 130.5, 130.4, 128.9, 127.6, 126.7, 124.8, 124.1, 111.7, 111.2, 90.3, 74.6, 74.4, 62.9, 60.9, 32.5, 14.0.

HRMS (ESI-TOF) m/z: **[M + Na]$^+$** calculated for $C_{25}H_{20}{}^{35}Cl_2N_2O_3Na^+$: 489.0743, found: 489.0734; calculated for $C_{25}H_{20}{}^{35}Cl^{37}ClN_2O_3Na^+$: 491.0714, found: 491.0714; calculated for $C_{25}H_{20}{}^{37}Cl_2N_2O_3Na^+$: 493.0684, found: 493.0681.

ethyl-3-(2,2-dicyano-1-phenylvinyl)-6-(naphthalen-2-yl)-2,3,4,7-tetrahydrooxepine-3-carboxylate 4z

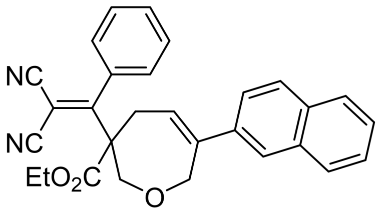

Prepared according to the general procedure to afford **4z** (36.8 mg, m. p. = 131 – 133 °C) in 82% yield as yellow solid. The regioisomeric ratio was determined to be >20:1 by crude ^{1}H NMR analysis.

NMR and HRMS data for the product **4z**:

^{1}H NMR (600 MHz, CDCl$_3$) δ (ppm): 7.83 – 7.74 (m, 3H), 7.57 – 7.43 (m, 6H), 7.30 – 7.19 (m, 3H), 5.63 (t, J = 6.6 Hz, 1H), 4.81 (d, J = 17.4 Hz, 1H), 4.76 (d, J = 15.0 Hz, 1H), 4.61 (d, J = 12.0 Hz, 1H), 4.39 – 4.28 (m, 2H), 4.22 (d, J = 13.2 Hz, 1H), 3.06 (dd, J = 14.4, 7.2 Hz, 1H), 3.01 (dd, J = 14.4, 6.0 Hz, 1H), 1.36 (t, J = 7.2 Hz, 3H).

^{13}C NMR (150 MHz, CDCl$_3$) δ (ppm): 180.7, 170.5, 141.2, 136.3, 135.6, 133.1, 132.7, 130.4, 128.8, 128.1, 128.0, 127.6, 126.8, 126.4, 126.2, 124.2, 123.8, 122.8, 111.8, 111.3, 90.2, 75.0, 74.4, 62.8, 61.0, 32.6, 14.1.

HRMS (ESI-TOF) m/z: **[M + Na]$^+$** calculated for $C_{29}H_{24}N_2O_3Na^+$: 471.1679, found: 471.1680.

ethyl-3-(2,2-dicyano-1-phenylvinyl)-6-(thiophen-2-yl)-2,3,4,7-tetrahydrooxepine-3-carboxylate 4aa

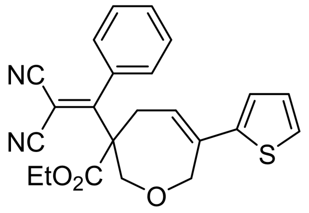

Prepared according to the general procedure to afford **4aa** (36.0 mg) in 89% yield as dark yellow semisolid. The regioisomeric ratio was determined to be >20:1 by crude ^{1}H NMR analysis.

NMR and HRMS data for the product **4aa**:

^{1}H NMR (600 MHz, CDCl$_3$) δ (ppm): 7.54 – 7.45 (m, 3H), 7.22 (brs, 2H), 7.14 (d, J = 5.4 Hz, 1H), 6.94 (dd, J = 5.4, 4.2 Hz, 1H), 6.77 (d, J = 3.6 Hz, 1H), 5.55 (t, J = 6.0 Hz, 1H), 4.70 (d, J = 18.0 Hz, 1H), 4.66 (d, J = 18.0 Hz, 1H), 4.61 (d, J = 12.0 Hz, 1H), 4.37 – 4.26 (m, 2H), 4.10 (d, J = 12.6 Hz, 1H), 2.94 (dd, J = 14.4, 7.2 Hz, 1H), 2.89 (dd, J = 15.6, 7.2 Hz, 1H), 1.35 (t, J = 7.2 Hz, 3H).

^{13}C NMR (150 MHz, CDCl$_3$) δ (ppm): 180.6, 170.5, 141.6, 135.4, 134.6, 130.4, 128.9, 127.5, 126.7, 124.3, 122.6, 120.7, 111.7, 111.3, 90.2, 74.5, 74.0, 62.8, 61.4, 32.1, 14.0.

HRMS (ESI-TOF) m/z: **[M + Na]$^+$** calculated for $C_{23}H_{20}N_2O_3SNa^+$: 427.1087, found: 427.1096.

6. Synthetic Transformation of 3a and 4a

*6.1 Procedure for the hydrolysis of cyano group on **3a***

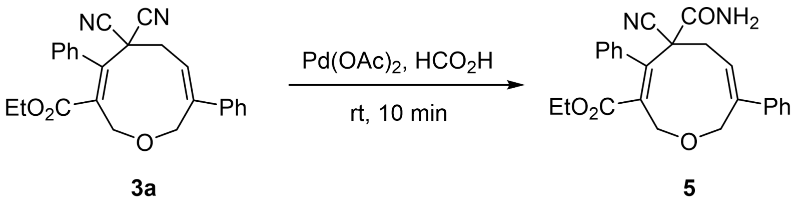

A mixture of nine-membered product **3a** (39.8 mg, 0.10 mmol) and $Pd(OAc)_2$ (1.1 mg, 0.005 mmol) in HCOOH (1.0 mL) was stirred at room temperature for 10 min and then diluted with water. The mixture was saturated with sodium carbonate until the pH = 7 – 8, followed by extraction with ethyl acetate (3 × 5 mL). The combined organic phase was washed with saturated brine (3 × 5 mL), dried over Na_2SO_4, filtered, and concentrated in vacuo. The crude product was purified by column chromatography on silica gel (petroleum ether/ ethyl acetate = 6/1 to 2/1) to afford **5** (33.7 mg) as colorless semisolid in 81% yields, which was dried under vacuum and further analyzed by ^{1}H NMR, ^{13}C NMR, HRMS, *etc.*

ethyl-(3*E*,7*Z*)-5-carbamoyl-5-cyano-4,8-diphenyl-2,5,6,9-tetrahydrooxonine-3-carboxylate 5

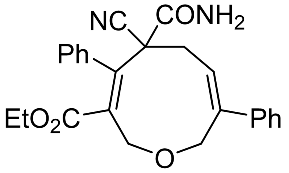

Purification of the crude product *via* column chromatography delivered **5** (33.7 mg) in 81% yield as colorless semisolid.

NMR and HRMS data for the product **5**:

^{1}H NMR (600 MHz, $CDCl_3$) δ (ppm): 7.52 (d, *J* = 7.8 Hz, 2H), 7.39 – 7.26 (m, 6H), 7.10 (brs, 2H), 6.21 (dd, *J* = 12.0, 7.2 Hz, 1H), 6.00 (d, *J* = 127.2 Hz, 2H), 4.77 (d, *J* = 13.8 Hz, 1H), 4.62 (d, *J* = 13.8 Hz, 1H), 4.61 (d, *J* = 13.8 Hz, 1H), 4.55 (d, *J* = 13.2 Hz, 1H), 4.00 (dd, *J* = 14.4, 12.0 Hz, 1H), 3.79 – 3.63 (m, 2H), 3.27 (dd, *J* = 14.4, 6.6 Hz, 1H), 0.69 (t, *J* = 7.2 Hz, 3H).

^{13}C NMR (150 MHz, $CDCl_3$) δ (ppm): 168.9, 166.6, 142.2, 141.5, 138.7, 137.7, 137.0, 129.4, 128.8, 128.3, 128.0, 127.6, 126.3, 126.1, 119.7, 68.5, 66.1, 61.0, 54.7, 36.6, 13.3.

HRMS (ESI-TOF) m/z: **$[M + Na]^+$** calculated for $C_{25}H_{24}N_2O_4Na^+$: 439.1628, found: 439.1634.

*6.2 Procedure for the reductive ring-opening reaction of **3a***

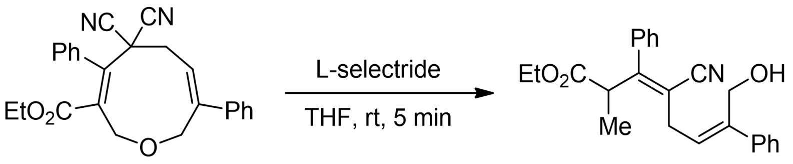

3a **6**

Nine-membered product **3a** (39.8 mg, 0.10 mmol) was dissolved in THF (1.0 mL) and stirred at room temperature. To this solution was slowly added L-Selectride (0.15 mL, 1.0 M in THF). The reaction mixture was stirred for 5 min at the same temperature, diluted with brine (5 mL) and extracted with ethyl acetate (3 × 5 mL). The combined organic phase was dried over Na_2SO_4, filtered and concentrated in vacuo. The crude product was purified by column chromatography on silica gel (petroleum ether/ ethyl acetate = 10/1 to 3/1) to afford **6** (17.6 mg) as pale yellow oil in 47% yields, which was dried under vacuum and further analyzed by ^{1}H NMR, ^{13}C NMR, HRMS, *etc*.

ethyl-(3*Z*,6*Z*)-4-cyano-8-hydroxy-2-methyl-3,7-diphenylocta-3,6-dienoate 6

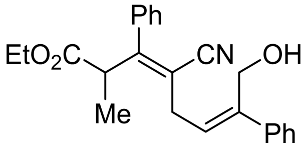

Purification of the crude product *via* column chromatography delivered **6** (17.6 mg) in 47% yield as pale yellow oil.

NMR and HRMS data for the product **6**:

^{1}H NMR (600 MHz, CDCl$_3$) δ (ppm): 7.49 (d, *J* = 6.6 Hz, 2H), 7.41 – 7.34 (m, 5H), 7.30 (t, *J* = 6.6 Hz, 1H), 7.23 – 7.19 (m, 2H), 5.92 (t, *J* = 7.2 Hz, 1H), 4.66 (s, 2H), 4.21 – 4.09 (m, 2H), 3.94 (q, *J* = 7.2 Hz, 1H), 3.46 (t, *J* = 7.8 Hz, 2H), 1.88 (brs, 1H), 1.26 (d, *J* = 6.6 Hz, 3H), 1.22 (t, *J* = 7.2 Hz, 3H).

^{13}C NMR (150 MHz, CDCl$_3$) δ (ppm): 171.7, 156.7, 142.5, 140.3, 137.6, 129.0, 128.6, 128.5, 128.0, 127.7, 126.5, 124.9, 118.8, 113.5, 61.6, 59.9, 43.0, 29.7, 15.3, 14.0.

HRMS (ESI-TOF) m/z: **[M + Na]$^+$** calculated for $C_{24}H_{25}NO_3Na^+$: 398.1727, found: 398.1731.

*6.3 Procedure for the retro-Knoevenagel reaction of seven-membered product **4a***

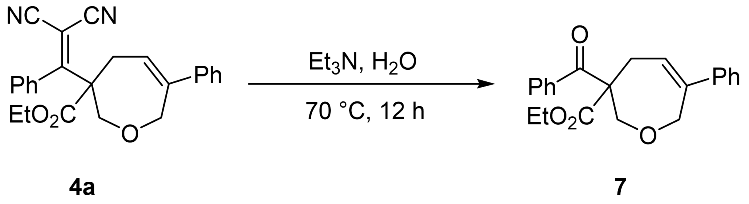

A glass tube was charged with seven-membered product **4a** (39.8 mg, 0.1 mmol), triethylamine (50 μL) in H_2O (1.0 mL). The mixture was stirred at 70 °C for 12 hour. Then the mixture was added with water (5 mL) and extracted with ethyl acetate (5 mL × 3). The organic layer was dried over Na_2SO_4, filtered and concentrated in vacuo. The crude product was purified by column chromatography on silica gel (petroleum ether/dichloromethane = 3/1 to 1/1) to afford **7** (18.2 mg) as pale yellow oil in 52% yields, which was dried under vacuum and further analyzed by ^{1}H NMR, ^{13}C NMR, HRMS, *etc*.

ethyl-3-benzoyl-6-phenyl-2,3,4,7-tetrahydrooxepine-3-carboxylate 7

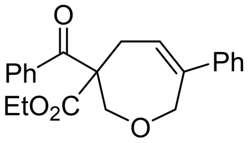

Purification of the crude product *via* column chromatography delivered **7** (18.2 mg) in 52% yield as pale yellow oil.

NMR and HRMS data for the product **7**:

^{1}H NMR (600 MHz, CDCl$_3$) δ (ppm): 7.80 (d, *J* = 7.8 Hz, 2H), 7.54 (t, *J* = 7.8 Hz, 1H), 7.43 (t, *J* = 8.4 Hz, 2H), 7.32 – 7.22 (m, 3H), 7.19 (d, *J* = 8.4 Hz, 2H), 5.88 (t, *J* = 6.6 Hz, 1H), 4.76 (d, *J* = 16.2 Hz, 1H), 4.69 (d, *J* = 18.0 Hz, 1H), 4.51 (d, *J* = 12.6 Hz, 1H), 4.34 (d, *J* = 12.0 Hz, 1H), 4.22 – 4.10 (m, 2H), 3.14 (d, *J* = 6.6 Hz, 2H), 1.10 (t, *J* = 7.2 Hz, 3H).

^{13}C NMR (150 MHz, CDCl$_3$) δ (ppm): 195.3, 172.1, 140.6, 139.5, 135.6, 133.0, 128.6, 128.5, 128.3, 127.4, 125.9, 122.9, 74.9, 72.3, 65.8, 61.8, 29.7, 13.9.

HRMS (ESI-TOF) m/z: **[M + Na]$^+$** calculated for $C_{22}H_{22}O_4Na^+$: 373.1410, found: 373.1411.

*6.4 Procedure for the removal of benzylidene malononitrile of **4a***

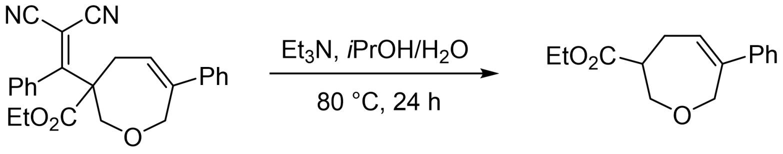

4a **8**

A glass tube was charged with seven-membered product **4a** (39.8 mg, 0.1 mmol), triethylamine (100 μL) in *i*-PrOH/H_2O (2 mL, 3:1 (*v*/*v*)). The mixture was stirred at 80 °C for 24 hour. Then the mixture was added with water (5 mL) and extracted with ethyl acetate (5 mL × 3). The organic layer was dried over Na_2SO_4, filtered and concentrated in vacuo. The crude product was purified by column chromatography on silica gel (petroleum ether/dichloromethane = 3/1 to 1/1) to afford **8** (22.9 mg) as pale yellow oil in 93% yields, which was dried under vacuum and further analyzed by ^{1}H NMR, ^{13}C NMR, HRMS, *etc*.

ethyl-6-phenyl-2,3,4,7-tetrahydrooxepine-3-carboxylate 8

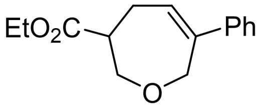

Prepared according to the general procedure to afford **8** (22.9 mg) in 93% yield as pale yellow oil.

NMR and HRMS data for the product **8**:

^{1}H NMR (600 MHz, $CDCl_3$) δ (ppm): 7.33 – 7.28 (m, 2H), 7.27 – 7.22 (m, 3H), 6.05 (t, *J* = 6.0 Hz, 1H), 4.61 (t, *J* = 14.4 Hz, 1H), 4.59 – 4.55 (m, 1H), 4.21 – 4.07 (m, 4H), 3.05 – 2.98 (m, 1H), 2.82 – 2.74 (m, 1H), 2.72 – 2.66 (m, 1H), 1.26 (t, *J* = 7.2 Hz, 3H).

^{13}C NMR (150 MHz, $CDCl_3$) δ (ppm): 173.1, 142.3, 140.8, 128.3, 127.2, 127.0, 125.9, 72.8, 72.5, 60.7, 45.3, 27.9, 14.2.

HRMS (ESI-TOF) m/z: **[M + Na]$^+$** calculated for $C_{15}H_{18}O_3Na^+$: 269.1148, found: 269.1147.

7. Procedure for the Synthesis of Medium-Sized Azacycles

*7.1 Procedure for the synthesis of nine-membered product **10***

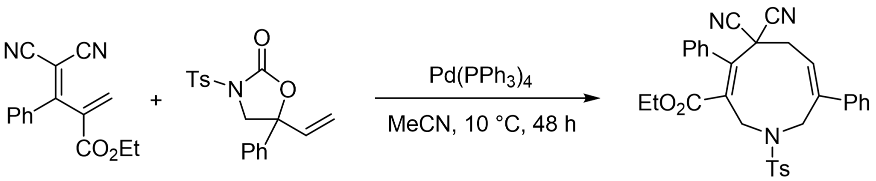

1a **9** **10**

To an over-dried Schlenk tube was added $Pd(PPh_3)_4$ (5 mol %), after which the tube was evacuated and back-filled with argon three times. Subsequently, under the protection of argon, a solution of allylidenemalononitrile **1a** (0.10 mmol) and oxazolidinone **9**[4] (0.15 mmol) in dry MeCN (1.0 mL) was added via syringe and the reaction mixture was stirred at 10 °C for 48 hours. Then the mixture was concentrated and purified by column chromatography on silica gel (petroleum ether/ ethyl acetate = 20/1 to 6/1) to afford the corresponding **10** (33.7 mg) as white solid in 61% yield, which were dried under vacuum and further analyzed by ^{1}H NMR, ^{13}C NMR, HRMS, *etc*.

ethyl-(3*E*,7*Z*)-5,5-dicyano-4,8-diphenyl-1-tosyl-2,5,6,9-tetrahydro-1H-azonine-3-carboxylate 10

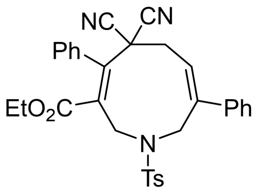

Prepared according to the general procedure to afford **10** (33.7 mg, m. p. = 152 – 154 °C) in 61% yield as white solid. The regioisomeric ratio was determined to be >20:1 by crude ^{1}H NMR analysis.

NMR and HRMS data for the product **10**:

^{1}H NMR (600 MHz, $CDCl_3$, 55 °C) δ (ppm): 7.43 – 7.34 (m, 7H), 7.34 – 7.27 (m, 3H), 7.18 (d, J = 5.4 Hz, 2H), 7.12 (d, J = 8.4 Hz, 2H), 6.05 (t, J = 9.0 Hz, 1H), 4.32 (brs, 4H), 3.84 (q, J = 7.8 Hz, 2H), 3.57 (brs, 2H), 2.38 (s, 3H), 0.85 (t, J = 7.2 Hz, 3H).

^{13}C NMR (150 MHz, $CDCl_3$, 55 °C) δ (ppm): 166.4, 143.6, 143.3, 140.0, 137.1, 136.7, 135.8, 135.6, 129.73, 129.70, 128.7, 128.6, 128.4, 128.2, 127.2, 126.8, 123.7, 114.1, 61.7, 46.8, 45.2, 40.1, 38.8, 21.4, 13.4.

HRMS (ESI-TOF) m/z: **[M + Na]$^+$** calculated for $C_{32}H_{29}N_3O_4SNa^+$: 574.1771, found: 574.1769.

7.2 Procedure for the synthesis of seven-membered product ***11***

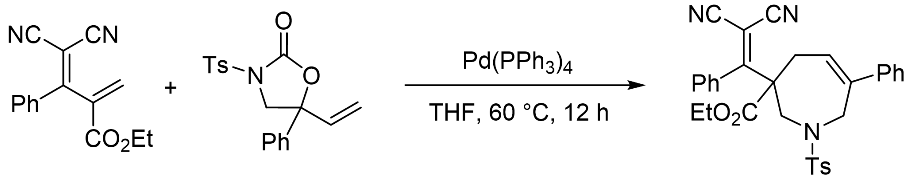

1a **9** **11**

To an over-dried Schlenk tube was added $Pd(PPh_3)_4$ (5 mol %), after which the tube was evacuated and back-filled with argon three times. Subsequently, under the protection of argon, a solution of allylidenemalononitrile **1a** (0.10 mmol) and oxazolidinone **9** (0.15 mmol) in dry THF (1.0 mL) was added via syringe and the reaction mixture was stirred at 60 °C for 12 hours. Then the mixture was concentrated and purified by column chromatography on silica gel (petroleum ether/ dichloromethane = 3/1 to 1/1, then petroleum ether/ethyl acetate = 10/1 to 6/1) to afford the corresponding **11** (46.9 mg) as white solid in 85% yield, which were dried under vacuum and further analyzed by ^{1}H NMR, ^{13}C NMR, HRMS, *etc*.

ethyl-3-(2,2-dicyano-1-phenylvinyl)-6-phenyl-1-tosyl-2,3,4,7-tetrahydro-1H-azepine-3-carboxylate 11

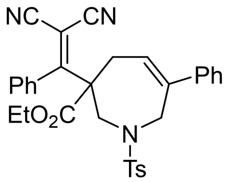

Prepared according to the general procedure to afford **11** (46.9 mg, m. p. = 101 – 105 °C) in 85% yield as white solid. The regioisomeric ratio was determined to be >20:1 by crude ^{1}H NMR analysis.

NMR and HRMS data for the product **11**:

^{1}H NMR (600 MHz, CDCl$_3$) δ (ppm): 7.78 (d, *J* = 8.4 Hz, 2H), 7.63 – 7.47 (m, 4H), 7.41 (d, *J* = 7.8 Hz, 2H), 7.34 – 7.28 (m, 4H), 7.17 (d, *J* = 8.4 Hz, 2H), 5.52 (t, *J* = 7.8 Hz, 1H), 4.64 (d, *J* = 16.2 Hz, 1H), 4.45 – 4.33 (m, 3H), 4.23 (d, *J* = 15.0 Hz, 1H), 3.88 (d, *J* = 16.8 Hz, 1H), 3.07 (dd, *J* = 14.4, 6.0 Hz, 1H), 2.50 (s, 3H), 2.45 (dd, *J* = 14.4, 8.4 Hz, 1H), 1.41 (t, *J* = 7.2 Hz,

3H).

^{13}C NMR (150 MHz, CDCl$_3$) δ (ppm): 181.3, 170.1, 144.2, 140.0, 139.9, 135.2, 134.8, 130.3, 130.1, 128.9, 128.5, 127.9, 127.1, 126.8, 125.9, 122.2, 111.7, 111.4, 90.4, 63.1, 58.2, 54.3, 53.6, 33.6, 21.5, 14.1.

HRMS (ESI-TOF) m/z: **[M + Na]$^+$** calculated for $C_{32}H_{29}N_3O_4SNa^+$: 574.1771, found: 574.1772.

8. General Procedure and Mechanism Studies for the [2+2] Cycloaddition

8.1 General procedure for the transannular [2+2] cycloaddition

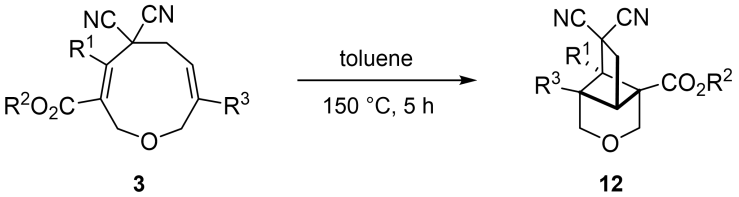

To an over-dried Schlenk tube was added the nine-membered products **3** (0.10 mmol), after which the tube was evacuated and back-filled with argon three times. Subsequently, under the protection of argon, dry toluene (1.0 mL) was added via syringe and the reaction mixture was stirred at 150 °C for 5 hours. Then the mixture was cooled to room temperature and purified by column chromatography on silica gel (petroleum ether/ ethyl acetate = 20/1 to 10/1 to afford the corresponding **12** in 64–86% yields.

ethyl-9,9-dicyano-1,6-diphenyl-4-oxatricyclo[4.3.0.0^{2,7}]nonane-2-carboxylate 12a

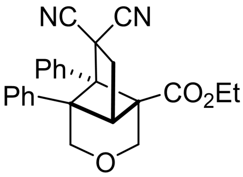

Prepared according to the general procedure to afford **12a** (30.2 mg, m. p. = 158 – 160 °C) in 76% yield as white solid. The diastereomeric ratio was determined to be >20:1 by crude ^{1}H NMR analysis.

NMR and HRMS data for the product **12a**:

^{1}H NMR (600 MHz, CDCl$_3$) δ (ppm): 8.15 (d, *J* = 7.2 Hz, 2H), 7.61 – 7.47 (m, 4H), 7.46 – 7.33 (m, 3H), 7.10 (brs, 1H), 4.48 (d, *J* = 12.0 Hz, 1H), 4.33 – 4.26 (m, 1H), 4.24 – 4.20 (m, 1H), 4.18 (d, *J* = 12.0 Hz, 1H), 4.14 (d, *J* = 11.4 Hz, 1H), 3.86 (d, *J* = 11.4 Hz, 1H), 3.31 (d, *J* = 12.6 Hz, 1H), 3.28 (s, 1H), 3.02 (d, *J* = 12.6 Hz, 1H), 1.30 (t, *J* = 7.2 Hz, 3H).

^{13}C NMR (150 MHz, CDCl$_3$) δ (ppm): 169.1, 135.1, 131.9, 130.0, 129.1, 128.9, 128.8, 128.7, 127.4, 115.5, 114.9, 71.1, 70.8, 66.5, 62.4, 58.2, 57.8, 46.3, 38.3, 37.8, 13.6.

HRMS (ESI-TOF) m/z: **[M + Na]$^+$** calculated for $C_{25}H_{22}N_2O_3Na^+$: 421.1523, found: 420.1524.

ethyl-1-(3-chlorophenyl)-9,9-dicyano-6-phenyl-4-oxatricyclo[4.3.0.0^{2,7}]nonane-2-carboxyl

ate 12b

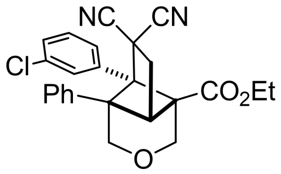

Prepared according to the general procedure to afford **12b** (33.3 mg, m. p. = 187 – 192 °C) in 77% yield as white solid. The diastereomeric ratio was determined to be >20:1 by crude ^{1}H NMR analysis.

NMR and HRMS data for the product **12b**:

^{1}H NMR (600 MHz, CDCl$_3$) δ (ppm): 8.22 (s, 1H), 8.09 – 7.99 (m, 1H), 7.65 – 7.46 (m, 3H), 7.44 – 7.37 (m, 3H), 7.10 (brs, 1H), 4.45 (d, *J* = 11.4 Hz, 1H), 4.34 – 4.21 (m, 2H), 4.13 (t, *J* = 10.8 Hz, 2H), 3.86 (d, *J* = 11.4 Hz, 1H), 3.30 (d, *J* = 12.6 Hz, 1H), 3.27 (s, 1H), 3.02 (d, *J* = 12.0 Hz, 1H), 1.35 (t, *J* = 7.2 Hz, 3H).

^{13}C NMR (150 MHz, CDCl$_3$) δ (ppm): 168.9, 135.0, 134.7, 134.1, 130.1, 129.4, 129.2, 129.0, 128.9, 127.5, 126.6, 115.1, 114.6, 70.8, 70.5, 66.5, 62.7, 58.3, 58.1, 46.6, 38.3, 37.8, 13.6.

HRMS (ESI-TOF) m/z: **[M + Na]$^+$** calculated for $C_{25}H_{21}{}^{35}ClN_2O_3Na^+$: 455.1133, found: 455.1129; calculated for $C_{25}H_{21}{}^{37}ClN_2O_3Na^+$: 457.1103, found: 457.1110.

ethyl-9,9-dicyano-6-phenyl-1-(p-tolyl)-4-oxatricyclo[4.3.0.0^{2,7}]nonane-2-carboxylate 12c

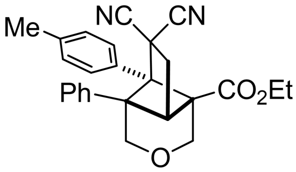

Prepared according to the general procedure to afford **12c** (30.1 mg, m. p. = 161 – 165 °C) in 73% yield as white solid. The diastereomeric ratio was determined to be >20:1 by crude ^{1}H NMR analysis.

NMR and HRMS data for the product **12c**:

^{1}H NMR (600 MHz, CDCl$_3$) δ (ppm): 8.02 (d, *J* = 8.4 Hz, 2H), 7.52 (brs, 1H), 7.44 – 7.30 (m, 5H), 7.08 (brs, 1H), 4.47 (d, *J* = 10.8 Hz, 1H), 4.33 – 4.26 (m, 1H), 4.24 – 4.15 (m, 1H), 4.18 (d, *J* = 12.0 Hz, 1H), 4.13 (d, *J* = 11.4 Hz, 1H), 3.84 (d, *J* = 11.4 Hz, 1H), 3.30 (d, *J* = 13.2 Hz, 1H), 3.26 (s, 1H), 3.00 (d, *J* = 12.6 Hz, 1H), 2.44 (s, 3H), 1.30 (t, *J* = 7.2 Hz, 3H).

^{13}C NMR (150 MHz, CDCl$_3$) δ (ppm): 169.2, 139.0, 135.2, 129.6, 128.9, 128.8, 128.7, 128.6, 127.4, 115.6, 115.0, 71.1, 70.8, 66.5, 62.3, 58.1, 57.7, 46.2, 38.3, 37.7, 21.2, 13.6.

HRMS (ESI-TOF) m/z: **[M + Na]$^+$** calculated for $C_{26}H_{24}N_2O_3Na^+$: 435.1679, found: 435.1685.

ethyl-9,9-dicyano-1-(naphthalen-2-yl)-6-phenyl-4-oxatricyclo[4.3.0.0^{2,7}]nonane-2-carboxylate 12d

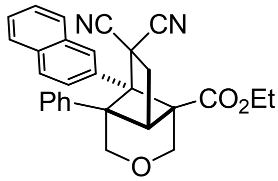

Prepared according to the general procedure to afford **12d** (36.8 mg, m. p. = 205 – 206 °C) in 82% yield as white solid. The diastereomeric ratio was determined to be >20:1 by crude ^{1}H NMR analysis.

NMR and HRMS data for the product **12d**:

^{1}H NMR (600 MHz, CDCl$_3$) δ (ppm): 8.68 (s, 1H), 8.24 (d, *J* = 11.4 Hz, 1H), 8.03 (d, *J* = 9.0 Hz, 1H), 7.98 – 7.91 (m, 2H), 7.67 – 7.51 (m, 3H), 7.48 – 7.33 (m, 3H), 7.14 (brs, 1H), 4.57 (d, *J* = 11.4 Hz, 1H), 4.37 – 4.29 (m, 1H), 4.28 – 4.21 (m, 1H), 4.24 (d, *J* = 11.4 Hz, 1H), 4.19 (d, *J* = 12.0 Hz, 1H), 3.90 (d, *J* = 11.4 Hz, 1H), 3.36 (d, *J* = 13.2 Hz, 1H), 3.33 (s, 1H), 3.07 (d, *J* = 12.0 Hz, 1H), 1.33 (t, *J* = 7.2 Hz, 3H).

^{13}C NMR (150 MHz, CDCl$_3$) δ (ppm): 169.2, 135.1, 133.11, 133.08, 130.1, 129.4, 129.1, 129.0, 128.8, 128.7, 128.6, 127.6, 127.2, 126.7, 125.4, 115.6, 115.0, 71.3, 70.9, 66.6, 62.5, 58.4, 58.0, 46.4, 38.4, 37.9, 13.7.

HRMS (ESI-TOF) m/z: **[M + Na]$^+$** calculated for $C_{29}H_{24}N_2O_3Na^+$: 471.1679, found: 471.1684.

ethyl-9,9-dicyano-6-phenyl-1-(thiophen-2-yl)-4-oxatricyclo[4.3.0.0^{2,7}]nonane-2-carboxylate 12e

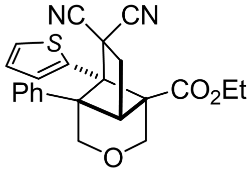

Prepared according to the general procedure to afford **12e** (29.9 mg, m. p. = 171 – 175 °C) in 74% yield as colorless semisolid. The diastereomeric ratio was determined to be >20:1 by crude ^{1}H NMR analysis.

NMR and HRMS data for the product **12e**:

^{1}H NMR (600 MHz, CDCl$_3$) δ (ppm): 7.80 (d, *J* = 4.8 Hz, 1H), 7.56 (d, *J* = 4.8 Hz, 1H), 7.39 (t, *J* = 7.8 Hz, 1H), 7.34 (t, *J* = 7.2 Hz, 1H), 7.32 – 7.26 (m, 2H), 7.23 (dd, *J* = 4.8, 3.0 Hz, 1H), 7.04 (d, *J* = 7.2 Hz, 1H), 4.57 (d, *J* = 10.8 Hz, 1H), 4.35 (d, *J* = 11.4 Hz, 1H), 4.27 – 4.20 (m, 1H), 4.16 – 4.08 (m, 1H), 4.13 (d, *J* = 12.0 Hz, 1H), 3.84 (d, *J* = 12.6 Hz, 1H), 3.37 (s, 1H), 3.32 (d, *J* = 13.2 Hz, 1H), 3.02 (d, *J* = 13.2 Hz, 1H), 1.21 (t, *J* = 7.2 Hz, 3H).

^{13}C NMR (150 MHz, CDCl$_3$) δ (ppm): 168.7, 134.5, 132.7, 129.4, 128.91, 128.86, 127.6, 127.3, 126.8, 115.4, 114.5, 70.0, 68.0, 65.7, 62.2, 58.6, 58.0, 45.2, 38.6, 37.3, 13.5.

HRMS (ESI-TOF) m/z: **[M + Na]$^+$** calculated for $C_{23}H_{20}N_2O_3SNa^+$: 427.1087, found: 427.1092.

tert-butyl-9,9-dicyano-1,6-diphenyl-4-oxatricyclo[4.3.0.0^{2,7}]nonane-2-carboxylate 12f

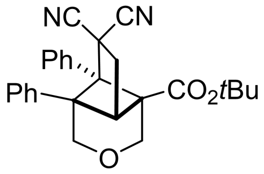

Prepared according to the general procedure to afford **12f** (29.9 mg) in 70% yield as colorless semisolid. The diastereomeric ratio was determined to be >20:1 by crude ^{1}H NMR analysis.

NMR and HRMS data for the product **12f**:

^{1}H NMR (600 MHz, CDCl$_3$) δ (ppm): 8.17 (d, *J* = 7.8 Hz, 2H), 7.63 (brs, 1H), 7.55 (t, *J* = 7.2 Hz, 2H), 7.48 (t, *J* = 9.6 Hz, 1H), 7.45 – 7.35 (m, 3H), 7.11 (brs, 1H), 4.40 (d, *J* = 11.4 Hz, 1H), 4.14 (d, *J* = 12.0 Hz, 1H), 4.08 (d, *J* = 10.8 Hz, 1H), 3.80 (d, *J* = 12.6 Hz, 1H), 3.24 (d, *J* = 13.8 Hz, 2H), 3.04 (d, *J* = 13.2 Hz, 1H), 1.54 (s, 9H).

^{13}C NMR (150 MHz, CDCl$_3$) δ (ppm): 168.2, 135.4, 132.1, 130.1, 129.0, 128.9, 128.79, 128.76, 127.6, 115.6, 115.1, 84.3, 71.2, 70.8, 67.0, 58.8, 57.7, 46.0, 38.6, 37.9, 27.9.

HRMS (ESI-TOF) m/z: **[M + Na]$^+$** calculated for $C_{27}H_{26}N_2O_3Na^+$: 449.1836, found: 449.1838.

ethyl-6-(3-bromophenyl)-9,9-dicyano-1-phenyl-4-oxatricyclo[4.3.0.0^{2,7}]nonane-2-carboxylate 12g

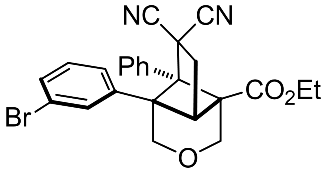

Prepared according to the general procedure to afford **12g** (41.0 mg, m. p. = 160 – 162 °C) in 86% yield as colorless semisolid. The diastereomeric ratio was determined to be >20:1 by crude ^{1}H NMR analysis.

NMR and HRMS data for the product **12g**:

^{1}H NMR (600 MHz, CDCl$_3$) δ (ppm): 8.11 (d, *J* = 7.8 Hz, 2H), 7.56 (t, *J* = 6.6 Hz, 2H), 7.53 – 7.41 (m, 3H), 7.26 (brs, 2H), 4.55 – 4.40 (m, 1H), 4.34 – 4.27 (m, 1H), 4.24 – 4.17 (m, 1H), 4.17 – 4.11 (m, 2H), 3.84 (d, *J* = 11.4 Hz, 1H), 3.33 (d, *J* = 13.2 Hz, 1H), 3.26 (s, 1H), 2.96 (d, *J* = 13.2 Hz, 1H), 1.30 (t, *J* = 7.2 Hz, 3H).

^{13}C NMR (150 MHz, CDCl$_3$) δ (ppm): 168.9, 137.5, 131.9, 131.5, 130.4, 129.3, 129.2, 129.0, 128.6, 125.6, 122.9, 115.3, 114.9, 71.2, 70.6, 66.4, 62.5, 58.1, 57.6, 46.3, 38.2, 37.7, 13.6.

HRMS (ESI-TOF) m/z: **[M + Na]$^+$** calculated for $C_{25}H_{21}{}^{79}BrN_2O_3Na^+$: 499.0628, found: 499.0622; calculated for $C_{25}H_{21}{}^{81}BrN_2O_3Na^+$: 501.0607, found: 501.0606.

ethyl-9,9-dicyano-6-(4-ethylphenyl)-1-phenyl-4-oxatricyclo[4.3.0.0^{2,7}]nonane-2-carboxylate 12h

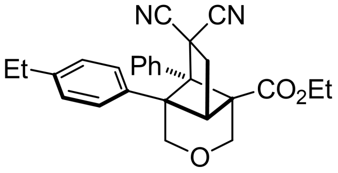

Prepared according to the general procedure to afford **12h** (31.6 mg, m. p. = 168 – 169 °C) in 74% yield as colorless semisolid. The diastereomeric ratio was determined to be >20:1 by crude ^{1}H NMR analysis.

NMR and HRMS data for the product **12h**:

^{1}H NMR (600 MHz, CDCl$_3$) δ (ppm): 8.15 (d, *J* = 7.8 Hz, 2H), 7.55 (t, *J* = 7.8 Hz, 2H), 7.49 (t, *J* = 7.8 Hz, 1H), 7.43 (brs, 1H), 7.21 (d, *J* = 8.4 Hz, 2H), 7.01 (brs, 1H), 4.45 (d, *J* = 11.4 Hz, 1H), 4.34 – 4.27 (m, 1H), 4.25 – 4.18 (m, 1H), 4.15 (d, *J* = 11.4 Hz, 1H), 4.12 (d, *J* = 11.4 Hz,

1H), 3.83 (d, J = 11.4 Hz, 1H), 3.28 (d, J = 13.2 Hz, 1H), 3.25 (s, 1H), 3.02 (d, J = 12.0 Hz, 1H), 2.65 (q, J = 7.2 Hz, 2H), 1.31 (t, J = 7.2 Hz, 3H), 1.23 (t, J = 7.2 Hz, 3H).

^{13}C NMR (150 MHz, CDCl$_3$) δ (ppm): 169.2, 144.9, 132.11, 132.06, 130.0, 129.0, 128.8, 128.7, 128.3, 115.6, 114.9, 71.2, 70.9, 66.5, 62.3, 58.2, 57.7, 46.4, 38.3, 37.8, 28.4, 15.2, 13.6.

HRMS (ESI-TOF) m/z: **[M + Na]$^+$** calculated for $C_{27}H_{26}N_2O_3Na^+$: 449.1836, found: 449.1839.

ethyl-9,9-dicyano-6-(3,4-dichlorophenyl)-1-phenyl-4-oxatricyclo[4.3.0.0^{2,7}]nonane-2-carboxylate 12i

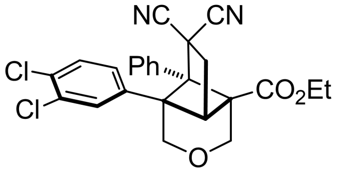

Prepared according to the general procedure to afford **12i** (39.2 mg, m. p. = 189 – 193 °C) in 84% yield as colorless semisolid. The diastereomeric ratio was determined to be >20:1 by crude ^{1}H NMR analysis.

NMR and HRMS data for the product **12i**:

^{1}H NMR (600 MHz, CDCl$_3$) δ (ppm): 8.09 (d, J = 8.4 Hz, 2H), 7.74 – 7.29 (m, 5H), 7.20 – 6.89 (m, 1H), 4.45 (d, J = 12.0 Hz, 1H), 4.34 – 4.27 (m, 1H), 4.25 – 4.17 (m, 1H), 4.12 (d, J = 11.4 Hz, 2H), 3.82 (d, J = 11.4 Hz, 1H), 3.35 (d, J = 12.6 Hz, 1H), 3.25 (s, 1H), 2.92 (d, J = 12.6 Hz, 1H), 1.30 (t, J = 7.2 Hz, 3H).

^{13}C NMR (150 MHz, CDCl$_3$) δ (ppm): 168.8, 135.4, 133.2, 131.4, 131.0, 129.4, 129.3, 129.2, 129.1, 128.5, 126.7, 115.1, 115.0, 71.2, 70.5, 66.4, 62.6, 58.2, 57.3, 46.3, 38.2, 37.7, 13.6.

HRMS (ESI-TOF) m/z: **[M + Na]$^+$** calculated for $C_{25}H_{20}{}^{35}Cl_2N_2O_3Na^+$: 489.0743, found: 489.0750; calculated for $C_{25}H_{20}{}^{35}Cl^{37}ClN_2O_3Na^+$: 491.0714, found: 491.0726; calculated for $C_{25}H_{20}{}^{37}Cl_2N_2O_3Na^+$: 493.0684, found: 493.0688.

ethyl-9,9-dicyano-1-phenyl-6-(thiophen-2-yl)-4-oxatricyclo[4.3.0.0^{2,7}]nonane-2-carboxylate 12j

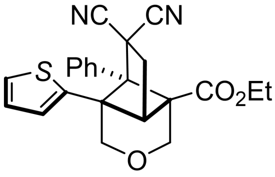

Prepared according to the general procedure to afford **12j** (25.9 mg, m. p. = 198 – 199 °C) in 64% yield as colorless semisolid. The diastereomeric ratio was determined to be >20:1 by crude ^{1}H NMR analysis.

NMR and HRMS data for the product **12j**:

^{1}H NMR (600 MHz, CDCl$_3$) δ (ppm): 8.17 (d, *J* = 7.2 Hz, 2H), 7.55 (t, *J* = 6.6 Hz, 2H), 7.49 (t, *J* = 7.8 Hz, 1H), 7.45 (d, *J* = 4.8 Hz, 1H), 7.10 (d, *J* = 2.4 Hz, 1H), 7.07 (dd, *J* = 4.8, 3.6 Hz, 1H), 4.45 – 4.33 (m, 1H), 4.36 (d, *J* = 10.8 Hz, 1H), 4.33 – 4.25 (m, 1H), 4.15 (d, *J* = 11.4 Hz, 1H), 4.05 (d, *J* = 12.0 Hz, 1H), 3.81 (d, *J* = 11.4 Hz, 1H), 3.37 (s, 2H), 3.28 (s, 1H), 1.40 (t, *J* = 7.2 Hz, 3H).

^{13}C NMR (150 MHz, CDCl$_3$) δ (ppm): 169.1, 137.8, 131.3, 129.3, 129.0, 128.9, 128.1, 127.2, 127.0, 114.9, 114.2, 73.0, 70.7, 66.1, 62.7, 58.3, 54.9, 46.8, 39.1, 38.1, 13.8.

HRMS (ESI-TOF) m/z: **[M + Na]$^+$** calculated for $C_{23}H_{20}N_2O_3SNa^+$: 427.1087, found: 427.1087.

8.2 Mechanism studies for the transannular [2+2] cycloaddition

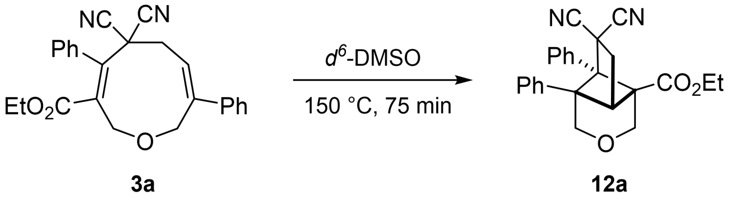

To an over-dried NMR tube was added the nine-membered products **3** (20 mg). Subsequently, dry *d*^{6}-DMSO (0.6 mL) was added via syringe and the reaction mixture was probed by ^{1}H NMR at 150 °C for 75 min and ^{1}H NMR spectra results were collected every 15 minutes.

From the ^{1}H NMR spectra results, it is clear that the peaks of the product are increasing and the peaks of the substrate material are decreasing. When zooming in on the spectrum at 6.29 ppm, we could still observe a new small triplet peak overlapping in the substrate's triplet peak, which might support an isomerization of the styryl moiety (*E* to *Z*).

9. Preliminary Evaluation of Biological Activity for Compounds 12

9.1 Cell culture and cellular proliferation assay

The A549, PC12, SH-SY5Y, A375 and MDA-MB231 cell line were obtained from the Type Culture Collection of the Chinese Academy of Sciences, Shanghai, China. A549 and PC12 cells were grown in RPMI 1640 (Hyclone, USA) supplemented with 10% fetal bovine serum (FBS, Gibco, USA), while SH-SY5Y, A375 and MDA-MB231 cells were grown in DMEM (Hyclone, USA) supplemented with 10% fetal bovine serum (FBS, Gibco, USA). All the cells were maintained at 37 °C in a humidified incubator containing 5% CO_2. A 20 μM chemical stock solution was prepared by dissolving in DMSO before cell viability assays. Cell viability was determined by MTT (Sigma-Aldrich) assays. All the five cell lines were seeded in 96-well plates 24 h earlier before treated by the chemicals for 24 h. Chemical treatment concentration set to 20 μM and paclitaxel was prepared as a positive control (5 μM, 24 h). After the treatment, 200 μL fresh medium containing 20 μL MTT (5 mg/mL) was added to each pore to replace the chemical containing medium and incubated for 4 h. Then discarded the medium and added 150 μL DMSO to dissolve purple crystals. The absorbance value at 570 nm was determined. The mean percentage of cell survival rates was determined from data of three individual experiments.

*9.2 The Mean Inhibitory Ratio of Compounds **12** against A Panel of Cancer Cell Lines*

MTT assay was applied to determine the cell viability after 24 h treatment of tested compounds. All the typical cancer cell lines were obtained from American Type Culture Collection (ATCC, USA). PTX is FDA approved to be used for AIDS-related Kaposi sarcoma, breast cancer, non-small cell lung cancer, and ovarian cancer. Results exerted that PTX showed a significant inhibitory effect on most type of cancer, except triple negative breast cancer (TNBC) MDA-MB-231 at the concentration of 5 μM. Interestingly, compound **12a** had a more critical effect on MDA-MB-231 than other compounds at the concentration of 20 μM, suggesting a promising application for TNBC therapy after further exploration. However, its effect on lung cancer A-549 was least effective compare to other compounds. Compounds **12c**, **12j** and **12i** could suppress A-549 proliferation significantly, while compounds **12e** and **12f** displayed a moderate inhibitory effect. PC12 is a rat pheochromocytoma cell line. Compounds **12c** and **12h** could block the growth of PC12 slightly, while compounds **12i** and **12j** manifested

a dramatic inhibitory effect. SH-SY-5Y is a neuroblastoma cell line. Most of synthesized compounds could interfere with the proliferative ability of SH-SY-5Y, except compound **12j**. A375 cells are human melanoma cancer cells containing the endogenous B-Raf mutation V600E. The ratio of inhibited A375 cells for all the tested compounds were above 50%.

Table S3. The mean inhibitory ratio.[a]

Compounds	Inhibitory Ratio (%)				
	A549	PC12	SH-SY5Y	A375	MDA-MB231
**12a**	6.82	25.41	68.31	64.36	46.33
**12b**	27.72	4.41	31.10	54.72	40.32
**12c**	74.76	43.18	22.39	38.23	9.53
**12d**	9.07	14.14	64.31	79.73	41.57
**12e**	59.50	16.88	67.40	70.63	35.63
**12f**	51.40	22.66	29.92	42.25	17.58
**12g**	30.57	19.77	56.87	40.53	30.00
**12h**	38.93	45.39	46.35	66.19	37.04
**12i**	73.76	61.87	39.36	36.22	22.27
**12j**	74.89	72.66	10.87	35.98	1.48
**PTX**	66.14	67.63	57.47	77.23	38.75

[a] Each compound was tested in triplicate; the data are presented as the mean values.

10. Experiments for Mechanism Studies

10.1 The tracking experiments

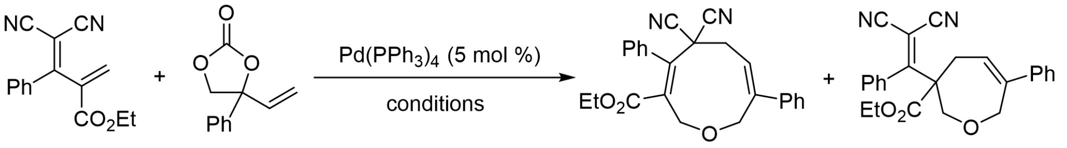

I. The tracking experiments of the [5+4] cyclization

Five reactions were parallelly carried out following the same operational procedure:

To over-dried Schlenk tubes was added $Pd(PPh_3)_4$ (5 mol %), after which the tube was evacuated and back-filled with argon three times. Subsequently, under the protection of argon, a solution of allylidenemalononitrile **1a** (0.10 mmol) and vinylethylene carbonate **2a** (0.15 mmol) in dry MeCN (1.0 mL) was added.

Then, the five reactions were stirred at 20 °C for 3 hours, 6 hours, 12 hours, 18 hours and 24 hours, respectively. After the corresponding time, the reaction mixture was filtered through a plug of silica (eluting with ethyl acetate) and concentrated, which were dried under vacuum and further analyzed by ^1H NMR with CH_2Br_2 as the internal standard. The results were listed as follow:

Table S4. The results of the tracking experiments **I**.

reaction time (h)	3	6	12	18	24
NMR yield of **3a** (%)	22	57	71	85	96
NMR yield of **4a** (%)	<1	<1	<1	<1	<1

II. The tracking experiments of the [5+2] cyclization

Six reactions were parallelly carried out following the same operational procedure:

To over-dried Schlenk tubes was added $Pd(PPh_3)_4$ (5 mol %), after which the tube was evacuated and back-filled with argon three times. Subsequently, under the protection of argon, a solution of allylidenemalononitrile **1a** (0.10 mmol) and vinylethylene carbonate **2a** (0.15 mmol) in dry THF (1.0 mL) was added.

Then, the six reactions were stirred at 60 °C for 45 min, 1.5 hours, 3 hours, 6 hours, 9 hours and 12 hours, respectively. After the corresponding time, the reaction mixture was cooled to room temperature, filtered through a plug of silica (eluting with ethyl acetate) and concentrated, which were dried under vacuum and further analyzed by ^{1}H NMR with CH_2Br_2 as the internal standard. The results were listed as follow:

Table S5. The results of the tracking experiments **II**.

reaction time (h)	0.75	1.5	3	6	9	12
NMR yield of **3a** (%)	87	85	71	60	36	<1
NMR yield of **4a** (%)	6	8	21	32	55	91

*10.2 The transformation from **3a** to **4a***

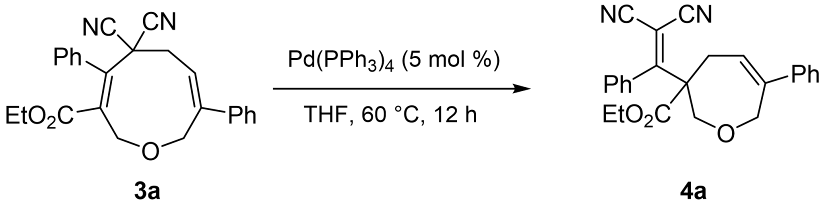

To an over-dried Schlenk tube was added $Pd(PPh_3)_4$ (5 mol %), after which the tube was evacuated and back-filled with argon three times. Subsequently, under the protection of argon, a solution of nine-membered product **3a** (39.8 mg, 0.10 mmol) in dry THF (1.0 mL) was added *via* syringe and the reaction mixture was stirred at 60 °C for 12 hours. Then the mixture was concentrated and purified by column chromatography on silica gel (petroleum ether/ethyl acetate = 10/1) to afford **4a** (38.2 mg) as pale yellow solid in 96% yields.

10.3 The effect of the loading of VEC on the regioisomeric ratio

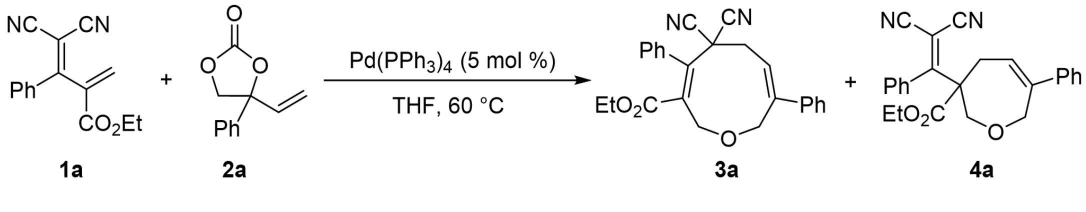

Four reactions were parallelly carried out under the similar operational procedure:

To over-dried Schlenk tubes was added $Pd(PPh_3)_4$ (5 mol %), after which the tube was evacuated and back-filled with argon three times. Subsequently, under the protection of argon, a solution of allylidenemalononitrile **1a** (0.10 mmol) and vinylethylene carbonate **2a** in dry THF (1.0 mL) was added.

The amounts of **2a** in the four reactions respectively refer to 0.15 mmol, 0.25 mmol, 0.35 mmol and 0.45 mmol. Then, the four reactions were stirred at 60 °C for 12 hours, respectively. After then, the reaction mixture was cooled to room temperature, filtered through a plug of silica (eluting with ethyl acetate) and concentrated, which were dried under vacuum and further analyzed by ^1H NMR with CH_2Br_2 as the internal standard. The results were listed as follow:

Table S6. The results of the control experiments.

The ratio of **1a**:**2a**	1:1.5	1:2.5	1:3.5	1:4.5
NMR yield of **3a** (%)	<1	72	85	92
NMR yield of **4a** (%)	91	21	6	<1

10.4 The effect of the loading of ligand on the regioisomeric ratio

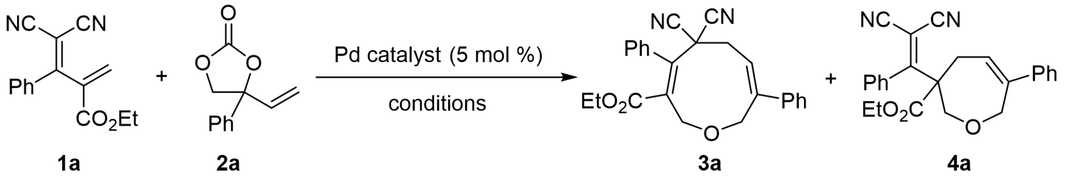

I. Condition: THF, 20 °C

Five reactions were parallelly carried out under the similar operational procedure:

To over-dried Schlenk tubes was added $Pd_2(dba)_3{\cdot}CHCl_3$ (2.5 mol %) and PPh_3, after which the tube was evacuated and back-filled with argon three times. Then under the protection of argon, dry THF (0.5 mL) was added and stirred at 20 °C for 1 hour. The amounts of PPh_3 in the five reactions respectively refer to 5 mol %, 10 mol %, 20 mol %, 40 mol %, and 80 mol %.

Subsequently, under the protection of argon, a solution of allylidenemalononitrile **1a** (0.10 mmol) and vinylethylene carbonate **2a** in dry THF (0.5 mL) was added respectively. Then, the five reactions were stirred at 20 °C for 12 hours. After then, the reaction mixture was filtered through a plug of silica (eluting with ethyl acetate) and concentrated, which were dried under

vacuum and further analyzed by ^1H NMR with CH_2Br_2 as the internal standard.

II. Condition: THF, 60 °C

Four reactions were parallelly carried out under the similar operational procedure:

To over-dried Schlenk tubes was added $Pd_2(dba)_3 \cdot CHCl_3$ (2.5 mol %) and PPh_3, after which the tube was evacuated and back-filled with argon three times. Then under the protection of argon, dry THF (0.5 mL) was added and stirred at 20 °C for 1 hour. The amounts of PPh_3 in the four reactions respectively refer to 5 mol %, 10 mol %, 20 mol % and 40 mol %.

Subsequently, under the protection of argon, a solution of allylidenemalononitrile **1a** (0.10 mmol) and vinylethylene carbonate **2a** in dry THF (0.5 mL) was added respectively. Then, the four reactions were stirred at 60 °C for 12 hours. After then, the reaction mixture was cooled to room temperature, filtered through a plug of silica (eluting with ethyl acetate) and concentrated, which were dried under vacuum and further analyzed by ^1H NMR with CH_2Br_2 as the internal standard.

III. Condition: MeCN, 60 °C

Four reactions were parallelly carried out under the similar operational procedure:

To over-dried Schlenk tubes was added $Pd_2(dba)_3 \cdot CHCl_3$ (2.5 mol %) and PPh_3, after which the tube was evacuated and back-filled with argon three times. Then under the protection of argon, dry MeCN (0.5 mL) was added and stirred at 20 °C for 1 hour. The amounts of PPh_3 in the four reactions respectively refer to 5 mol %, 10 mol %, 20 mol % and 40 mol %.

Subsequently, under the protection of argon, a solution of allylidenemalononitrile **1a** (0.10 mmol) and vinylethylene carbonate **2a** in dry MeCN (0.5 mL) was added respectively. Then, the four reactions were stirred at 60 °C for 12 hours. After then, the reaction mixture was cooled to room temperature, filtered through a plug of silica (eluting with ethyl acetate) and concentrated, which were dried under vacuum and further analyzed by ^1H NMR with CH_2Br_2 as the internal standard.

The results were listed as follow:

Table S7. The results of effect of the loading of ligand.[a]

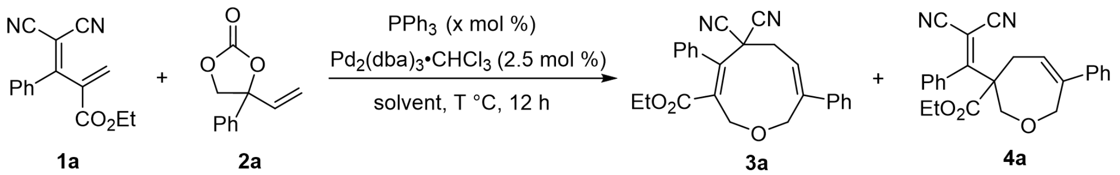

entry	PPh_3 (x mol%)	solvent	T (°C)	yield (%)[b]	**3a**:**4a**[c]
1	5	THF	20	18	>20:1
2	10	THF	20	66	>20:1
3	20	THF	20	82	10.5:1
4	40	THF	20	79	5.7:1
5	80	THF	20	54	1:1.7
6	5	THF	60	39	>20:1
7	10	THF	60	96	1.9:1
8	20	THF	60	89	2.2:1
9	40	THF	60	96	<1:20
10	5	MeCN	60	69	>20:1
11	10	MeCN	60	90	11.9:1
12	20	MeCN	60	57	8.2:1
13	40	MeCN	60	45	2.3:1

[a] Unless noted otherwise, the reactions were carried out with **1a** (0.10 mmol), **2a** (0.15 mmol), and Pd catalyst (5 mol %) in solvent (1.0 mL) for 12 h. And the Pd/ligand complex was pre-prepared with $Pd_2(dba)_3 \cdot CHCl_3$ and PPh_3 in solvent at rt for 1 h. [b] Yield was determined by ^{1}H-NMR analysis with CH_2Br_2 as the internal standard. [c] The ratio of **3a**:**4a** was determined by ^{1}H-NMR analysis of the crude reaction mixture. [d]

11. Crystal Data and Structure Refinement

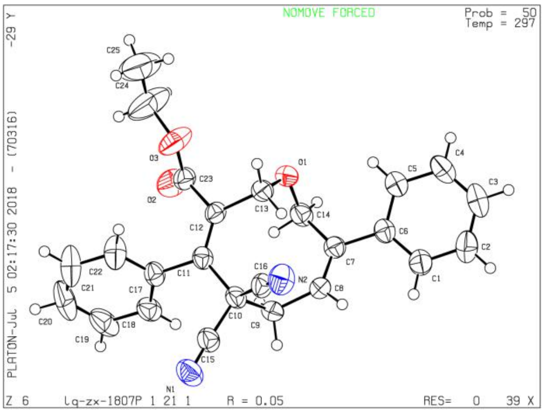

≡

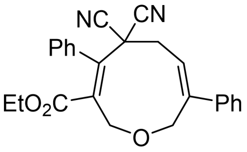

3a
CCDC 1943770

Identification code	**3a**
Empirical formula	$C_{25}H_{22}N_2O_3$
Formula weight	398.44
Temperature/K	296.6(3)
Crystal system	monoclinic
Space group	$P2_1$
a/Å	9.0919(5)
b/Å	9.9836(5)
c/Å	11.7719(5)
α/°	90
β/°	95.822(5)
γ/°	90
Volume/Å^3	1063.02(10)
Z	2
ρ_{calc}g/cm^3	1.245
μ/mm^{-1}	0.662
F(000)	420.0
Crystal size/mm^3	0.6 × 0.4 × 0.2
Radiation	CuKα (λ = 1.54184)
2Θ range for data collection/°	7.548 to 145.832
Index ranges	$-9 \le h \le 11, -7 \le k \le 12, -14 \le l \le 14$
Reflections collected	6415
Independent reflections	3358 [R_{int} = 0.0288, R_{sigma} = 0.0365]
Data/restraints/parameters	3358/1/272
Goodness-of-fit on F^2	1.042
Final R indexes [I>=2σ (I)]	R_1 = 0.0549, wR_2 = 0.1433
Final R indexes [all data]	R_1 = 0.0573, wR_2 = 0.1472
Largest diff. peak/hole / e Å^{-3}	0.24/-0.29

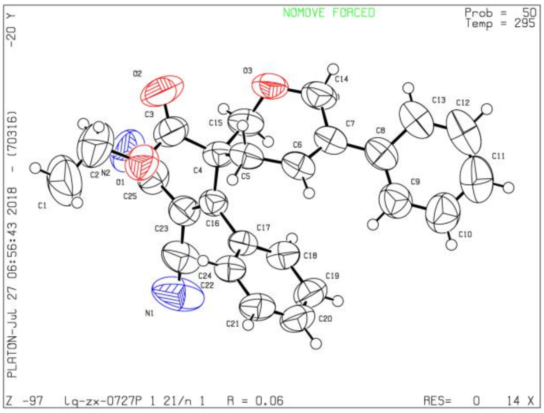

≡

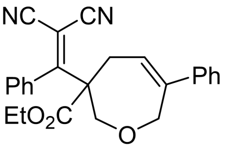

4a
CCDC 1943771

Identification code	**4a**
Empirical formula	$C_{25}H_{22}N_2O_3$
Formula weight	398.44
Temperature/K	295.4(4)
Crystal system	monoclinic
Space group	$P2_1/n$
a/Å	9.6576(5)
b/Å	13.2796(9)
c/Å	17.3578(10)
α/°	90
β/°	99.745(5)
γ/°	90
Volume/Å^3	2194.0(2)
Z	4
ρ_{calc}g/cm^3	1.206
μ/mm^{-1}	0.642
F(000)	840.0
Crystal size/mm^3	0.6 × 0.4 × 0.3
Radiation	CuKα (λ = 1.54184)
2Θ range for data collection/°	8.43 to 146.126
Index ranges	$-11 \le h \le 11$, $-16 \le k \le 16$, $-21 \le l \le 21$
Reflections collected	12330
Independent reflections	4284 [R_{int} = 0.0225, R_{sigma} = 0.0183]
Data/restraints/parameters	4284/0/272
Goodness-of-fit on F^2	1.044
Final R indexes [I>=2σ (I)]	R_1 = 0.0608, wR_2 = 0.1748
Final R indexes [all data]	R_1 = 0.0752, wR_2 = 0.1940
Largest diff. peak/hole / e Å^{-3}	0.18/-0.22

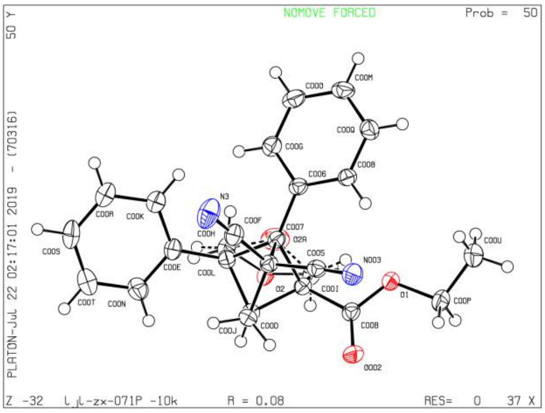

≡

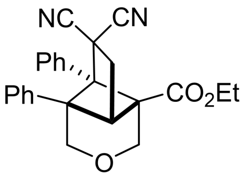

12a
CCDC 1943772

Identification code	**12a**
Empirical formula	$C_{25}H_{22}N_2O_3$
Formula weight	398.44
Temperature/K	225(100)
Crystal system	triclinic
Space group	P-1
a/Å	7.8691(9)
b/Å	9.8641(8)
c/Å	13.1840(9)
α/°	87.285(6)
β/°	85.531(8)
γ/°	74.852(9)
Volume/Å^3	984.42(16)
Z	2
ρ_{calc}g/cm^3	1.344
μ/mm^{-1}	0.715
F(000)	420.0
Crystal size/mm^3	0.6 × 0.2 × 0.1
Radiation	CuKα (λ = 1.54184)
2Θ range for data collection/°	6.728 to 144.716
Index ranges	-7 ≤ h ≤ 9, -12 ≤ k ≤ 12, -16 ≤ l ≤ 16
Reflections collected	10529
Independent reflections	3791 [R_{int} = 0.0750, R_{sigma} = 0.0560]
Data/restraints/parameters	3791/0/282
Goodness-of-fit on F^2	1.040
Final R indexes [I>=2σ (I)]	R_1 = 0.0793, wR_2 = 0.2132
Final R indexes [all data]	R_1 = 0.0877, wR_2 = 0.2314
Largest diff. peak/hole / e Å^{-3}	0.71/-0.47

12. References and Notes

1 (a) E. Krell, *Handbook of Laboratory Distillation*, Elseriver Publishing Company, Amsterdam-London-New York. 1963; (b) M. J. Rosengart, *The Technique of Distillation and Rectification in the Laboratory*, VEB Verlag Technik, Berlin, 1954; (c) F. Stage, *Angew. Chem.*, 1947, **19**, 175.

2 X. Jiang, D. Fu, X. Shi, S.; Wang and R. Wang, *Chem. Commun.*, 2011, **47**, 8289.

3 A. Khan, R. Zheng, Y. Kan, J. Ye, J. Xing and Y. J. Zhang, *Angew. Chem.*, *Int. Ed.*, 2014, **53**, 6439.

4 K. Ohmatsu, N. Imagawa and T. Ooi, *Nat. Chem.*, 2014, **6**, 47.

13. NMR Spectra

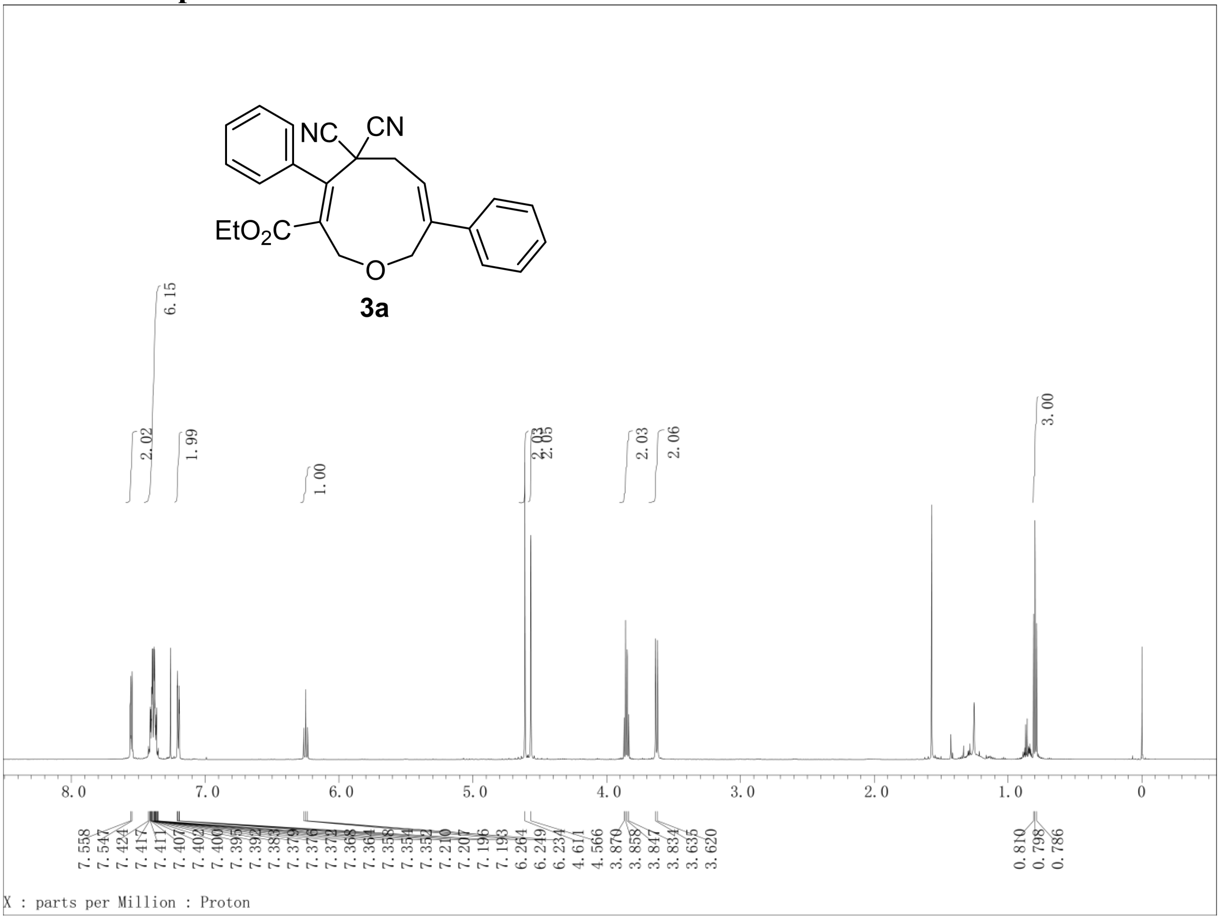

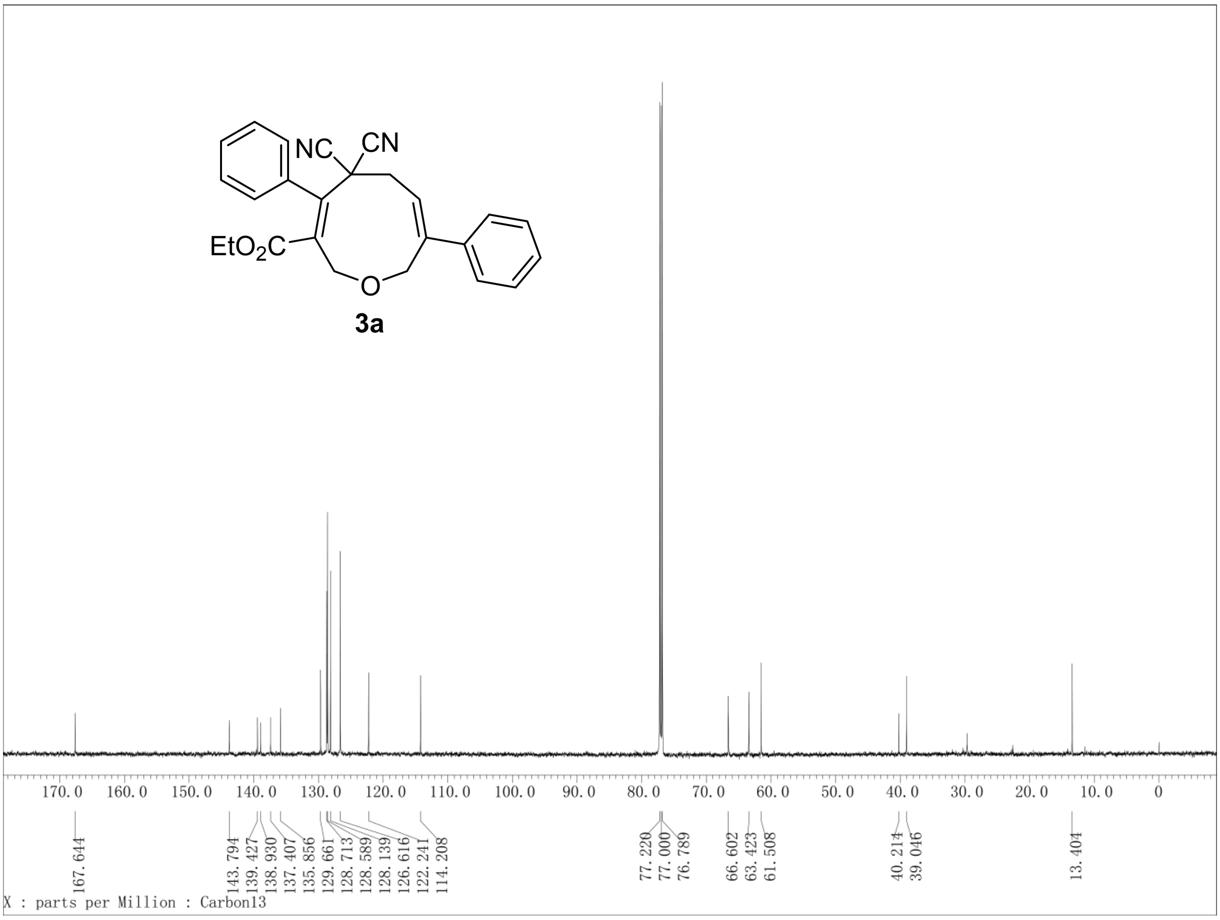

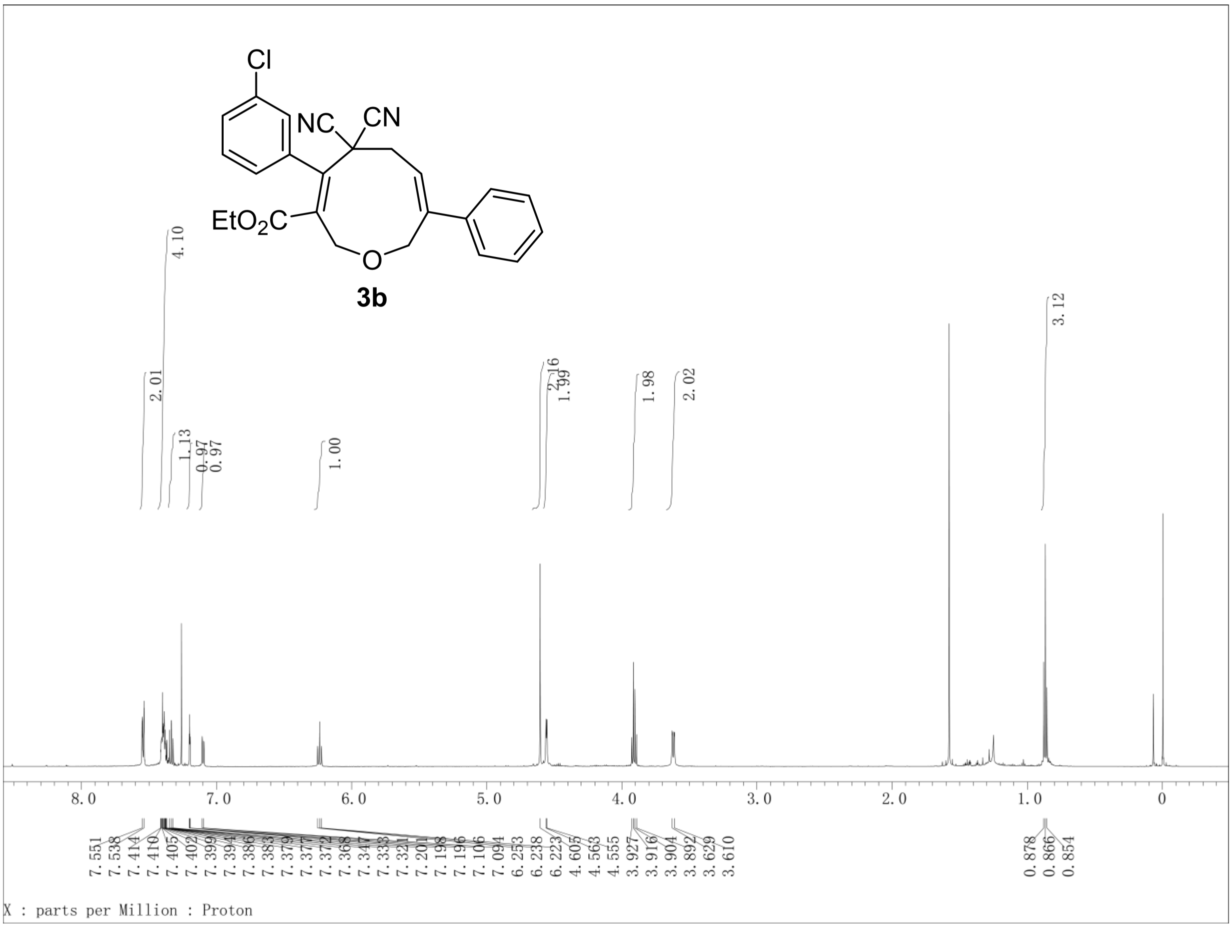
Cl
NC
CN
EtO2C
O
3b
2.01
4.10
1.13
0.97
0.97
1.00
2.16
1.99
1.98
2.02
3.12
8.0 7.0 6.0 5.0 4.0 3.0 2.0 1.0 0
7.551 7.538 7.414 7.410 7.405 7.402 7.399 7.394 7.386 7.383 7.379 7.377 7.372 7.368 7.347 7.333 7.321 7.201 7.198 7.196 7.106 7.094 6.253 6.238 6.223 4.605 4.563 4.555 3.927 3.916 3.904 3.892 3.629 3.610
0.878 0.866 0.854
X : parts per Million : Proton

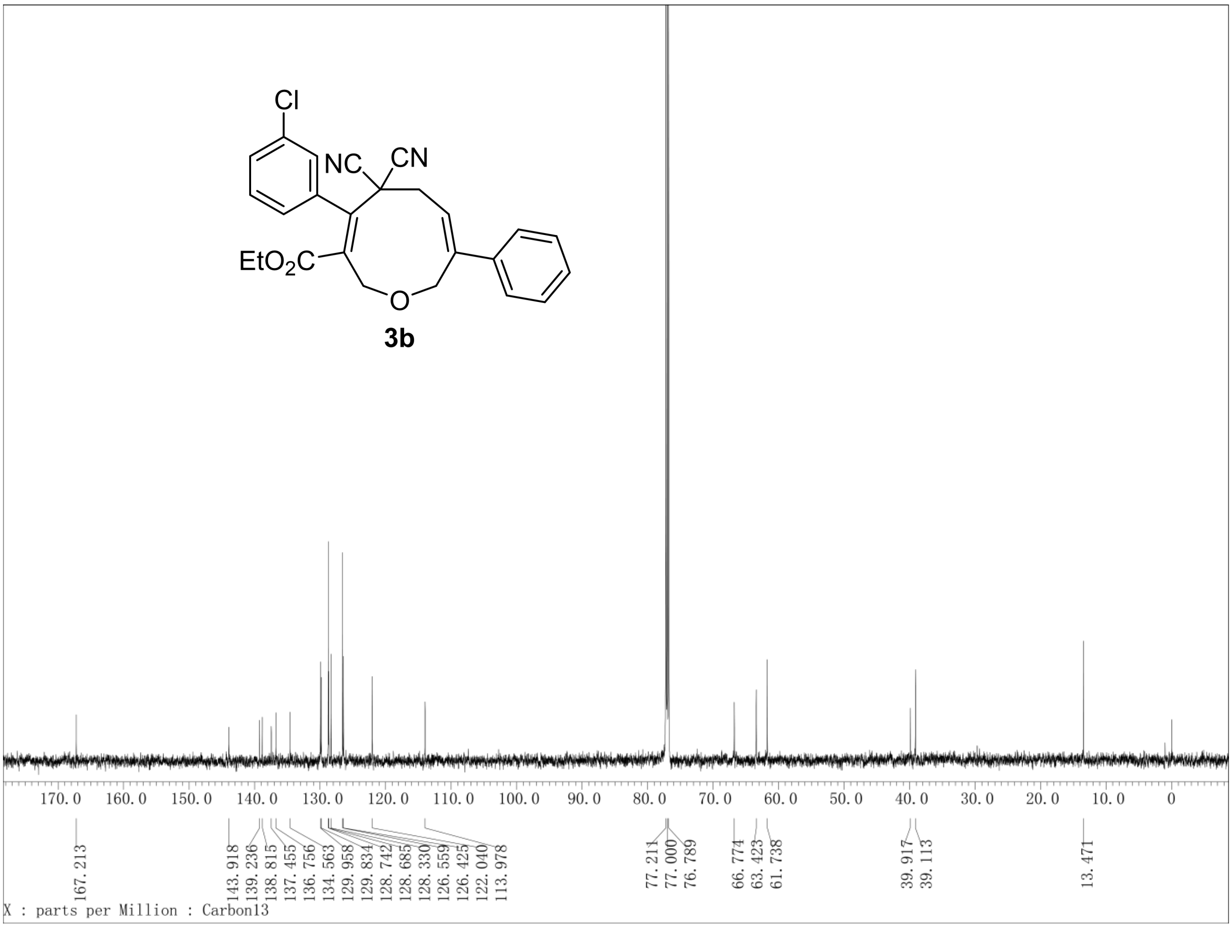
Cl
NC
CN
EtO2C
O
3b
170.0 160.0 150.0 140.0 130.0 120.0 110.0 100.0 90.0 80.0 70.0 60.0 50.0 40.0 30.0 20.0 10.0 0
167.213
143.918 139.236 138.815 137.455 136.756 134.563 129.958 129.834 128.742 128.685 128.330 126.559 126.425 122.040 113.978
77.211 77.000 76.789
66.774 63.423 61.738
39.917 39.113
13.471
X : parts per Million : Carbon13

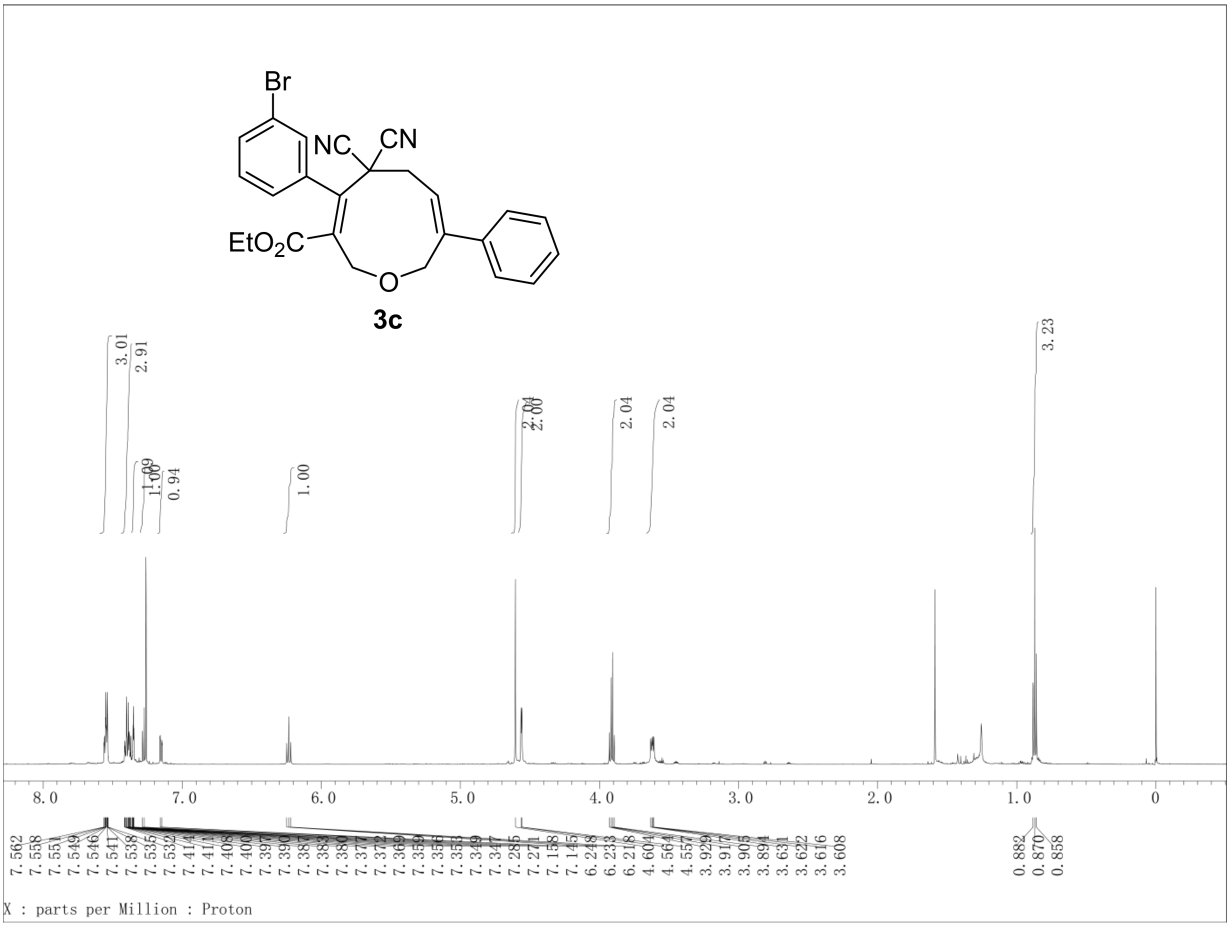
Br
NC
CN
EtO2C
O
3c
3.01
2.91
1.09
1.00
0.94
1.00
2.04
2.00
2.04
2.04
3.23
8.0 7.0 6.0 5.0 4.0 3.0 2.0 1.0 0
7.562 7.558 7.551 7.549 7.546 7.541 7.538 7.535 7.532 7.414 7.411 7.408 7.400 7.397 7.390 7.387 7.383 7.380 7.377 7.372 7.369 7.359 7.356 7.353 7.349 7.347 7.285 7.271 7.158 7.145 6.248 6.233 6.218 4.604 4.564 4.557 3.929 3.917 3.905 3.894 3.631 3.622 3.616 3.608
0.882 0.870 0.858
X : parts per Million : Proton

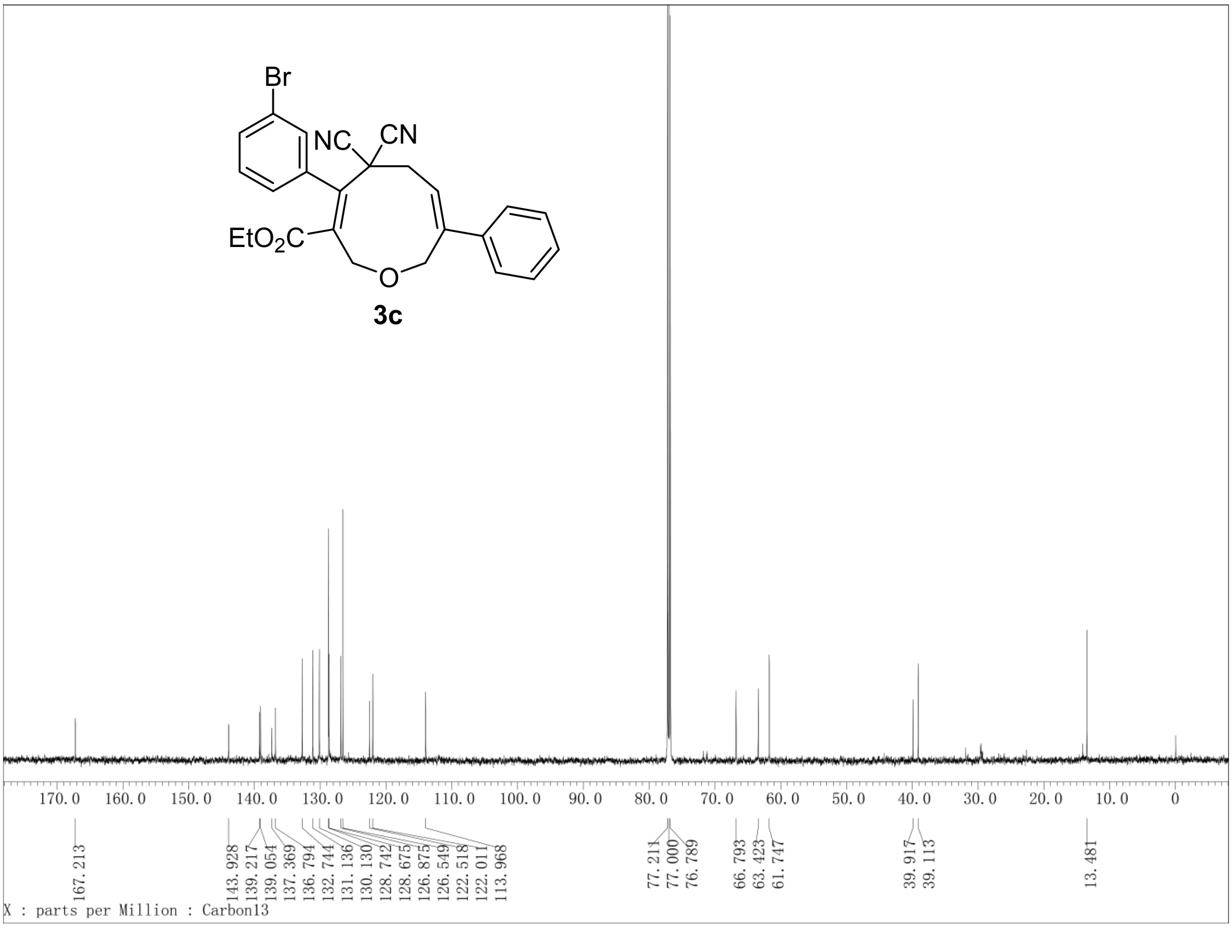
Br
NC
CN
EtO2C
O
3c
170.0 160.0 150.0 140.0 130.0 120.0 110.0 100.0 90.0 80.0 70.0 60.0 50.0 40.0 30.0 20.0 10.0 0
167.213
143.928 139.217 139.054 137.369 136.794 132.744 131.136 130.130 128.742 128.675 126.875 126.549 122.518 122.011 113.968
77.211 77.000 76.789
66.793 63.423 61.747
39.917 39.113
13.481
X : parts per Million : Carbon13

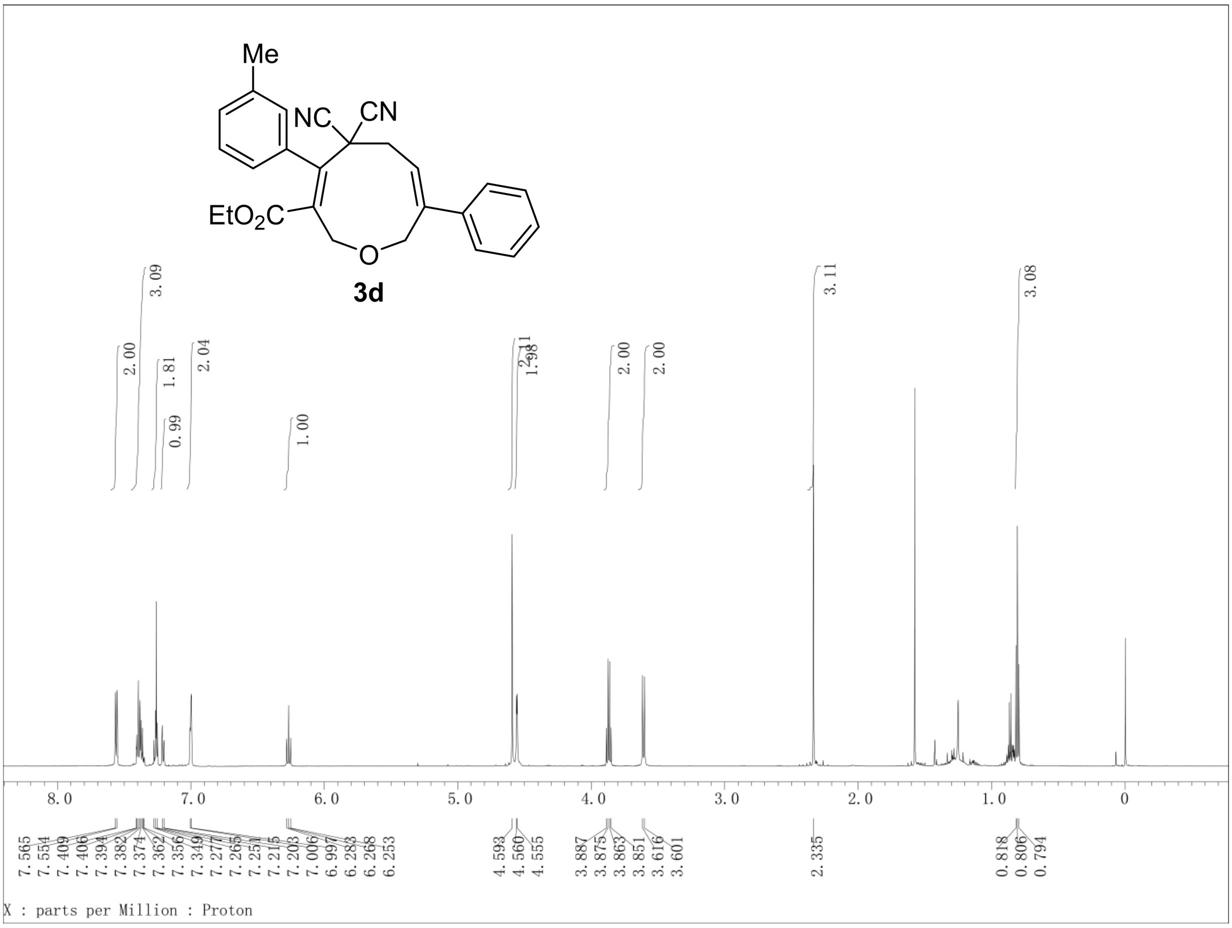
Me
NC
CN
EtO2C
O
3d
2.00
3.09
1.81
0.99
2.04
1.00
1.98
2.00
2.00
3.11
3.08
8.0 7.0 6.0 5.0 4.0 3.0 2.0 1.0 0
7.565
7.554
7.409
7.406
7.394
7.382
7.374
7.362
7.356
7.349
7.277
7.265
7.251
7.215
7.203
7.006
6.997
6.283
6.268
6.253
4.593
4.560
4.555
3.887
3.875
3.863
3.851
3.616
3.601
2.335
0.818
0.806
0.794
X : parts per Million : Proton

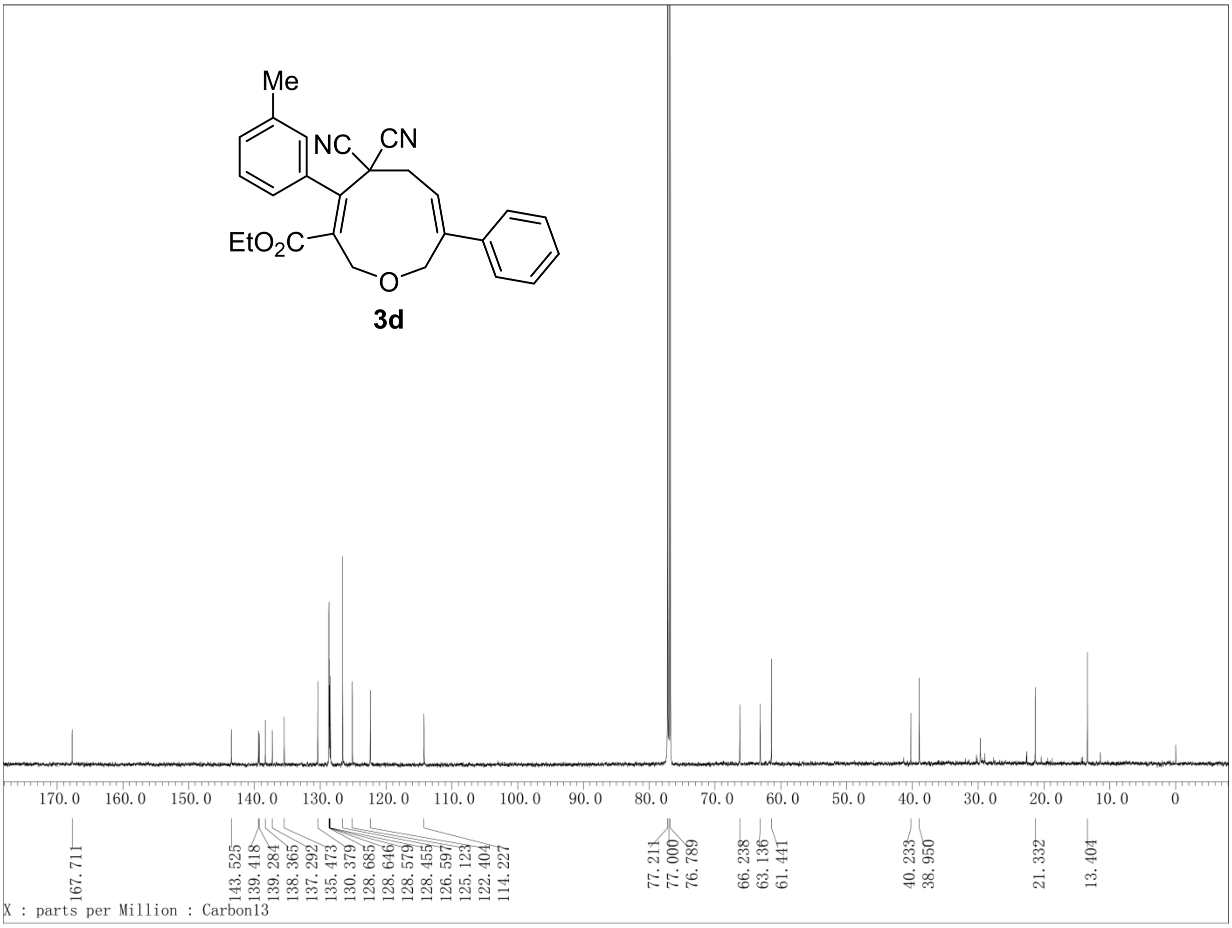
Me
NC
CN
EtO2C
O
3d
170.0 160.0 150.0 140.0 130.0 120.0 110.0 100.0 90.0 80.0 70.0 60.0 50.0 40.0 30.0 20.0 10.0 0
167.711
143.525
139.418
139.284
138.365
137.292
135.473
130.379
128.685
128.646
128.579
128.455
126.597
125.123
122.404
114.227
77.211
77.000
76.789
66.238
63.136
61.441
40.233
38.950
21.332
13.404
X : parts per Million : Carbon13

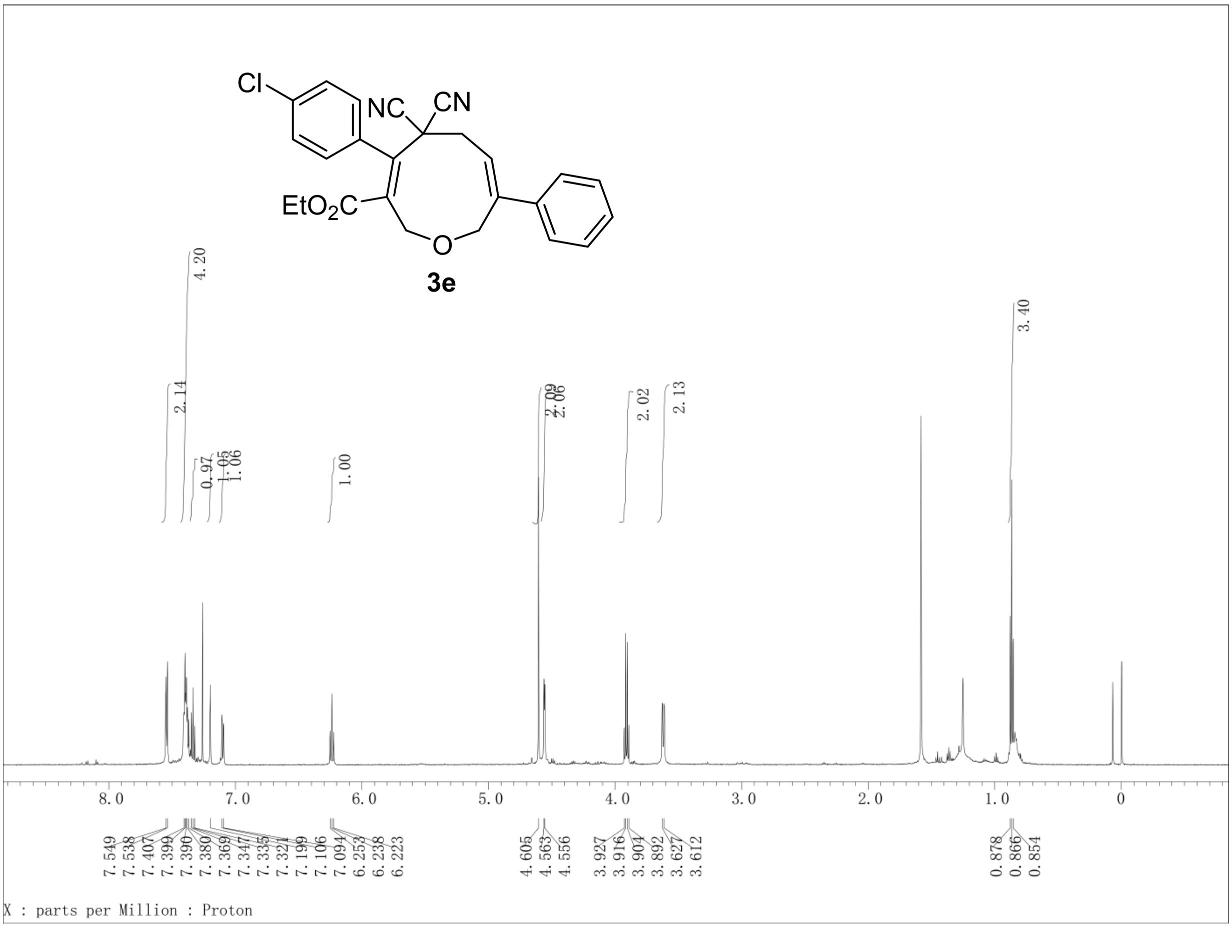
Cl
NC
CN
EtO2C
O
3e
4.20
2.14
0.97
1.05
1.06
1.00
2.09
2.06
2.02
2.13
3.40
8.0
7.0
6.0
5.0
4.0
3.0
2.0
1.0
0
7.549
7.538
7.407
7.399
7.390
7.380
7.369
7.347
7.335
7.321
7.199
7.106
7.094
6.253
6.238
6.223
4.605
4.563
4.556
3.927
3.916
3.904
3.892
3.627
3.612
0.878
0.866
0.854
X : parts per Million : Proton

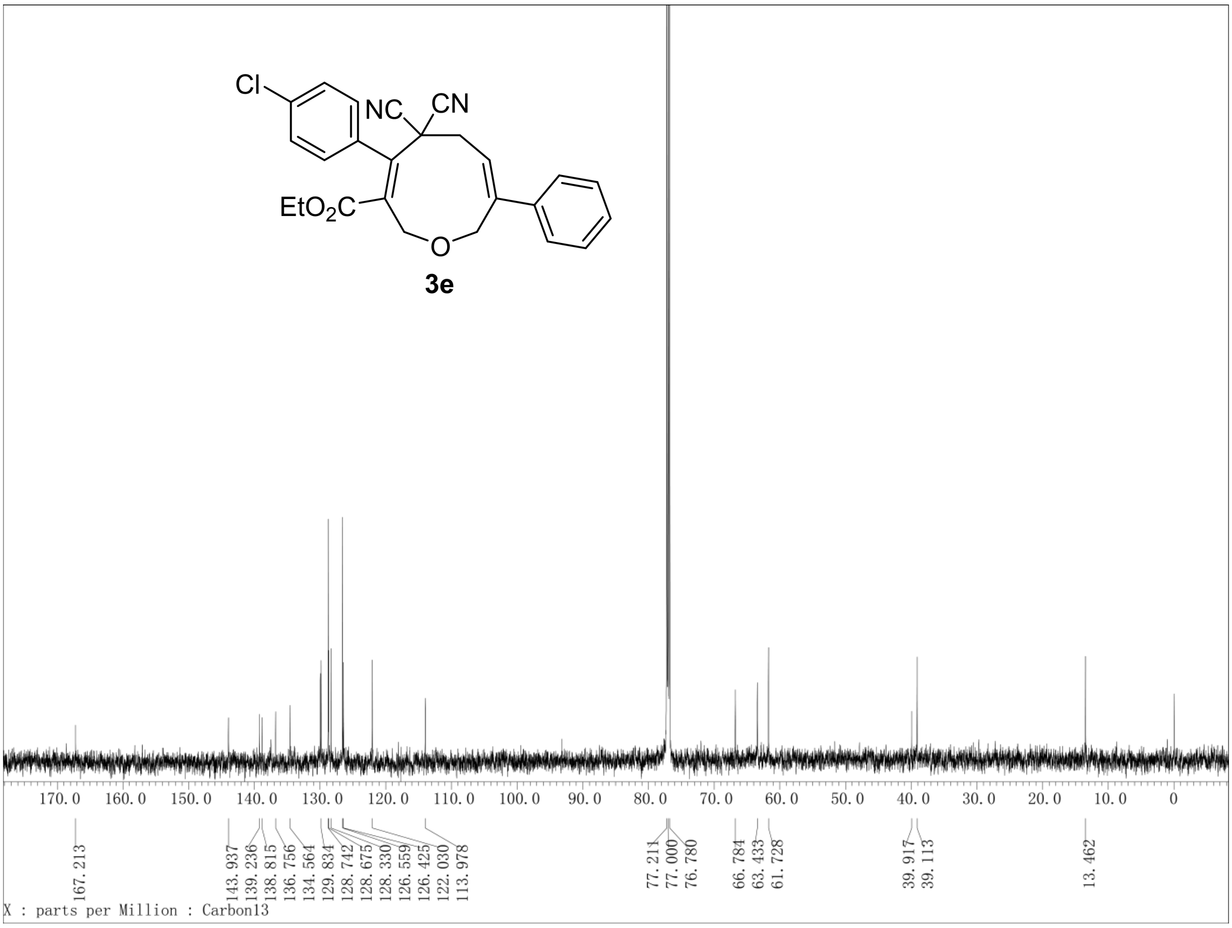
Cl
NC
CN
EtO2C
O
3e
170.0
160.0
150.0
140.0
130.0
120.0
110.0
100.0
90.0
80.0
70.0
60.0
50.0
40.0
30.0
20.0
10.0
0
167.213
143.937
139.236
138.815
136.756
134.564
129.834
128.742
128.675
128.330
126.559
126.425
122.030
113.978
77.211
77.000
76.780
66.784
63.433
61.728
39.917
39.113
13.462
X : parts per Million : Carbon13

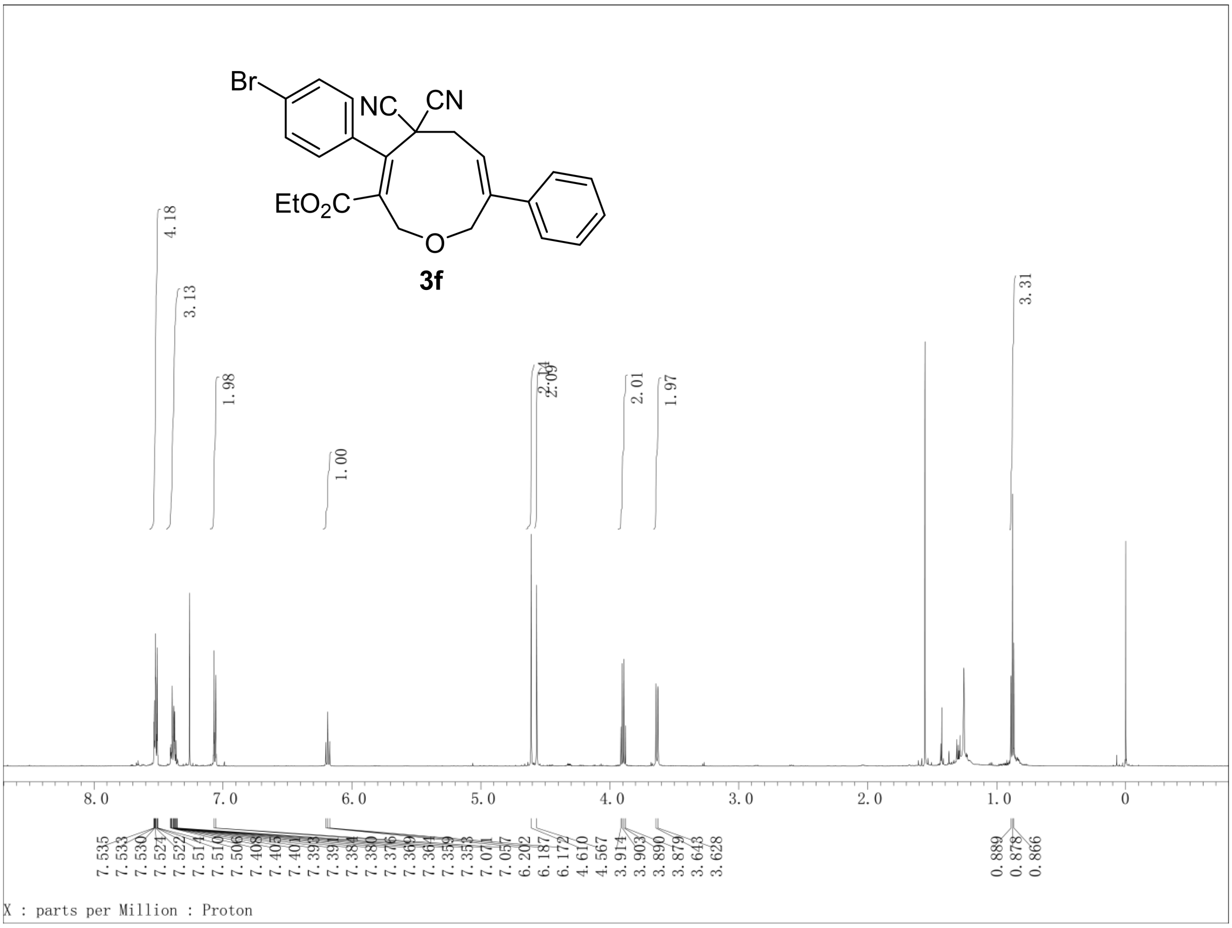

Br
NC
CN
EtO2C
O
3f
4.18
3.13
1.98
1.00
2.14
2.09
2.01
1.97
3.31
8.0 7.0 6.0 5.0 4.0 3.0 2.0 1.0 0
7.535 7.533 7.530 7.524 7.522 7.514 7.510 7.506 7.408 7.405 7.401 7.393 7.391 7.384 7.380 7.376 7.369 7.364 7.359 7.353 7.071 7.057 6.202 6.187 6.172 4.610 4.567 3.914 3.903 3.890 3.879 3.643 3.628
0.889 0.878 0.866
X : parts per Million : Proton

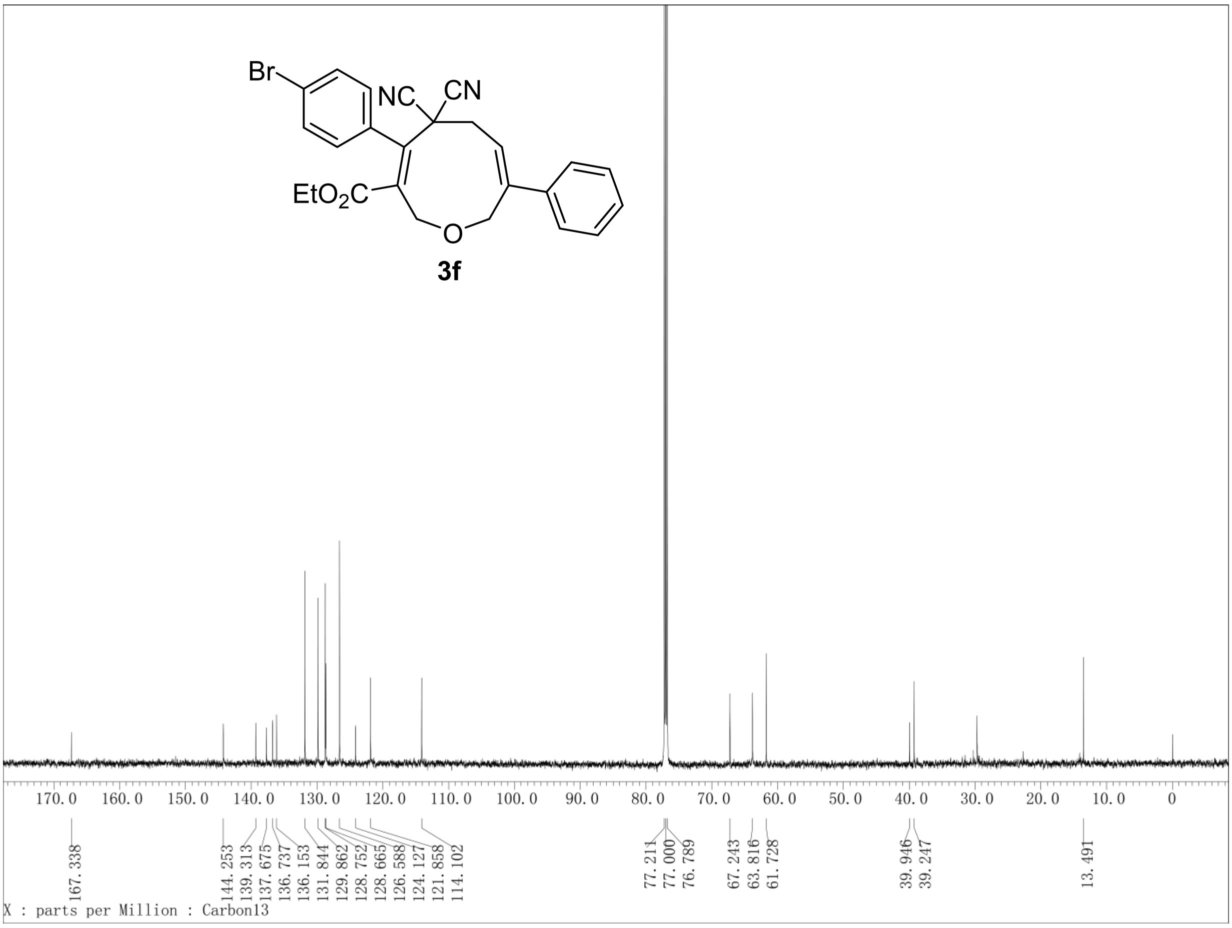

Br
NC
CN
EtO2C
O
3f
170.0 160.0 150.0 140.0 130.0 120.0 110.0 100.0 90.0 80.0 70.0 60.0 50.0 40.0 30.0 20.0 10.0 0
167.338
144.253 139.313 137.675 136.737 136.153 131.844 129.862 128.752 128.665 126.588 124.127 121.858 114.102
77.211 77.000 76.789
67.243 63.816 61.728
39.946 39.247
13.491
X : parts per Million : Carbon13

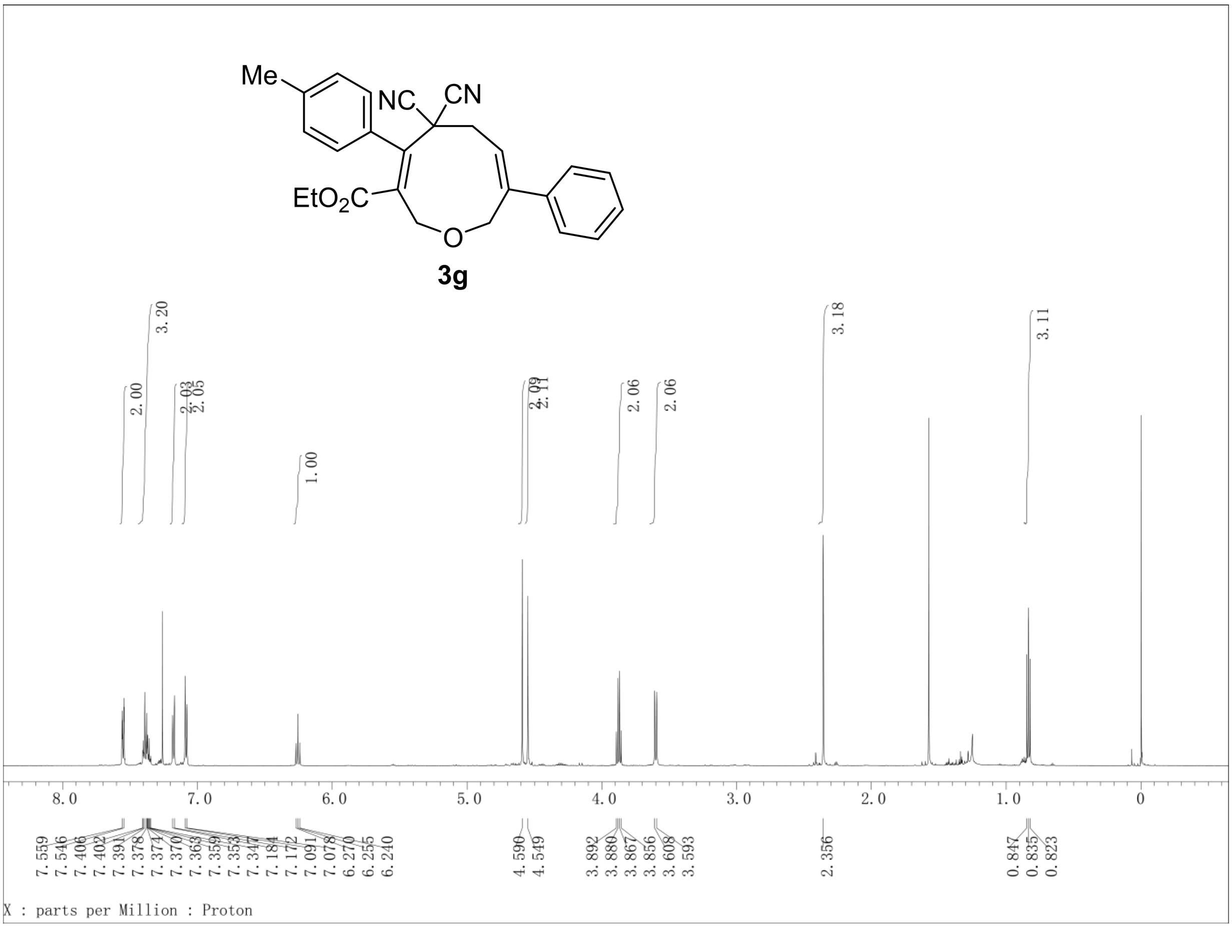
Me
NC
CN
EtO2C
O
3g
2.00
3.20
2.03
2.05
1.00
2.09
2.11
2.06
2.06
3.18
3.11
7.559 7.546 7.406 7.402 7.391 7.378 7.374 7.370 7.363 7.359 7.353 7.347 7.184 7.172 7.091 7.078 6.270 6.255 6.240
4.590 4.549
3.892 3.880 3.867 3.856 3.608 3.593
2.356
0.847 0.835 0.823
X : parts per Million : Proton

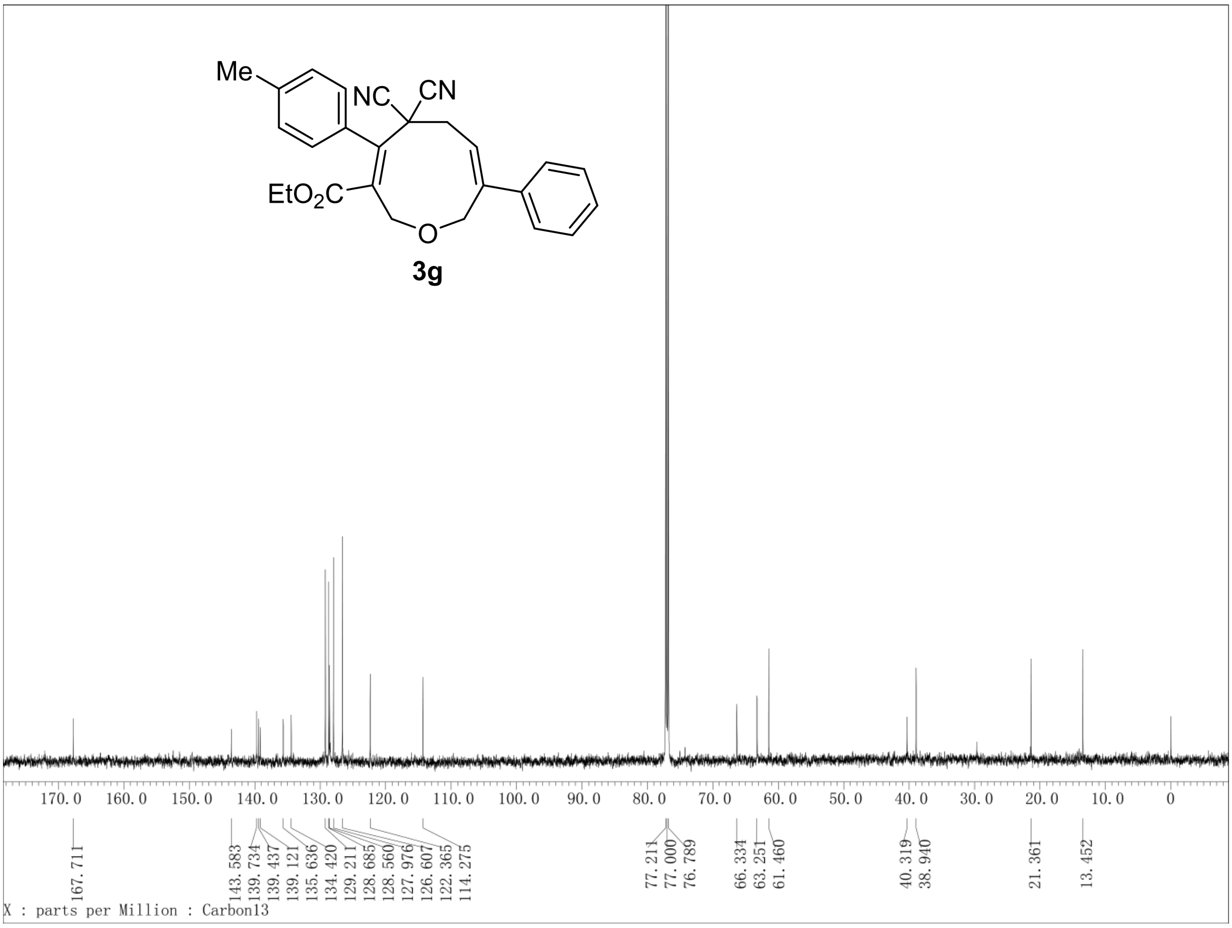
Me
NC
CN
EtO2C
O
3g
167.711
143.583 139.734 139.437 139.121 135.636 134.420 129.211 128.685 128.560 127.976 126.607 122.365 114.275
77.211 77.000 76.789
66.334 63.251 61.460
40.319 38.940
21.361
13.452
X : parts per Million : Carbon13

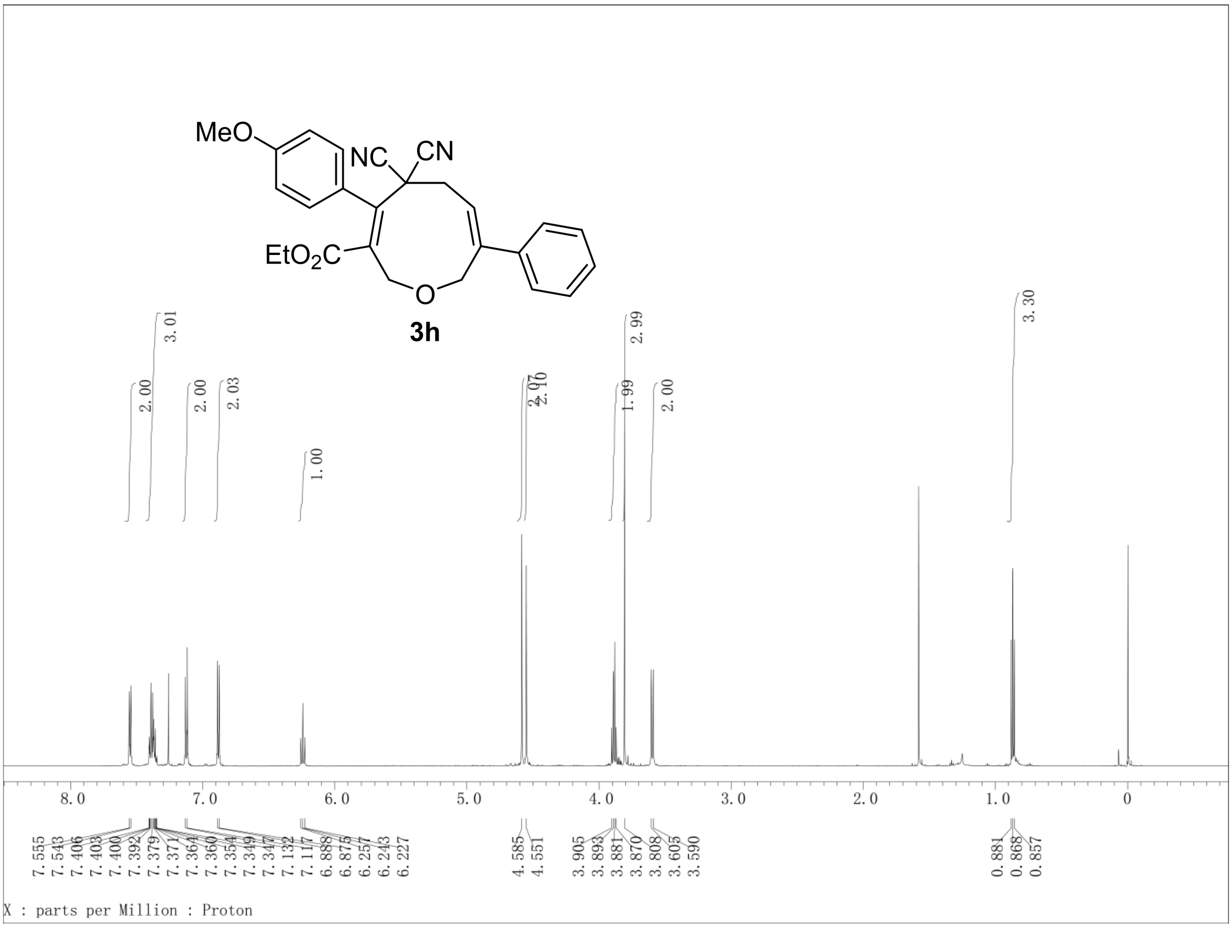
MeO
NC
CN
EtO2C
O
3h
2.00
3.01
2.00
2.03
1.00
2.07
2.10
1.99
2.99
2.00
3.30
8.0
7.0
6.0
5.0
4.0
3.0
2.0
1.0
0
7.555
7.543
7.406
7.403
7.400
7.392
7.379
7.371
7.364
7.360
7.354
7.349
7.347
7.132
7.117
6.888
6.875
6.257
6.243
6.227
4.585
4.551
3.905
3.893
3.881
3.870
3.808
3.605
3.590
0.881
0.868
0.857
X : parts per Million : Proton

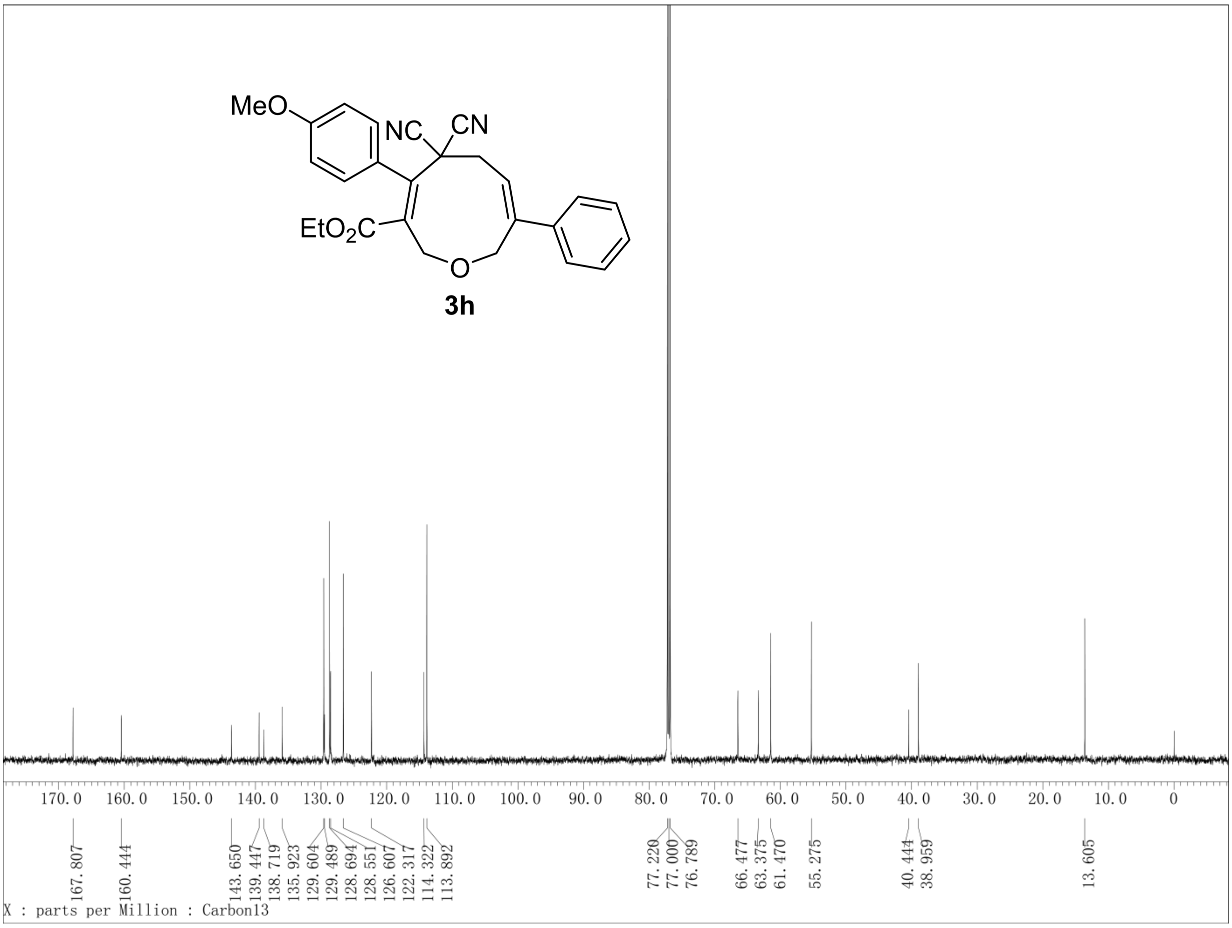
MeO
NC
CN
EtO2C
O
3h
170.0
160.0
150.0
140.0
130.0
120.0
110.0
100.0
90.0
80.0
70.0
60.0
50.0
40.0
30.0
20.0
10.0
0
167.807
160.444
143.650
139.447
138.719
135.923
129.604
129.489
128.694
128.551
126.607
122.317
114.322
113.892
77.220
77.000
76.789
66.477
63.375
61.470
55.275
40.444
38.959
13.605
X : parts per Million : Carbon13

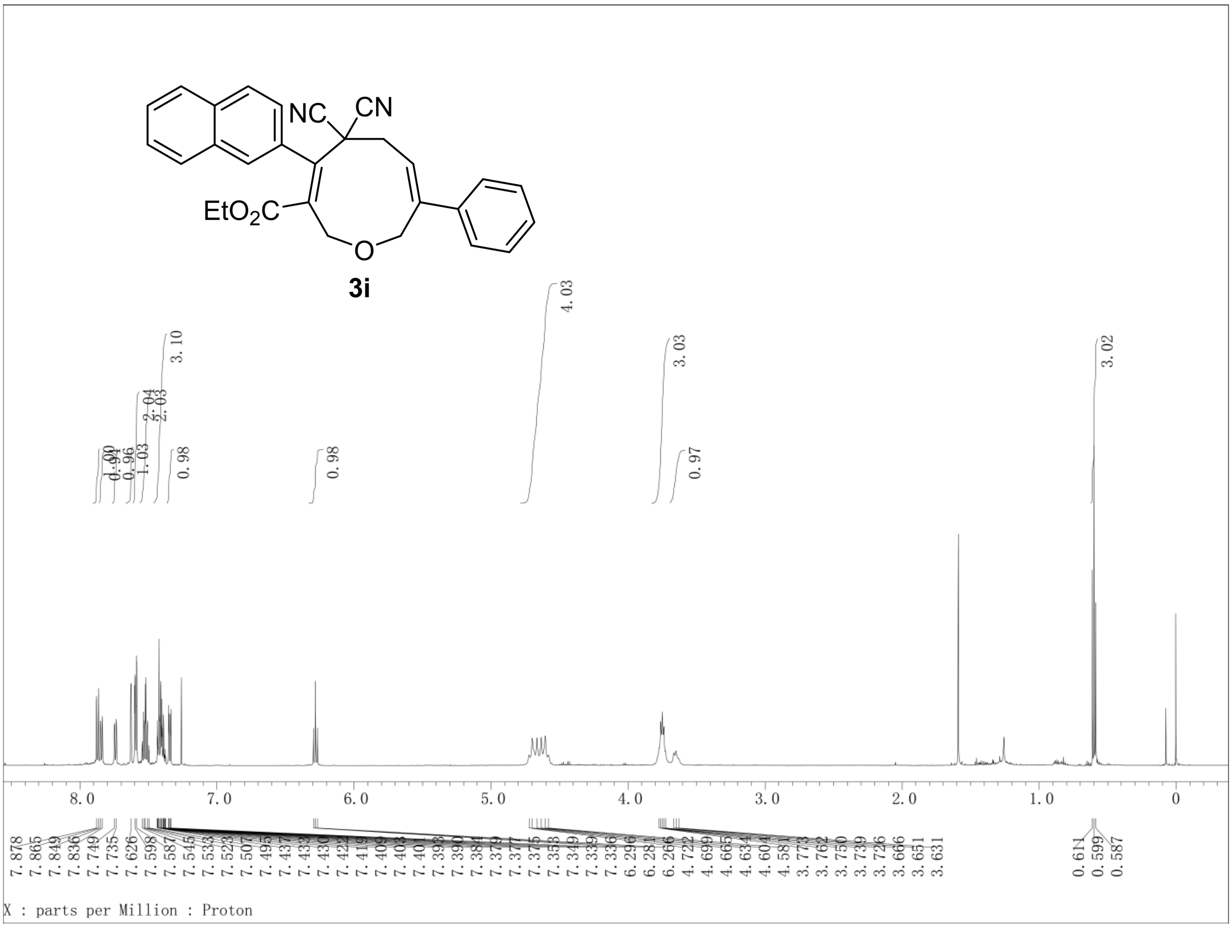
NC
CN
EtO2C
O
3i
1.00
0.94
0.96
1.03
2.04
2.03
3.10
0.98
0.98
4.03
3.03
0.97
3.02
8.0 7.0 6.0 5.0 4.0 3.0 2.0 1.0 0
7.878 7.865 7.849 7.836 7.749 7.735 7.626 7.598 7.587 7.545 7.533 7.523 7.507 7.495 7.437 7.433 7.430 7.422 7.419 7.409 7.403 7.401 7.398 7.390 7.384 7.379 7.377 7.375 7.353 7.349 7.339 7.336 6.296 6.281 6.266 4.722 4.699 4.665 4.634 4.604 4.581 3.773 3.762 3.750 3.739 3.726 3.666 3.651 3.631
0.611 0.599 0.587
X : parts per Million : Proton

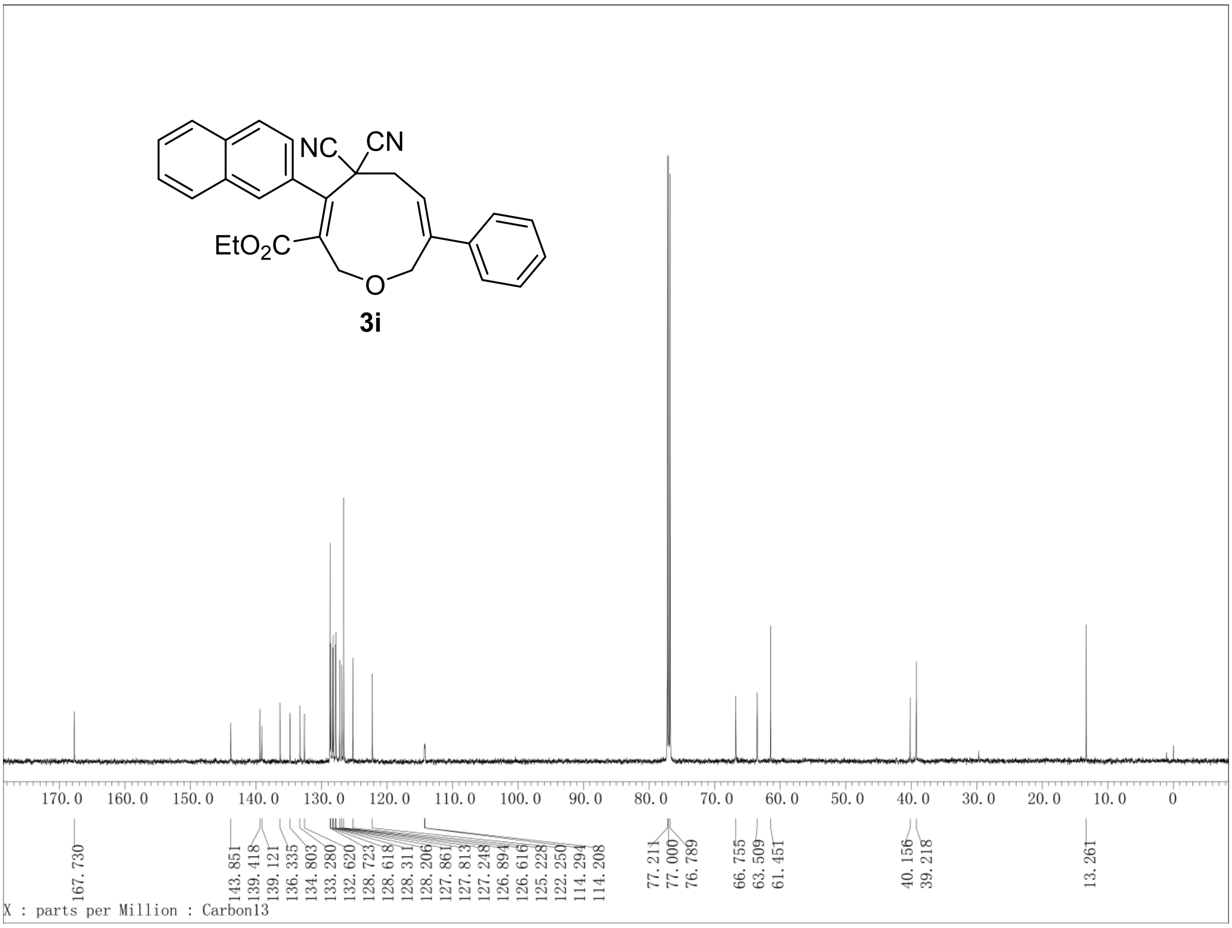
NC
CN
EtO2C
O
3i
170.0 160.0 150.0 140.0 130.0 120.0 110.0 100.0 90.0 80.0 70.0 60.0 50.0 40.0 30.0 20.0 10.0 0
167.730
143.851 139.418 139.121 136.335 134.803 133.280 132.620 128.723 128.618 128.311 128.206 127.861 127.813 127.248 126.894 126.616 125.228 122.250 114.294 114.208
77.211 77.000 76.789
66.755 63.509 61.451
40.156 39.218
13.261
X : parts per Million : Carbon13

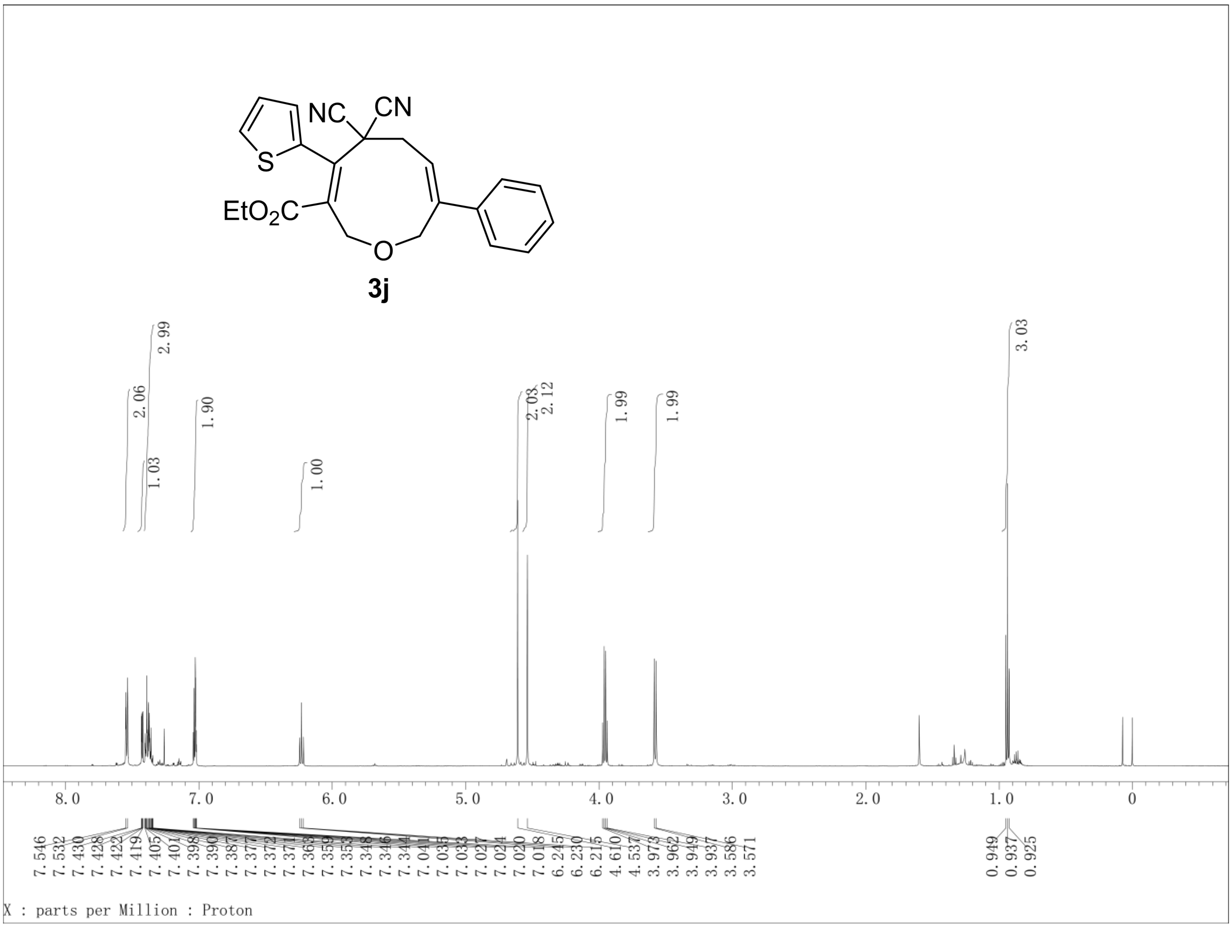
NC
CN
S
EtO_2C
O
3j
2.06
2.99
1.03
1.90
1.00
2.03
2.12
1.99
1.99
3.03
8.0
7.0
6.0
5.0
4.0
3.0
2.0
1.0
0
7.546
7.532
7.430
7.428
7.422
7.419
7.405
7.401
7.398
7.390
7.387
7.377
7.372
7.371
7.363
7.359
7.353
7.348
7.346
7.344
7.041
7.035
7.033
7.027
7.024
7.020
7.018
6.245
6.230
6.215
4.610
4.537
3.973
3.962
3.949
3.937
3.586
3.571
0.949
0.937
0.925
X : parts per Million : Proton

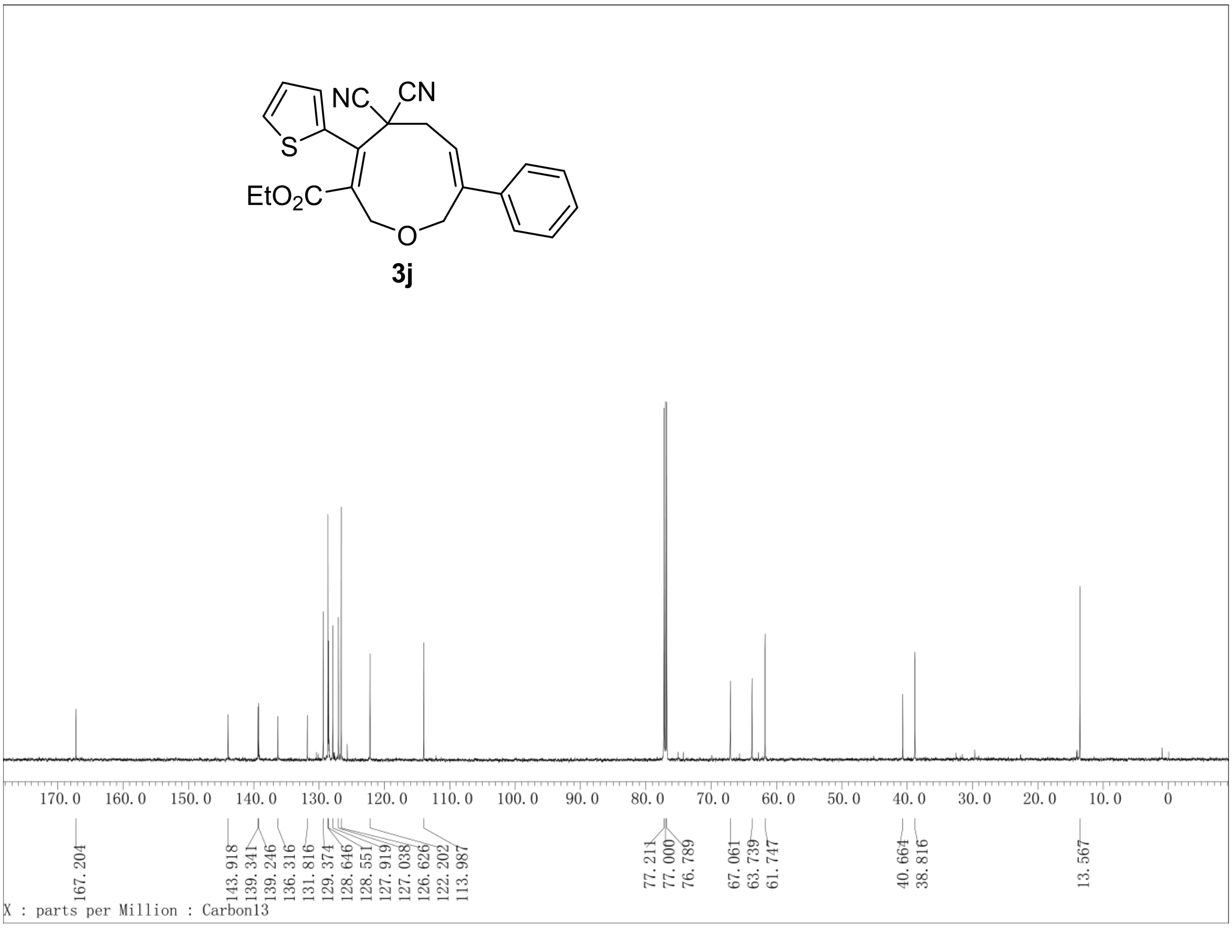
NC
CN
S
EtO_2C
O
3j
170.0
160.0
150.0
140.0
130.0
120.0
110.0
100.0
90.0
80.0
70.0
60.0
50.0
40.0
30.0
20.0
10.0
0
167.204
143.918
139.341
139.246
136.316
131.816
129.374
128.646
128.551
127.919
127.038
126.626
122.202
113.987
77.211
77.000
76.789
67.061
63.739
61.747
40.664
38.816
13.567
X : parts per Million : Carbon13

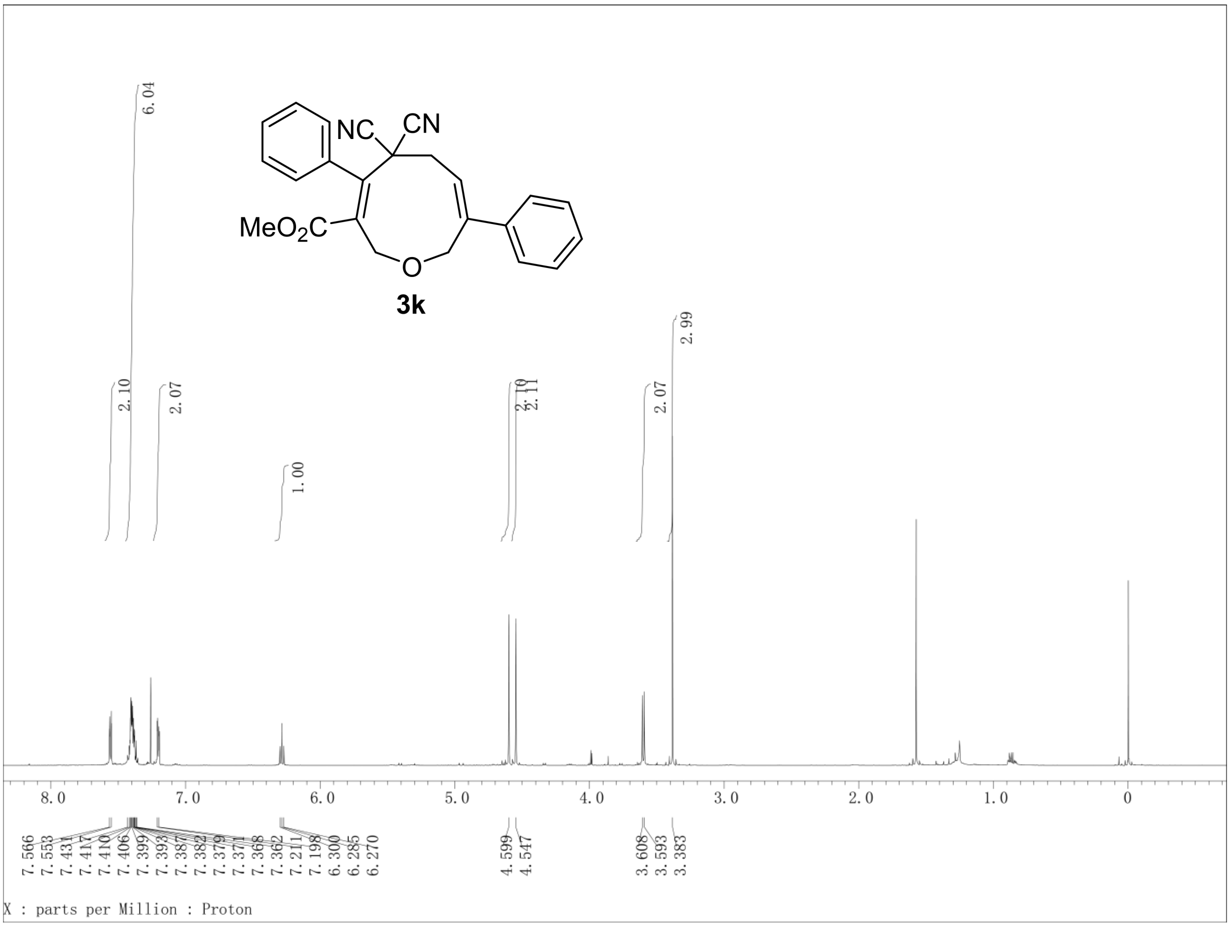
NC
CN
MeO2C
O
3k
6.04
2.10
2.07
1.00
2.10
2.11
2.07
2.99
8.0 7.0 6.0 5.0 4.0 3.0 2.0 1.0 0
7.566 7.553 7.431 7.417 7.410 7.406 7.399 7.393 7.387 7.382 7.379 7.371 7.368 7.362 7.211 7.198 6.300 6.285 6.270
4.599 4.547
3.608 3.593 3.383
X : parts per Million : Proton

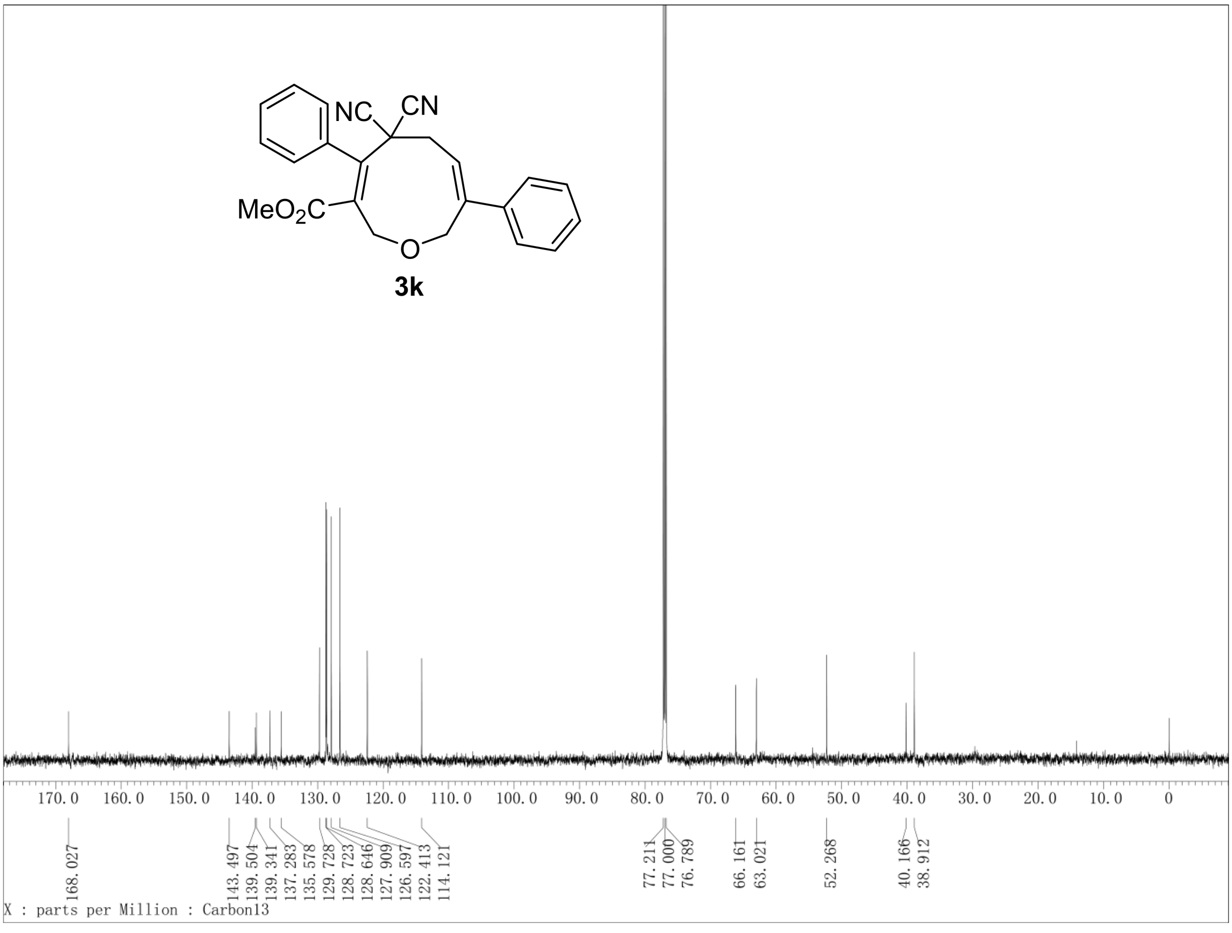
NC
CN
MeO2C
O
3k
170.0 160.0 150.0 140.0 130.0 120.0 110.0 100.0 90.0 80.0 70.0 60.0 50.0 40.0 30.0 20.0 10.0 0
168.027
143.497 139.504 139.341 137.283 135.578 129.728 128.723 128.646 127.909 126.597 122.413 114.121
77.211 77.000 76.789
66.161 63.021
52.268
40.166 38.912
X : parts per Million : Carbon13

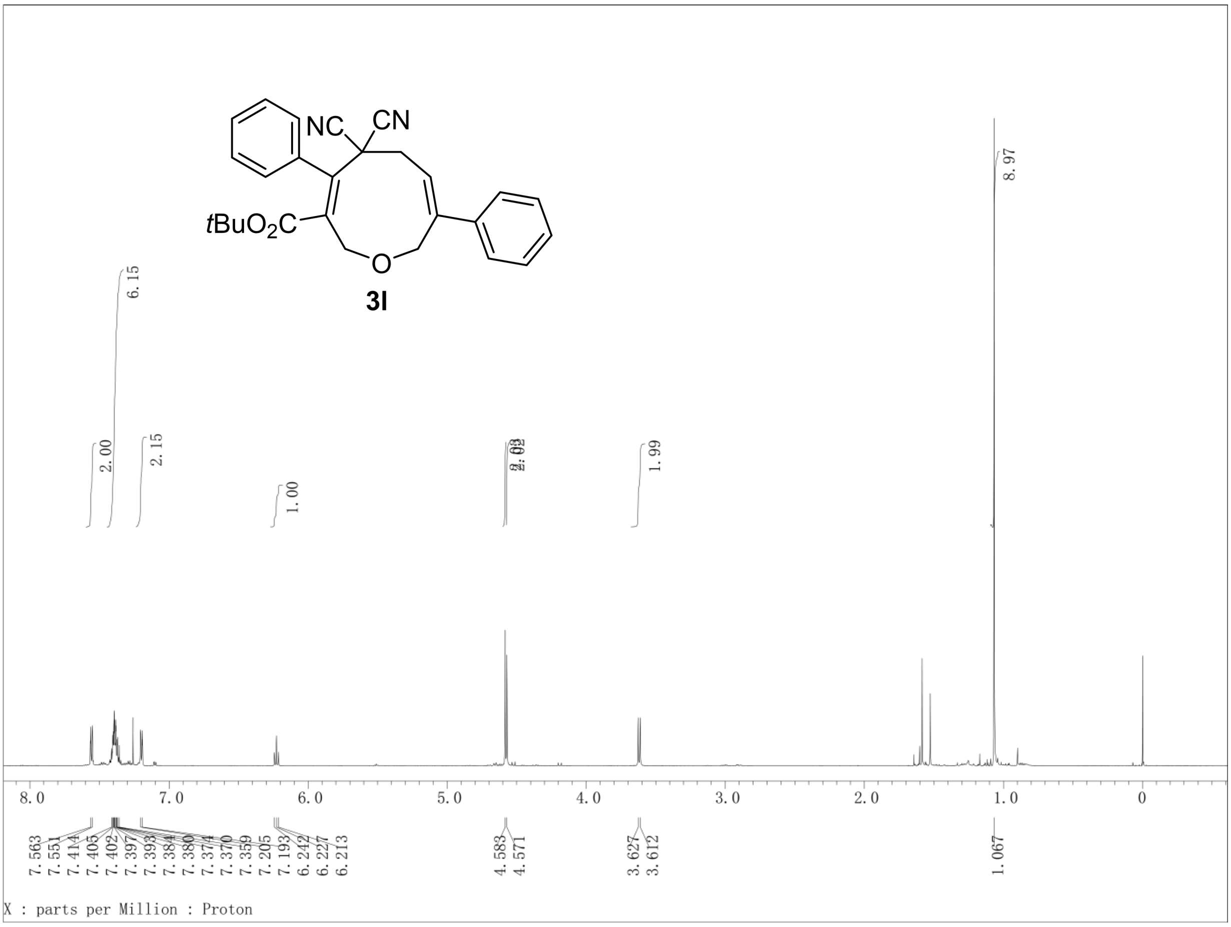
NC CN
tBuO2C
O
3l
8.97
6.15
2.00
2.15
1.00
2.03 2.02
1.99
8.0 7.0 6.0 5.0 4.0 3.0 2.0 1.0 0
7.563 7.551 7.414 7.405 7.402 7.397 7.393 7.384 7.380 7.374 7.370 7.359 7.205 7.193 6.242 6.227 6.213
4.583 4.571
3.627 3.612
1.067
X : parts per Million : Proton

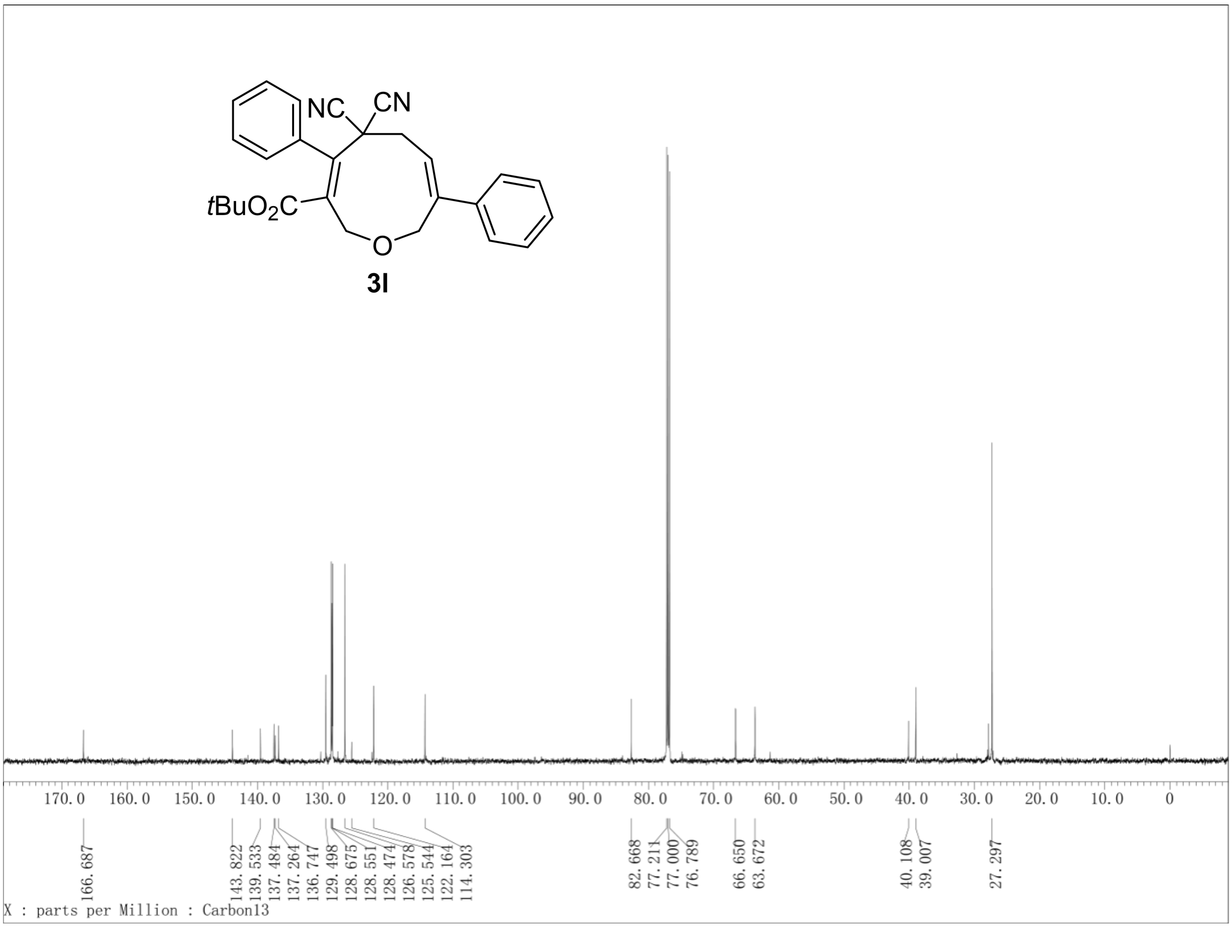
NC CN
tBuO2C
O
3l
170.0 160.0 150.0 140.0 130.0 120.0 110.0 100.0 90.0 80.0 70.0 60.0 50.0 40.0 30.0 20.0 10.0 0
166.687
143.822 139.533 137.484 137.264 136.747 129.498 128.675 128.551 128.474 126.578 125.544 122.164 114.303
82.668 77.211 77.000 76.789
66.650 63.672
40.108 39.007
27.297
X : parts per Million : Carbon13

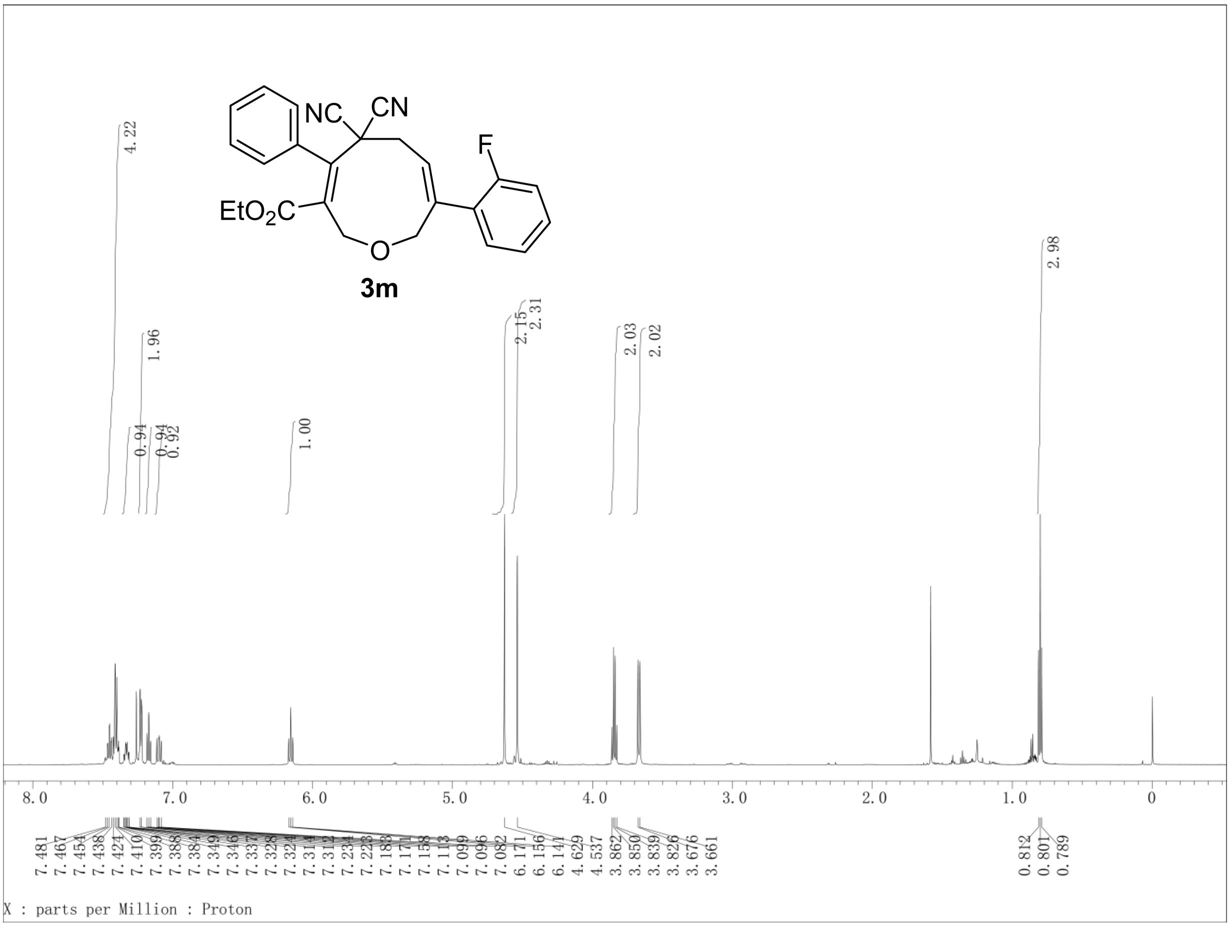
NC CN
F
EtO2C
O
3m
4.22
1.96
0.94
0.94
0.92
1.00
2.15
2.31
2.03
2.02
2.98
8.0 7.0 6.0 5.0 4.0 3.0 2.0 1.0 0
7.481 7.467 7.454 7.438 7.424 7.410 7.399 7.388 7.384 7.349 7.346 7.337 7.328 7.234 7.223 7.183 7.153 7.113 7.099 7.096 7.082 6.171 6.156 6.141 4.629 4.537 3.862 3.850 3.839 3.826 3.676 3.661
0.812 0.801 0.789
X : parts per Million : Proton

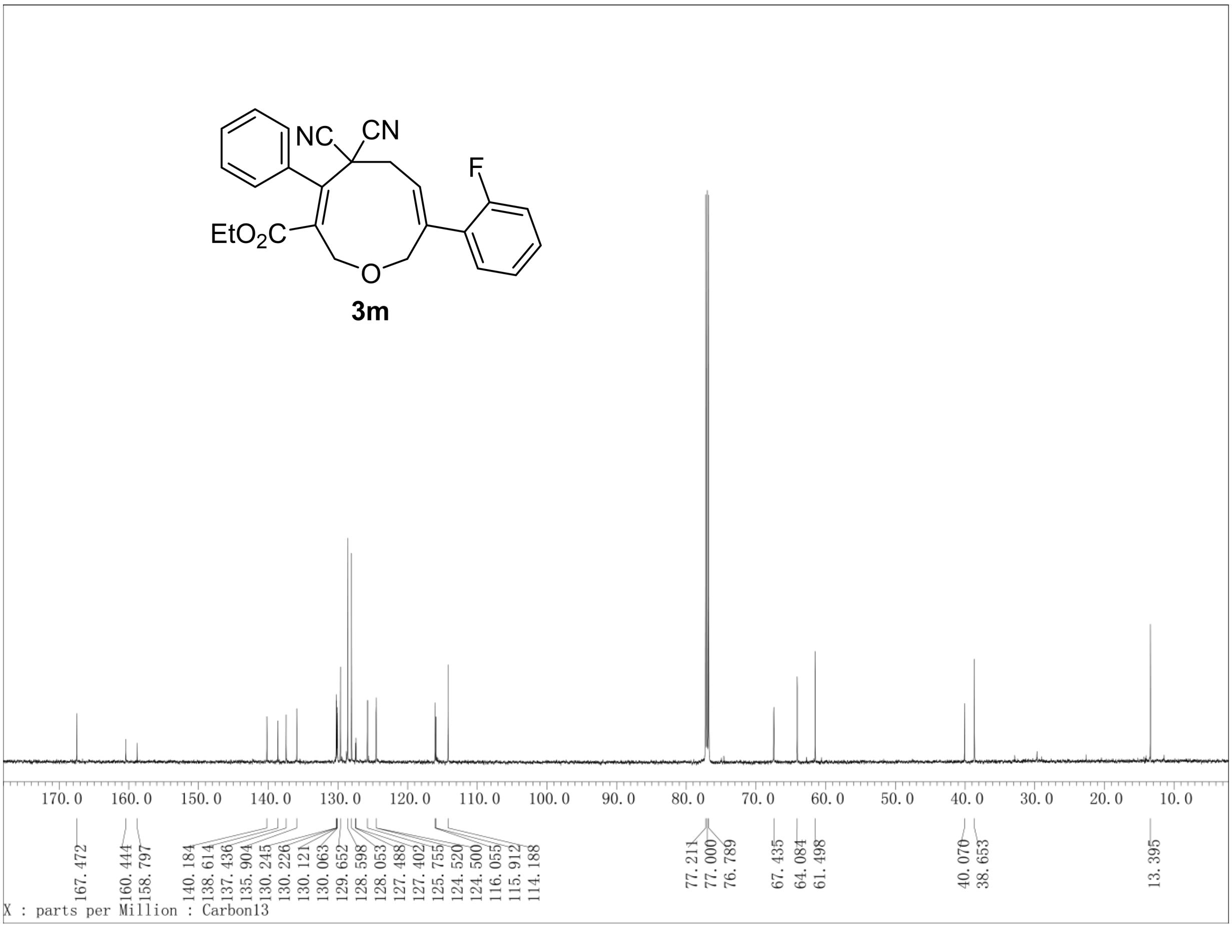
NC CN
F
EtO2C
O
3m
170.0 160.0 150.0 140.0 130.0 120.0 110.0 100.0 90.0 80.0 70.0 60.0 50.0 40.0 30.0 20.0 10.0
167.472
160.444 158.797
140.184 138.614 137.436 135.904 130.245 130.226 130.121 130.063 129.652 128.598 128.053 127.488 127.402 125.755 124.520 124.500 116.055 115.912 114.188
77.211 77.000 76.789
67.435 64.084 61.498
40.070 38.653
13.395
X : parts per Million : Carbon13

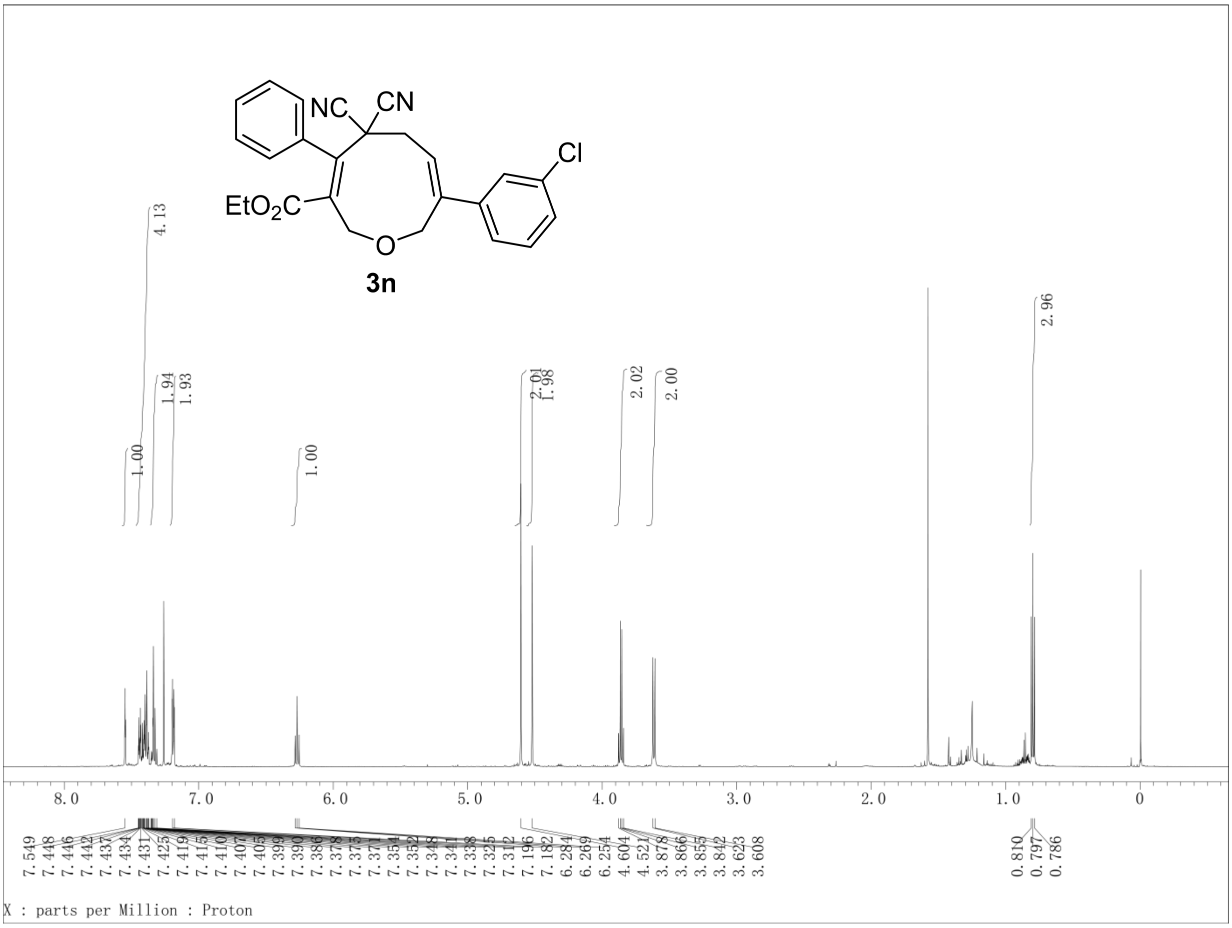
NC
CN
Cl
EtO2C
O
3n
4.13
1.00
1.94
1.93
1.00
2.01
1.98
2.02
2.00
2.96
8.0 7.0 6.0 5.0 4.0 3.0 2.0 1.0 0
7.549 7.448 7.446 7.442 7.437 7.434 7.431 7.425 7.419 7.415 7.410 7.407 7.405 7.399 7.390 7.386 7.378 7.375 7.371 7.354 7.352 7.348 7.341 7.338 7.325 7.312 7.196 7.182 6.284 6.269 6.254 4.604 4.521 3.878 3.866 3.855 3.842 3.623 3.608
0.810 0.797 0.786
X : parts per Million : Proton

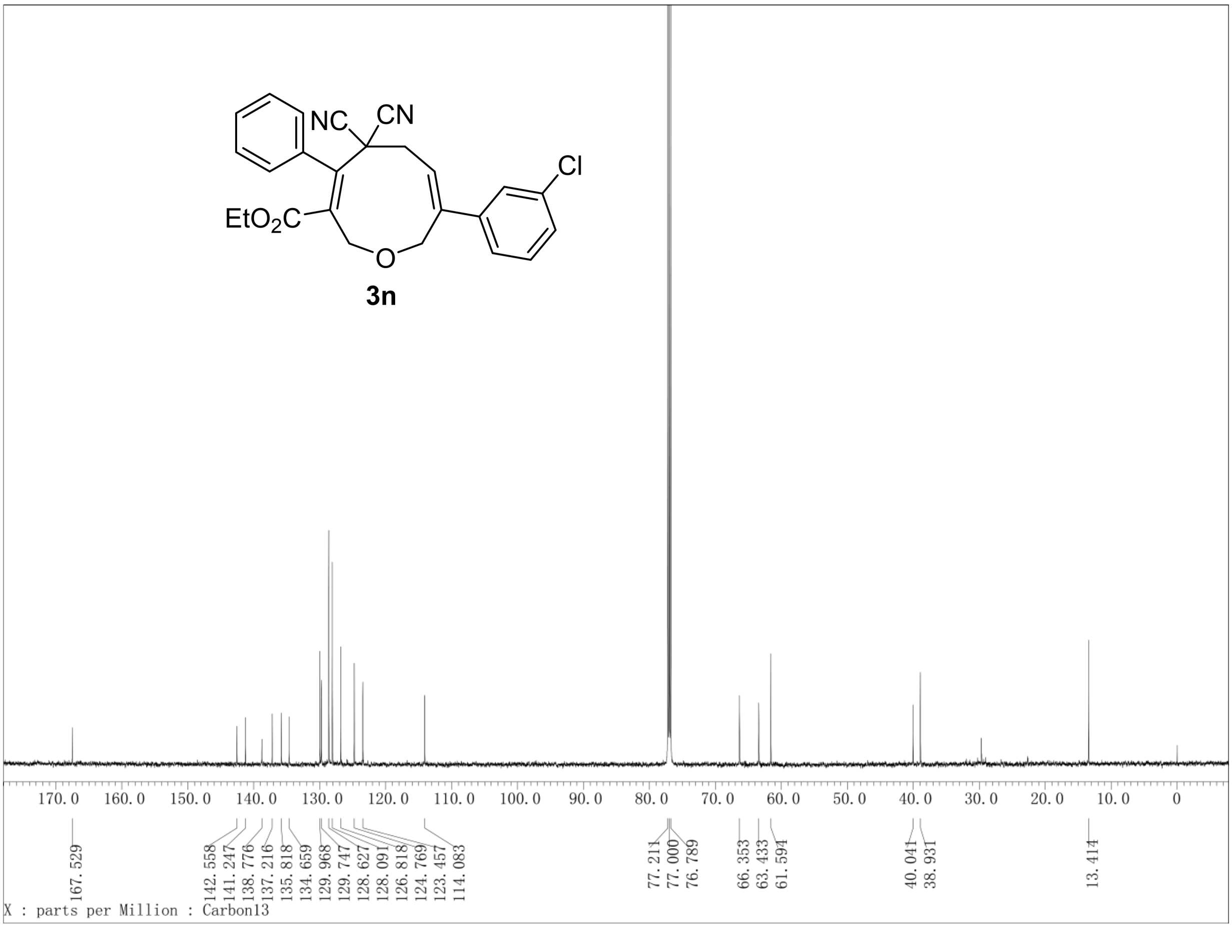
NC
CN
Cl
EtO2C
O
3n
170.0 160.0 150.0 140.0 130.0 120.0 110.0 100.0 90.0 80.0 70.0 60.0 50.0 40.0 30.0 20.0 10.0 0
167.529
142.558 141.247 138.776 137.216 135.818 134.659 129.968 129.747 128.627 128.091 126.818 124.769 123.457 114.083
77.211 77.000 76.789
66.353 63.433 61.594
40.041 38.931
13.414
X : parts per Million : Carbon13

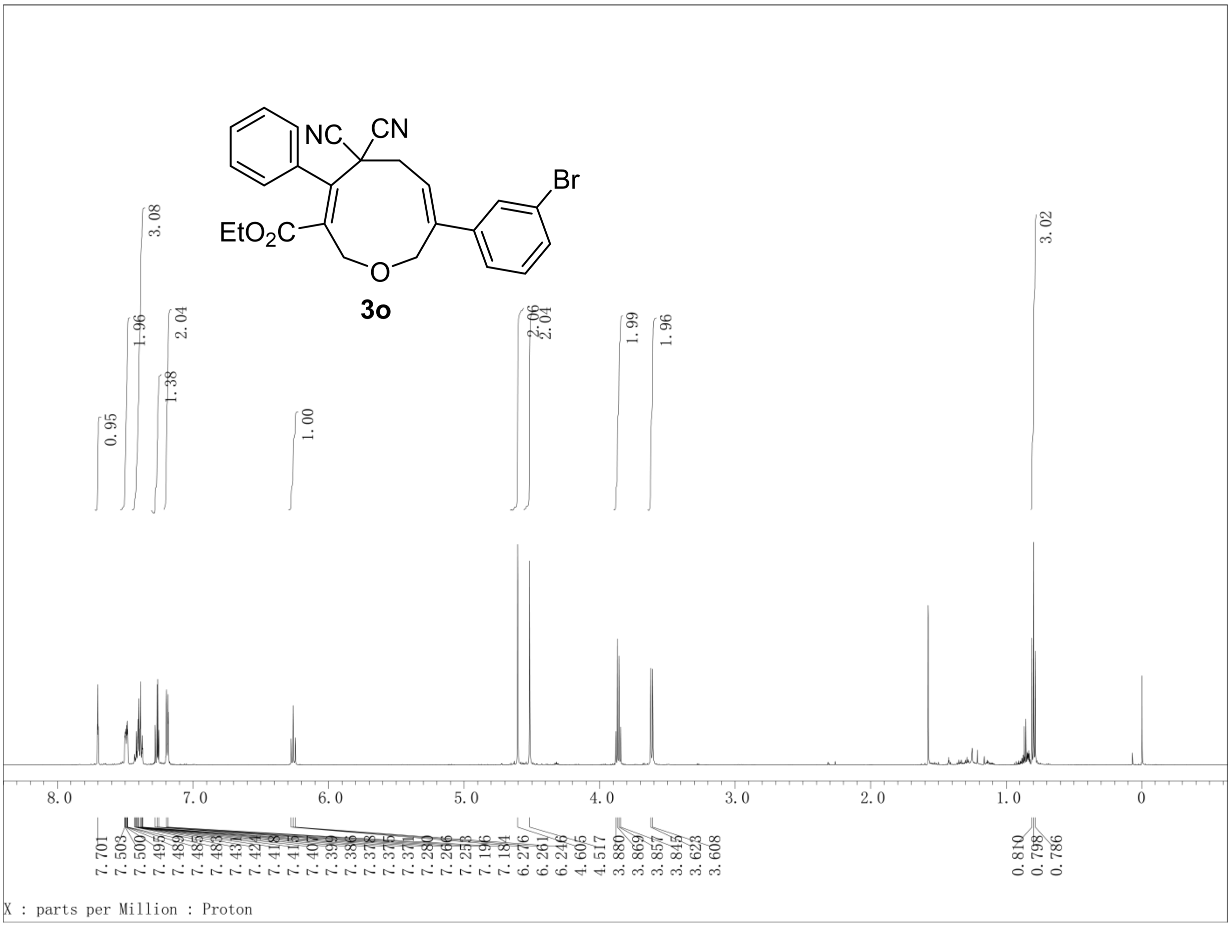
NC
CN
Br
EtO2C
O
3o
0.95
1.96
3.08
1.38
2.04
1.00
2.06
2.04
1.99
1.96
3.02
8.0 7.0 6.0 5.0 4.0 3.0 2.0 1.0 0
7.701 7.503 7.500 7.495 7.489 7.485 7.483 7.431 7.424 7.418 7.415 7.407 7.399 7.386 7.378 7.375 7.371 7.280 7.266 7.253 7.196 7.184 6.276 6.261 6.246 4.605 4.517 3.880 3.869 3.857 3.845 3.623 3.608
0.810 0.798 0.786
X : parts per Million : Proton

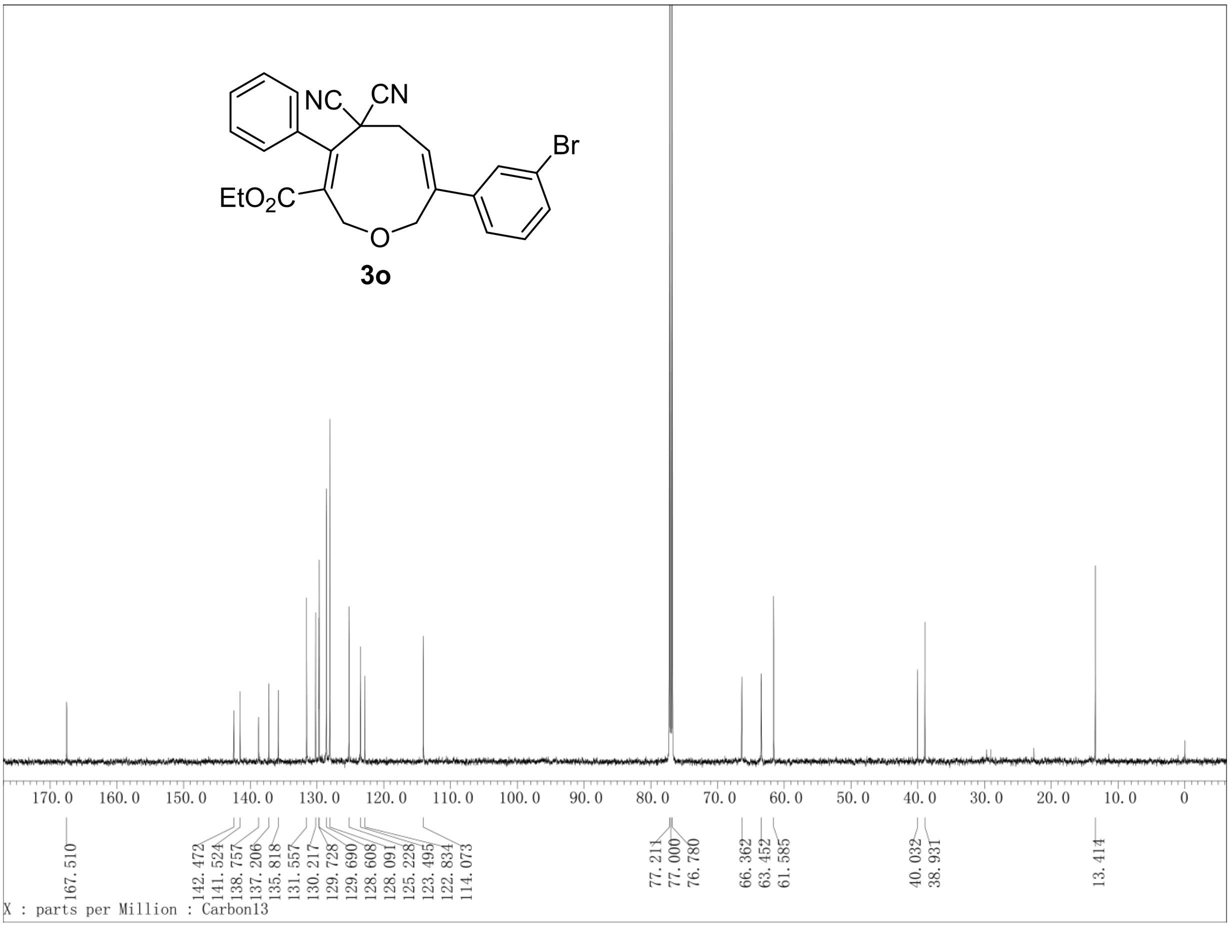
NC
CN
Br
EtO2C
O
3o
170.0 160.0 150.0 140.0 130.0 120.0 110.0 100.0 90.0 80.0 70.0 60.0 50.0 40.0 30.0 20.0 10.0 0
167.510
142.472 141.524 138.757 137.206 135.818 131.557 130.217 129.728 129.690 128.608 128.091 125.228 123.495 122.834 114.073
77.211 77.000 76.780
66.362 63.452 61.585
40.032 38.931
13.414
X : parts per Million : Carbon13

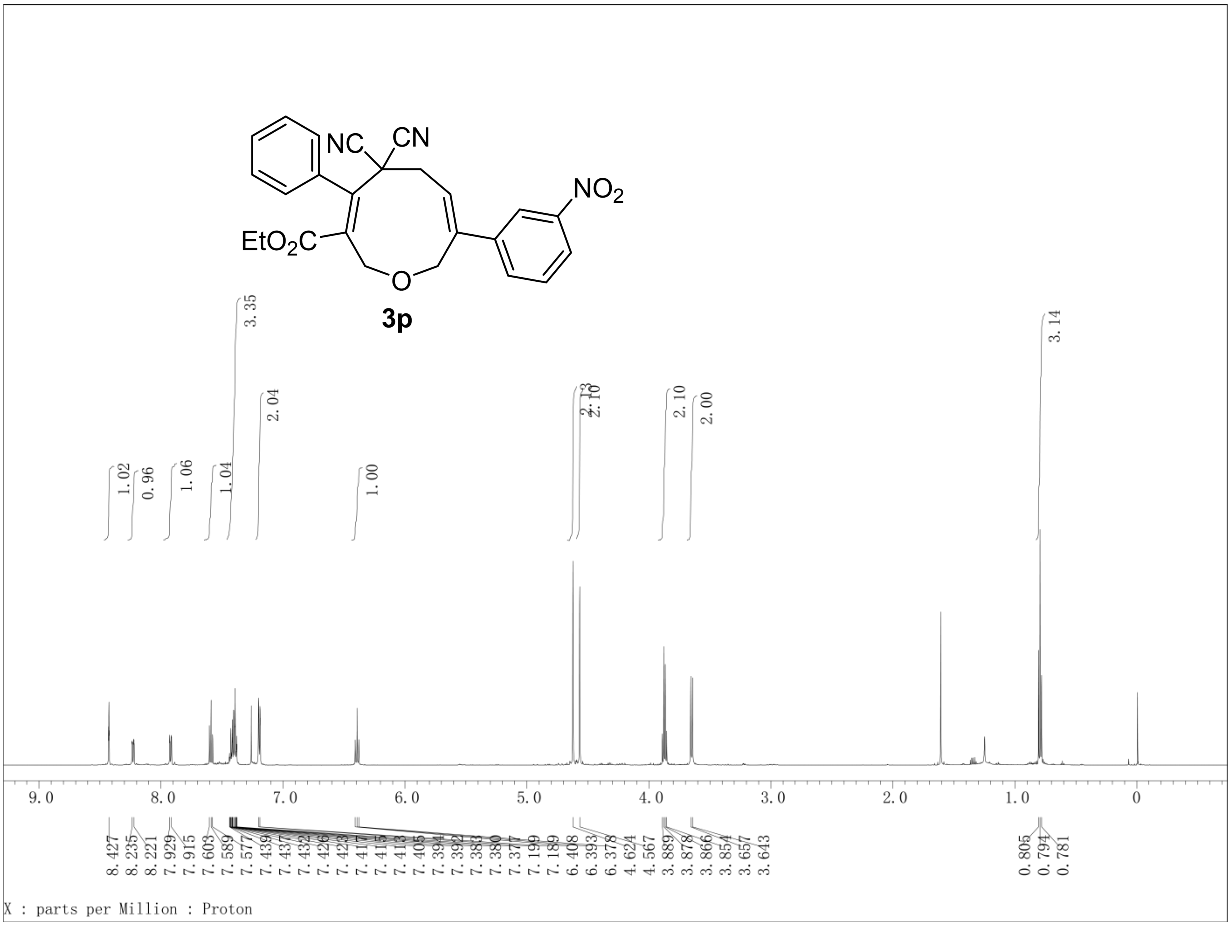
NC CN
NO2
EtO2C
O
3p
1.02 0.96 1.06 1.04 3.35 2.04 1.00 2.13 2.10 2.10 2.00 3.14
9.0 8.0 7.0 6.0 5.0 4.0 3.0 2.0 1.0 0
8.427 8.235 8.221 7.929 7.915 7.603 7.589 7.577 7.439 7.437 7.432 7.426 7.423 7.417 7.415 7.413 7.405 7.394 7.392 7.383 7.380 7.377 7.199 7.189 6.408 6.393 6.378 4.624 4.567 3.889 3.878 3.866 3.854 3.657 3.643
0.805 0.794 0.781
X : parts per Million : Proton

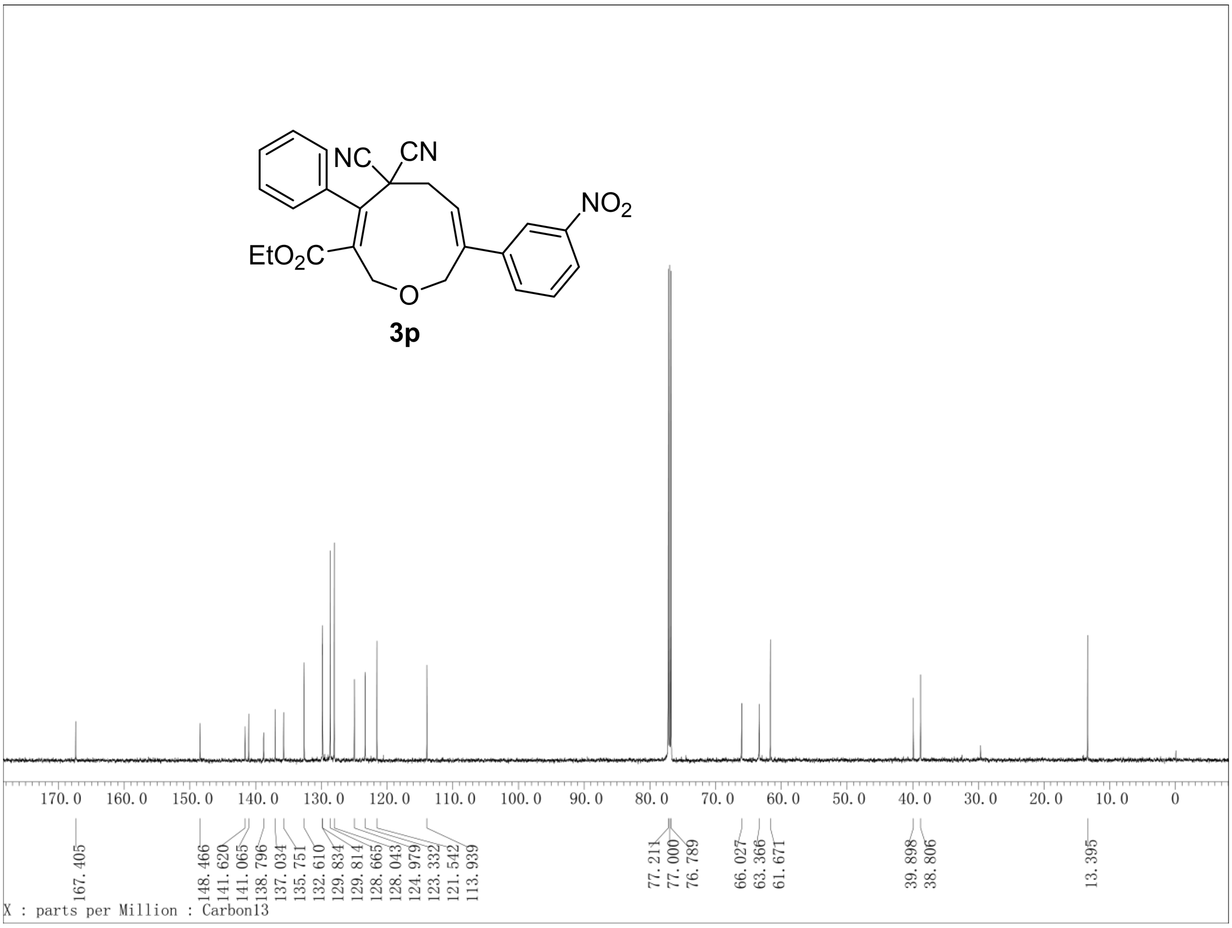
NC CN
NO2
EtO2C
O
3p
170.0 160.0 150.0 140.0 130.0 120.0 110.0 100.0 90.0 80.0 70.0 60.0 50.0 40.0 30.0 20.0 10.0 0
167.405
148.466 141.620 141.065 138.796 137.034 135.751 132.610 129.834 129.814 128.665 128.043 124.979 123.332 121.542 113.939
77.211 77.000 76.789
66.027 63.366 61.671
39.898 38.806
13.395
X : parts per Million : Carbon13

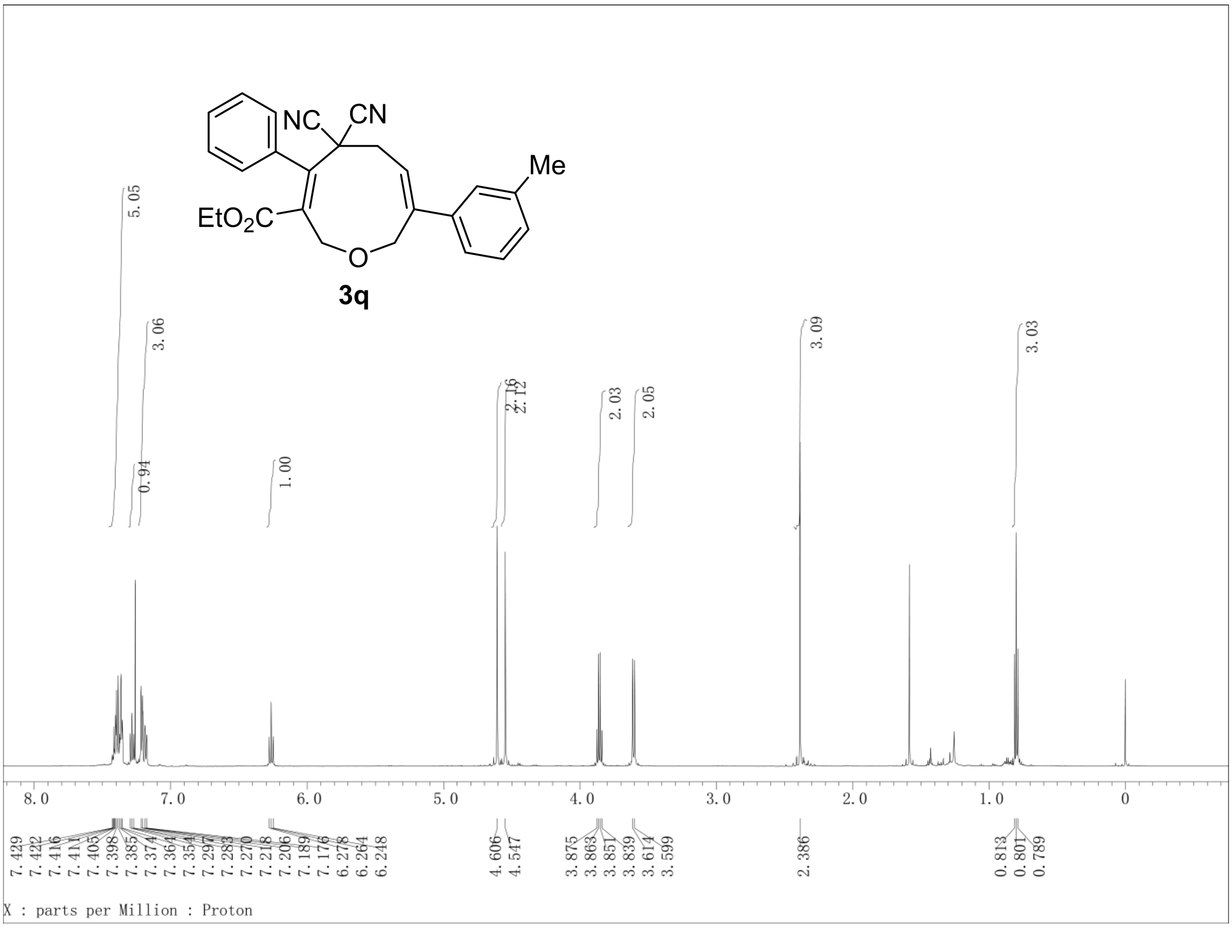
NC
CN
Me
EtO_2C
O
3q
5.05
3.06
0.94
1.00
2.16
2.12
2.03
2.05
3.09
3.03
8.0
7.0
6.0
5.0
4.0
3.0
2.0
1.0
0
7.429
7.422
7.416
7.411
7.405
7.398
7.385
7.374
7.364
7.354
7.297
7.283
7.270
7.218
7.206
7.189
7.176
6.278
6.264
6.248
4.606
4.547
3.875
3.863
3.851
3.839
3.614
3.599
2.386
0.813
0.801
0.789
X : parts per Million : Proton

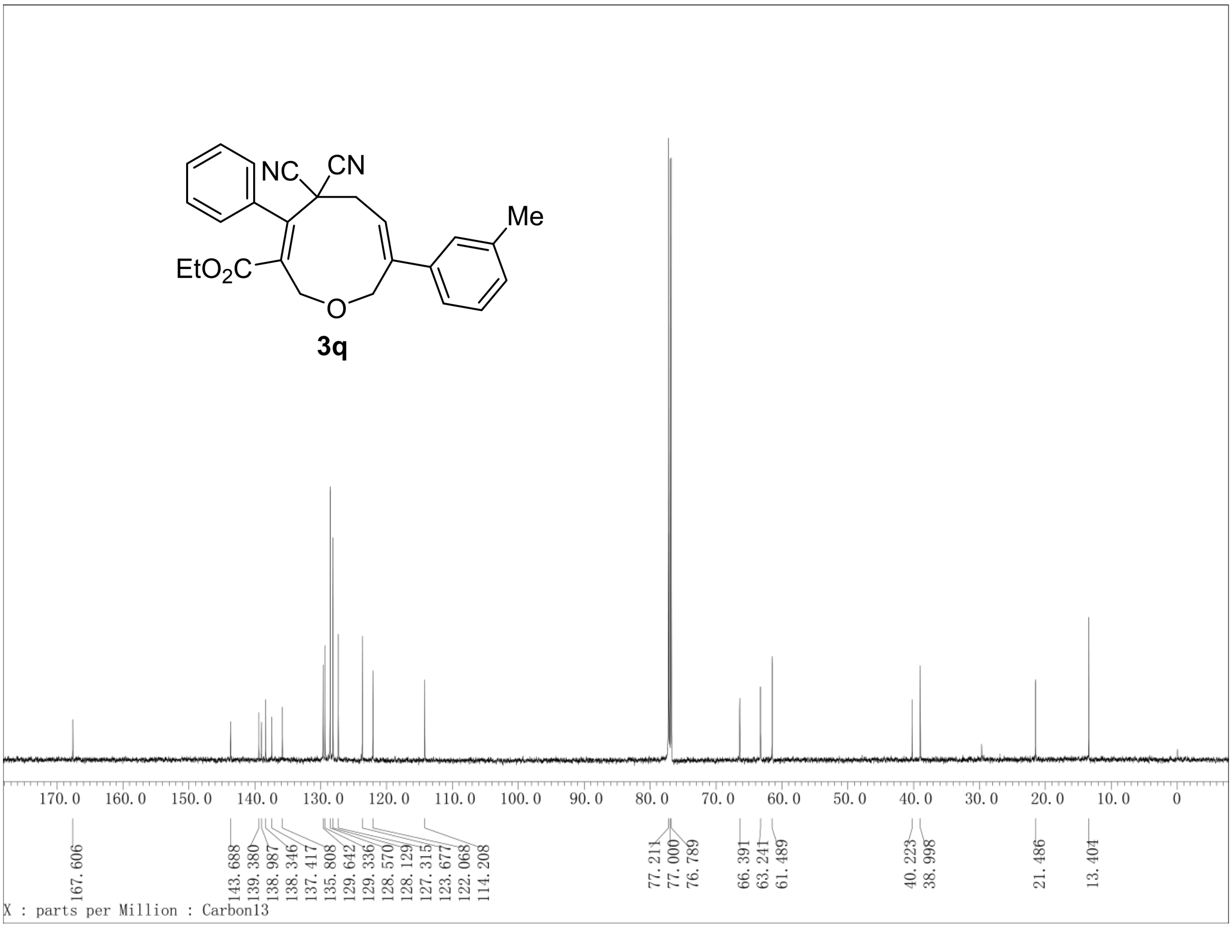
NC
CN
Me
EtO_2C
O
3q
170.0
160.0
150.0
140.0
130.0
120.0
110.0
100.0
90.0
80.0
70.0
60.0
50.0
40.0
30.0
20.0
10.0
0
167.606
143.688
139.380
138.987
138.346
137.417
135.808
129.642
129.336
128.570
128.129
127.315
123.677
122.068
114.208
77.211
77.000
76.789
66.391
63.241
61.489
40.223
38.998
21.486
13.404
X : parts per Million : Carbon13

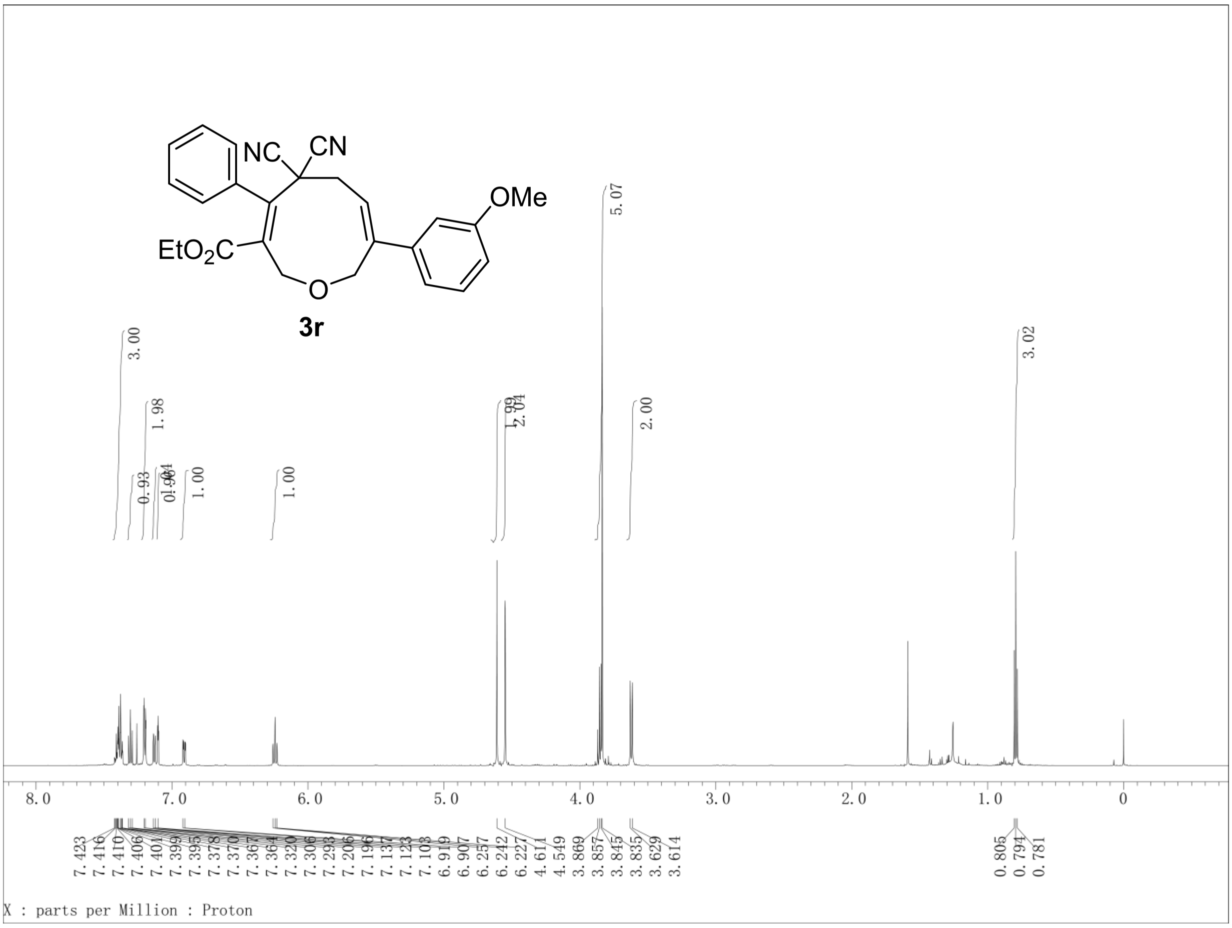
NC
CN
OMe
EtO2C
O
3r
3.00
1.98
0.93
1.00
1.00
1.00
2.00
5.07
3.02
8.0
7.0
6.0
5.0
4.0
3.0
2.0
1.0
0
7.423
7.416
7.410
7.406
7.401
7.399
7.395
7.378
7.370
7.367
7.364
7.320
7.306
7.293
7.206
7.196
7.137
7.123
7.103
6.919
6.907
6.257
6.242
6.227
4.611
4.549
3.869
3.857
3.845
3.835
3.629
3.614
0.805
0.794
0.781
X : parts per Million : Proton

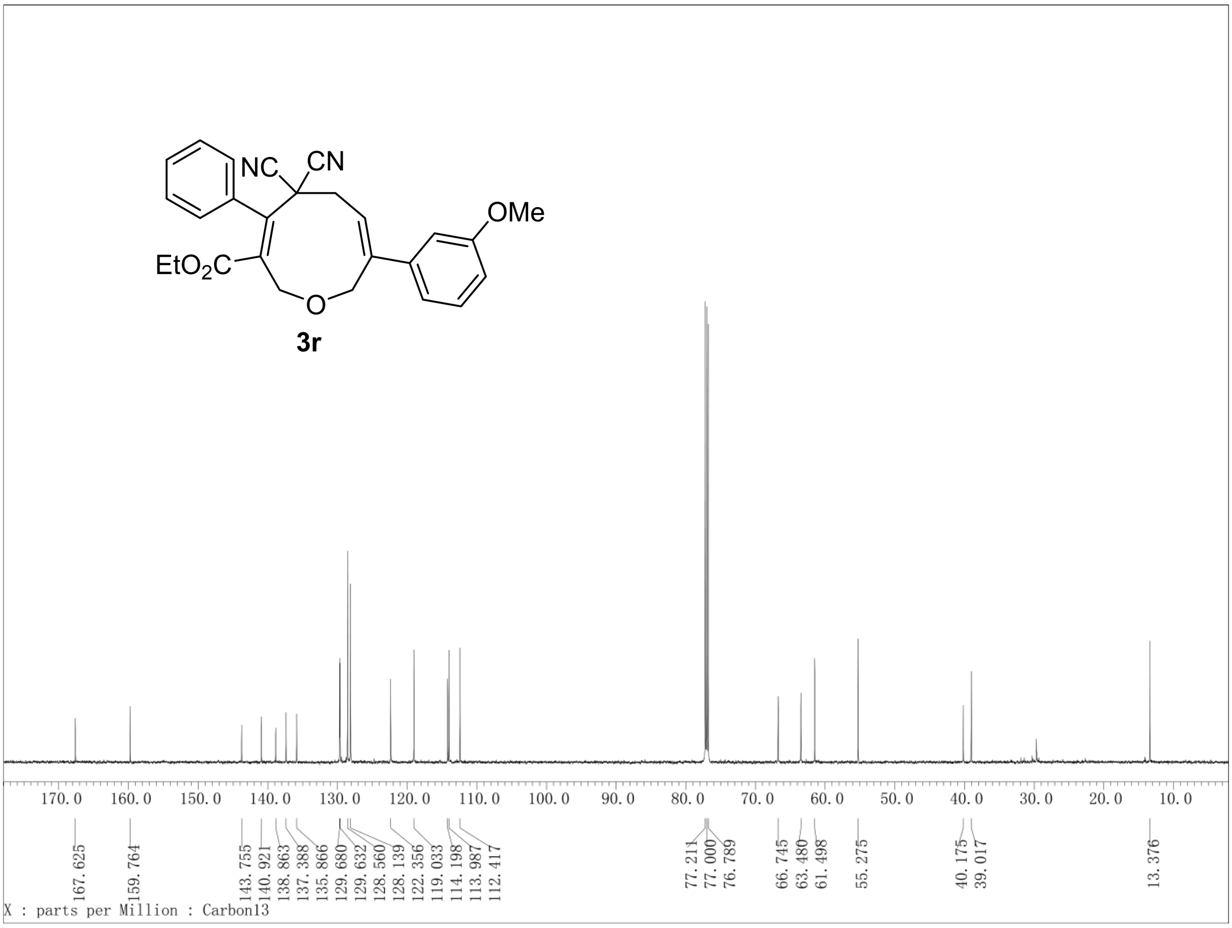
NC
CN
OMe
EtO2C
O
3r
170.0
160.0
150.0
140.0
130.0
120.0
110.0
100.0
90.0
80.0
70.0
60.0
50.0
40.0
30.0
20.0
10.0
167.625
159.764
143.755
140.921
138.863
137.388
135.866
129.680
129.632
128.560
128.139
122.356
119.033
114.198
113.987
112.417
77.211
77.000
76.789
66.745
63.480
61.498
55.275
40.175
39.017
13.376
X : parts per Million : Carbon13

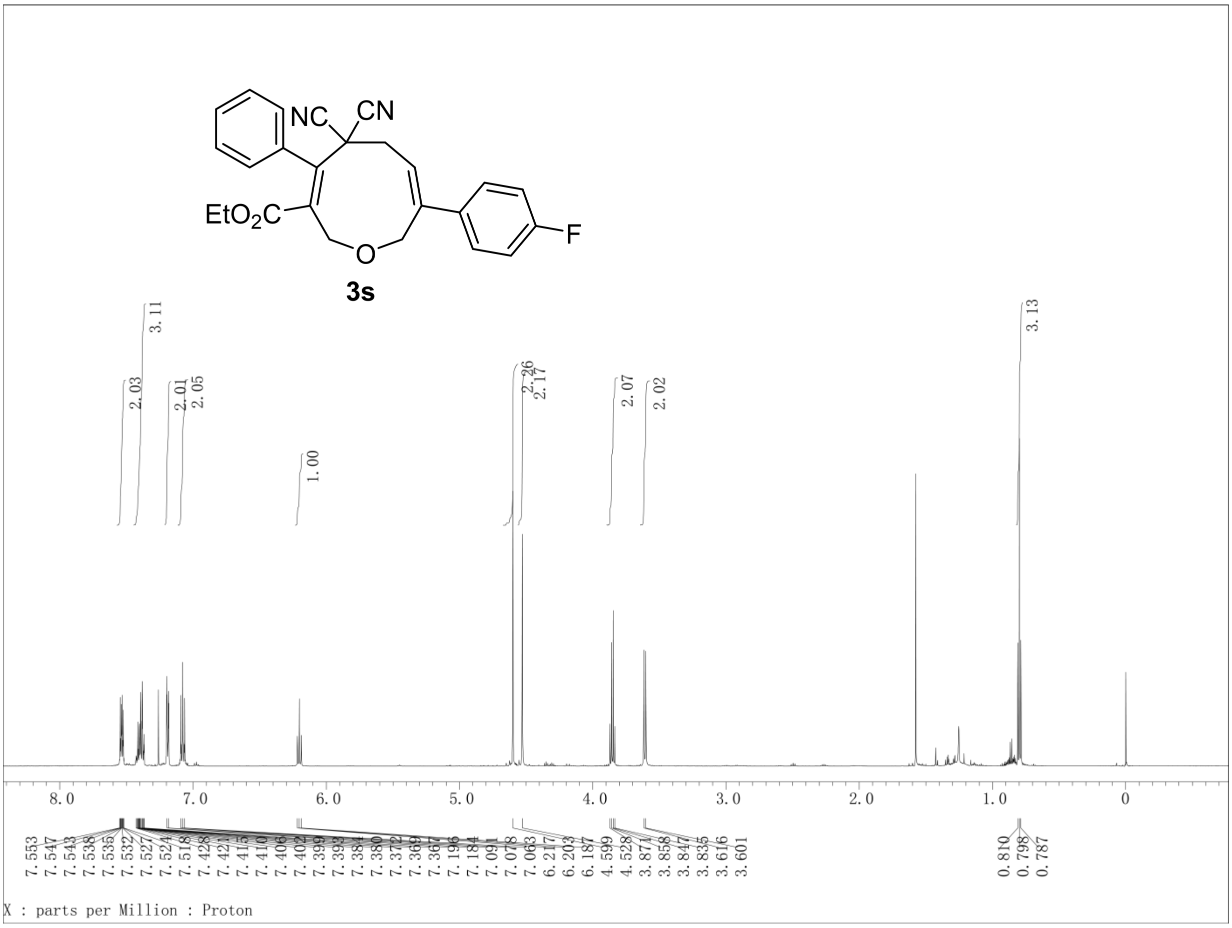

NC
CN
EtO2C
O
F
3s
8.0
7.0
6.0
5.0
4.0
3.0
2.0
1.0
0
X : parts per Million : Proton

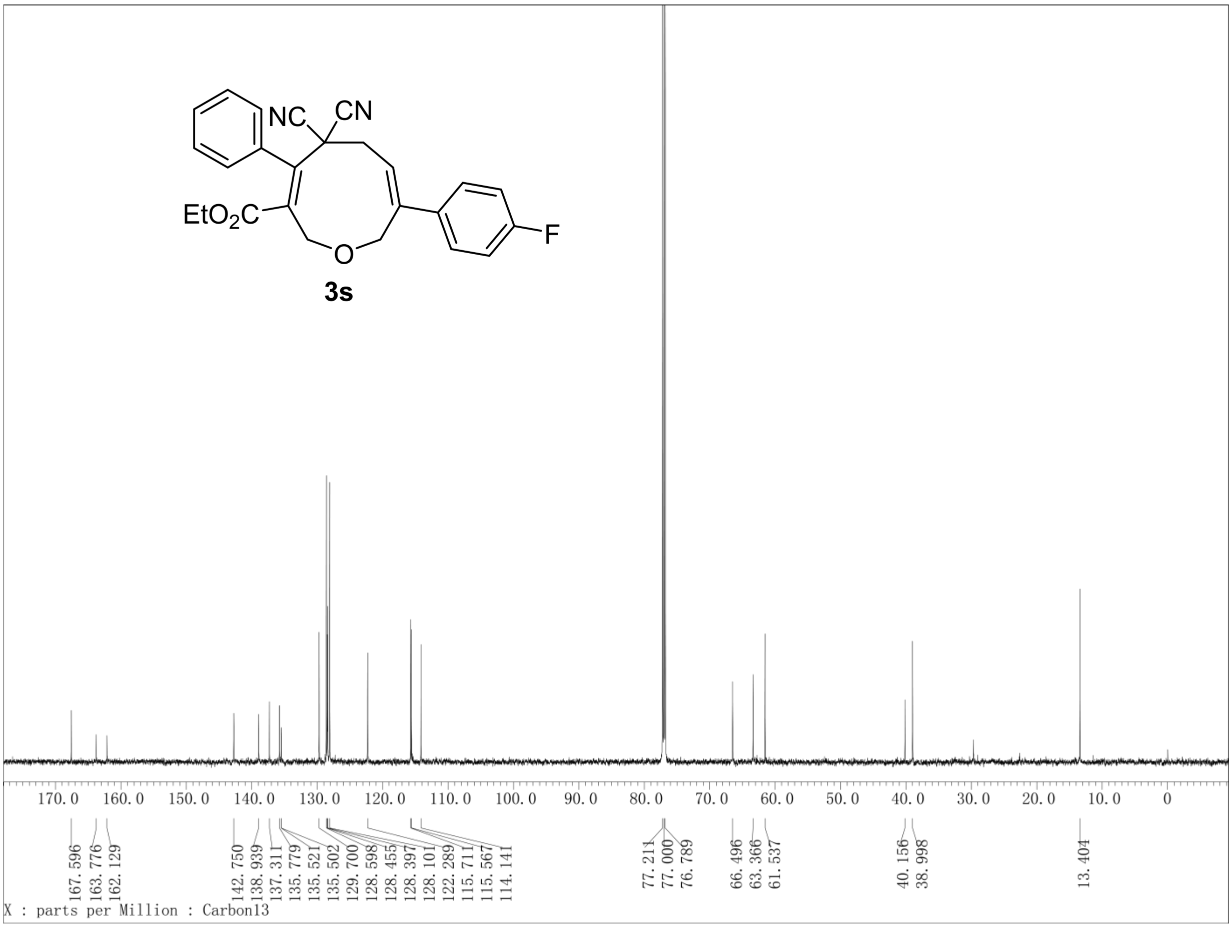

NC
CN
EtO2C
O
F
3s
170.0
160.0
150.0
140.0
130.0
120.0
110.0
100.0
90.0
80.0
70.0
60.0
50.0
40.0
30.0
20.0
10.0
0
167.596
163.776
162.129
142.750
138.939
137.311
135.779
135.521
135.502
129.700
128.598
128.455
128.397
128.101
122.289
115.711
115.567
114.141
77.211
77.000
76.789
66.496
63.366
61.537
40.156
38.998
13.404
X : parts per Million : Carbon13

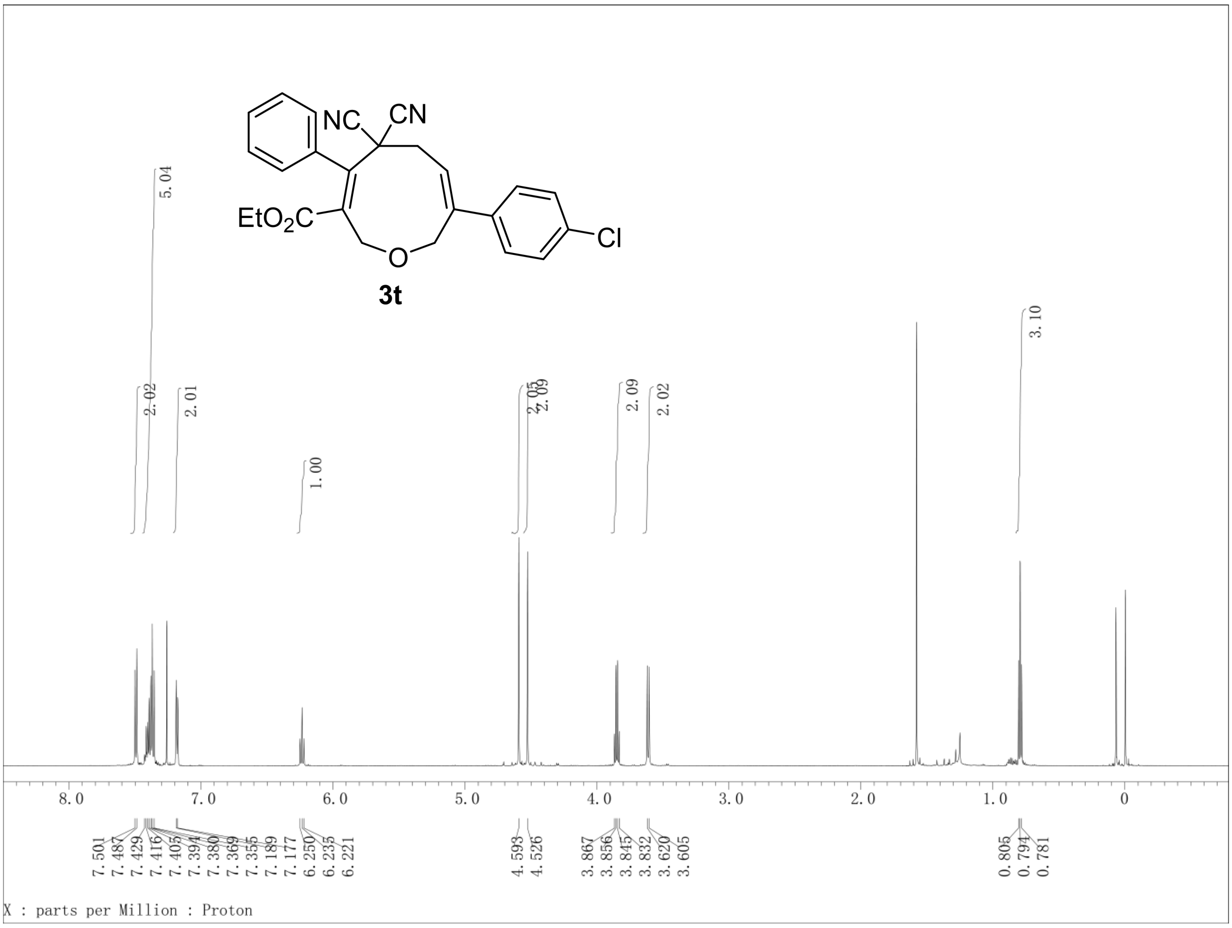
NC
CN
EtO2C
O
Cl
3t
5.04
2.02
2.01
1.00
2.05
2.09
2.09
2.02
3.10
8.0 7.0 6.0 5.0 4.0 3.0 2.0 1.0 0
7.501 7.487 7.429 7.416 7.405 7.394 7.380 7.369 7.355 7.189 7.177 6.250 6.235 6.221
4.593 4.526
3.867 3.856 3.845 3.832 3.620 3.605
0.805 0.794 0.781
X : parts per Million : Proton

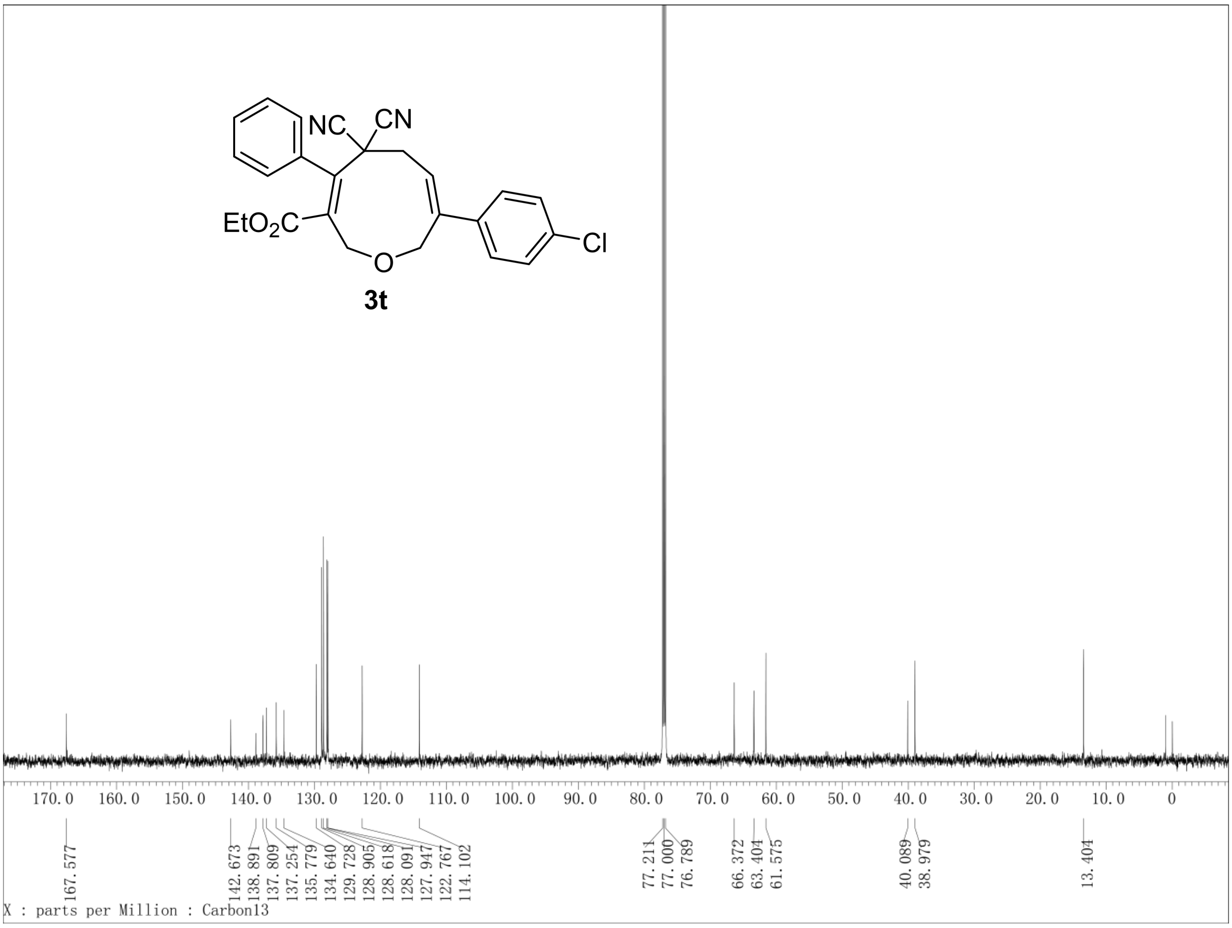
NC
CN
EtO2C
O
Cl
3t
170.0 160.0 150.0 140.0 130.0 120.0 110.0 100.0 90.0 80.0 70.0 60.0 50.0 40.0 30.0 20.0 10.0 0
167.577
142.673 138.891 137.809 137.254 135.779 134.640 129.728 128.905 128.618 128.091 127.947 122.767 114.102
77.211 77.000 76.789
66.372 63.404 61.575
40.089 38.979
13.404
X : parts per Million : Carbon13

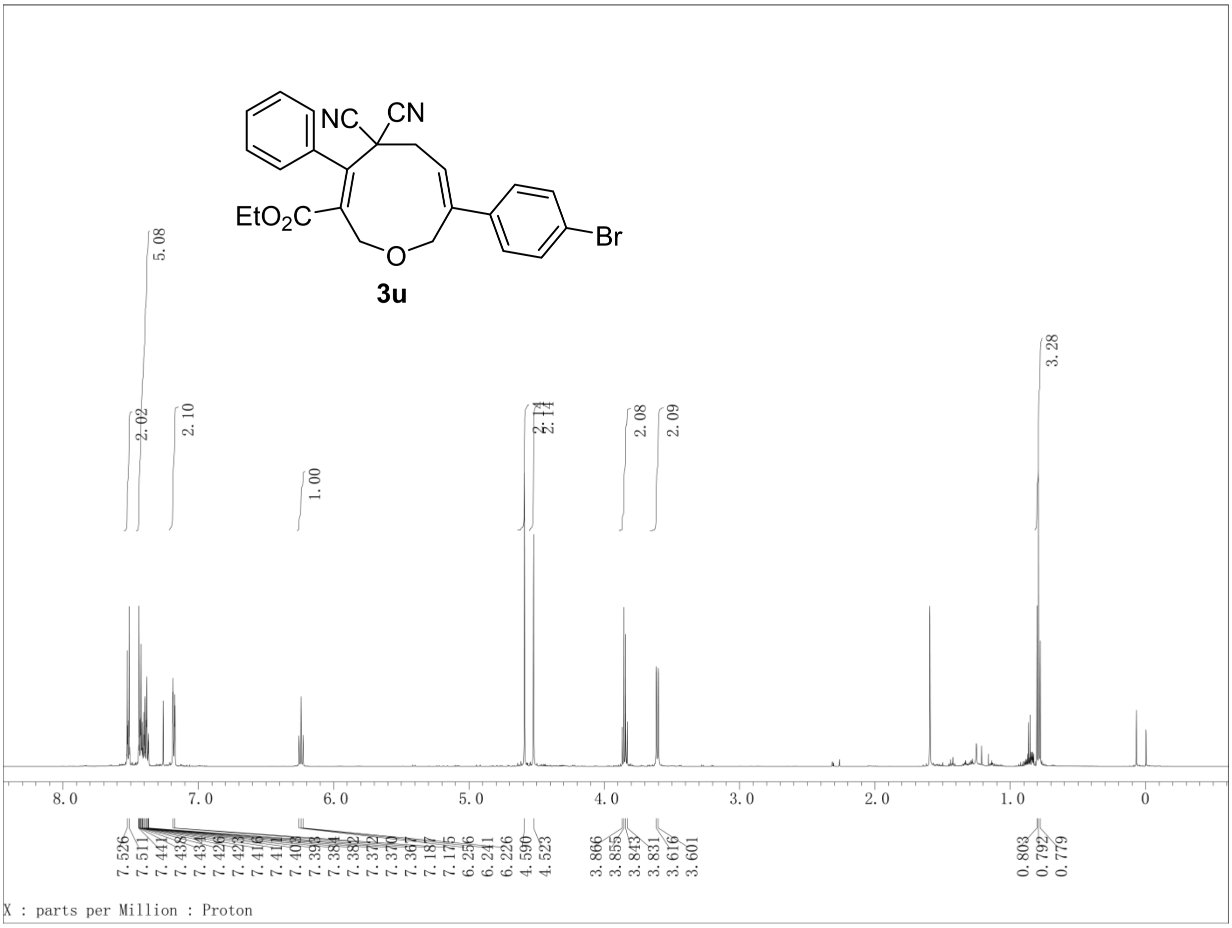
NC
CN
EtO2C
O
Br
3u
5.08
2.02
2.10
1.00
2.14
2.14
2.08
2.09
3.28
8.0
7.0
6.0
5.0
4.0
3.0
2.0
1.0
0
7.526
7.511
7.441
7.438
7.434
7.426
7.423
7.416
7.411
7.403
7.393
7.384
7.382
7.372
7.370
7.367
7.187
7.175
6.256
6.241
6.226
4.590
4.523
3.866
3.855
3.843
3.831
3.616
3.601
0.803
0.792
0.779
X : parts per Million : Proton

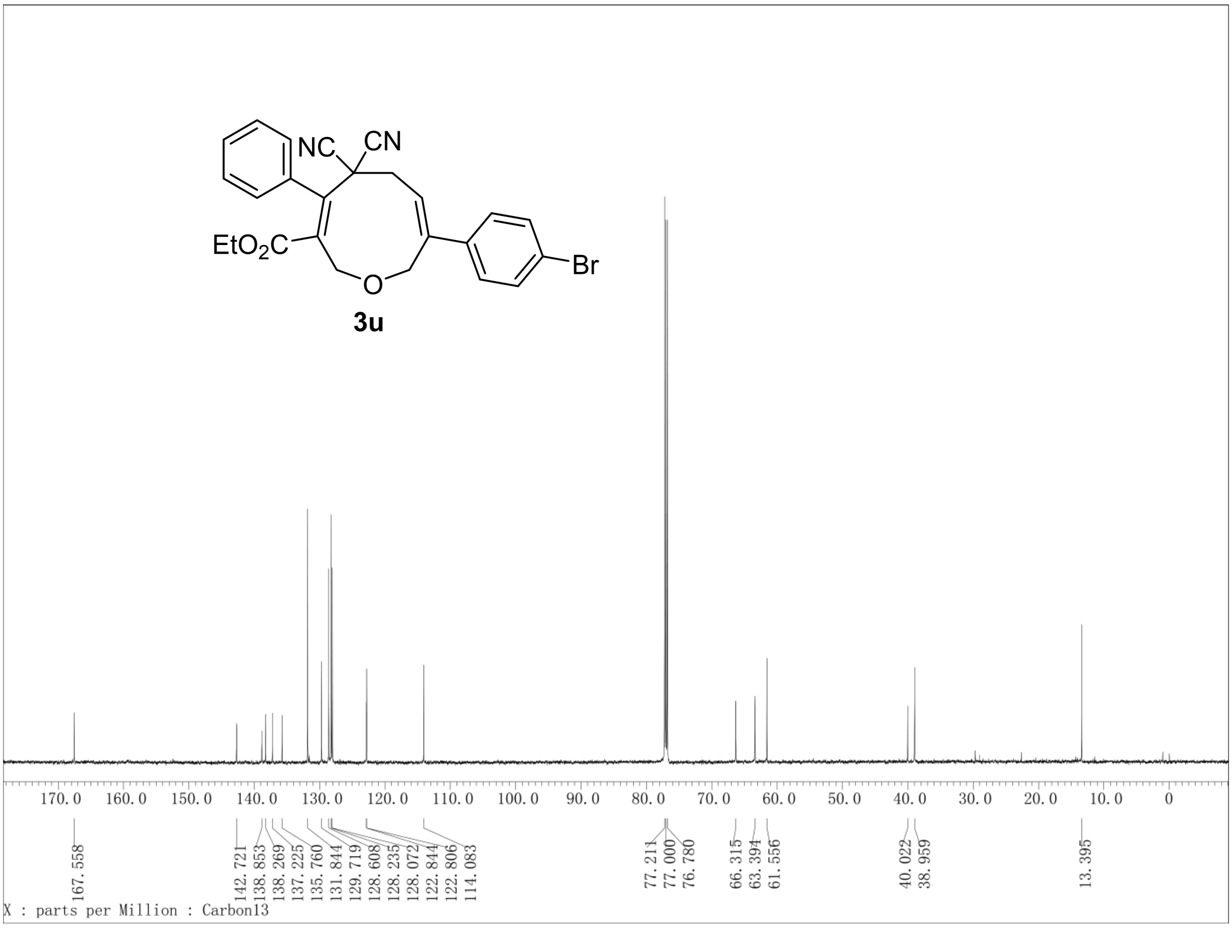
NC
CN
EtO2C
O
Br
3u
170.0
160.0
150.0
140.0
130.0
120.0
110.0
100.0
90.0
80.0
70.0
60.0
50.0
40.0
30.0
20.0
10.0
0
167.558
142.721
138.853
138.269
137.225
135.760
131.844
129.719
128.608
128.235
128.072
122.844
122.806
114.083
77.211
77.000
76.780
66.315
63.394
61.556
40.022
38.959
13.395
X : parts per Million : Carbon13

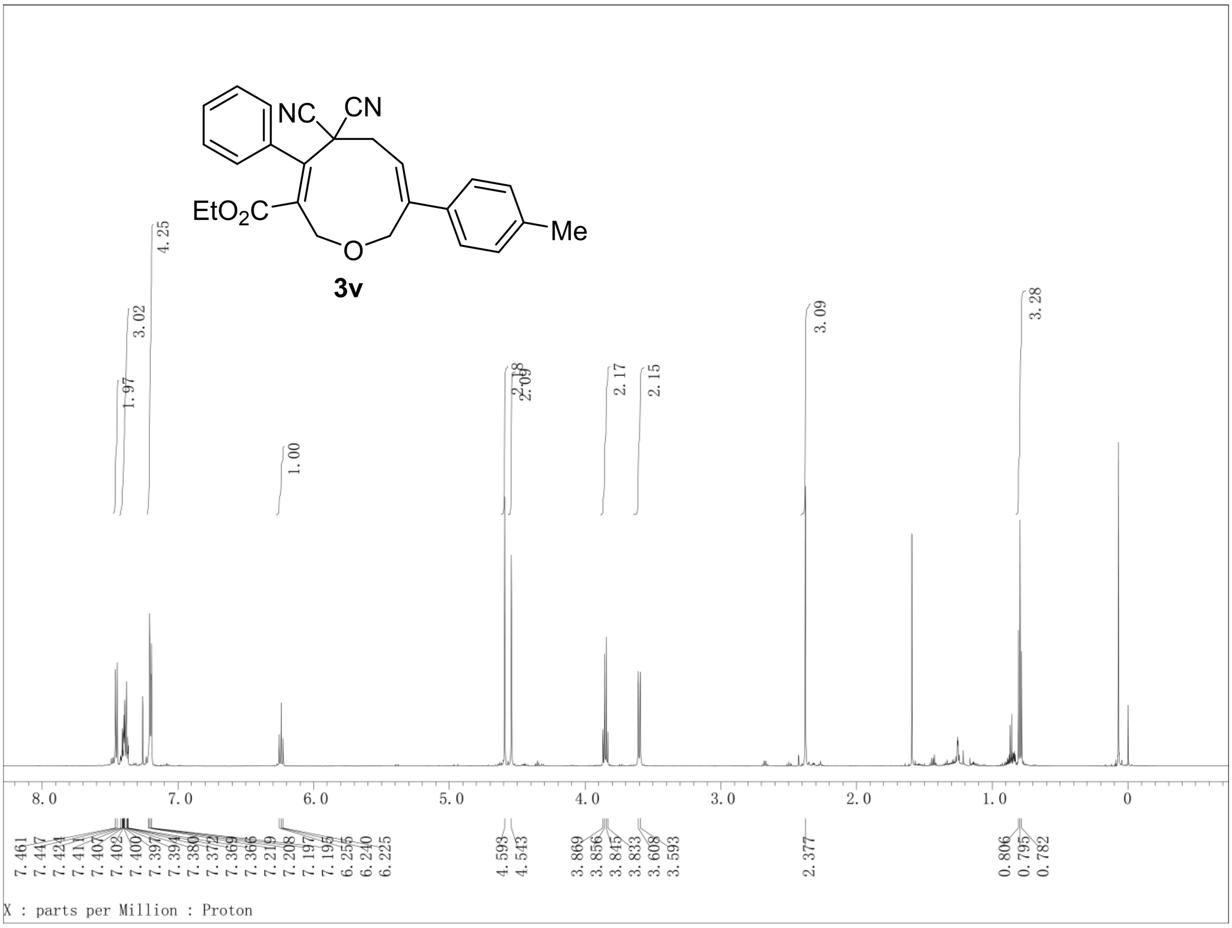
NC
CN
EtO2C
O
Me
3v
1.97
3.02
4.25
1.00
2.18
2.09
2.17
2.15
3.09
3.28
8.0 7.0 6.0 5.0 4.0 3.0 2.0 1.0 0
7.461 7.447 7.424 7.411 7.407 7.402 7.400 7.397 7.394 7.380 7.372 7.369 7.366 7.219 7.208 7.197 7.195 6.255 6.240 6.225
4.593 4.543
3.869 3.856 3.845 3.833 3.608 3.593
2.377
0.806 0.795 0.782
X : parts per Million : Proton

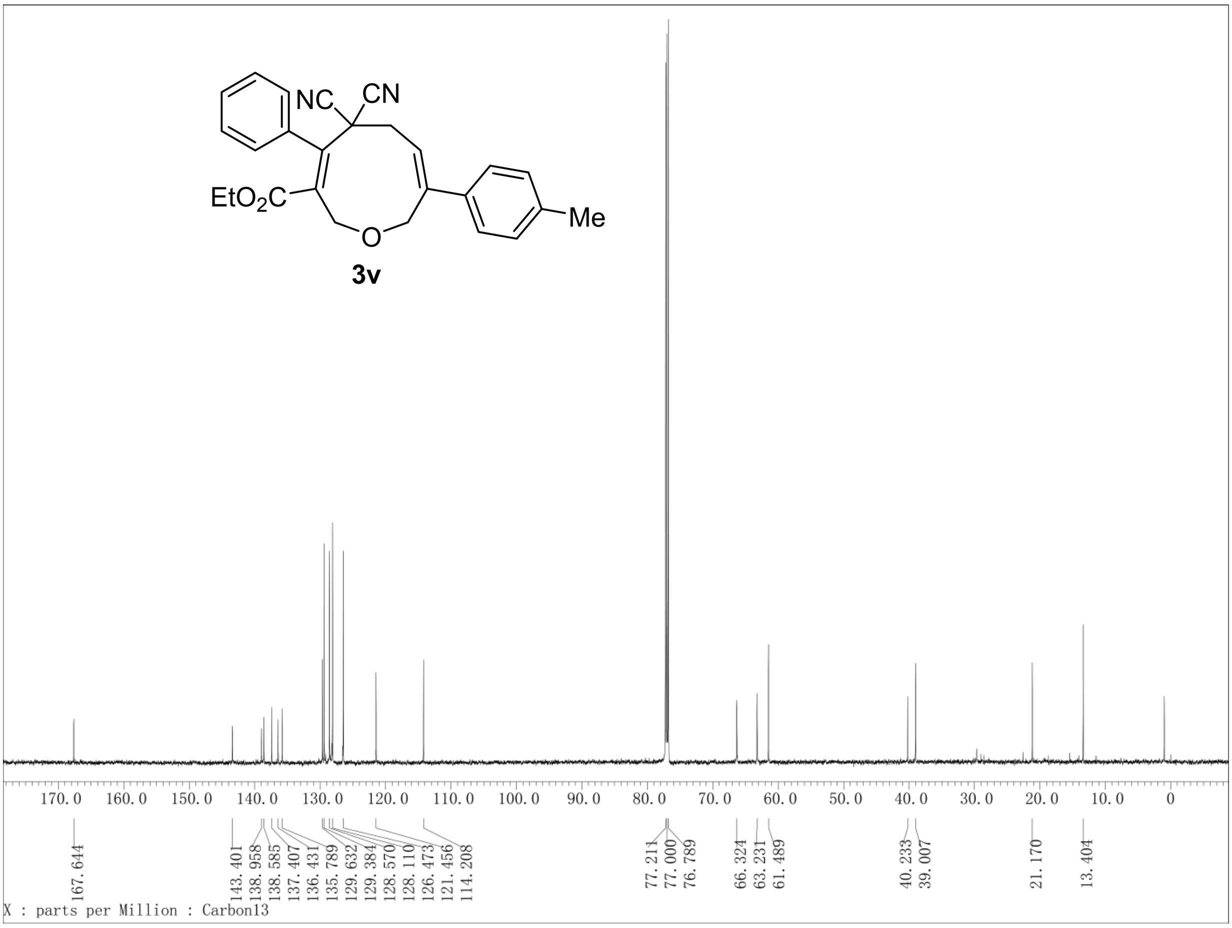
NC
CN
EtO2C
O
Me
3v
170.0 160.0 150.0 140.0 130.0 120.0 110.0 100.0 90.0 80.0 70.0 60.0 50.0 40.0 30.0 20.0 10.0 0
167.644
143.401 138.958 138.585 137.407 136.431 135.789 129.632 129.384 128.570 128.110 126.473 121.456 114.208
77.211 77.000 76.789
66.324 63.231 61.489
40.233 39.007
21.170
13.404
X : parts per Million : Carbon13

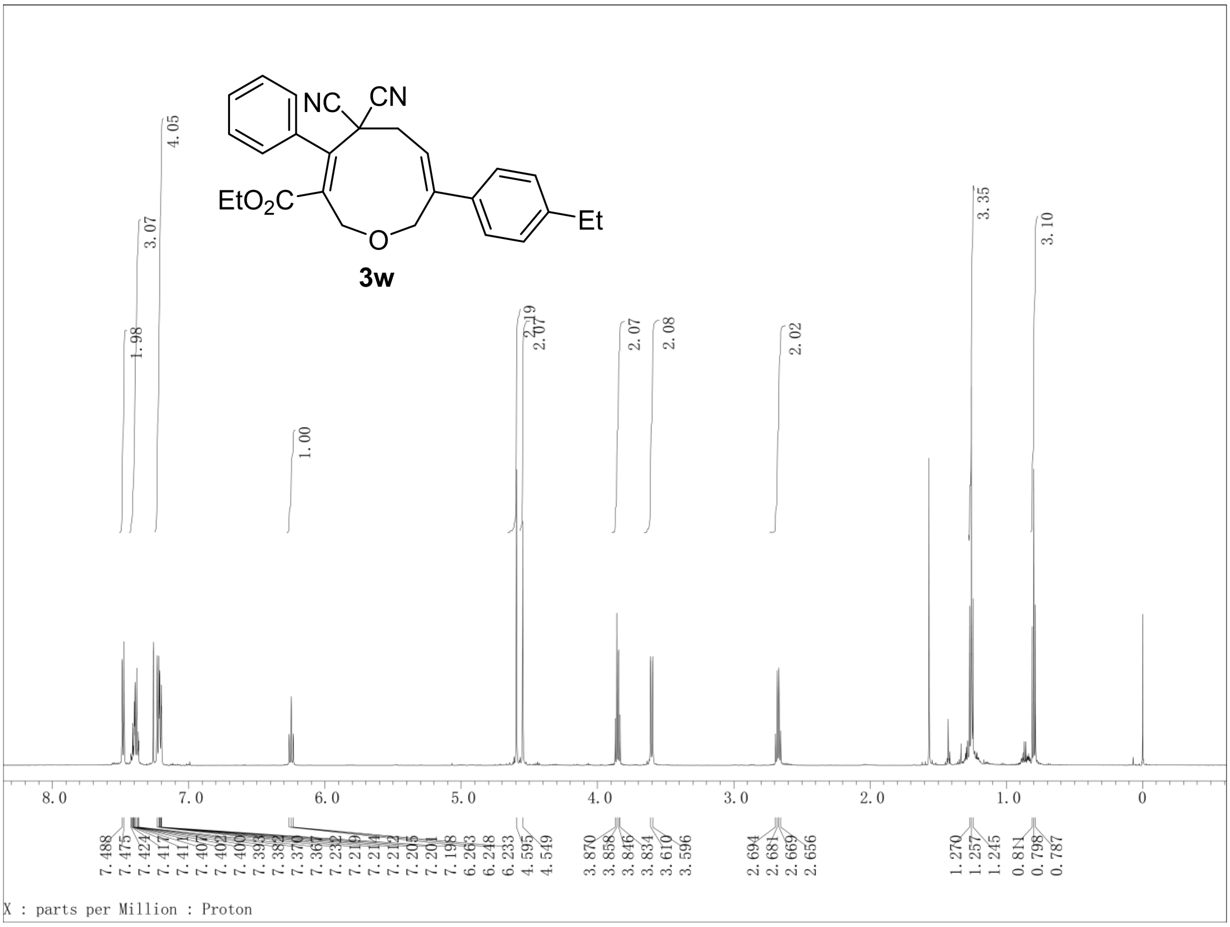
NC
CN
EtO2C
O
Et
3w
X : parts per Million : Proton

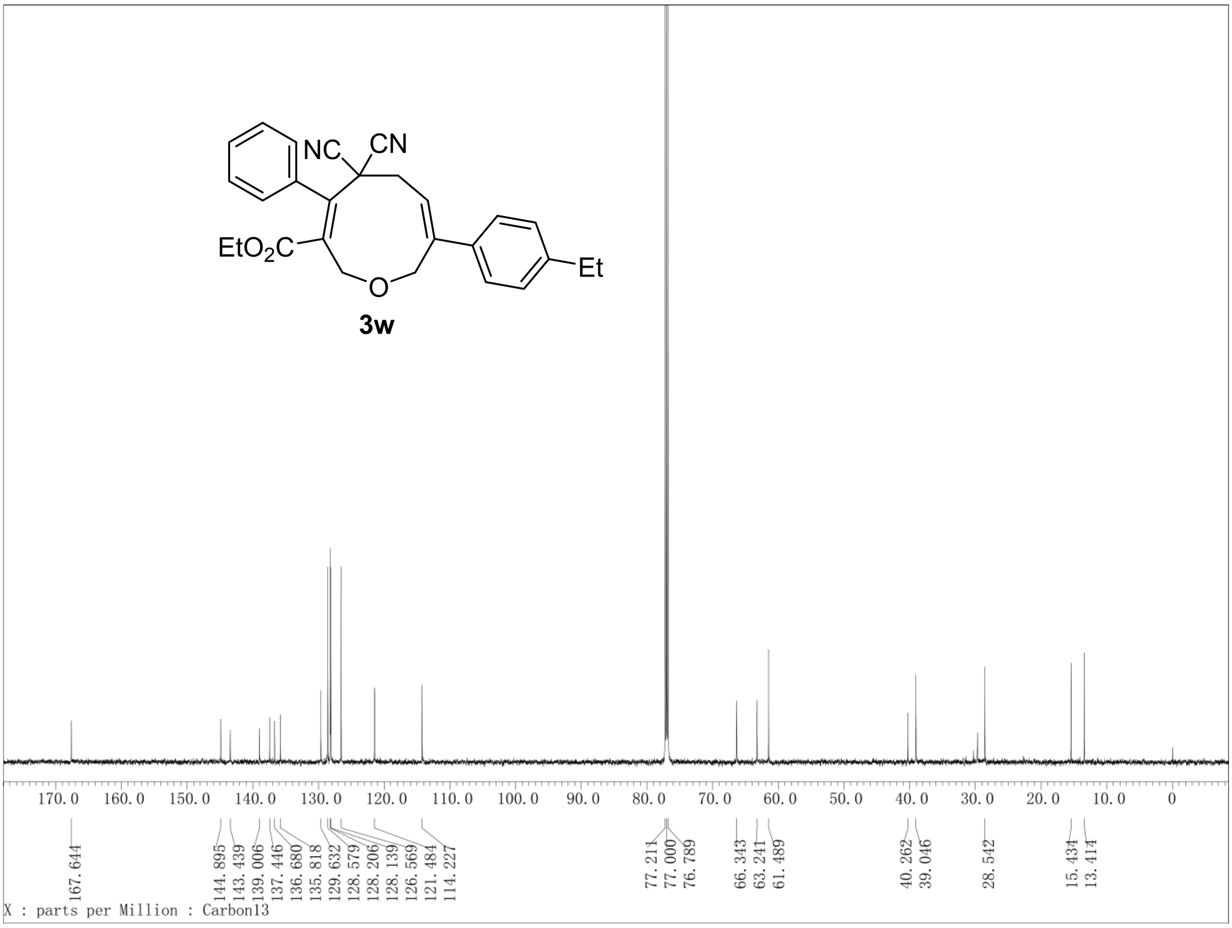
NC
CN
EtO2C
O
Et
3w
X : parts per Million : Carbon13

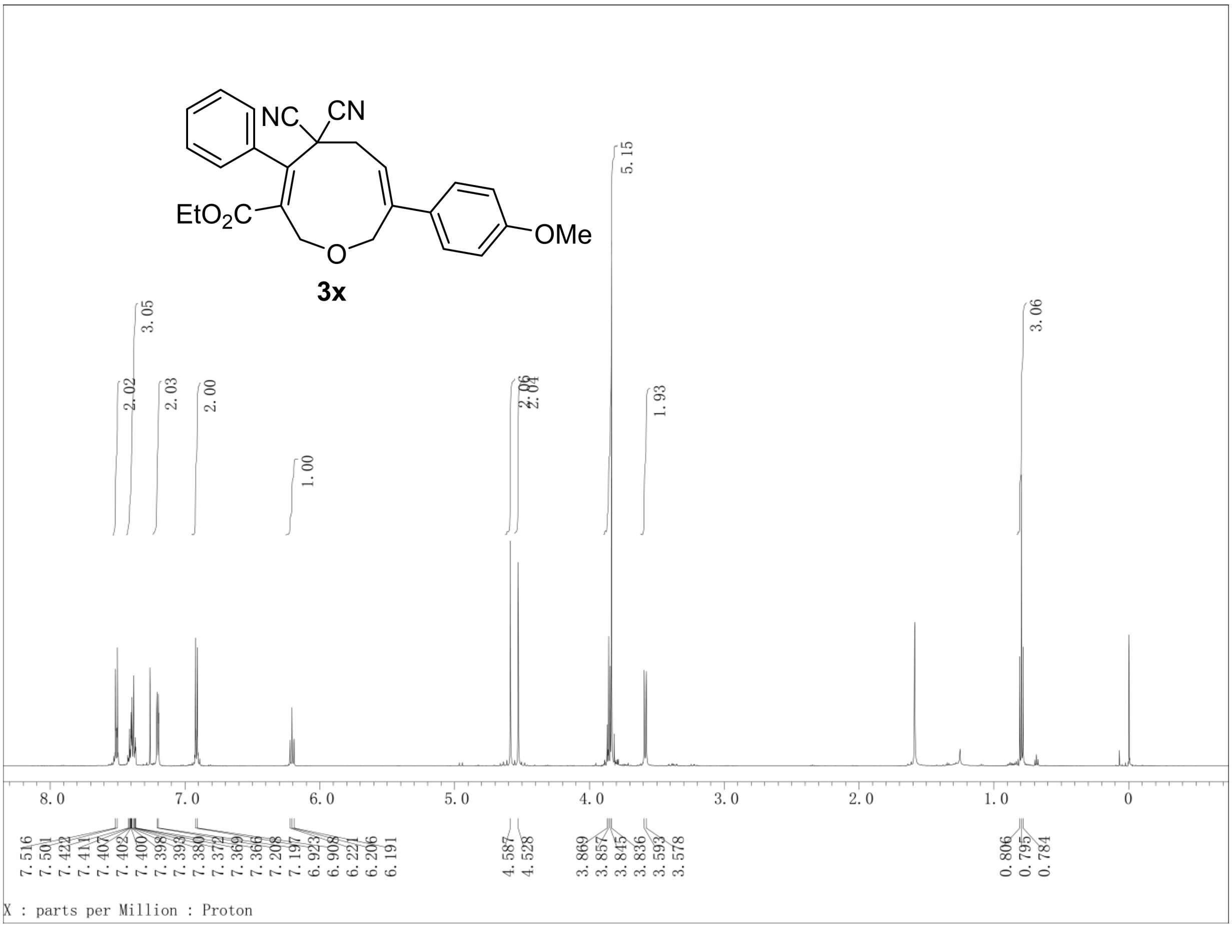
NC
CN
EtO2C
O
OMe
3x
3.05
2.02
2.03
2.00
1.00
2.06
2.04
5.15
1.93
3.06
8.0
7.0
6.0
5.0
4.0
3.0
2.0
1.0
0
7.516
7.501
7.422
7.411
7.407
7.402
7.400
7.398
7.393
7.380
7.372
7.369
7.366
7.208
7.197
6.923
6.908
6.221
6.206
6.191
4.587
4.528
3.869
3.857
3.845
3.836
3.593
3.578
0.806
0.795
0.784
X : parts per Million : Proton

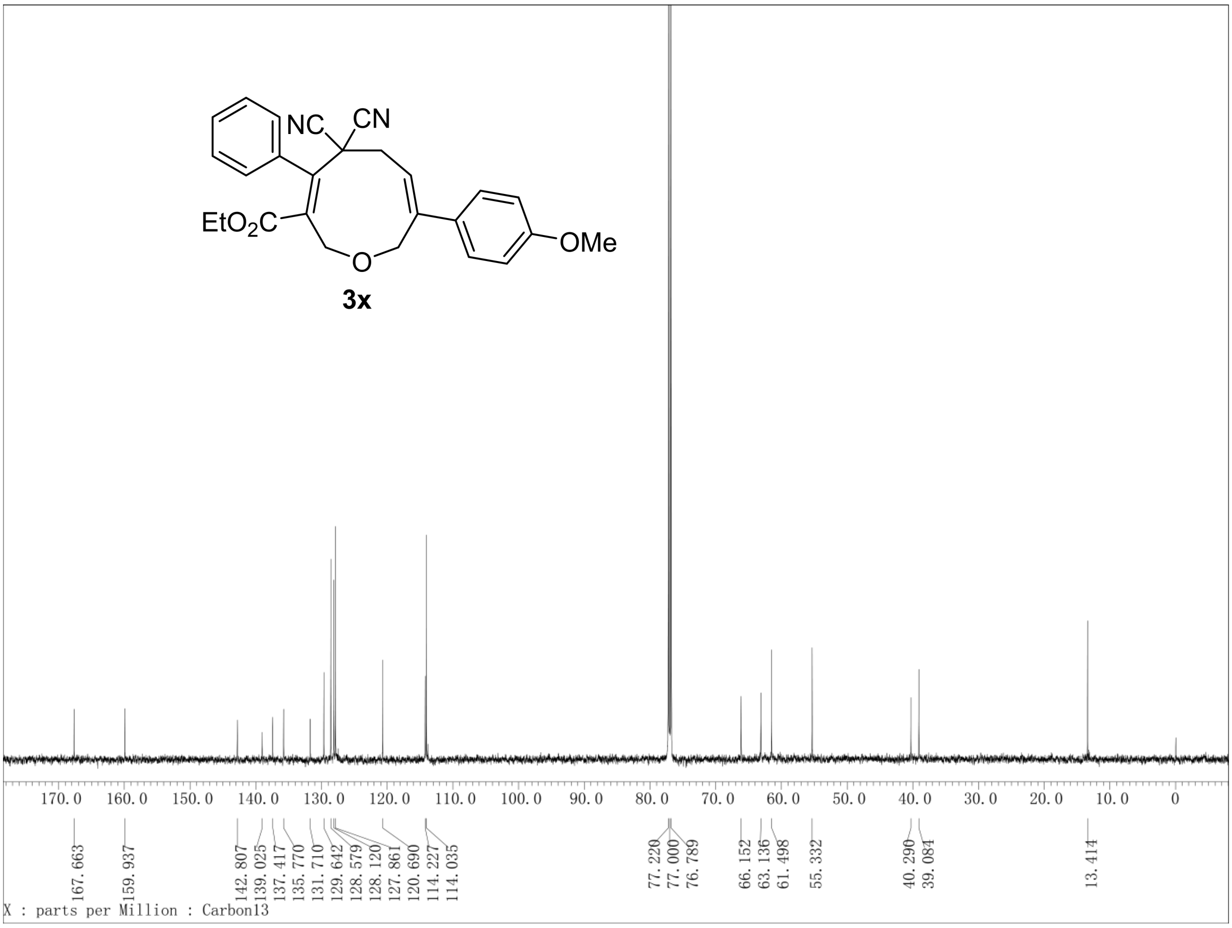
NC
CN
EtO2C
O
OMe
3x
170.0
160.0
150.0
140.0
130.0
120.0
110.0
100.0
90.0
80.0
70.0
60.0
50.0
40.0
30.0
20.0
10.0
0
167.663
159.937
142.807
139.025
137.417
135.770
131.710
129.642
128.579
128.120
127.861
120.690
114.227
114.035
77.220
77.000
76.789
66.152
63.136
61.498
55.332
40.290
39.084
13.414
X : parts per Million : Carbon13

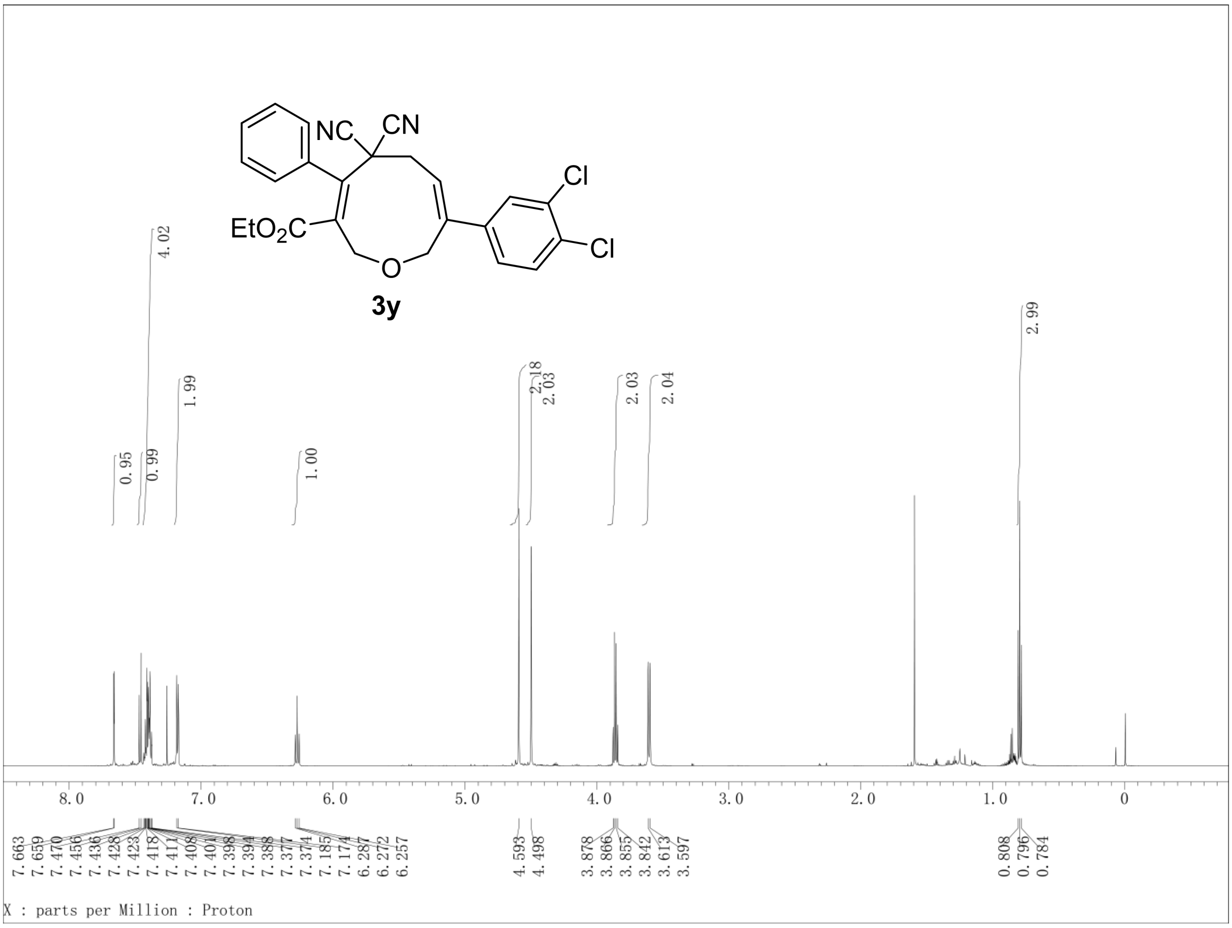
NC
CN
Cl
Cl
EtO2C
O
3y
X : parts per Million : Proton

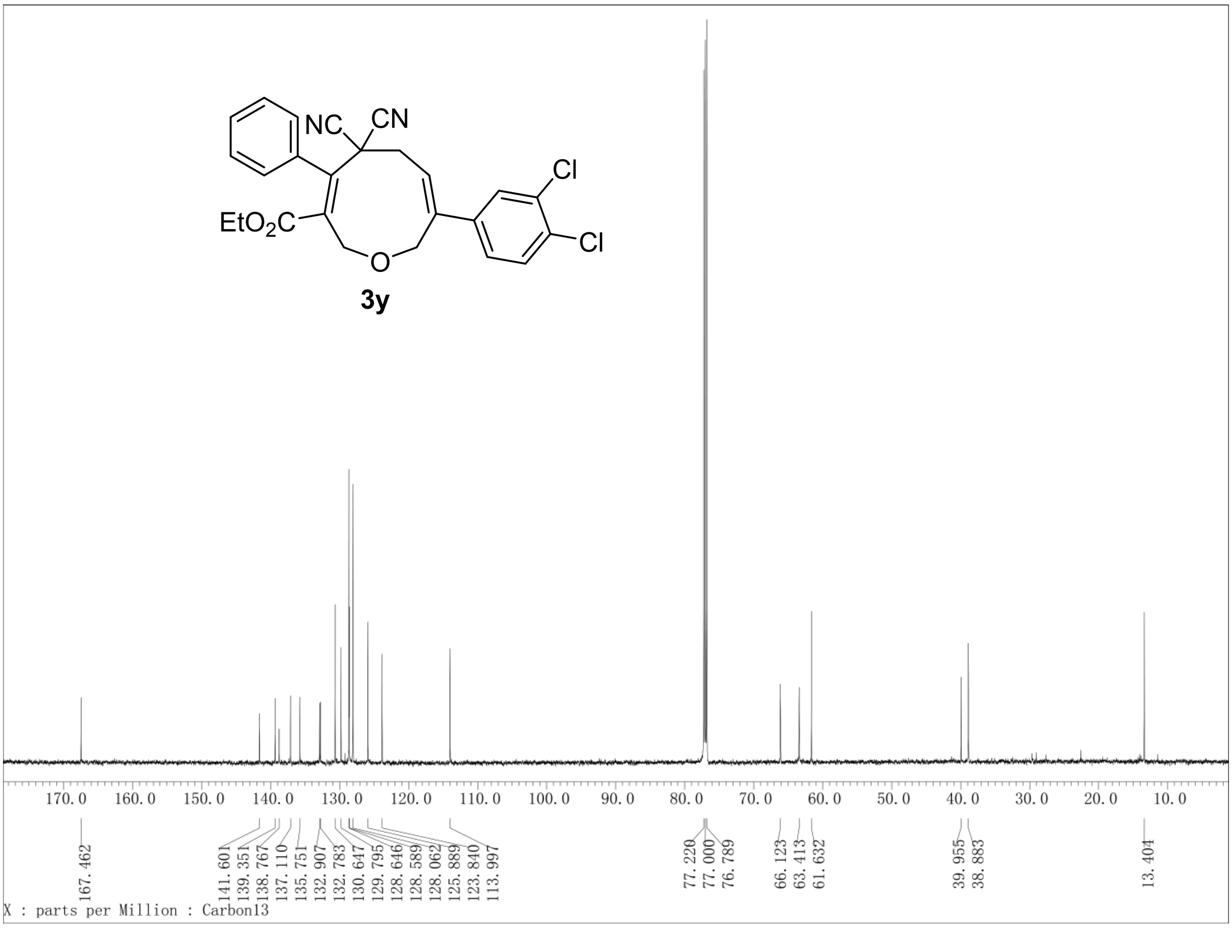
NC
CN
Cl
Cl
EtO2C
O
3y
X : parts per Million : Carbon13

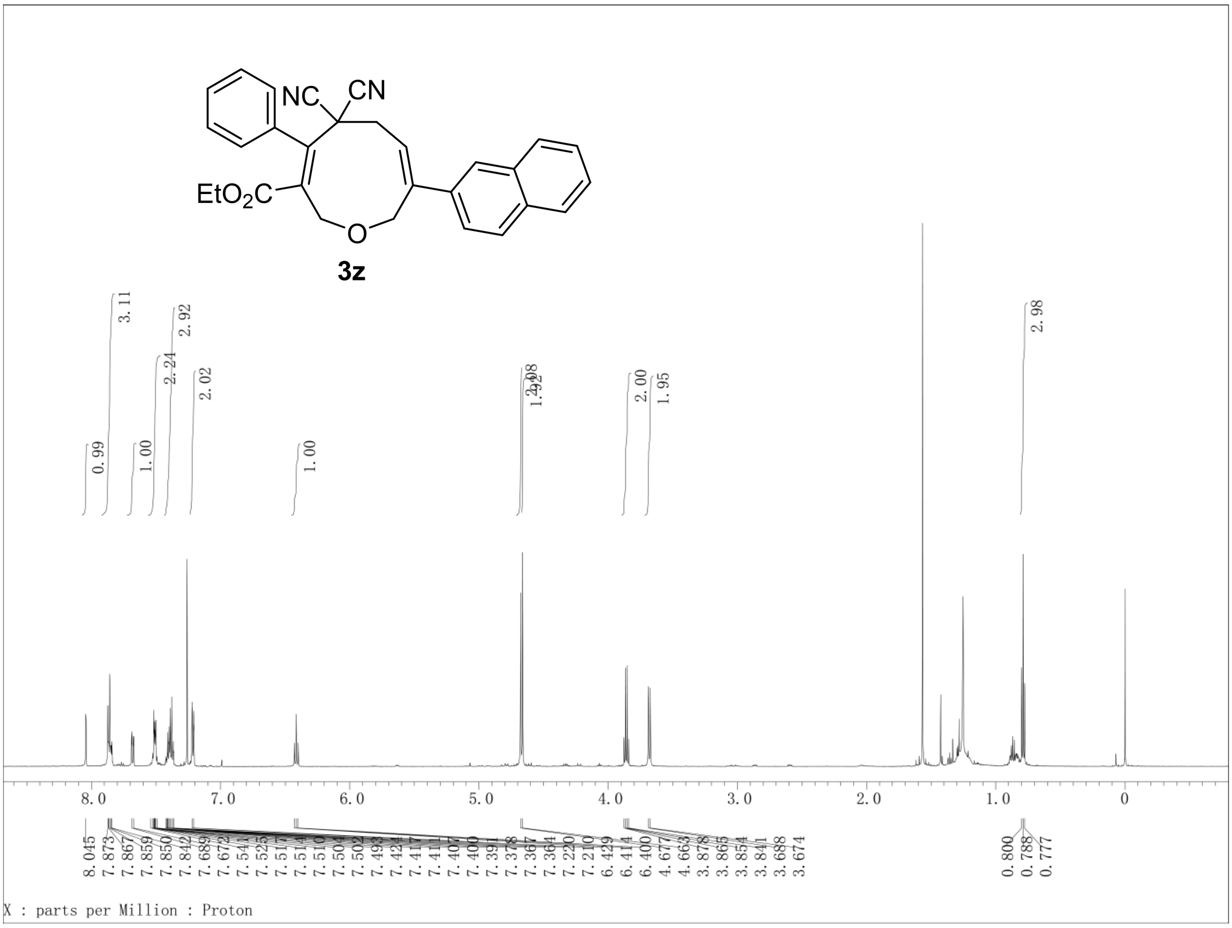
NC
CN
EtO2C
O
3z
X : parts per Million : Proton

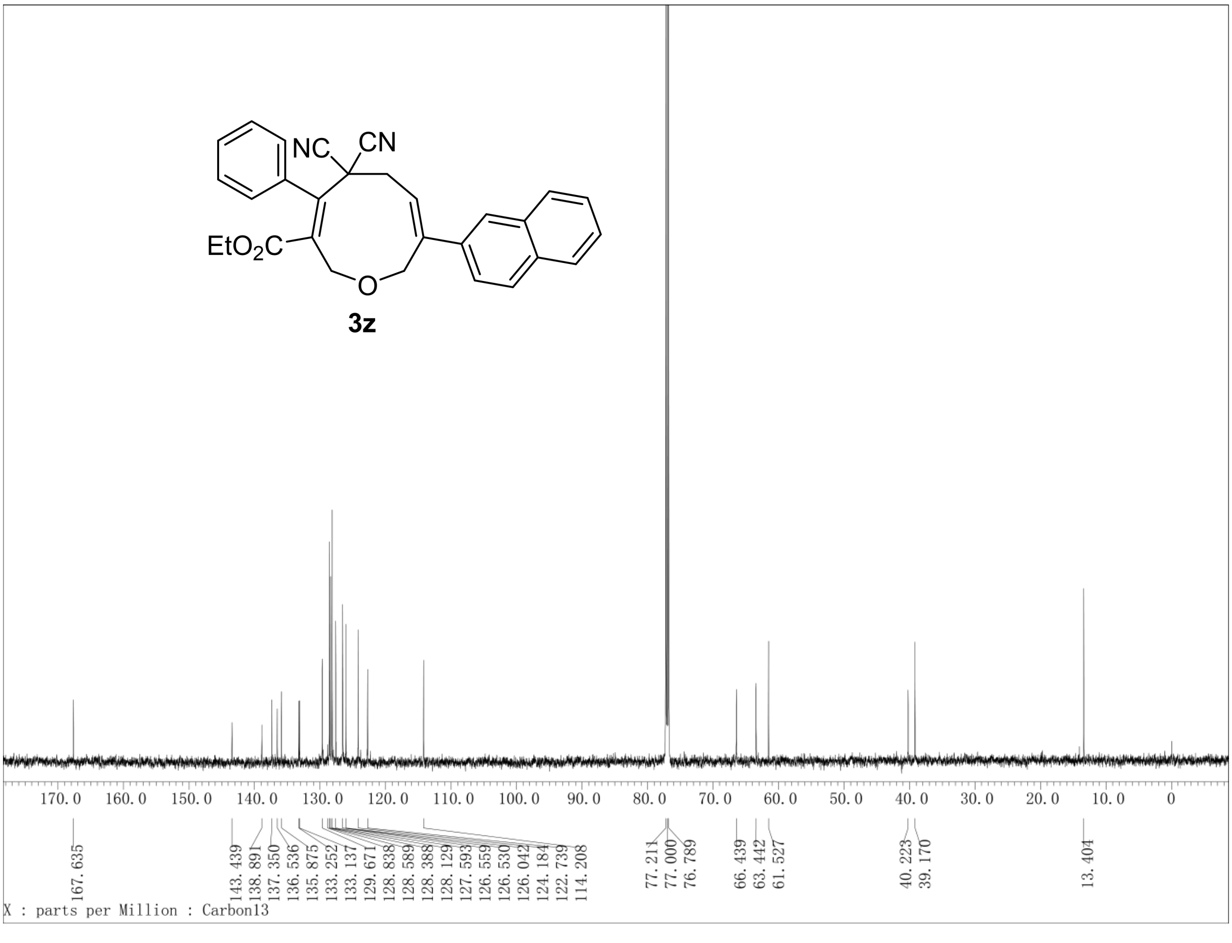
NC
CN
EtO2C
O
3z
X : parts per Million : Carbon13

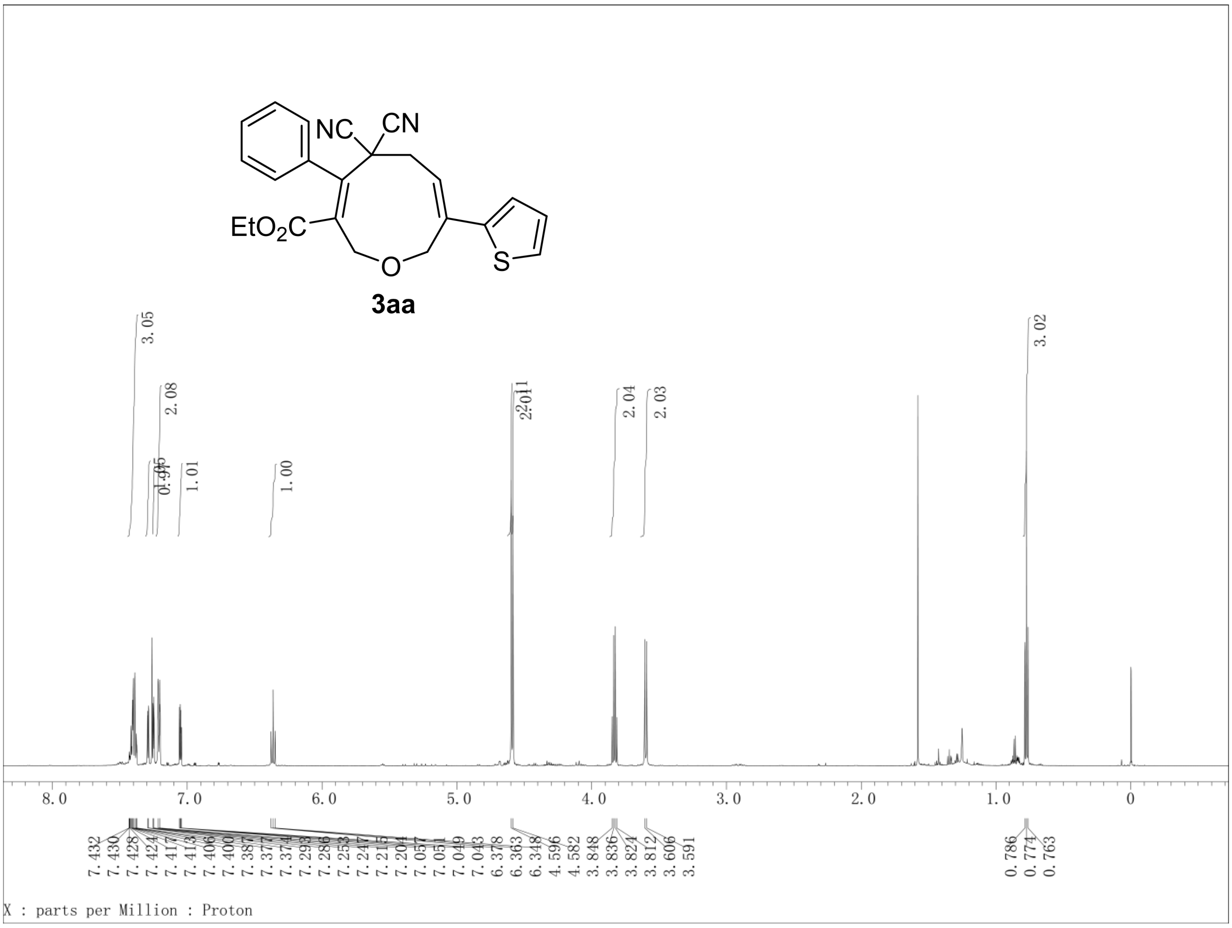
NC
CN
EtO2C
O
S
3aa
3.05
2.08
1.01
1.00
2.11
2.04
2.03
3.02
8.0 7.0 6.0 5.0 4.0 3.0 2.0 1.0 0
7.432 7.430 7.428 7.424 7.417 7.413 7.406 7.400 7.387 7.377 7.374 7.293 7.286 7.253 7.247 7.215 7.204 7.057 7.051 7.049 7.043 6.378 6.363 6.348 4.596 4.582 3.848 3.836 3.824 3.812 3.606 3.591
0.786 0.774 0.763
X : parts per Million : Proton

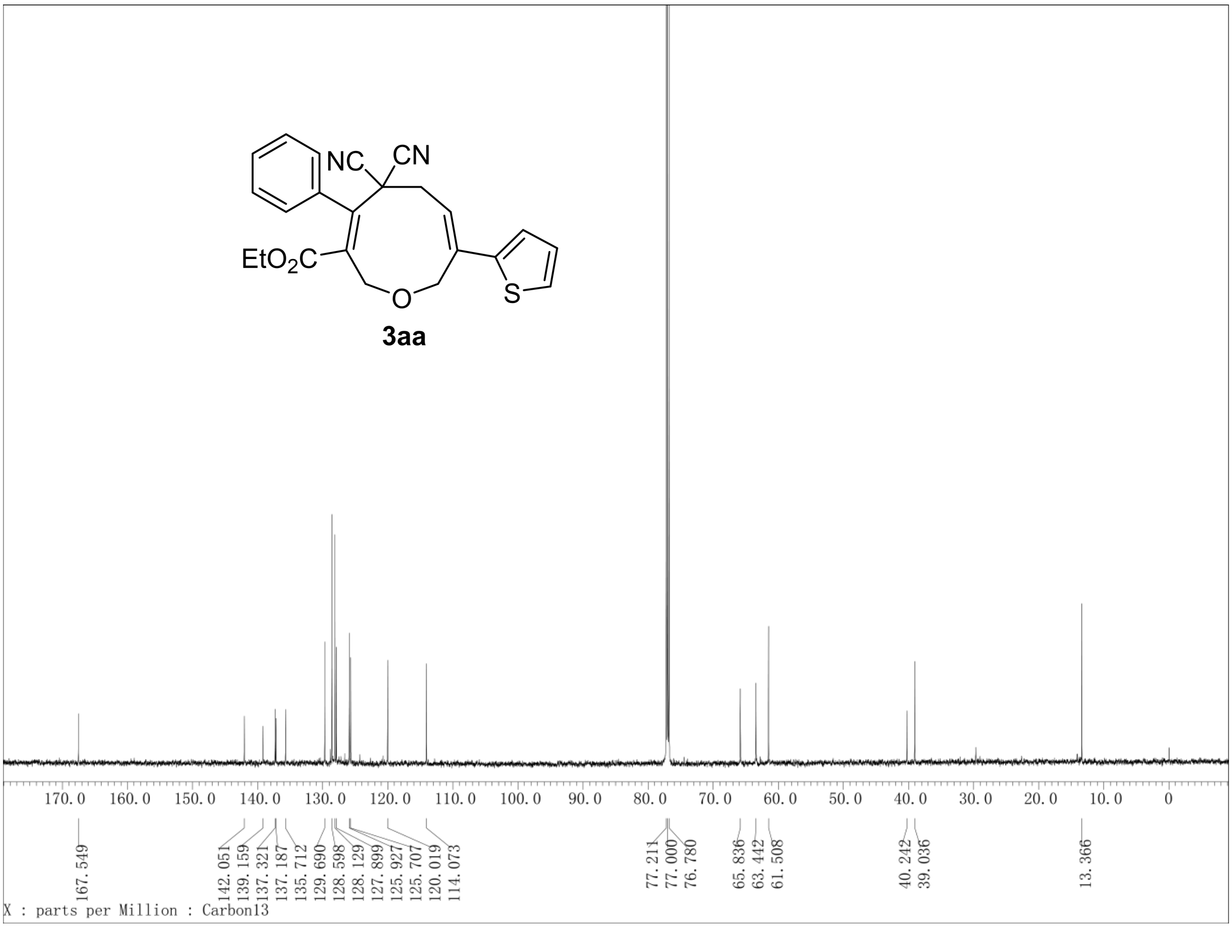
NC
CN
EtO2C
O
S
3aa
170.0 160.0 150.0 140.0 130.0 120.0 110.0 100.0 90.0 80.0 70.0 60.0 50.0 40.0 30.0 20.0 10.0 0
167.549
142.051 139.159 137.321 137.187 135.712 129.690 128.598 128.129 127.899 125.927 125.707 120.019 114.073
77.211 77.000 76.780
65.836 63.442 61.508
40.242 39.036
13.366
X : parts per Million : Carbon13

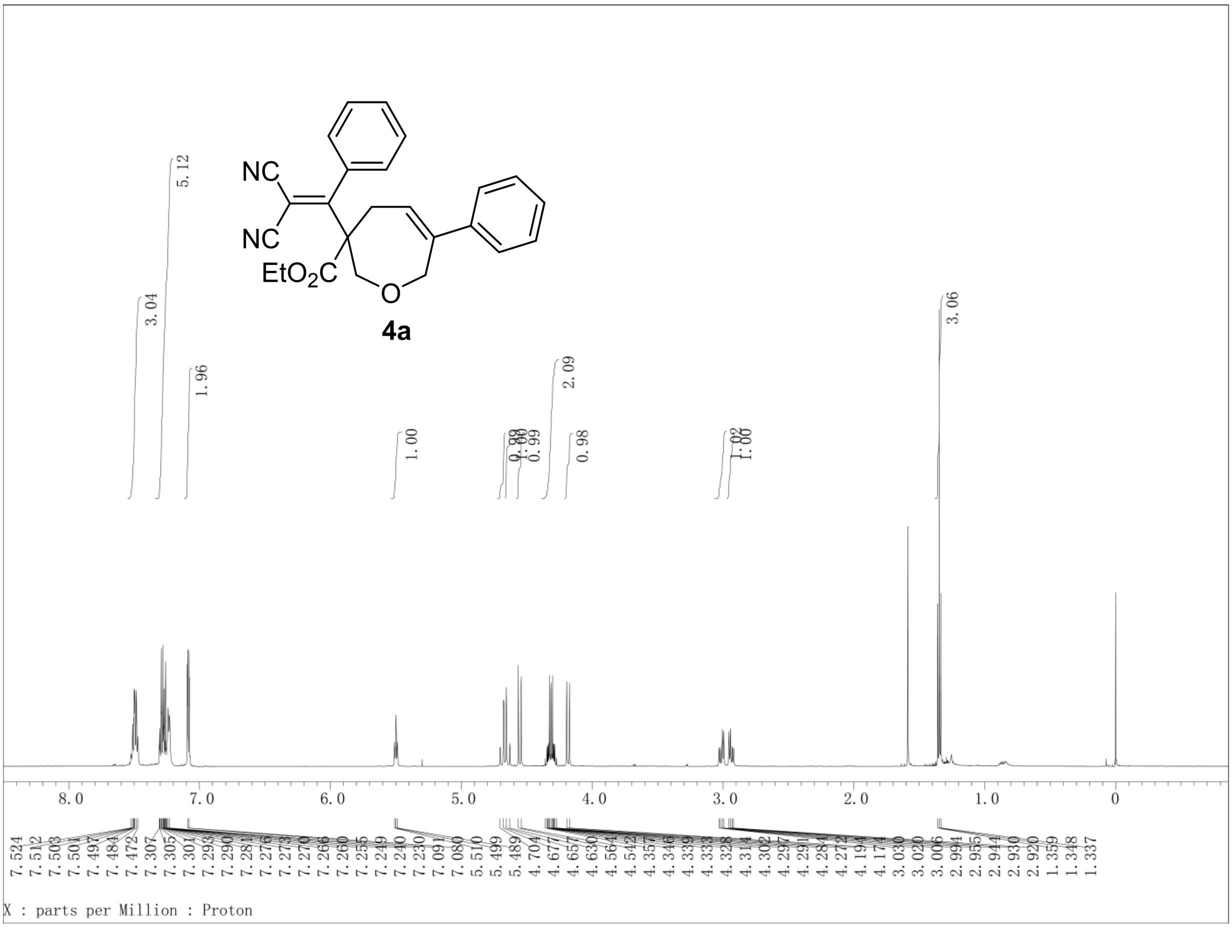

NC
NC
EtO2C
O
4a
3.04
5.12
1.96
1.00
0.99
2.09
0.98
3.06
8.0 7.0 6.0 5.0 4.0 3.0 2.0 1.0 0
7.524 7.512 7.503 7.501 7.497 7.484 7.472 7.307 7.305 7.301 7.293 7.290 7.281 7.276 7.273 7.270 7.266 7.260 7.255 7.249 7.240 7.230 7.091 7.080 5.510 5.499 5.489 4.704 4.677 4.657 4.630 4.564 4.542 4.357 4.346 4.339 4.333 4.328 4.314 4.302 4.297 4.291 4.284 4.272 4.194 4.174 3.030 3.020 3.006 2.994 2.955 2.944 2.930 2.920 1.359 1.348 1.337
X : parts per Million : Proton

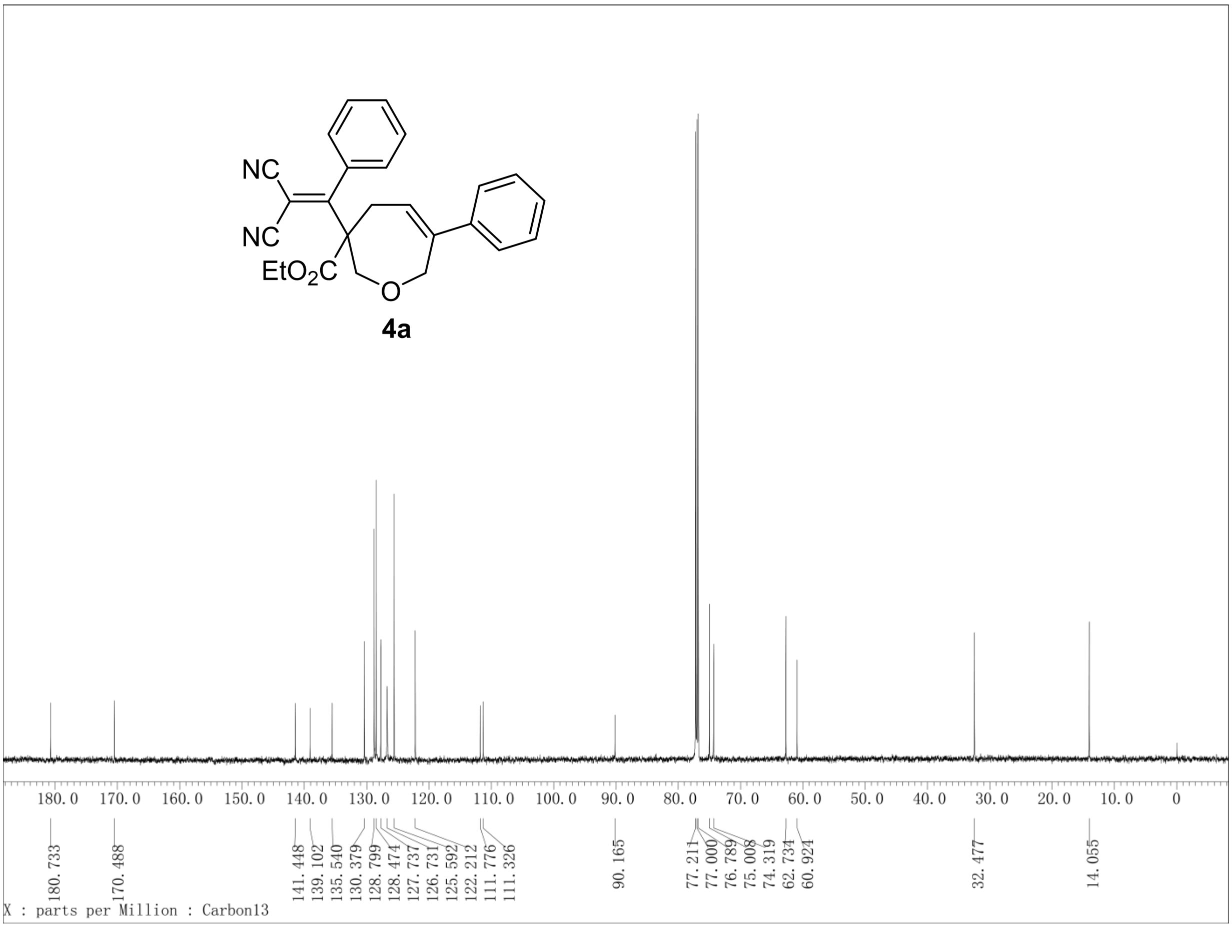

NC
NC
EtO2C
O
4a
180.0 170.0 160.0 150.0 140.0 130.0 120.0 110.0 100.0 90.0 80.0 70.0 60.0 50.0 40.0 30.0 20.0 10.0 0
180.733 170.488 141.448 139.102 135.540 130.379 128.799 128.474 127.737 126.731 125.592 122.212 111.776 111.326 90.165 77.211 77.000 76.789 75.008 74.319 62.734 60.921 32.477 14.055
X : parts per Million : Carbon13

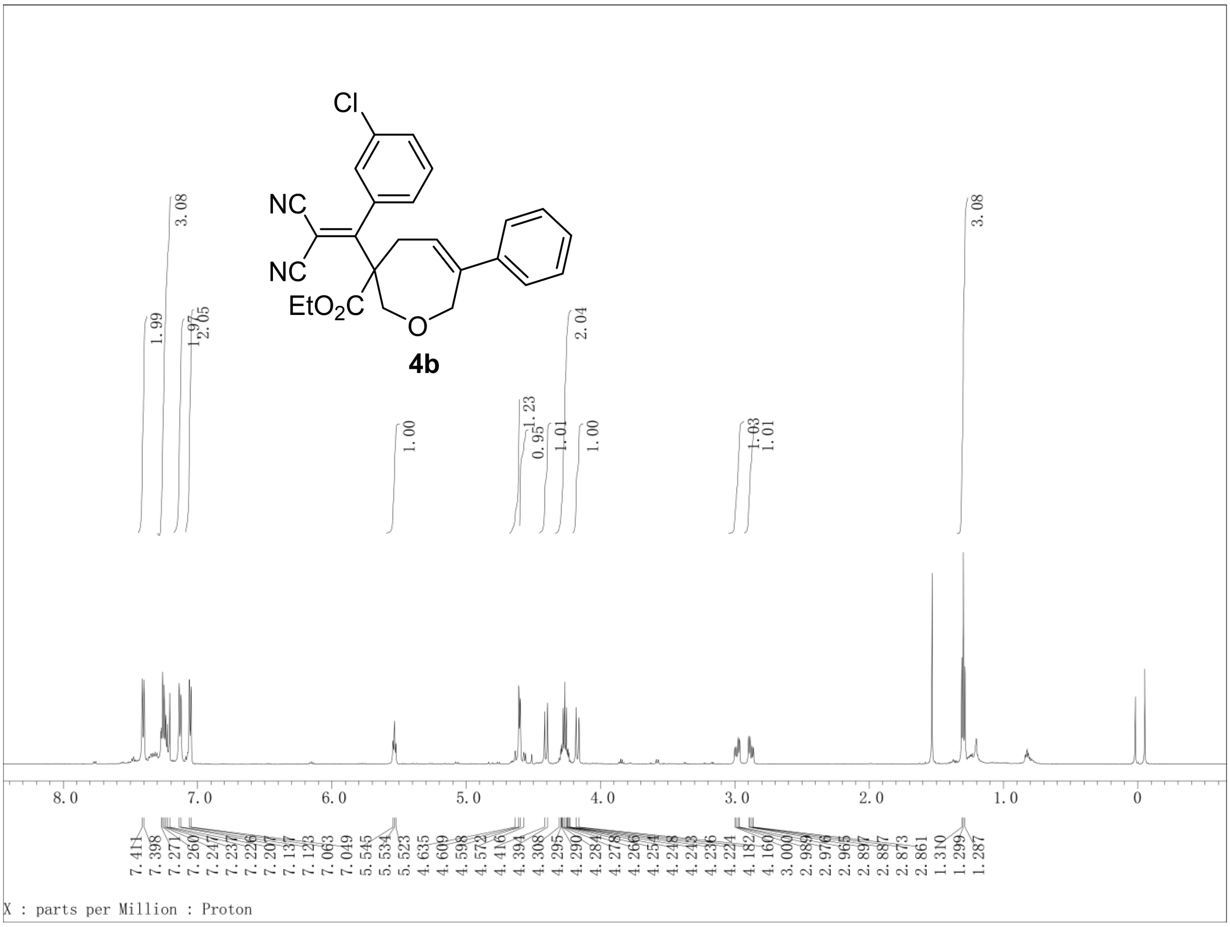
Cl
NC
NC
EtO2C
O
4b
1.99
3.08
1.97
2.05
1.00
1.23
0.95
1.01
2.04
1.00
1.03
1.01
3.08
8.0 7.0 6.0 5.0 4.0 3.0 2.0 1.0 0
7.411 7.398 7.271 7.260 7.247 7.237 7.226 7.207 7.137 7.123 7.063 7.049 5.545 5.534 5.523 4.635 4.609 4.598 4.572 4.416 4.394 4.308 4.295 4.290 4.284 4.278 4.266 4.254 4.248 4.243 4.236 4.224 4.182 4.160 3.000 2.989 2.976 2.965 2.897 2.887 2.873 2.861 1.310 1.299 1.287
X : parts per Million : Proton

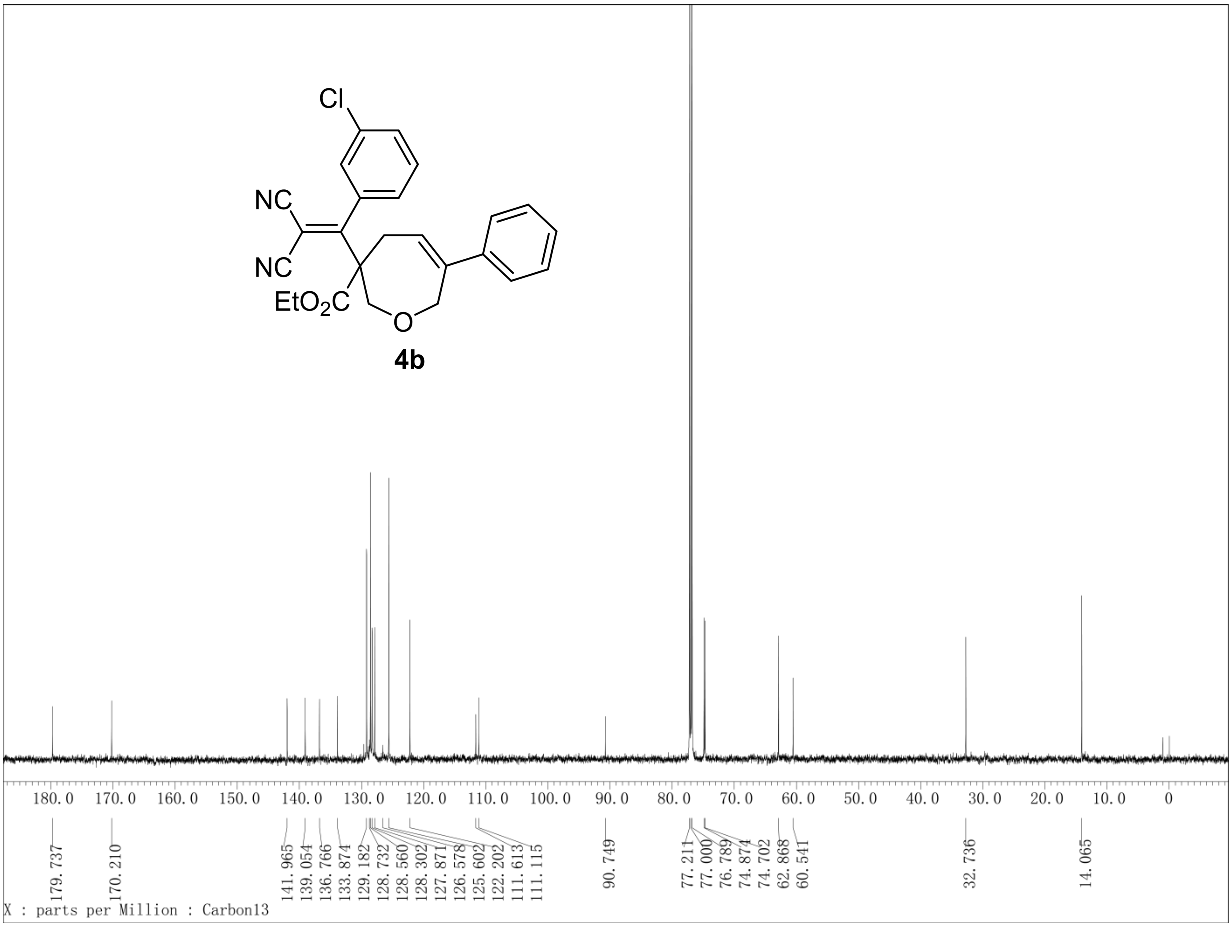
Cl
NC
NC
EtO2C
O
4b
180.0 170.0 160.0 150.0 140.0 130.0 120.0 110.0 100.0 90.0 80.0 70.0 60.0 50.0 40.0 30.0 20.0 10.0 0
179.737 170.210 141.965 139.054 136.766 133.874 129.182 128.732 128.560 128.302 127.871 126.578 125.602 122.202 111.613 111.115 90.749 77.211 77.000 76.789 74.874 74.702 62.868 60.541 32.736 14.065
X : parts per Million : Carbon13

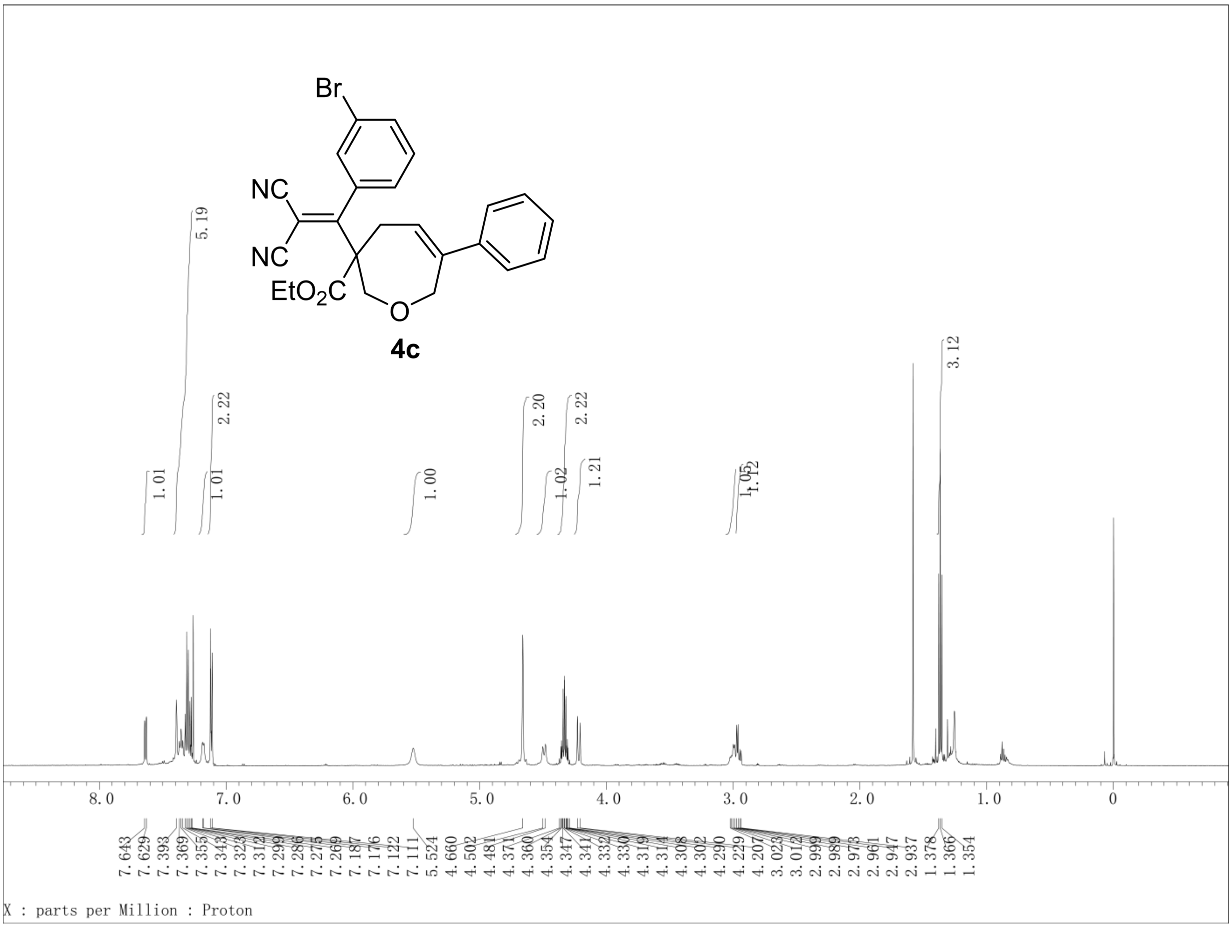
Br
NC
NC
EtO2C
O
4c
X : parts per Million : Proton

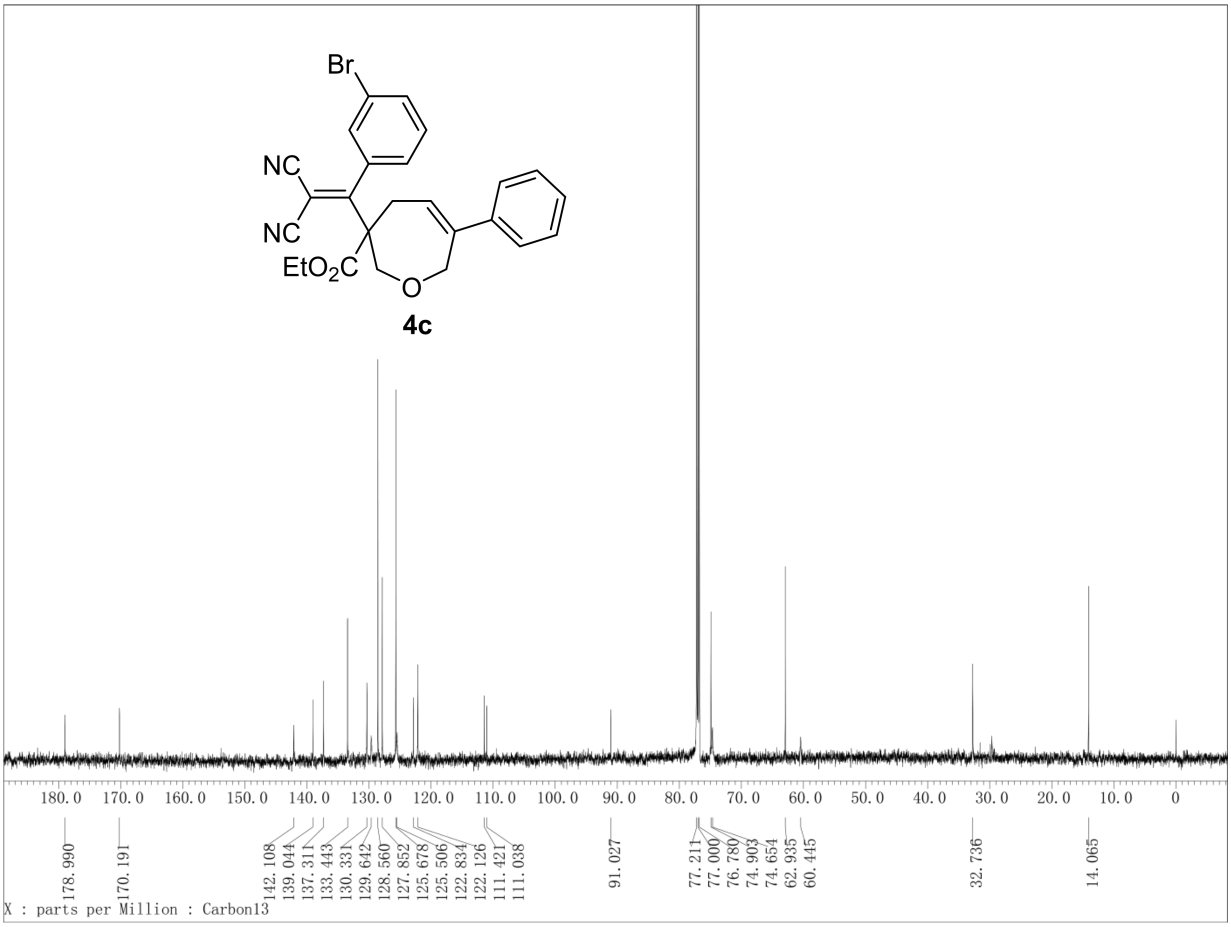
Br
NC
NC
EtO2C
O
4c
X : parts per Million : Carbon13

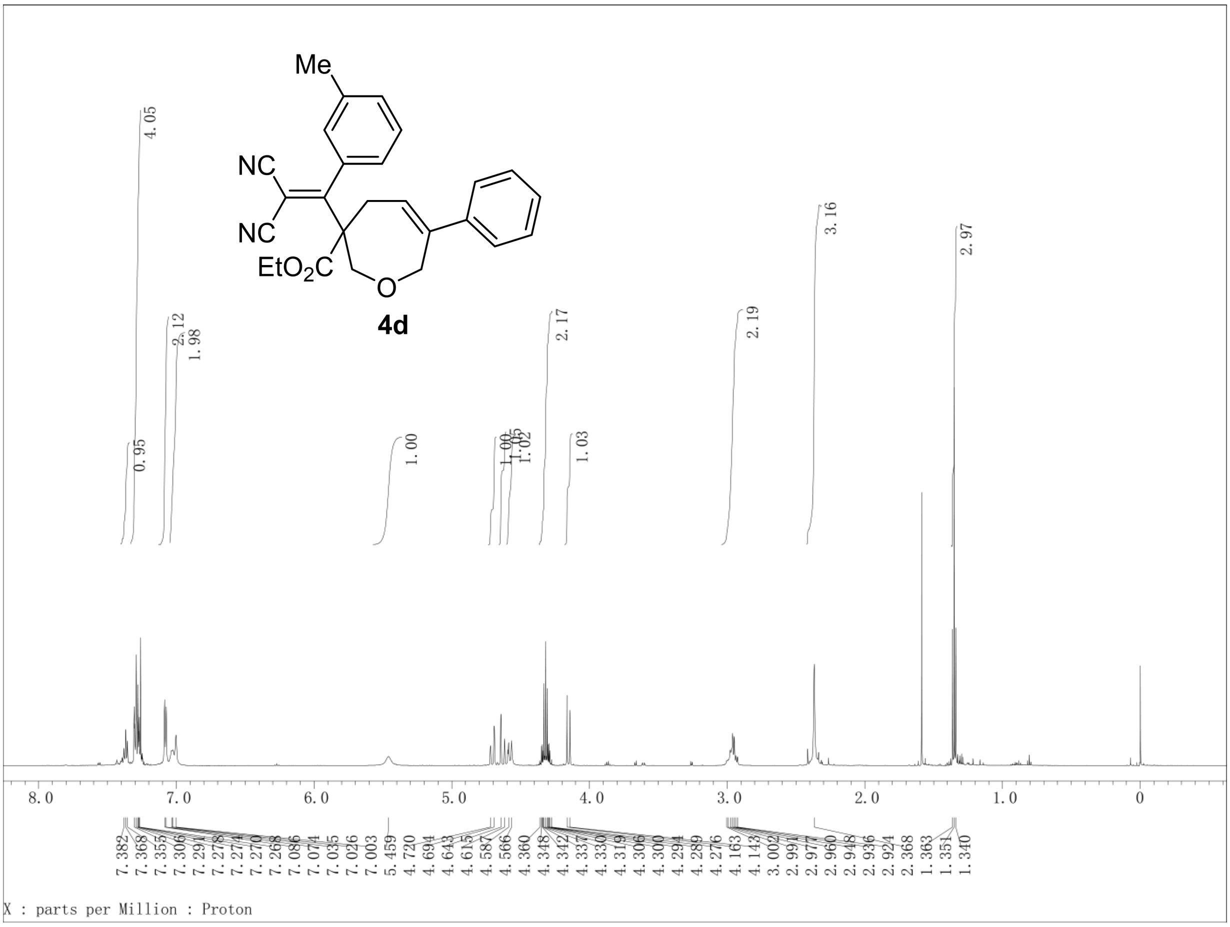
Me
NC
NC
EtO2C
O
4d
4.05
0.95
2.12
1.98
1.00
1.00
1.05
1.02
2.17
1.03
2.19
3.16
2.97
8.0 7.0 6.0 5.0 4.0 3.0 2.0 1.0 0
7.382 7.368 7.355 7.306 7.291 7.278 7.274 7.270 7.268 7.086 7.074 7.035 7.026 7.003 5.459 4.720 4.694 4.643 4.615 4.587 4.566 4.360 4.348 4.342 4.337 4.330 4.319 4.306 4.300 4.294 4.289 4.276 4.163 4.142 3.002 2.991 2.977 2.960 2.948 2.936 2.924 2.368 1.363 1.351 1.340
X : parts per Million : Proton

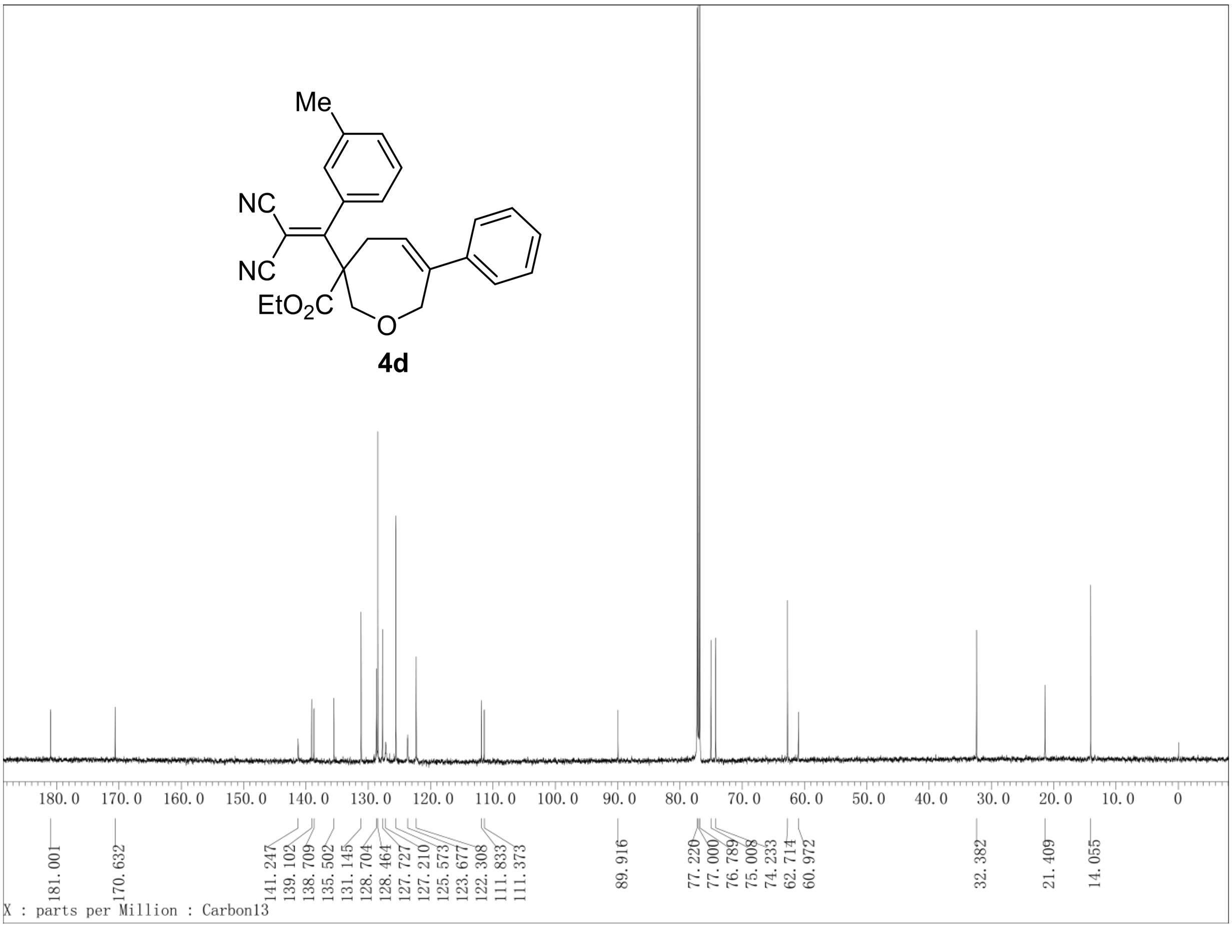
Me
NC
NC
EtO2C
O
4d
180.0 170.0 160.0 150.0 140.0 130.0 120.0 110.0 100.0 90.0 80.0 70.0 60.0 50.0 40.0 30.0 20.0 10.0 0
181.001
170.632
141.247 139.102 138.709 135.502 131.145 128.704 128.464 127.727 127.210 125.573 123.677 122.308 111.833 111.373
89.916
77.220 77.000 76.789 75.008 74.233 62.714 60.972
32.382
21.409
14.055
X : parts per Million : Carbon13

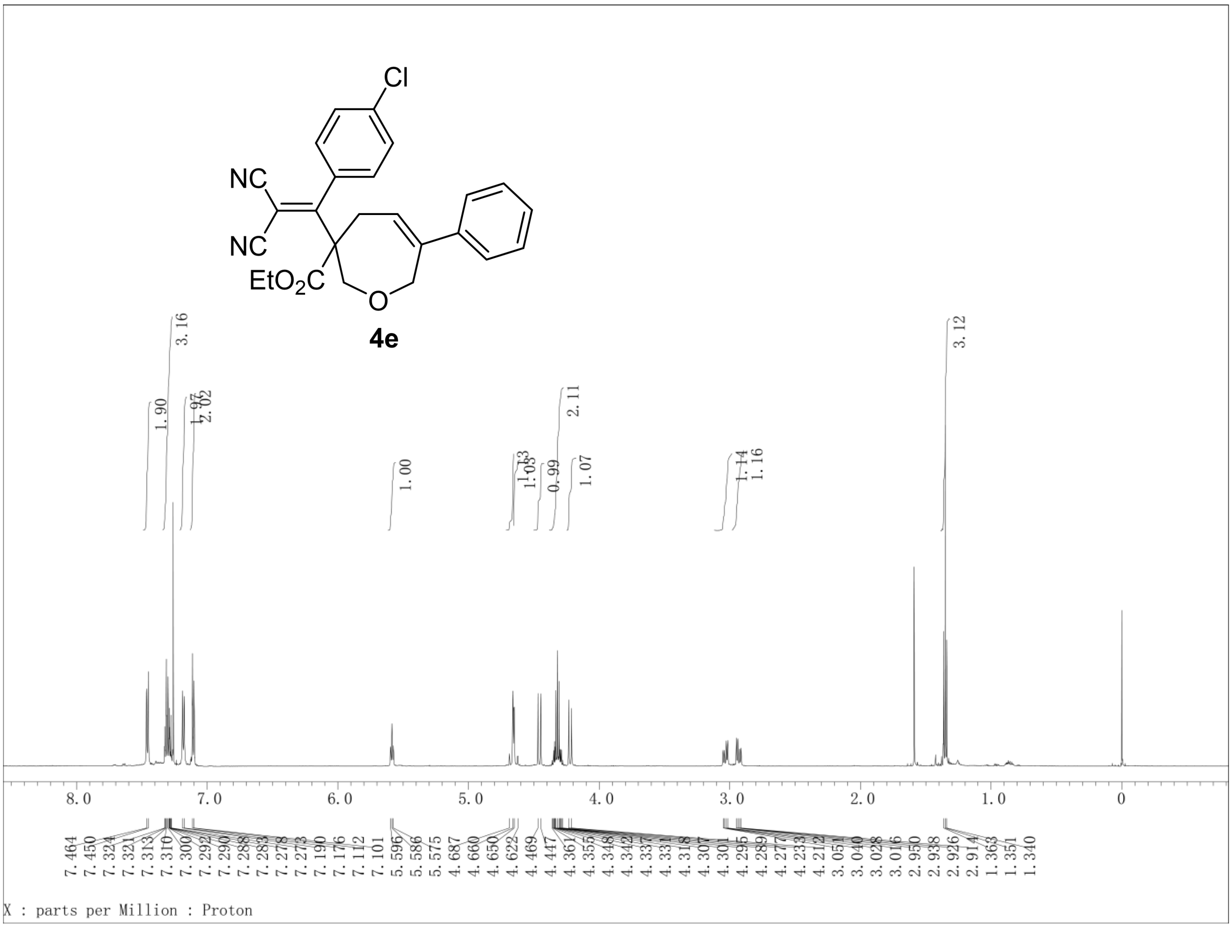

4e
7.464 7.450 7.324 7.321 7.313 7.310 7.300 7.292 7.290 7.288 7.283 7.278 7.273 7.190 7.176 7.112 7.101 5.596 5.586 5.575 4.687 4.660 4.650 4.622 4.469 4.447 4.361 4.355 4.348 4.342 4.337 4.331 4.318 4.307 4.301 4.295 4.289 4.277 4.233 4.212 3.051 3.040 3.028 3.016 2.950 2.938 2.926 2.914 1.363 1.351 1.340
X : parts per Million : Proton

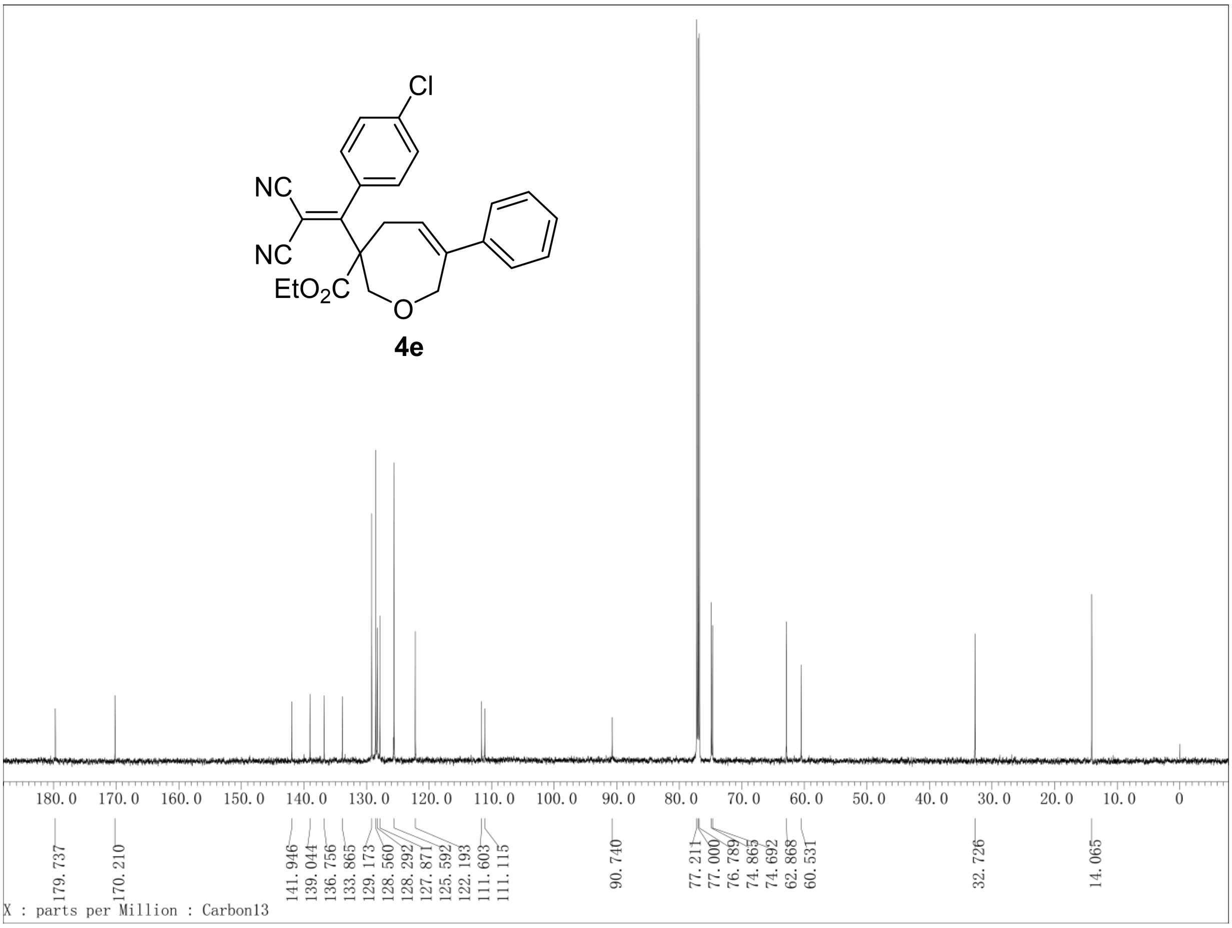

4e
179.737 170.210 141.946 139.044 136.756 133.865 129.173 128.560 128.292 127.871 125.592 122.193 111.603 111.115 90.740 77.211 77.000 76.789 74.865 74.692 62.868 60.531 32.726 14.065
X : parts per Million : Carbon13

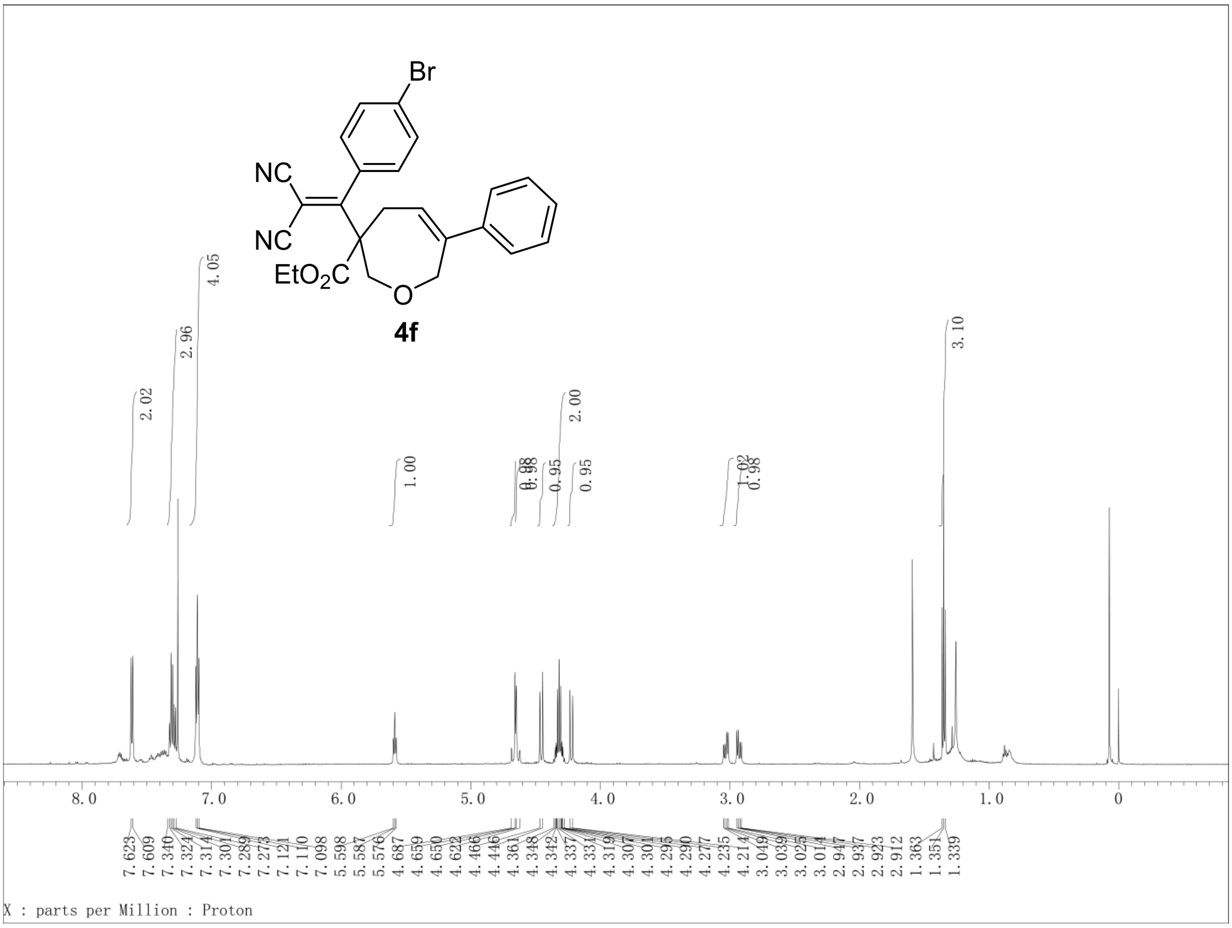
Br
NC
NC
EtO2C
O
4f
2.02
2.96
4.05
1.00
0.98
0.98
0.95
2.00
0.95
1.02
0.98
3.10
8.0 7.0 6.0 5.0 4.0 3.0 2.0 1.0 0
7.623 7.609 7.340 7.324 7.314 7.301 7.289 7.273 7.121 7.110 7.098 5.598 5.587 5.576 4.687 4.659 4.650 4.622 4.466 4.446 4.361 4.348 4.342 4.337 4.331 4.319 4.307 4.301 4.295 4.290 4.277 4.235 4.214 3.049 3.039 3.025 3.014 2.947 2.937 2.923 2.912 1.363 1.351 1.339
X : parts per Million : Proton

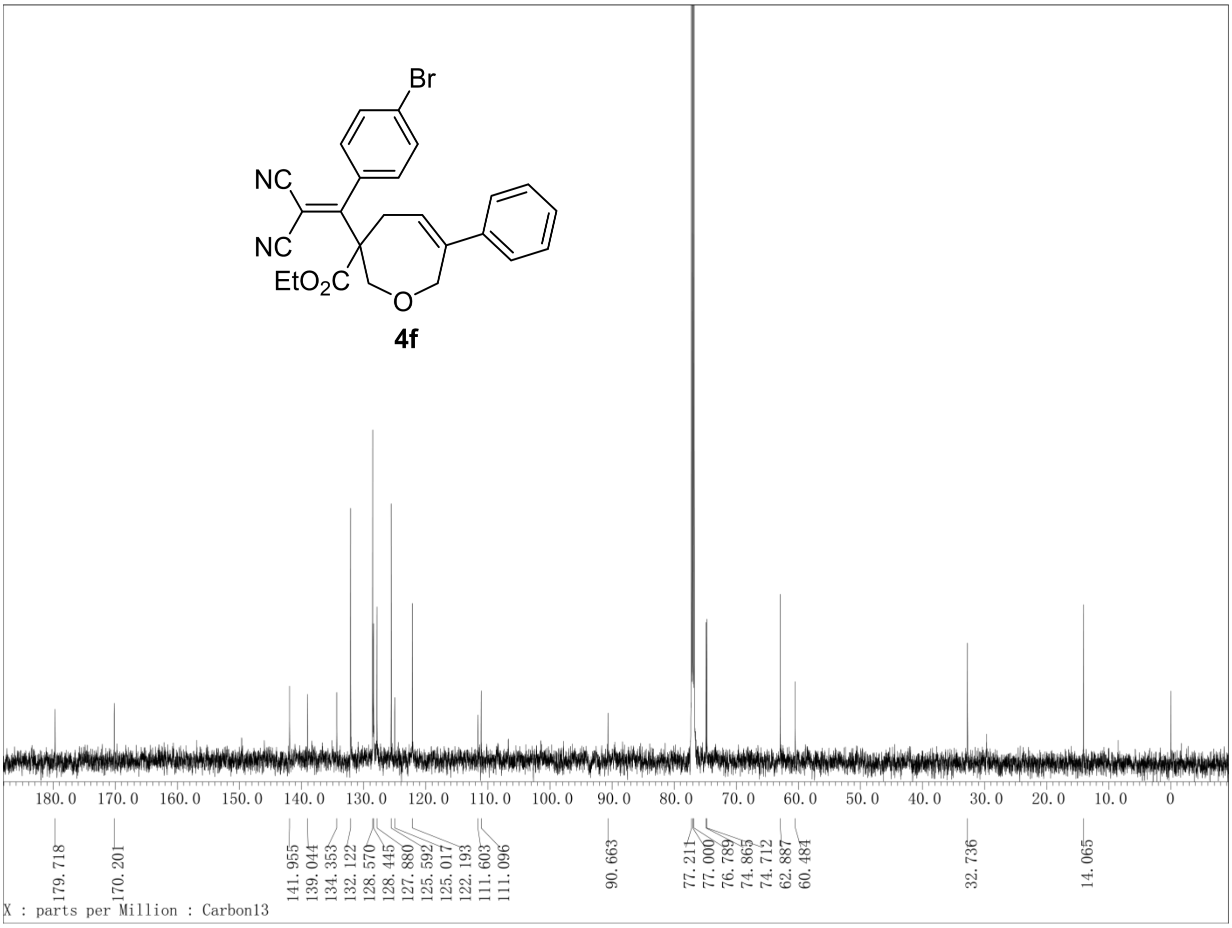
Br
NC
NC
EtO2C
O
4f
180.0 170.0 160.0 150.0 140.0 130.0 120.0 110.0 100.0 90.0 80.0 70.0 60.0 50.0 40.0 30.0 20.0 10.0 0
179.718 170.201 141.955 139.044 134.353 132.122 128.570 128.445 127.880 125.592 125.017 122.193 111.603 111.096 90.663 77.211 77.000 76.789 74.865 74.712 62.887 60.481 32.736 14.065
X : parts per Million : Carbon13

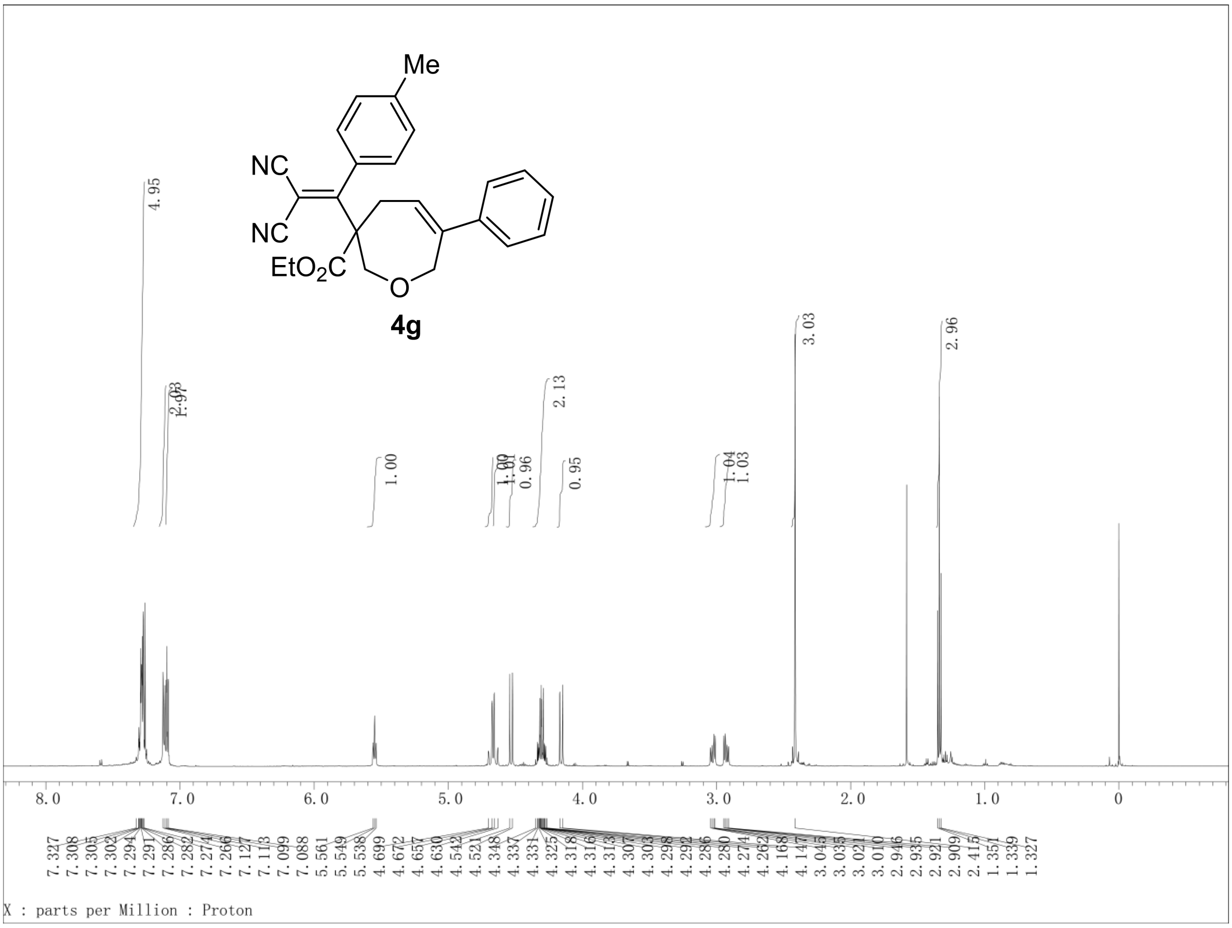
Me
NC
NC
EtO2C
O
4g
4.95
2.03
1.97
1.00
1.00
1.01
0.96
2.13
0.95
1.04
1.03
3.03
2.96
8.0 7.0 6.0 5.0 4.0 3.0 2.0 1.0 0
7.327 7.308 7.305 7.302 7.294 7.291 7.286 7.282 7.274 7.266 7.127 7.113 7.099 7.088 5.561 5.549 5.538 4.699 4.672 4.657 4.630 4.542 4.521 4.348 4.337 4.331 4.325 4.318 4.316 4.313 4.307 4.303 4.298 4.292 4.286 4.280 4.274 4.262 4.168 4.147 3.045 3.035 3.021 3.010 2.946 2.935 2.921 2.909 2.415 1.351 1.339 1.327
X : parts per Million : Proton

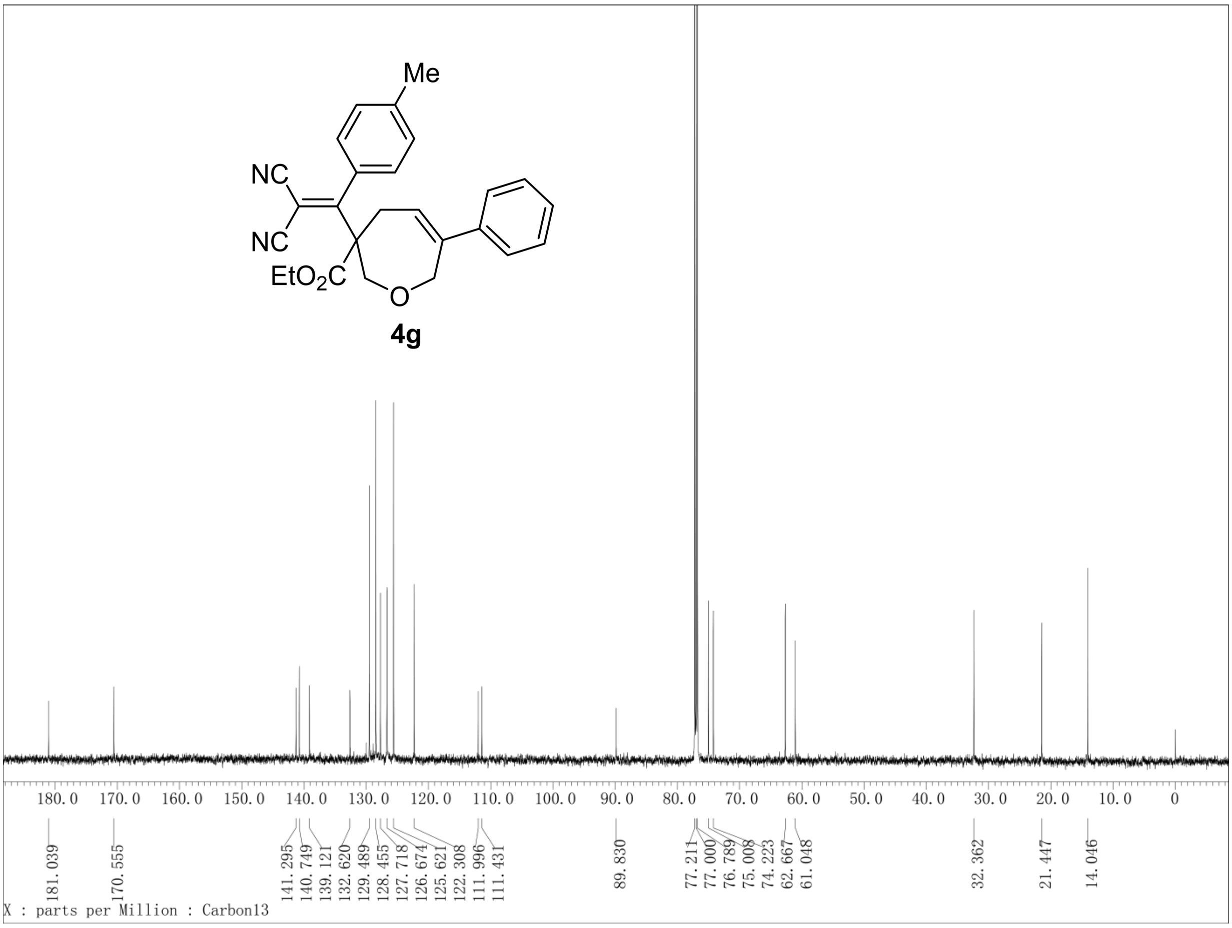
Me
NC
NC
EtO2C
O
4g
180.0 170.0 160.0 150.0 140.0 130.0 120.0 110.0 100.0 90.0 80.0 70.0 60.0 50.0 40.0 30.0 20.0 10.0 0
181.039 170.555 141.295 140.749 139.121 132.620 129.489 128.455 127.718 126.674 125.621 122.308 111.996 111.431 89.830 77.211 77.000 76.789 75.008 74.223 62.667 61.048 32.362 21.447 14.046
X : parts per Million : Carbon13

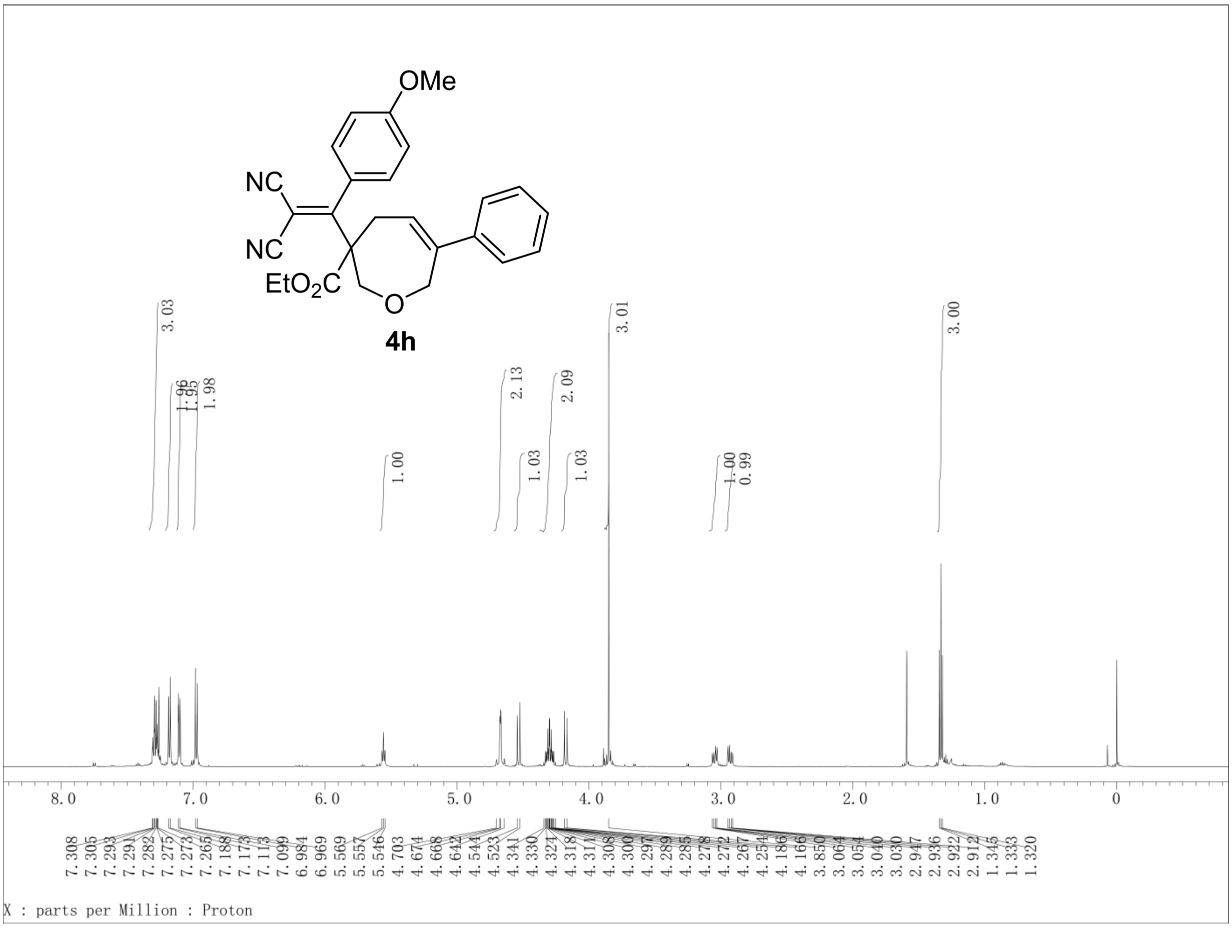
OMe
NC
NC
EtO2C
O
4h
3.03
1.96
1.95
1.98
1.00
2.13
1.03
2.09
1.03
3.01
1.00
0.99
3.00
8.0 7.0 6.0 5.0 4.0 3.0 2.0 1.0 0
7.308 7.305 7.293 7.291 7.282 7.275 7.273 7.265 7.188 7.173 7.113 7.099 6.984 6.969 5.569 5.557 5.546 4.703 4.674 4.668 4.642 4.544 4.523 4.341 4.330 4.327 4.318 4.317 4.308 4.300 4.297 4.289 4.285 4.278 4.272 4.267 4.254 4.186 4.166 3.850 3.064 3.054 3.040 3.030 2.947 2.936 2.922 2.912 1.345 1.333 1.320
X : parts per Million : Proton

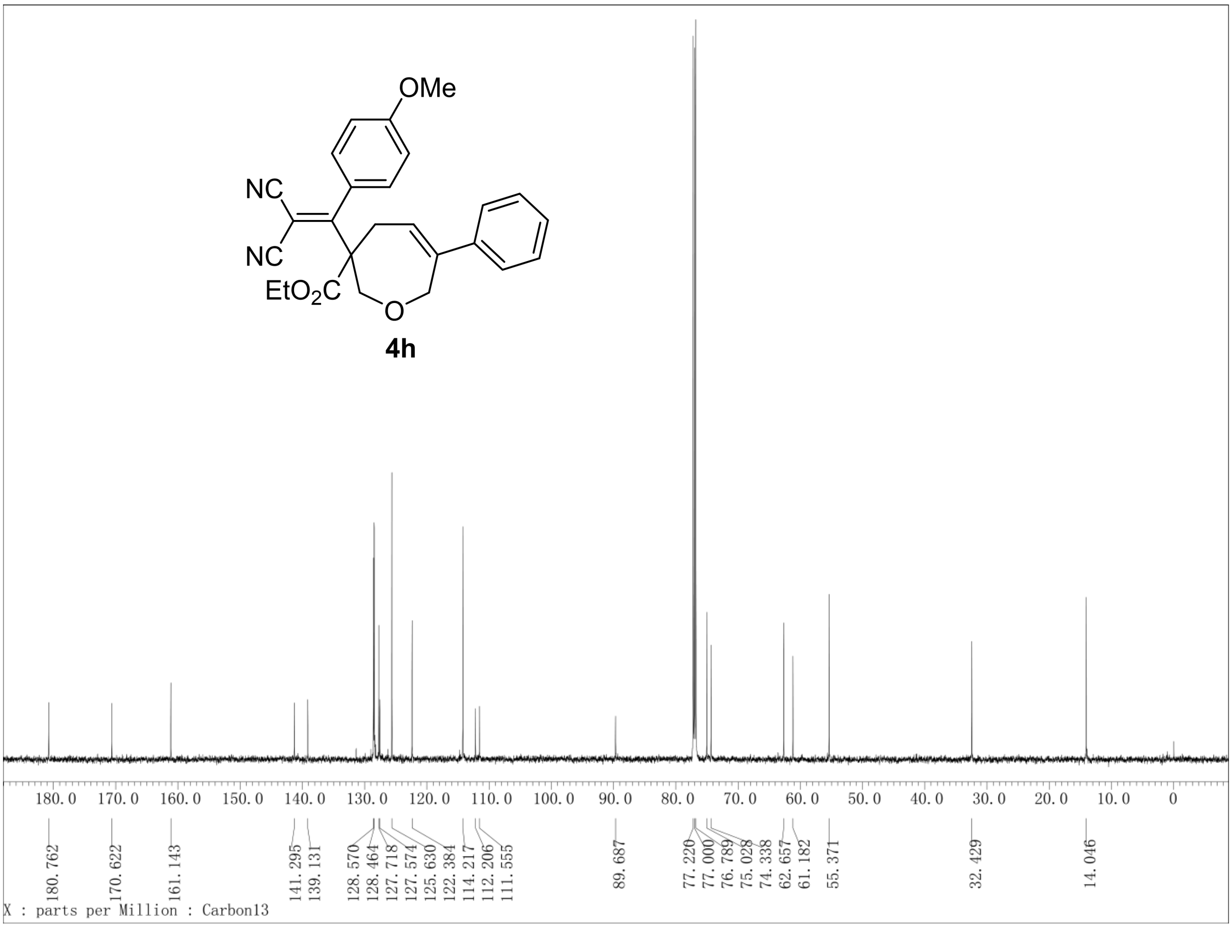
OMe
NC
NC
EtO2C
O
4h
180.0 170.0 160.0 150.0 140.0 130.0 120.0 110.0 100.0 90.0 80.0 70.0 60.0 50.0 40.0 30.0 20.0 10.0 0
180.762 170.622 161.143 141.295 139.131 128.570 128.464 127.718 127.574 125.630 122.384 114.217 112.206 111.555 89.687 77.220 77.000 76.789 75.028 74.338 62.657 61.182 55.371 32.429 14.046
X : parts per Million : Carbon13

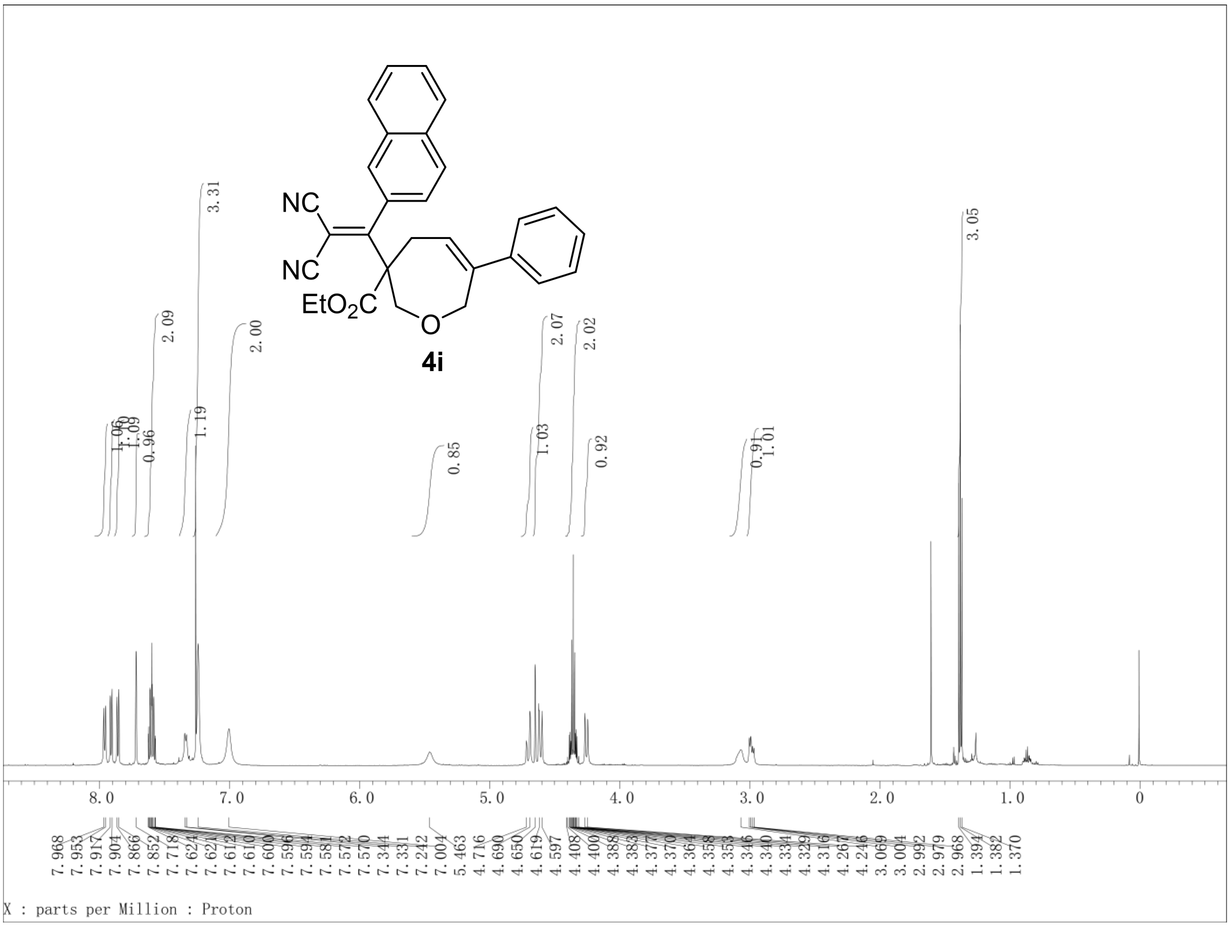

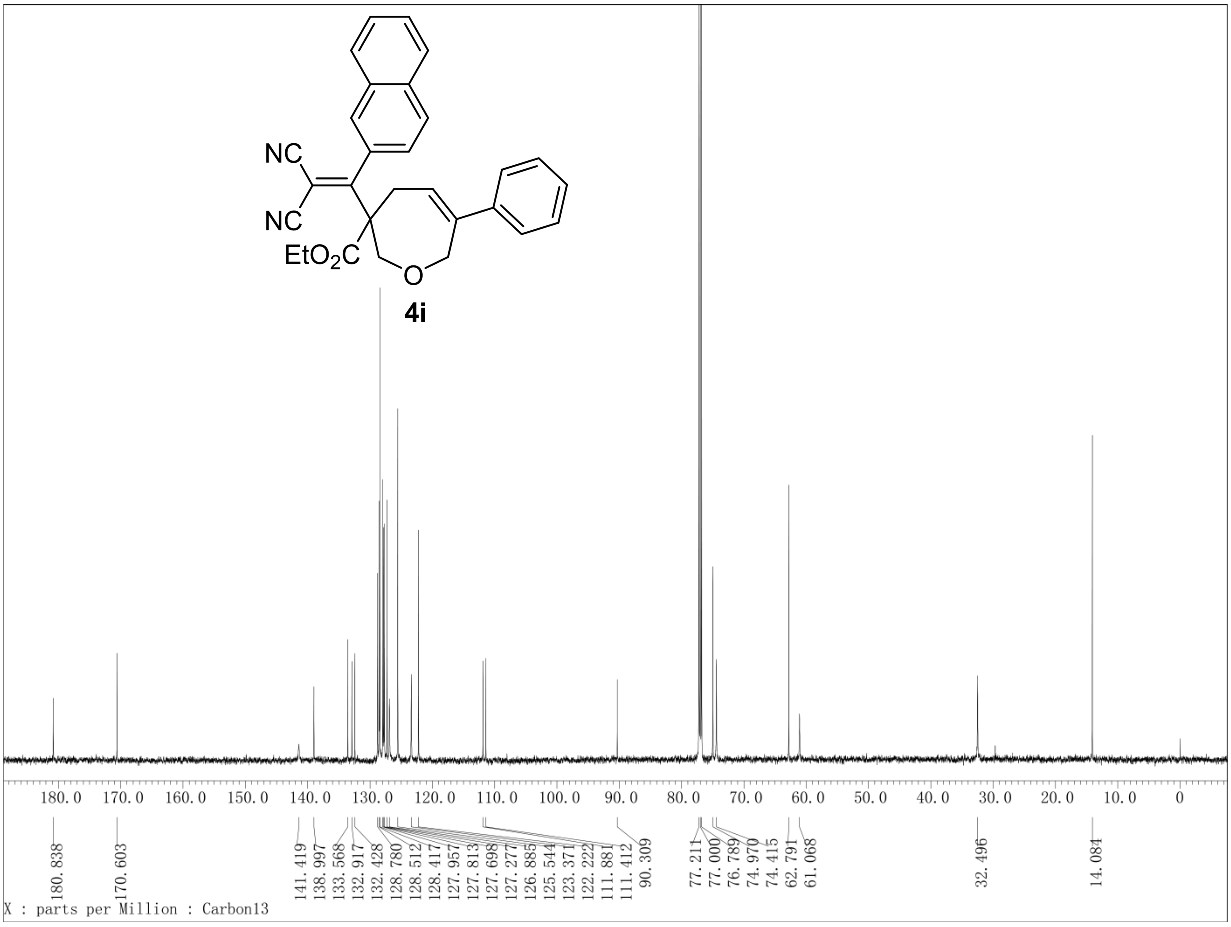

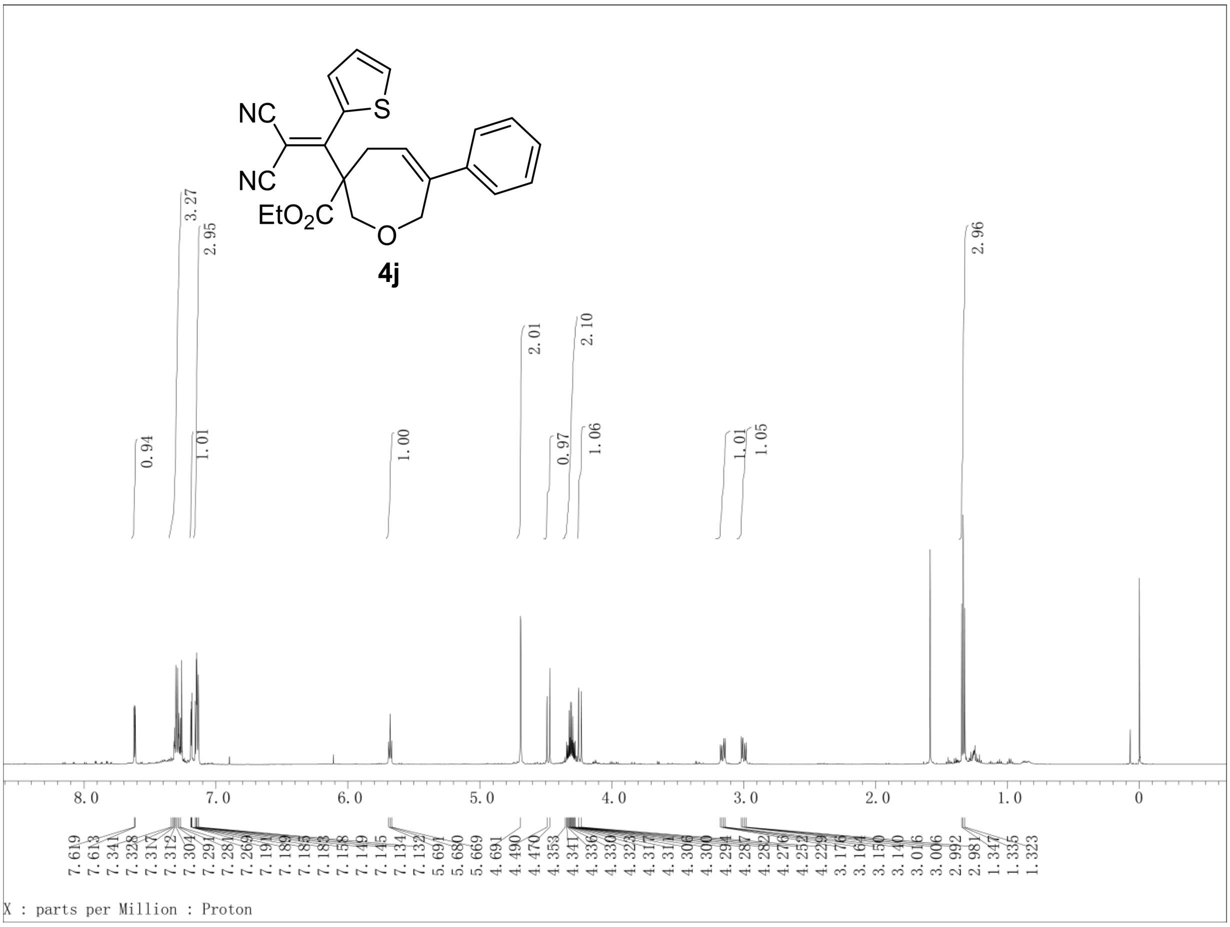
4j
X : parts per Million : Proton

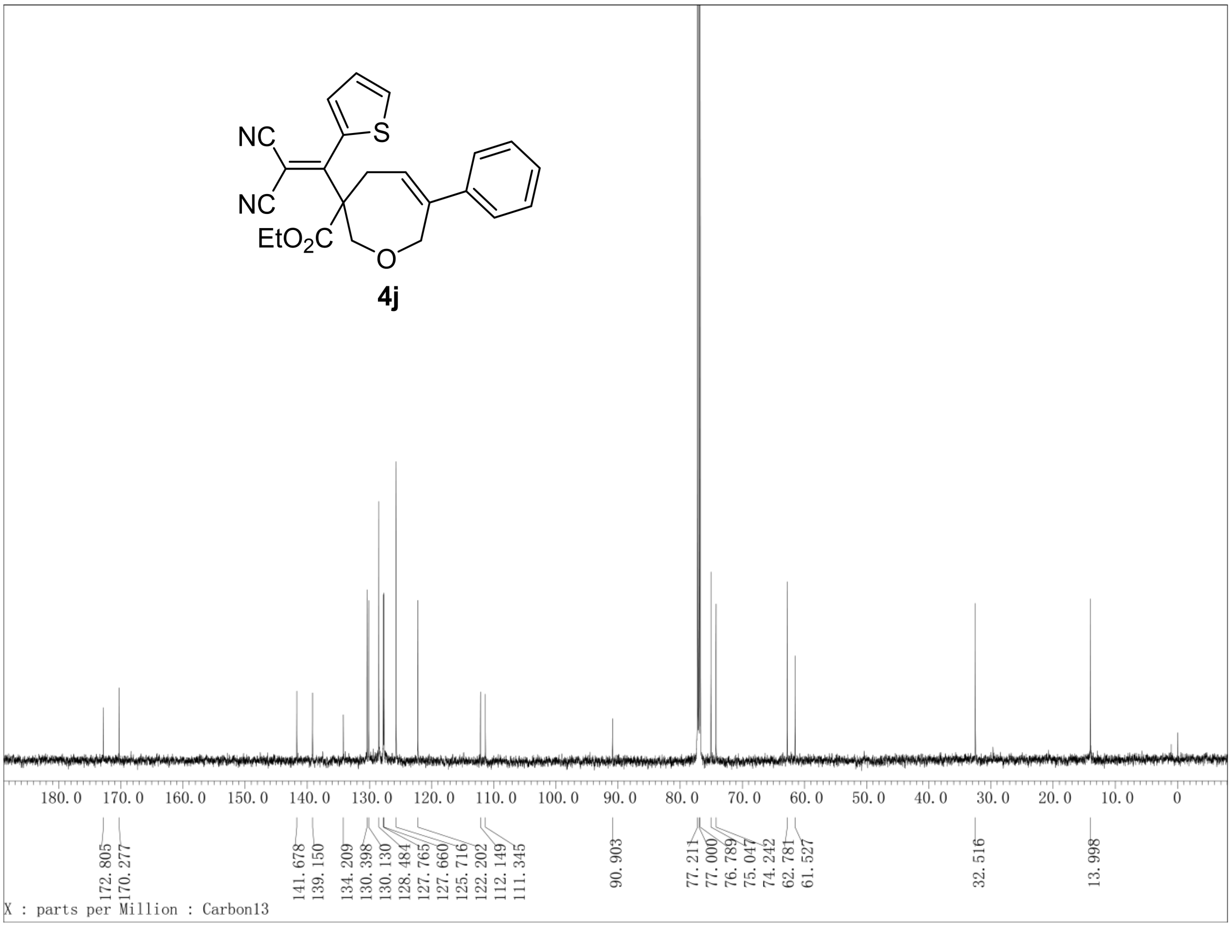
4j
X : parts per Million : Carbon13

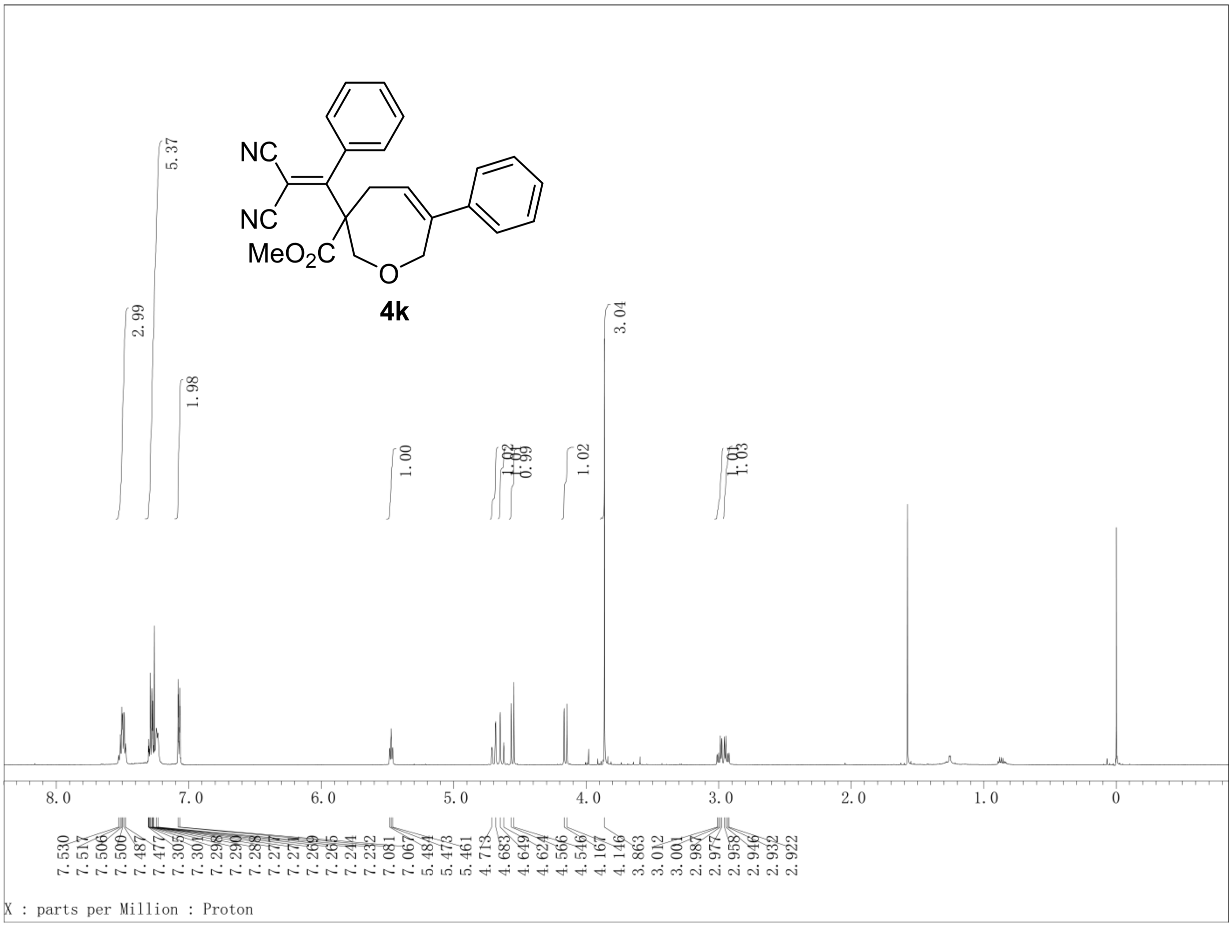
NC
NC
MeO2C
O
4k
2.99
5.37
1.98
1.00
0.99
1.02
3.04
1.01
1.03
8.0
7.0
6.0
5.0
4.0
3.0
2.0
1.0
0
7.530 7.517 7.506 7.500 7.487 7.477 7.305 7.301 7.298 7.290 7.288 7.27 7.271 7.269 7.265 7.244 7.232 7.081 7.067 5.484 5.473 5.461 4.713 4.683 4.649 4.624 4.566 4.546 4.167 4.146 3.863 3.012 3.001 2.987 2.977 2.958 2.946 2.932 2.922
X : parts per Million : Proton

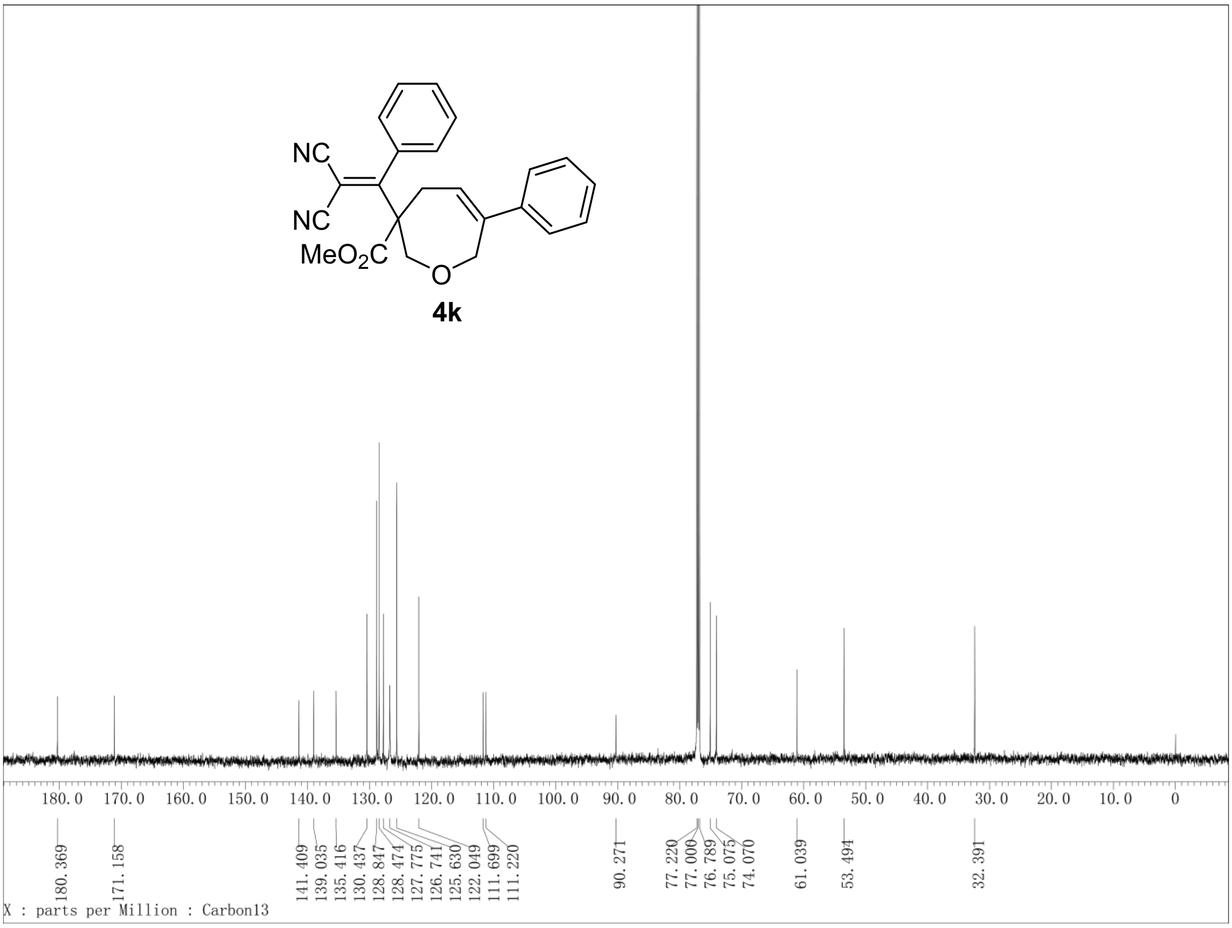
NC
NC
MeO2C
O
4k
180.0
170.0
160.0
150.0
140.0
130.0
120.0
110.0
100.0
90.0
80.0
70.0
60.0
50.0
40.0
30.0
20.0
10.0
0
180.369 171.158 141.409 139.035 135.416 130.437 128.847 128.474 127.775 126.741 125.630 122.049 111.699 111.220 90.271 77.220 77.000 76.789 75.075 74.070 61.039 53.494 32.391
X : parts per Million : Carbon13

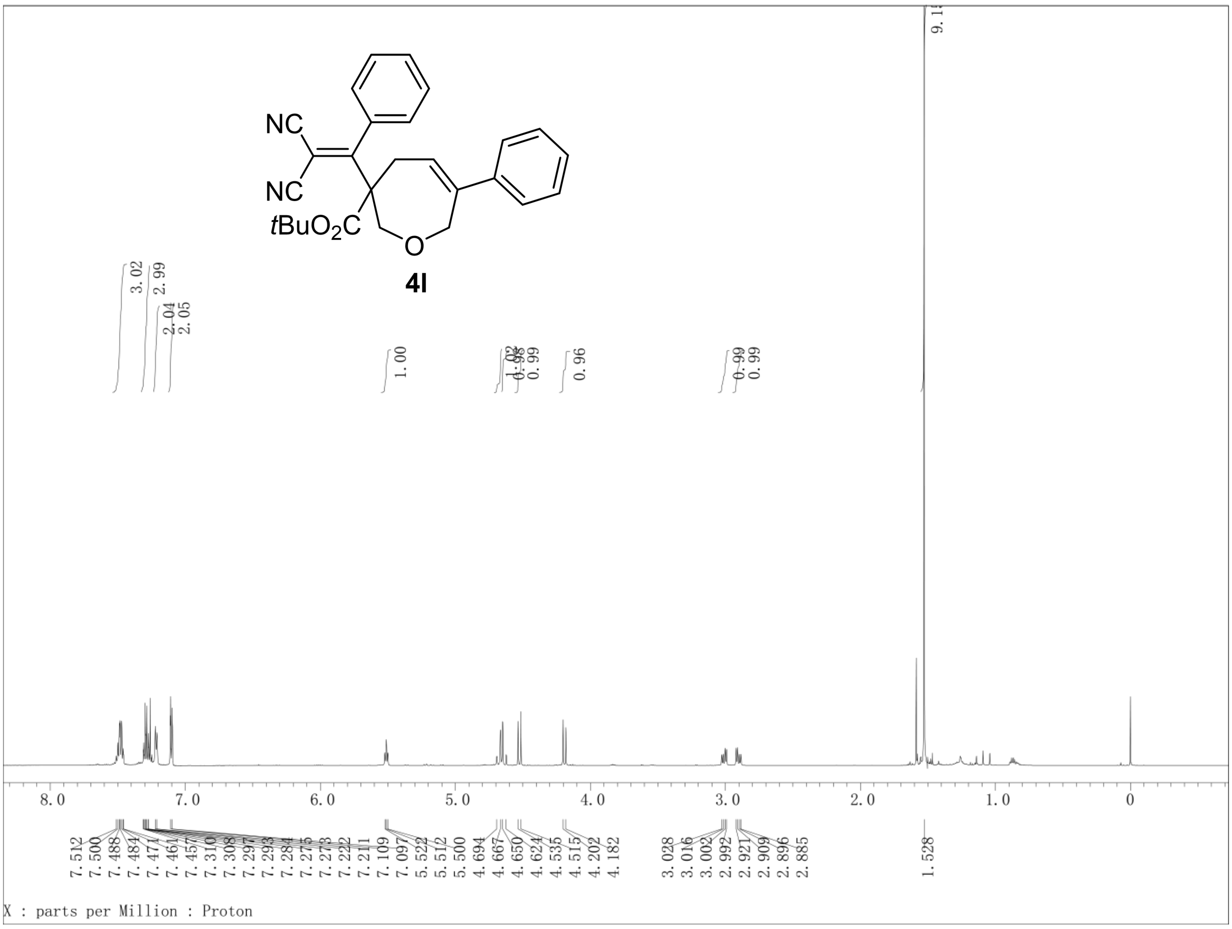
NC
NC
tBuO2C
O
4l
3.02
2.99
2.04
2.05
1.00
0.99
0.96
0.99
0.99
8.0 7.0 6.0 5.0 4.0 3.0 2.0 1.0 0
7.512 7.500 7.488 7.484 7.471 7.461 7.457 7.310 7.308 7.297 7.293 7.284 7.275 7.273 7.222 7.211 7.109 7.097 5.522 5.512 5.500 4.694 4.667 4.650 4.624 4.535 4.515 4.202 4.182
3.028 3.016 3.002 2.992 2.921 2.909 2.896 2.885
1.528
X : parts per Million : Proton

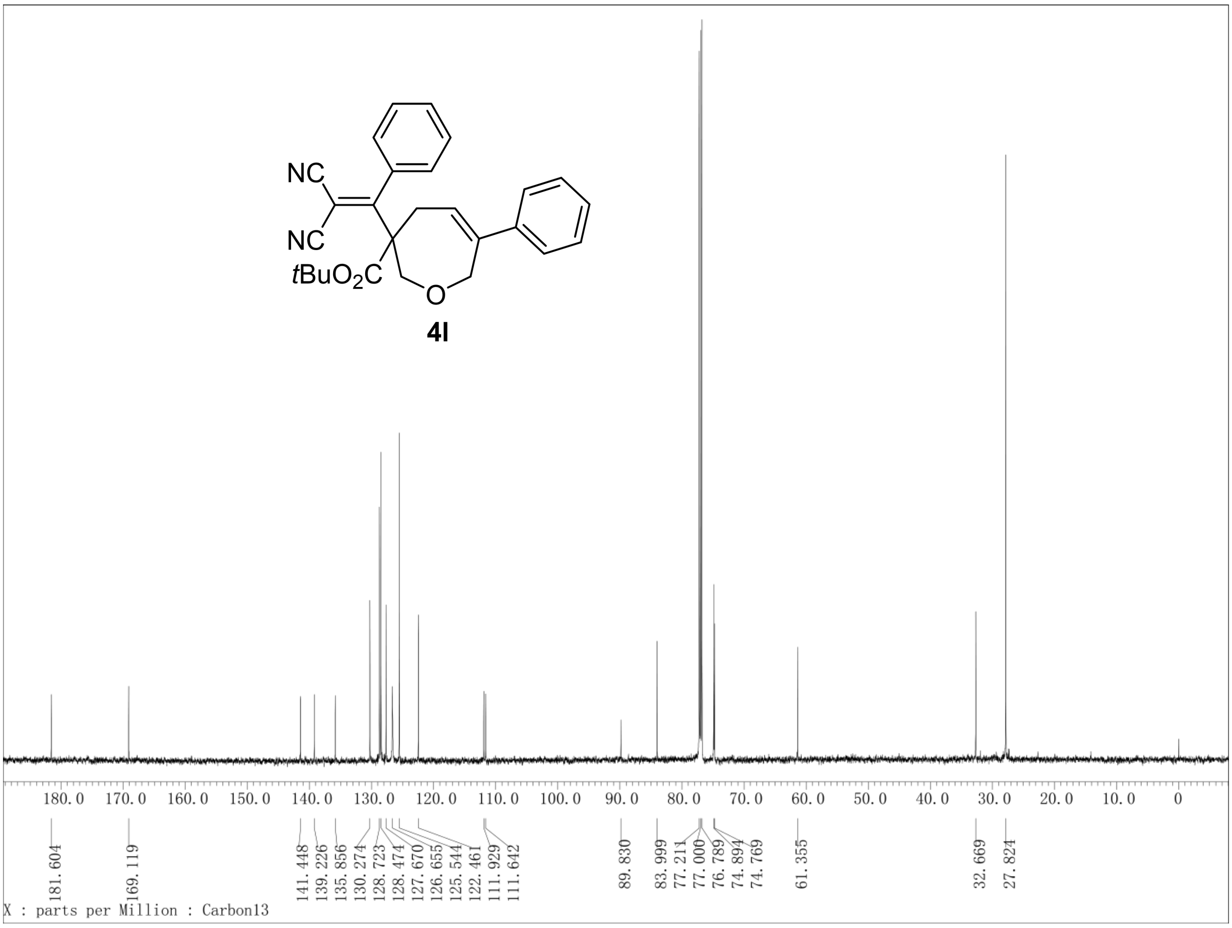
NC
NC
tBuO2C
O
4l
180.0 170.0 160.0 150.0 140.0 130.0 120.0 110.0 100.0 90.0 80.0 70.0 60.0 50.0 40.0 30.0 20.0 10.0 0
181.604
169.119
141.448 139.226 135.856 130.274 128.723 128.474 127.670 126.655 125.544 122.461 111.929 111.642
89.830
83.999 77.211 77.000 76.789 74.894 74.769
61.355
32.669 27.824
X : parts per Million : Carbon13

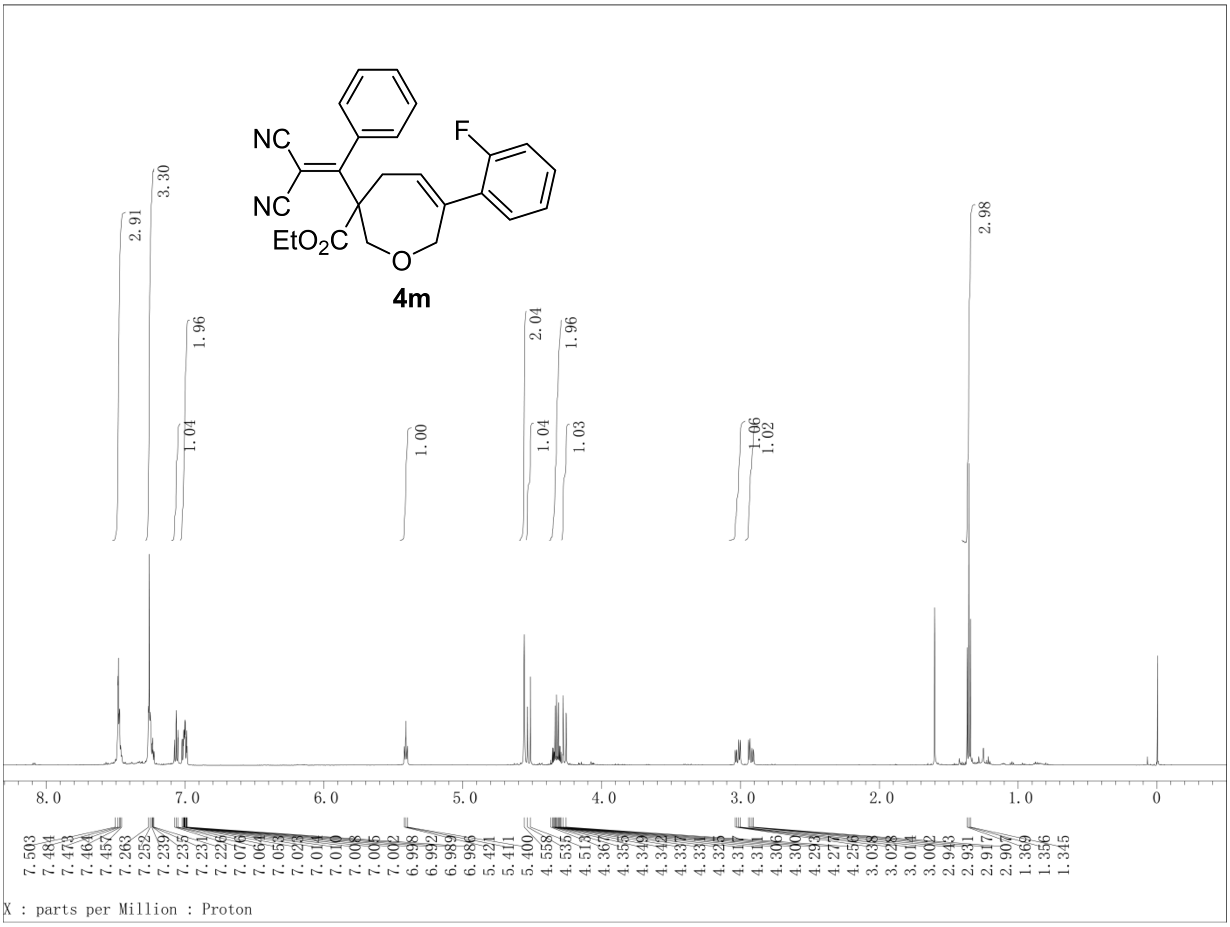
NC
NC
F
EtO2C
O
4m
2.91
3.30
1.96
1.04
1.00
2.04
1.04
1.96
1.03
1.06
1.02
2.98
8.0
7.0
6.0
5.0
4.0
3.0
2.0
1.0
0
7.503
7.484
7.473
7.464
7.457
7.263
7.252
7.239
7.235
7.231
7.226
7.076
7.064
7.053
7.023
7.014
7.010
7.008
7.005
7.002
6.998
6.992
6.989
6.986
5.421
5.411
5.400
4.558
4.535
4.513
4.367
4.355
4.349
4.342
4.337
4.334
4.325
4.317
4.311
4.306
4.300
4.293
4.277
4.256
3.038
3.028
3.014
3.002
2.943
2.931
2.917
2.907
1.369
1.356
1.345
X : parts per Million : Proton

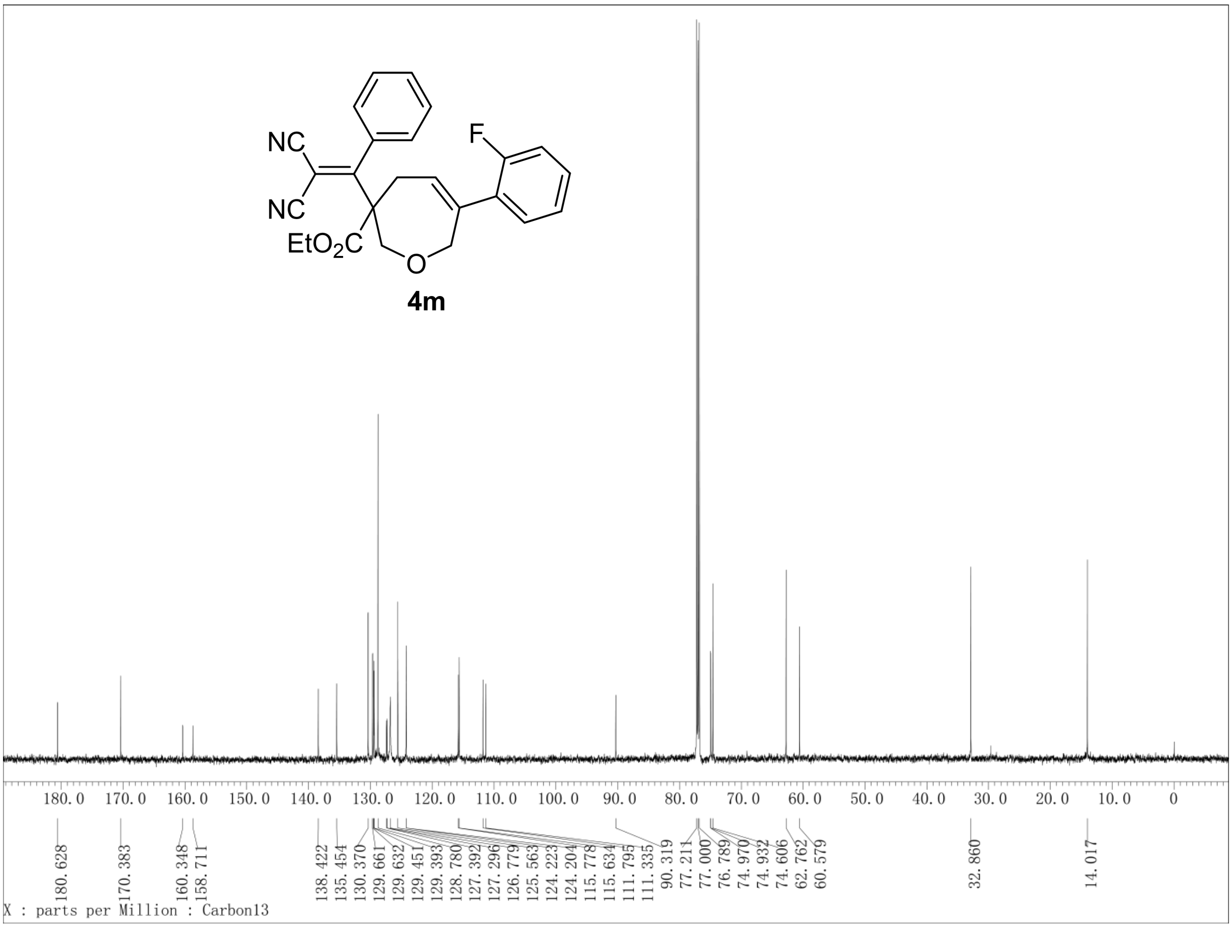
NC
NC
F
EtO2C
O
4m
180.0
170.0
160.0
150.0
140.0
130.0
120.0
110.0
100.0
90.0
80.0
70.0
60.0
50.0
40.0
30.0
20.0
10.0
0
180.628
170.383
160.348
158.711
138.422
135.454
130.370
129.661
129.632
129.451
129.393
128.780
127.392
127.296
126.779
125.563
124.223
124.204
115.778
115.634
111.795
111.335
90.319
77.211
77.000
76.789
74.970
74.932
74.606
62.762
60.579
32.860
14.017
X : parts per Million : Carbon13

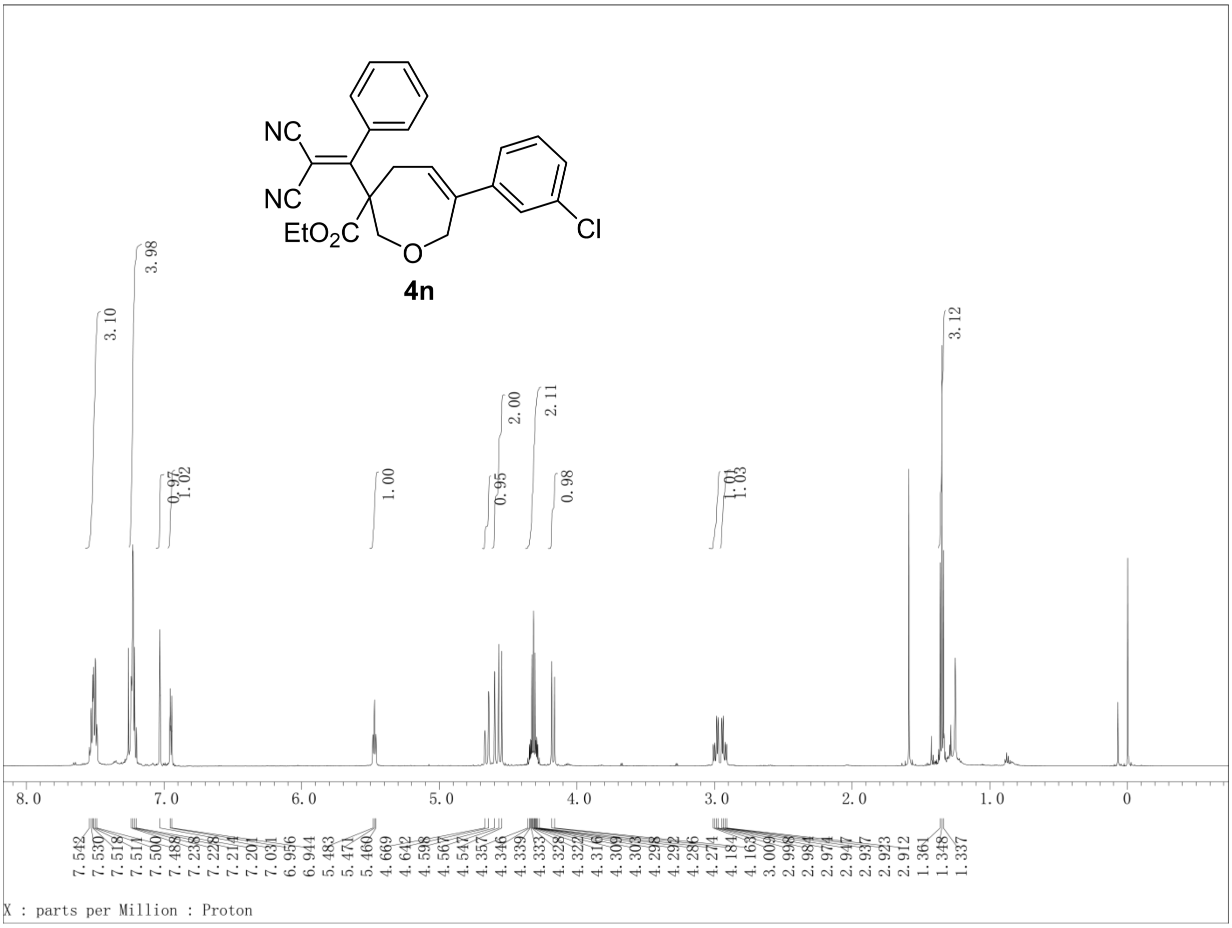
NC
NC
EtO2C
O
Cl
4n
3.10
3.98
0.97
1.02
1.00
0.95
2.00
2.11
0.98
1.01
1.03
3.12
8.0 7.0 6.0 5.0 4.0 3.0 2.0 1.0 0
7.542 7.530 7.518 7.511 7.500 7.488 7.238 7.228 7.214 7.201 7.031 6.956 6.944 5.483 5.471 5.460 4.669 4.642 4.598 4.567 4.547 4.357 4.346 4.339 4.333 4.328 4.322 4.316 4.309 4.303 4.298 4.292 4.286 4.274 4.184 4.163 3.009 2.998 2.984 2.974 2.947 2.937 2.923 2.912 1.361 1.348 1.337
X : parts per Million : Proton

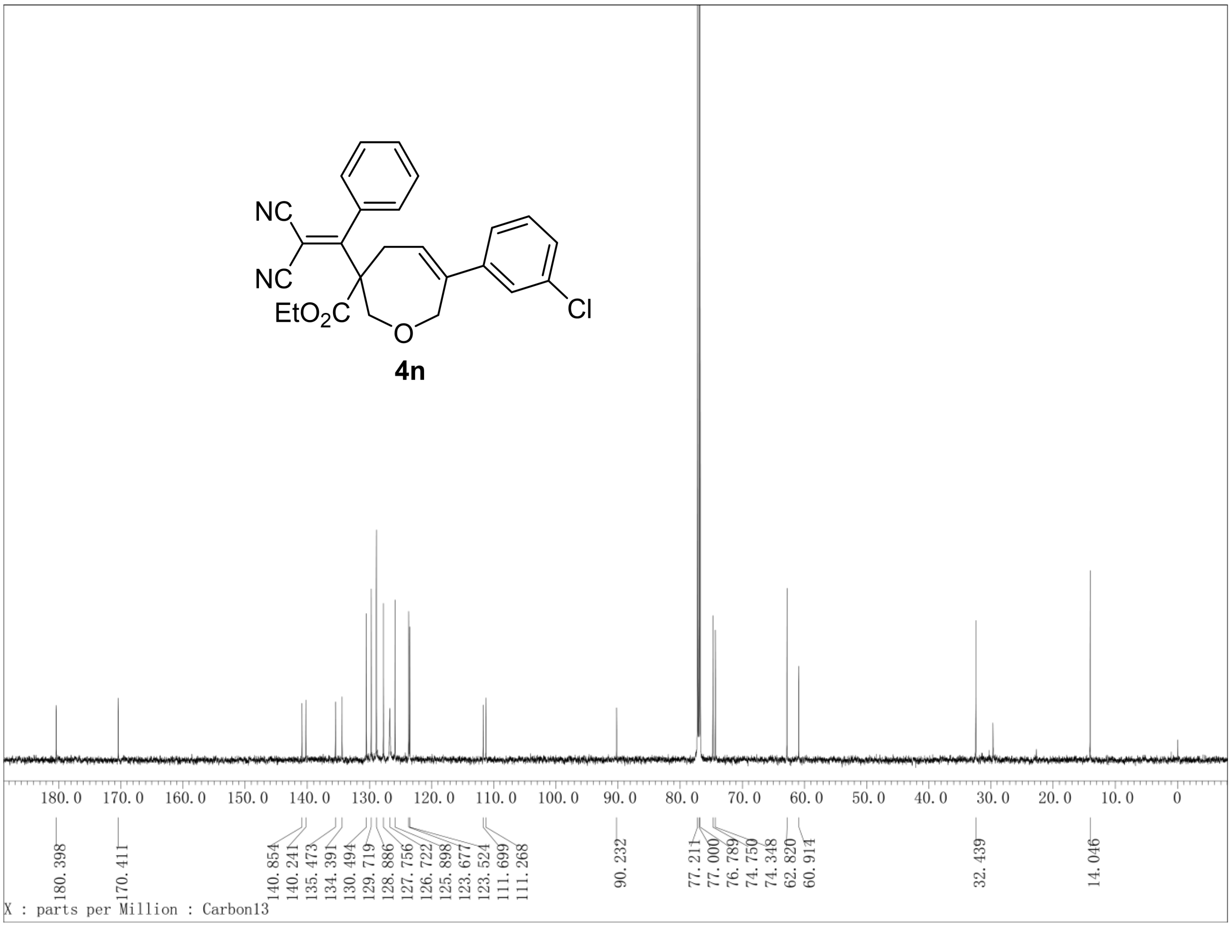
NC
NC
EtO2C
O
Cl
4n
180.0 170.0 160.0 150.0 140.0 130.0 120.0 110.0 100.0 90.0 80.0 70.0 60.0 50.0 40.0 30.0 20.0 10.0 0
180.398 170.411 140.854 140.241 135.473 134.391 130.494 129.719 128.886 127.756 126.722 125.898 123.677 123.524 111.699 111.268 90.232 77.211 77.000 76.789 74.750 74.348 62.820 60.914 32.439 14.046
X : parts per Million : Carbon13

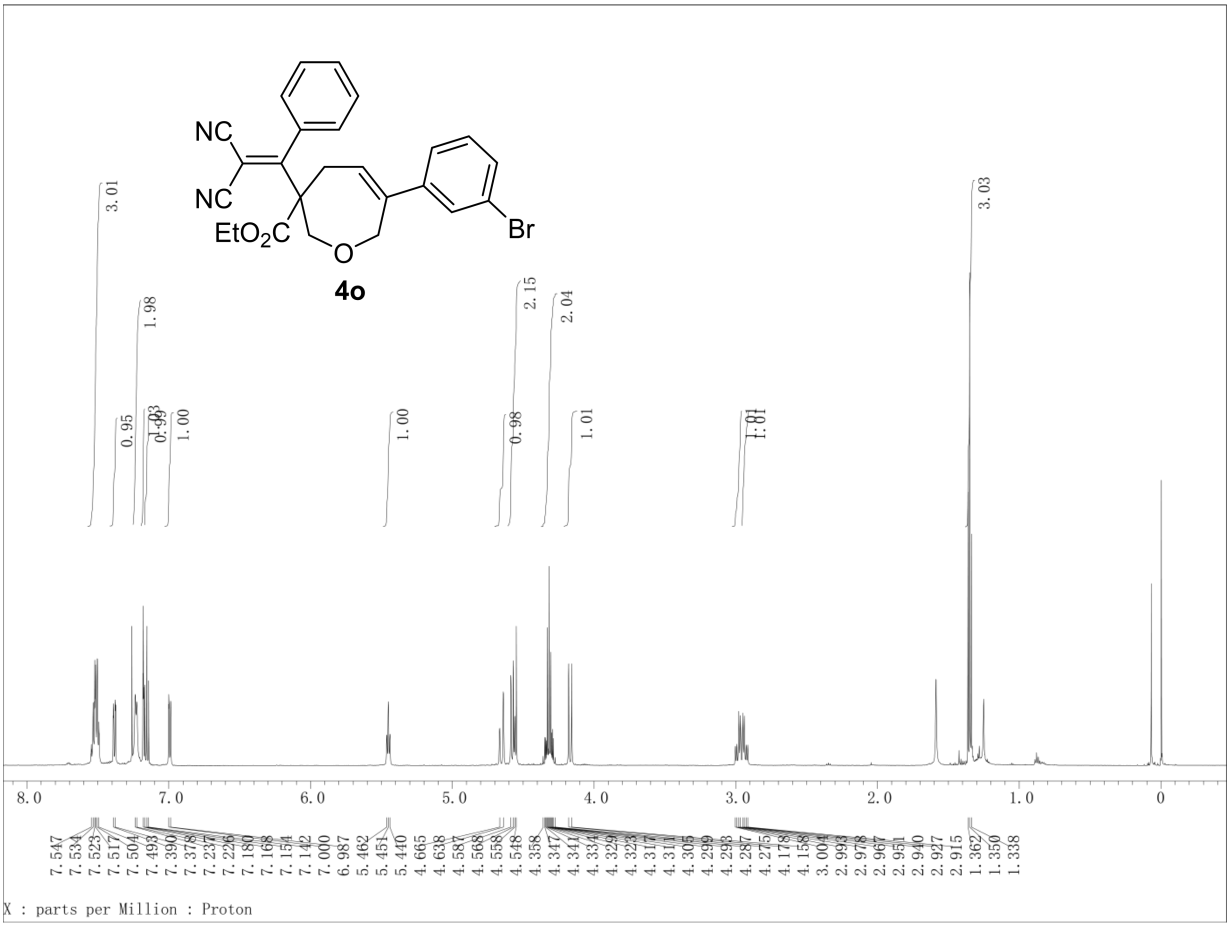

NC
NC
EtO2C
O
Br
4o
X : parts per Million : Proton

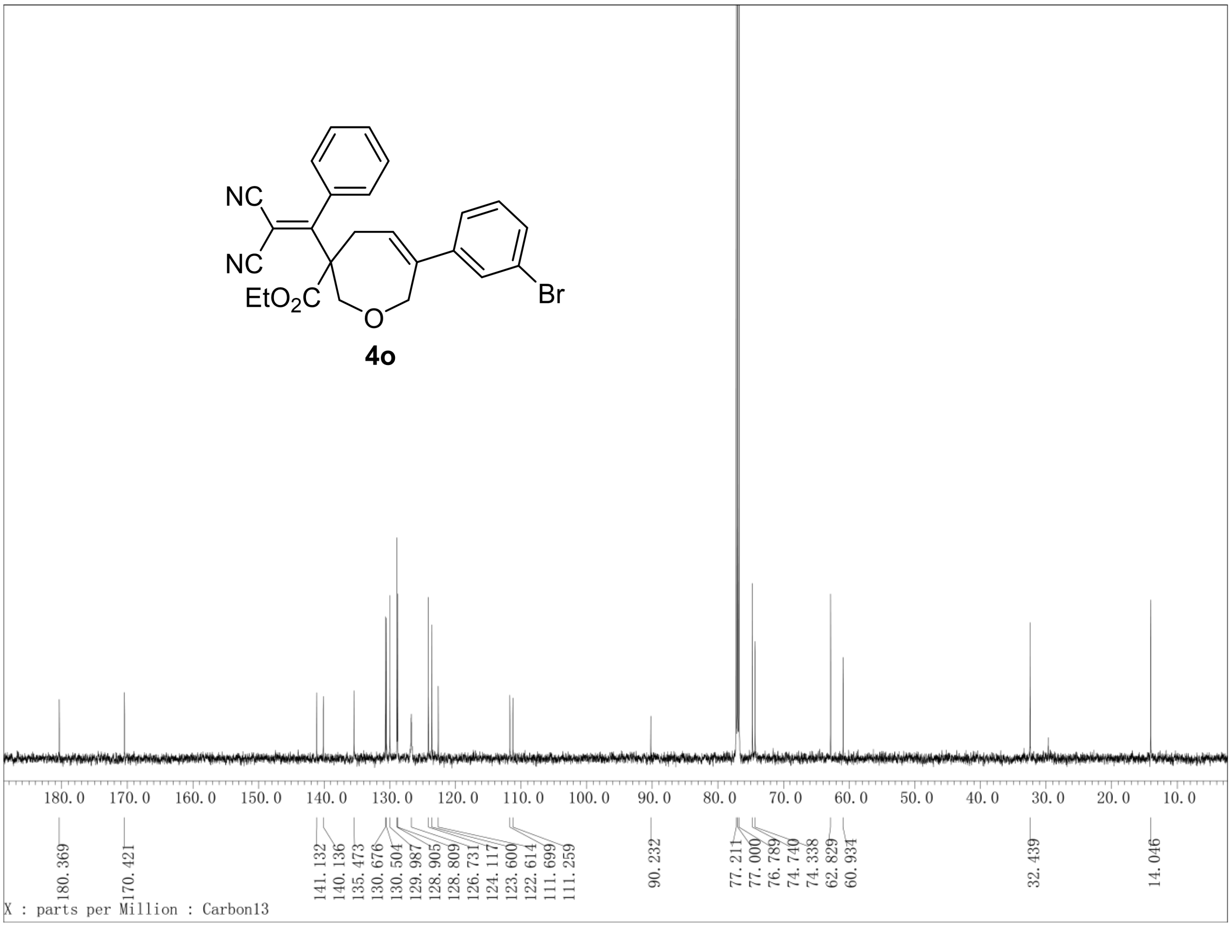

NC
NC
EtO2C
O
Br
4o
X : parts per Million : Carbon13

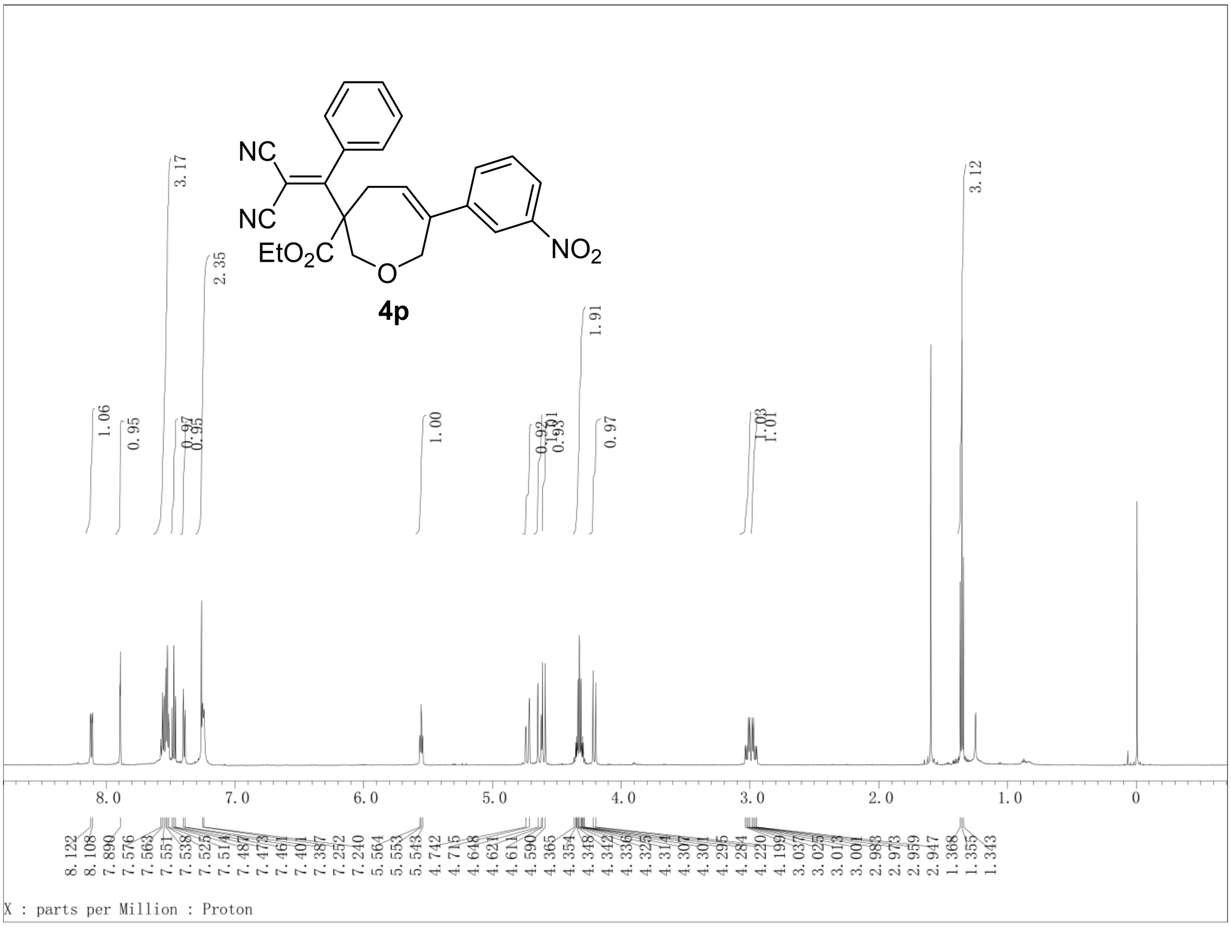
4p
X : parts per Million : Proton

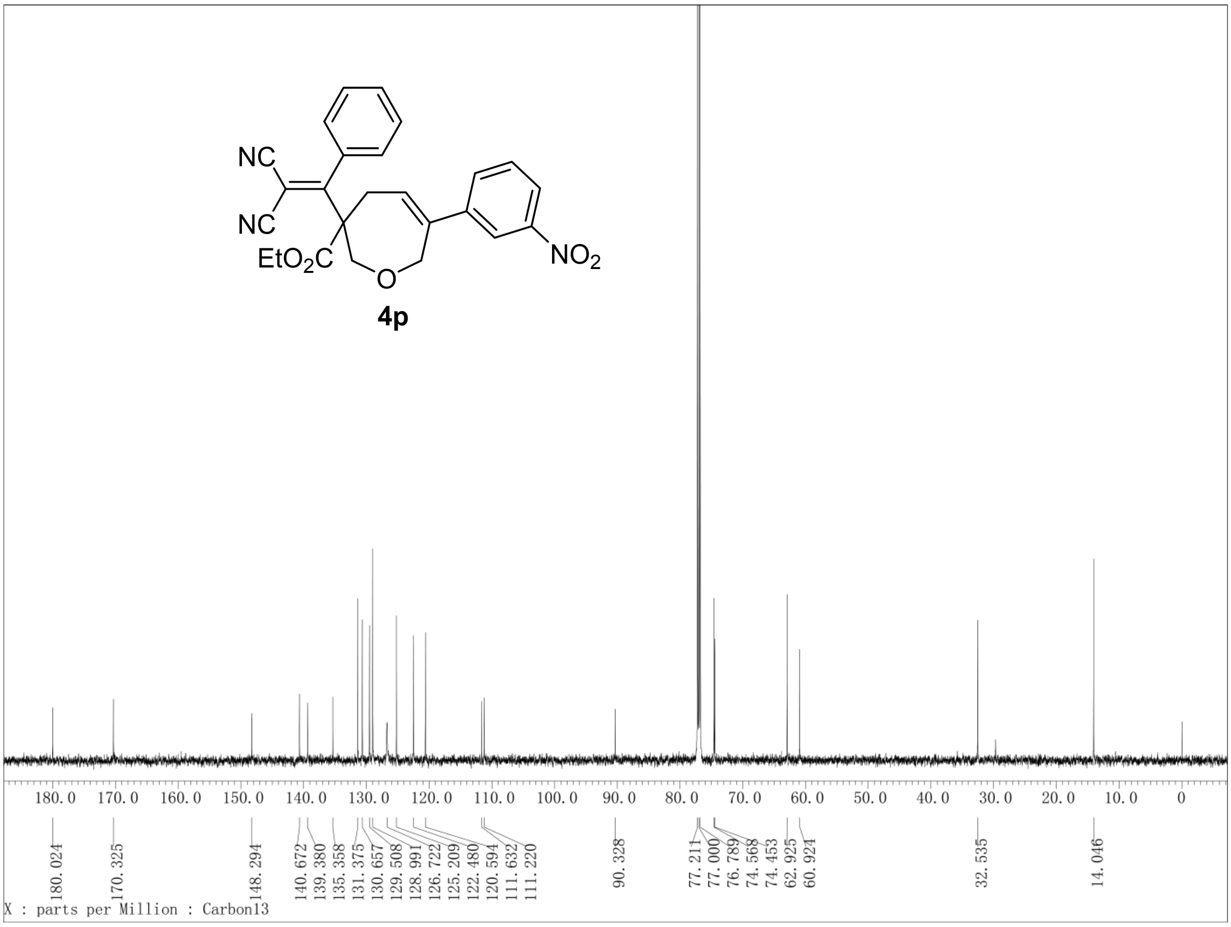
4p
X : parts per Million : Carbon13

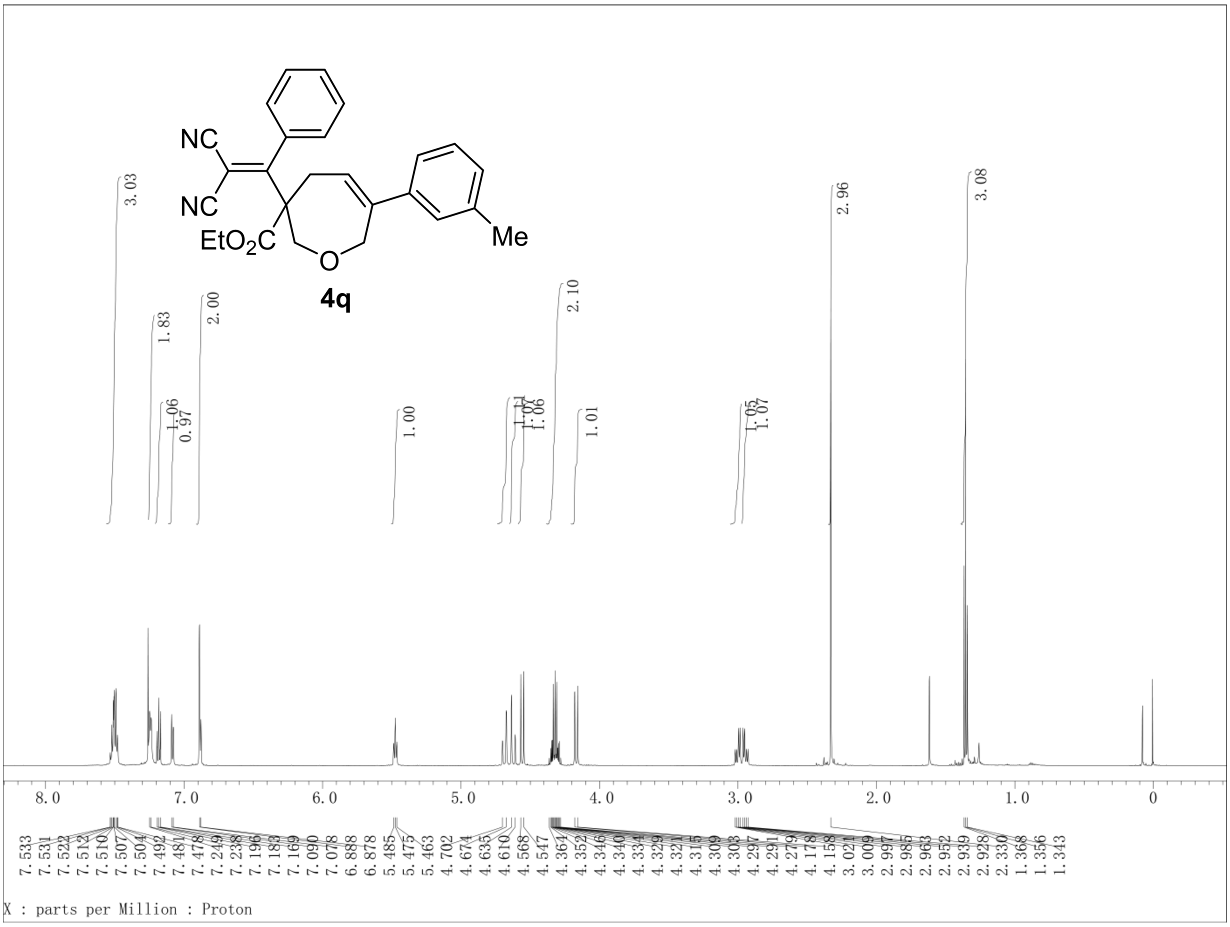

NC
NC
EtO2C
O
Me
4q
X : parts per Million : Proton

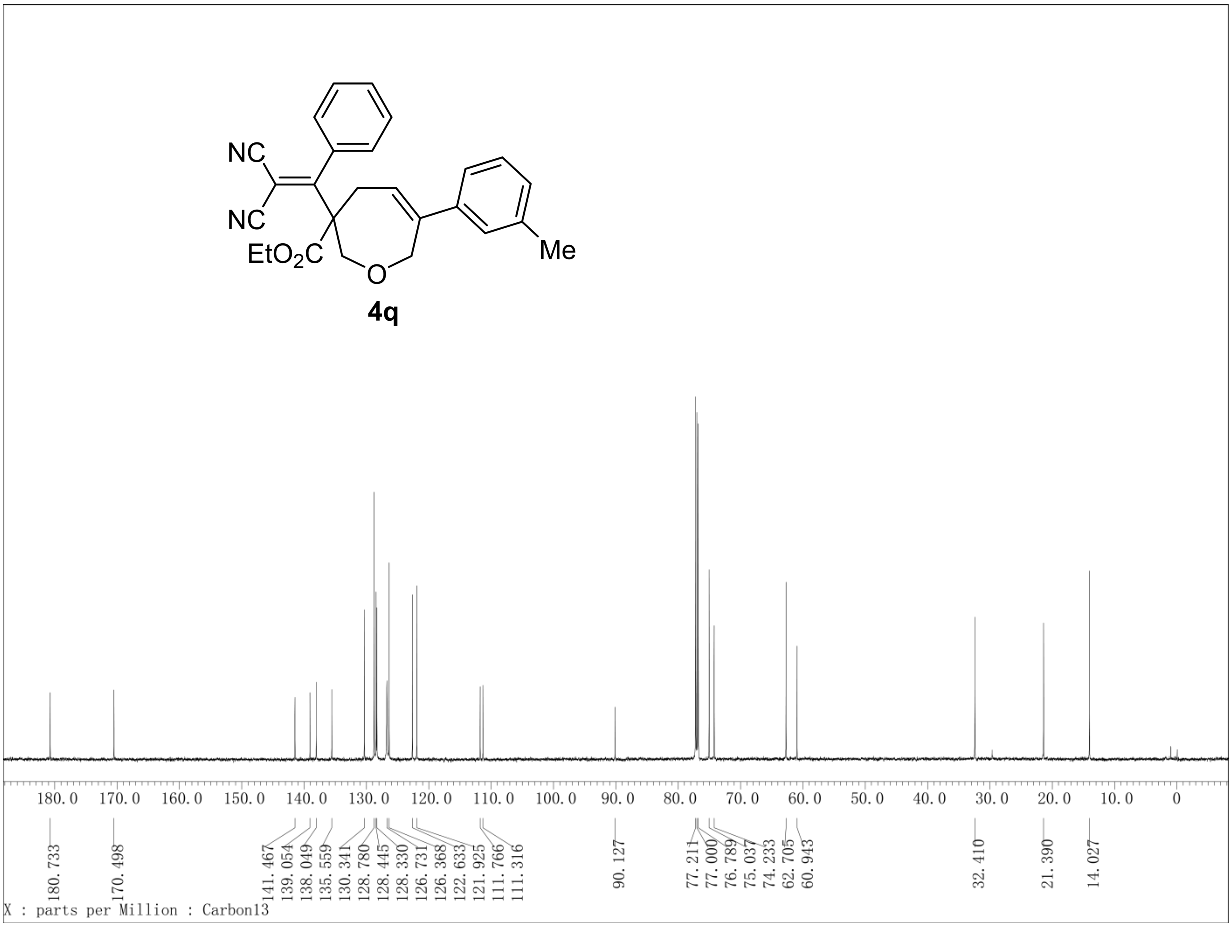

NC
NC
EtO2C
O
Me
4q
X : parts per Million : Carbon13

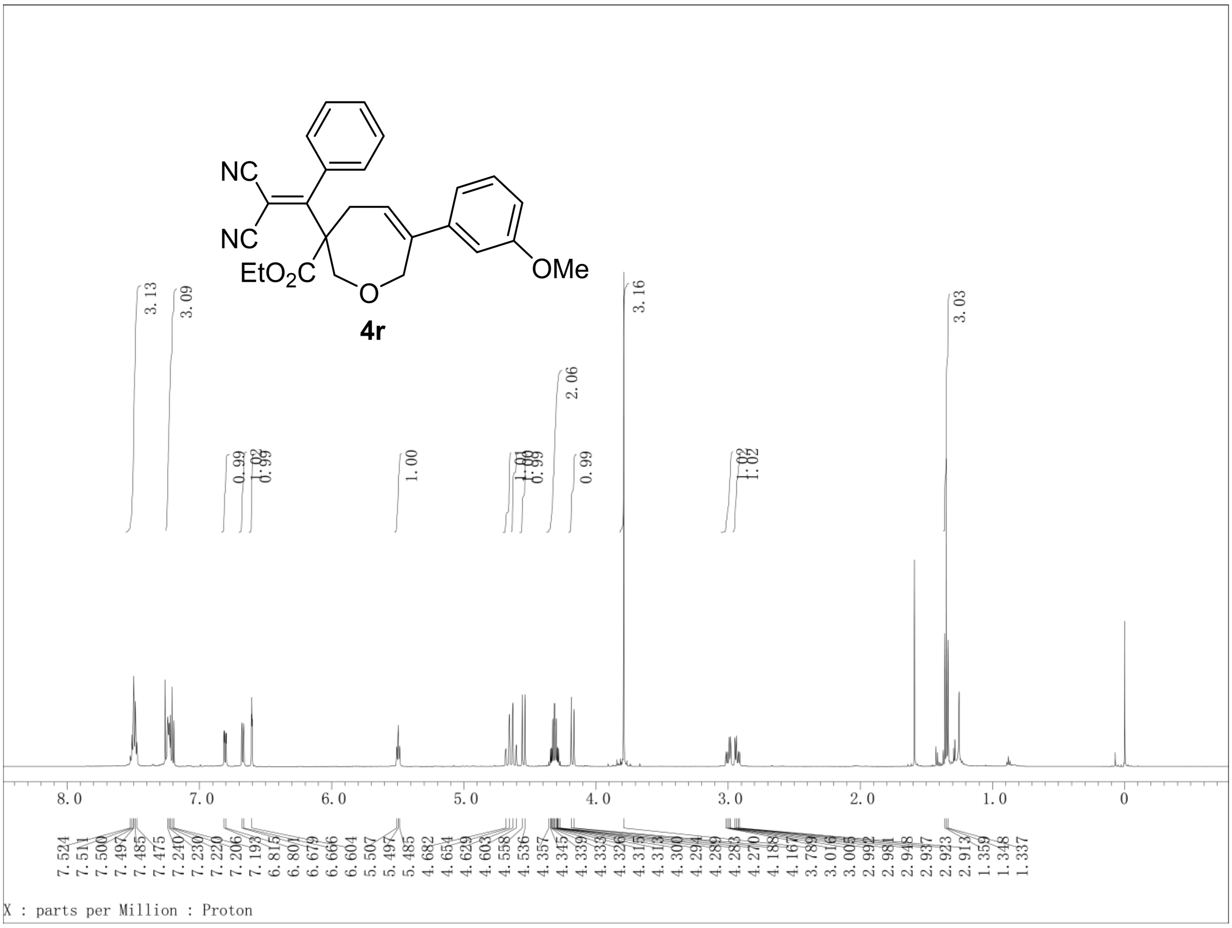
NC
NC
EtO2C
O
OMe
4r
3.13
3.09
0.99
1.02
0.99
1.00
1.01
1.00
0.99
2.06
0.99
3.16
1.02
1.02
3.03
8.0 7.0 6.0 5.0 4.0 3.0 2.0 1.0 0
7.524 7.511 7.500 7.497 7.485 7.475 7.240 7.230 7.220 7.206 7.193 6.815 6.801 6.679 6.666 6.604 5.507 5.497 5.485 4.682 4.654 4.629 4.603 4.558 4.536 4.357 4.345 4.339 4.333 4.326 4.315 4.313 4.300 4.294 4.289 4.283 4.270 4.188 4.167 3.789 3.016 3.005 2.992 2.981 2.948 2.937 2.923 2.913 1.359 1.348 1.337
X : parts per Million : Proton

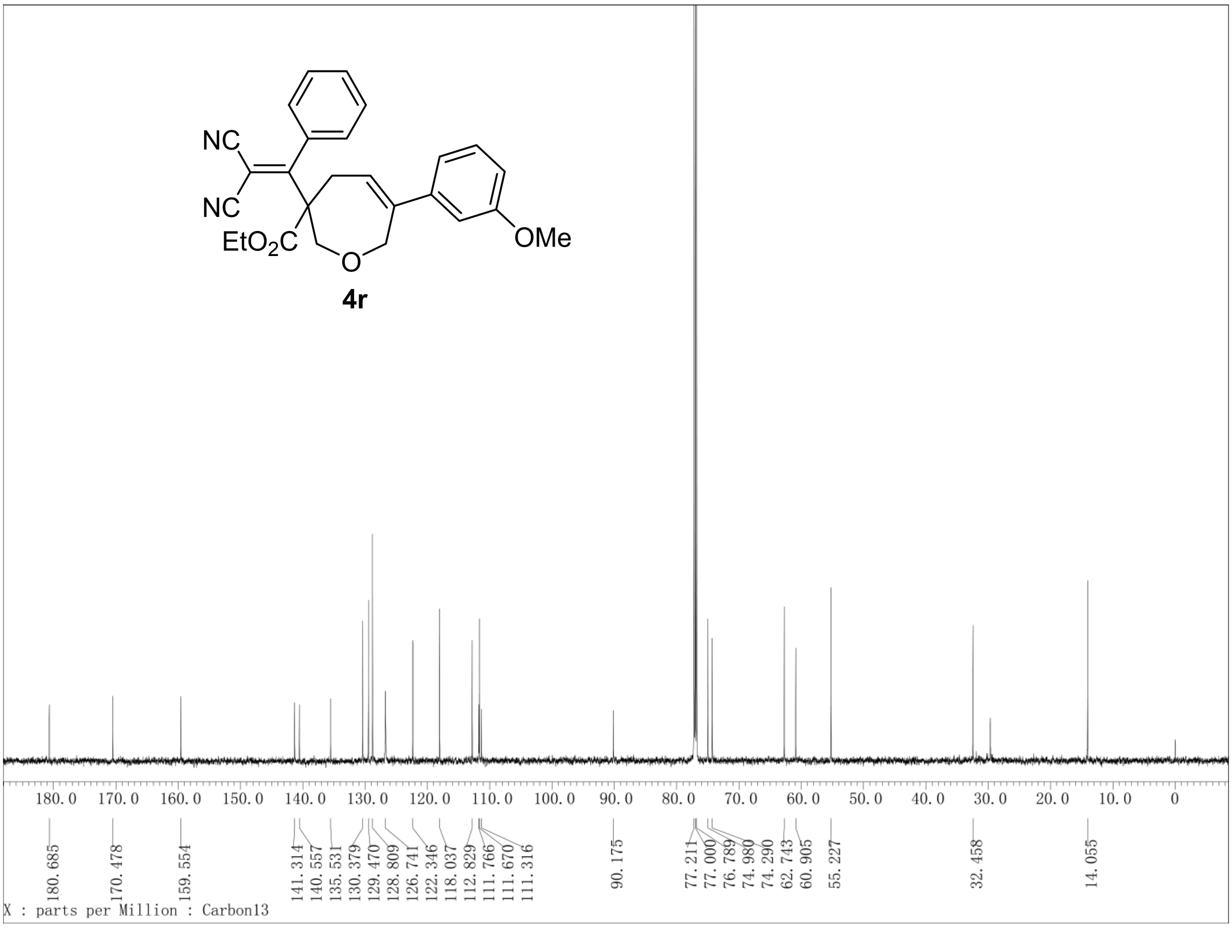
NC
NC
EtO2C
O
OMe
4r
180.0 170.0 160.0 150.0 140.0 130.0 120.0 110.0 100.0 90.0 80.0 70.0 60.0 50.0 40.0 30.0 20.0 10.0 0
180.685 170.478 159.554 141.314 140.557 135.531 130.379 129.470 128.809 126.741 122.346 118.037 112.829 111.766 111.670 111.316 90.175 77.211 77.000 76.789 74.980 74.290 62.743 60.905 55.227 32.458 14.055
X : parts per Million : Carbon13

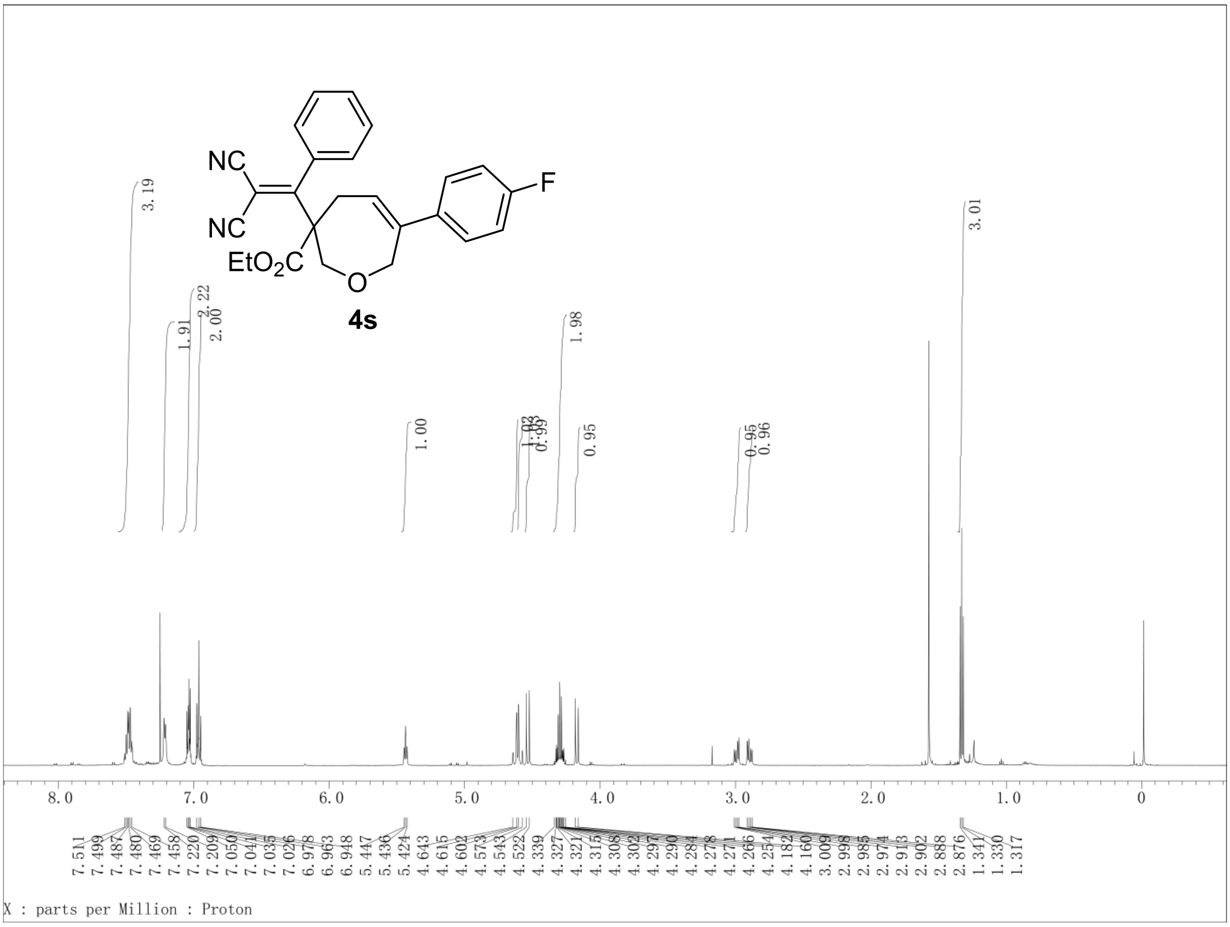
NC
NC
EtO2C
O
F
4s
3.19
1.91
2.22
2.00
1.00
1.02
1.03
0.99
1.98
0.95
0.95
0.96
3.01
8.0
7.0
6.0
5.0
4.0
3.0
2.0
1.0
0
7.511
7.499
7.487
7.480
7.469
7.458
7.220
7.209
7.050
7.041
7.035
7.026
6.978
6.963
6.948
5.447
5.436
5.424
4.643
4.615
4.602
4.573
4.543
4.522
4.339
4.327
4.321
4.315
4.308
4.302
4.297
4.290
4.284
4.278
4.271
4.266
4.254
4.182
4.160
3.009
2.998
2.985
2.974
2.913
2.902
2.888
2.876
1.341
1.330
1.317
X : parts per Million : Proton

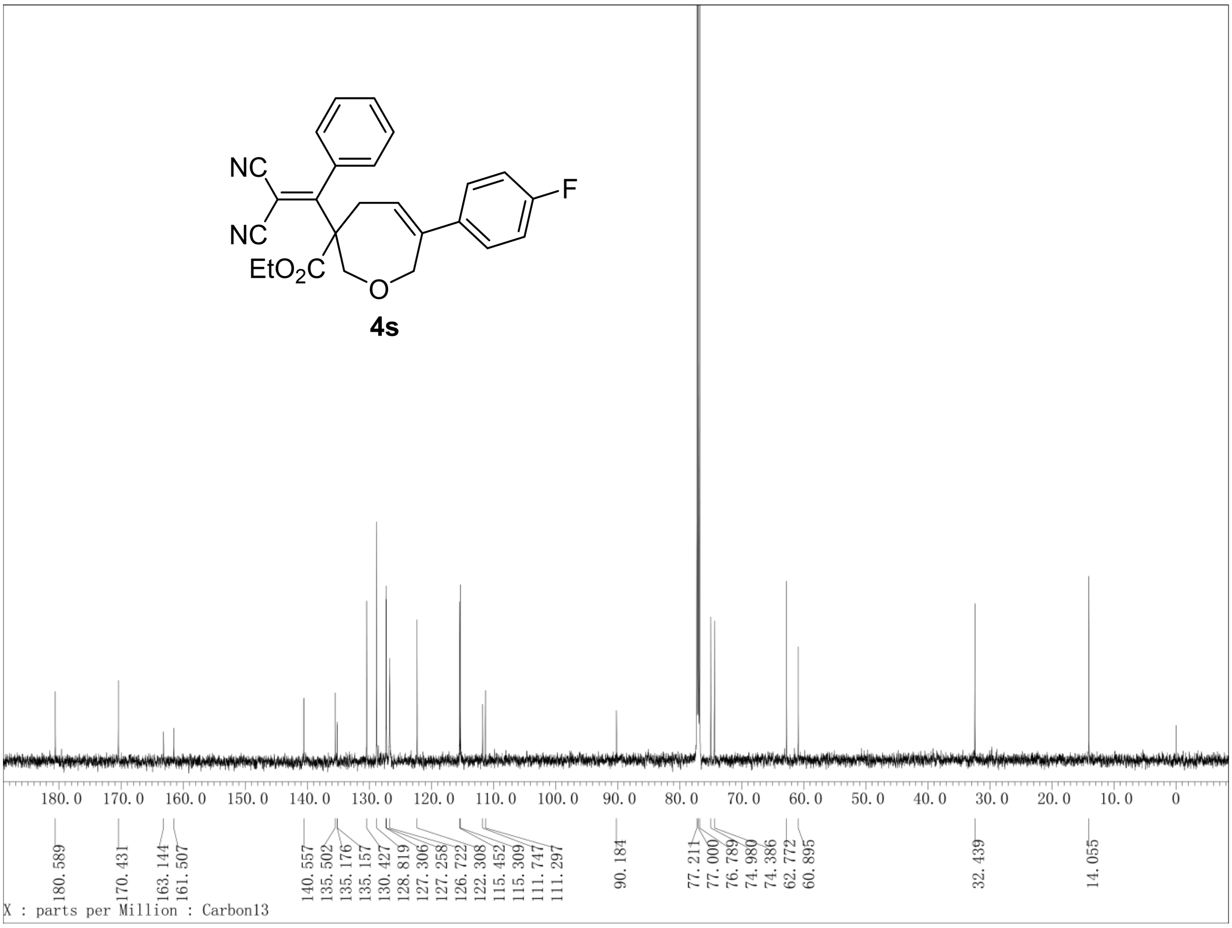
NC
NC
EtO2C
O
F
4s
180.0
170.0
160.0
150.0
140.0
130.0
120.0
110.0
100.0
90.0
80.0
70.0
60.0
50.0
40.0
30.0
20.0
10.0
0
180.589
170.431
163.144
161.507
140.557
135.502
135.176
135.157
130.427
128.819
127.306
127.258
126.722
122.308
115.452
115.309
111.747
111.297
90.184
77.211
77.000
76.789
74.980
74.386
62.772
60.895
32.439
14.055
X : parts per Million : Carbon13

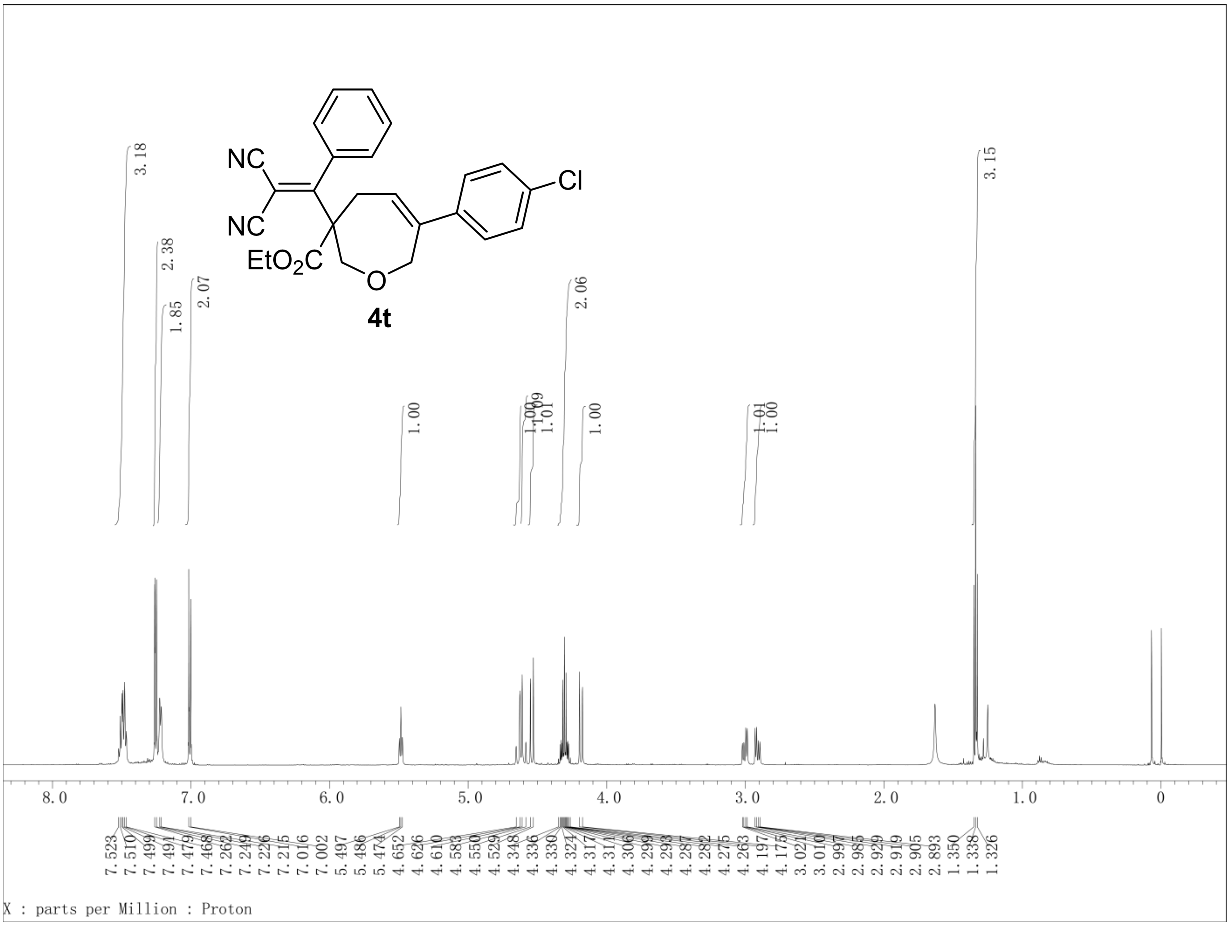
NC
NC
EtO2C
Cl
O
4t
3.18
2.38
1.85
2.07
1.00
1.00
1.09
1.01
2.06
1.00
1.01
1.00
3.15
7.523 7.510 7.499 7.491 7.479 7.468 7.262 7.249 7.226 7.215 7.016 7.002 5.497 5.486 5.474 4.652 4.626 4.610 4.583 4.550 4.529 4.348 4.336 4.330 4.324 4.317 4.311 4.306 4.299 4.293 4.287 4.282 4.275 4.263 4.197 4.175 3.021 3.010 2.997 2.985 2.929 2.919 2.905 2.893 1.350 1.338 1.326
X : parts per Million : Proton

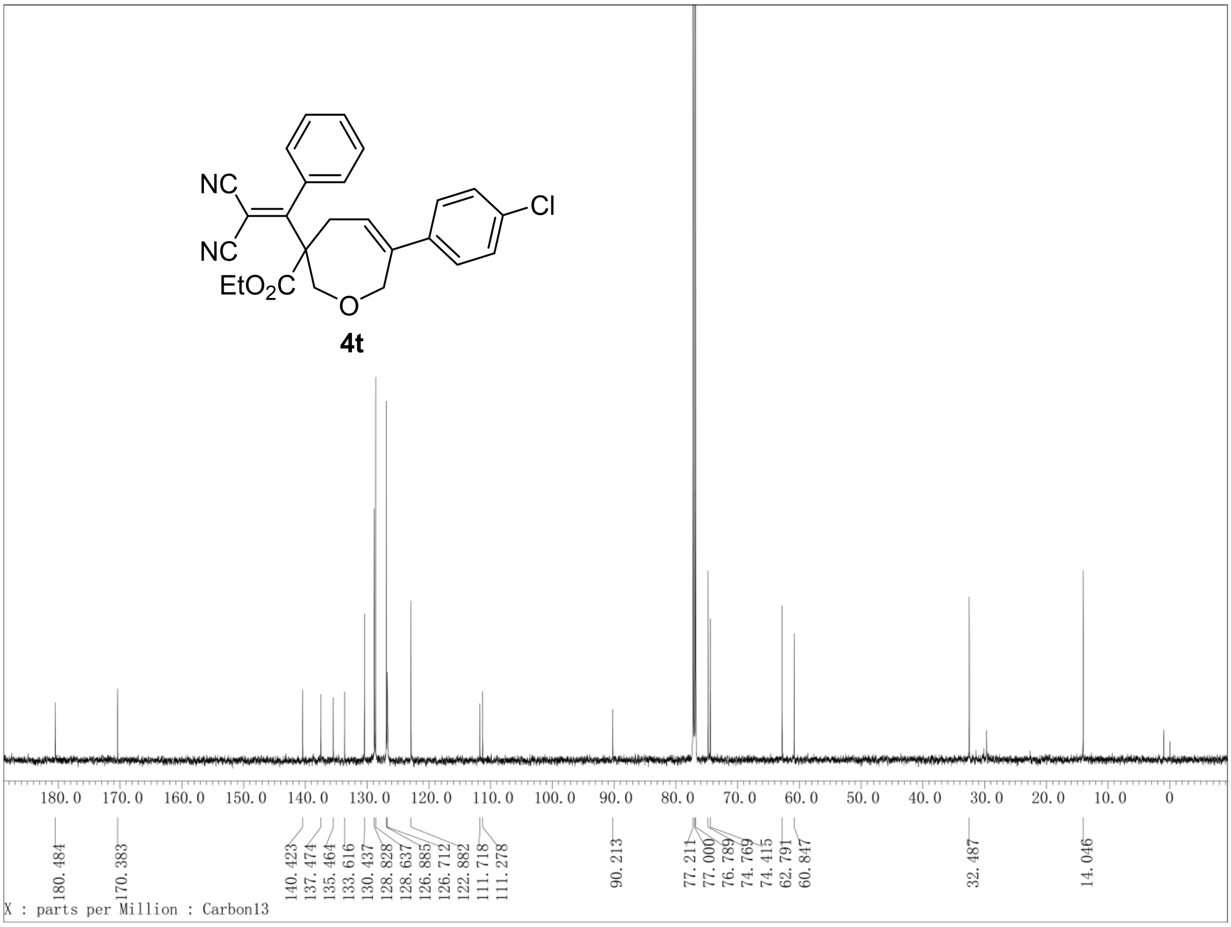
NC
NC
EtO2C
Cl
O
4t
180.484 170.383 140.423 137.474 135.464 133.616 130.437 128.828 128.637 126.885 126.712 122.882 111.718 111.278 90.213 77.211 77.000 76.789 74.769 74.415 62.791 60.847 32.487 14.046
X : parts per Million : Carbon13

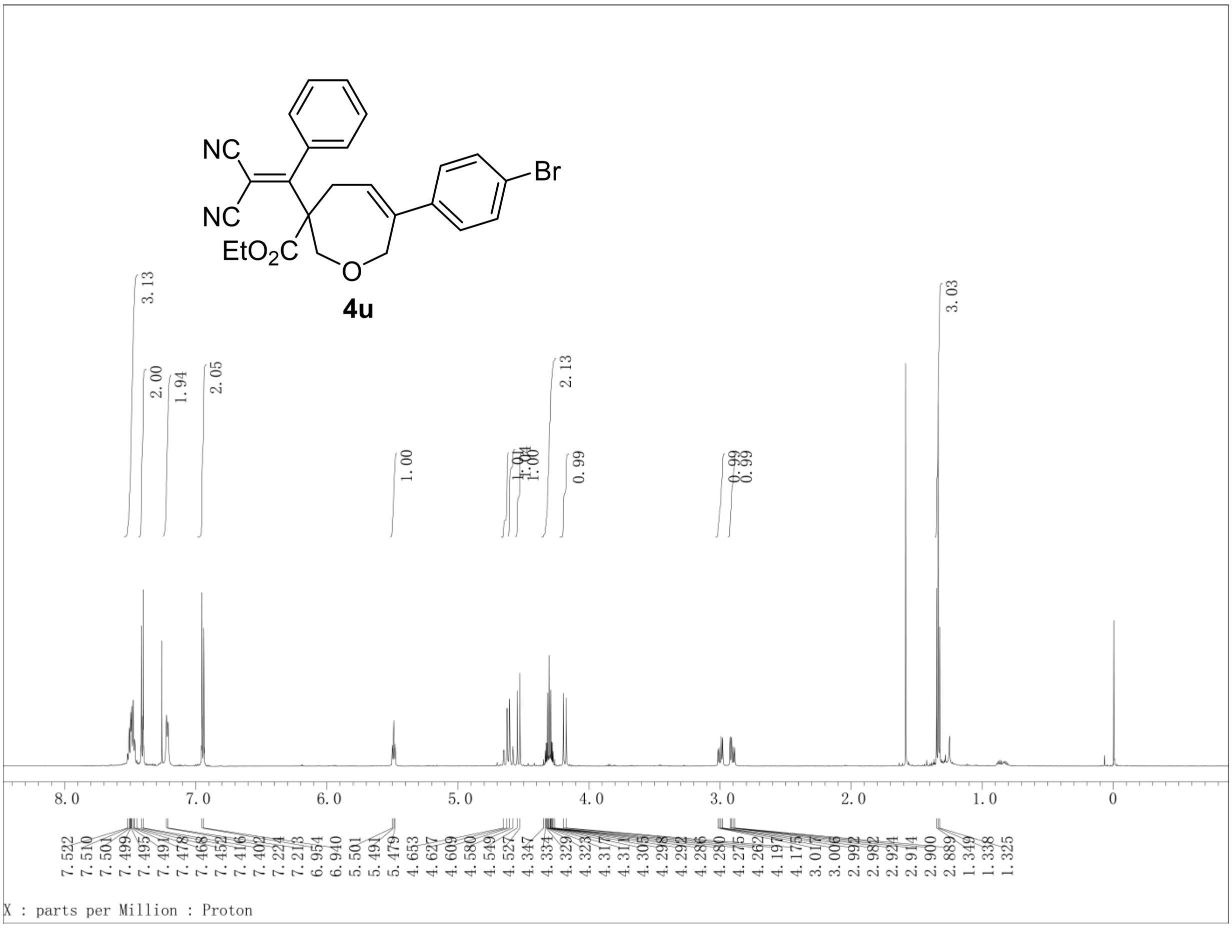
NC
NC
EtO$_2$C
O
Br
4u
3.13
2.00
1.94
2.05
1.00
2.13
0.99
0.99
0.99
3.03
8.0 7.0 6.0 5.0 4.0 3.0 2.0 1.0 0
7.522 7.510 7.501 7.499 7.495 7.491 7.478 7.468 7.452 7.416 7.402 7.224 7.213 6.954 6.940 5.501 5.491 5.479 4.653 4.627 4.609 4.580 4.549 4.527 4.347 4.334 4.329 4.323 4.317 4.311 4.305 4.298 4.292 4.286 4.280 4.275 4.262 4.197 4.175 3.017 3.006 2.992 2.982 2.924 2.914 2.900 2.889 1.349 1.338 1.325
X : parts per Million : Proton

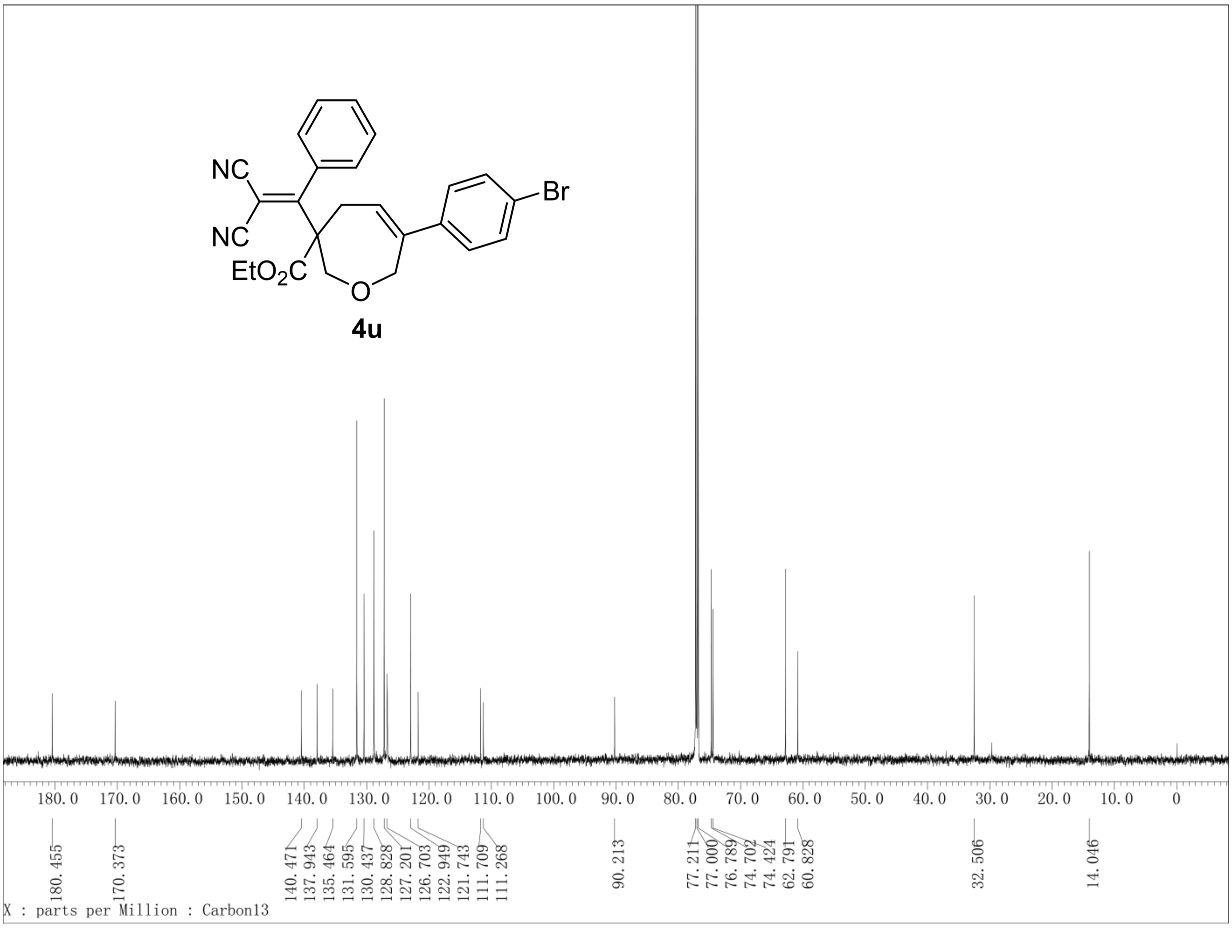
NC
NC
EtO$_2$C
O
Br
4u
180.0 170.0 160.0 150.0 140.0 130.0 120.0 110.0 100.0 90.0 80.0 70.0 60.0 50.0 40.0 30.0 20.0 10.0 0
180.455 170.373 140.471 137.943 135.464 131.595 130.437 128.828 127.201 126.703 122.949 121.743 111.709 111.268 90.213 77.211 77.000 76.789 74.702 74.424 62.791 60.828 32.506 14.046
X : parts per Million : Carbon13

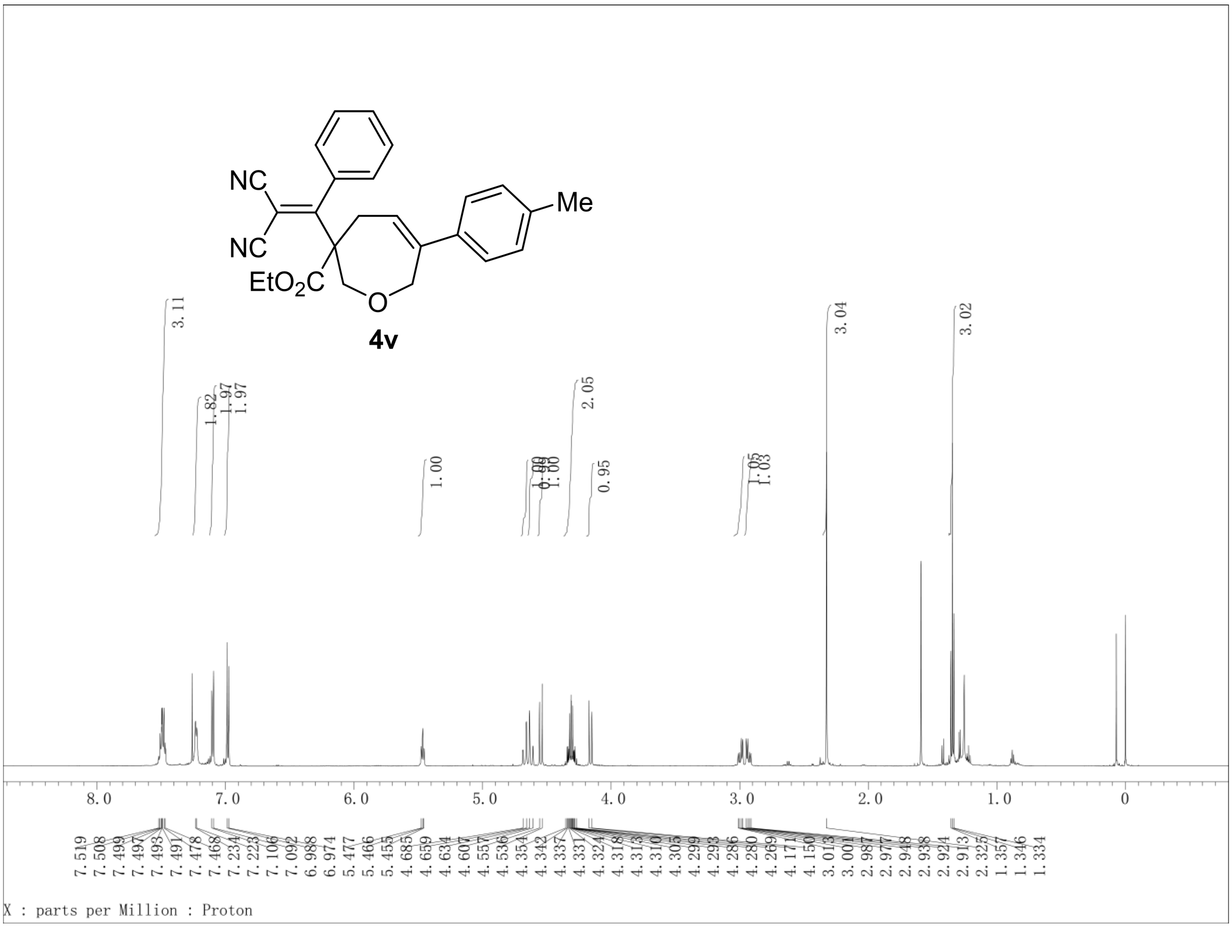

NC
NC
EtO2C
O
Me
4v
X : parts per Million : Proton

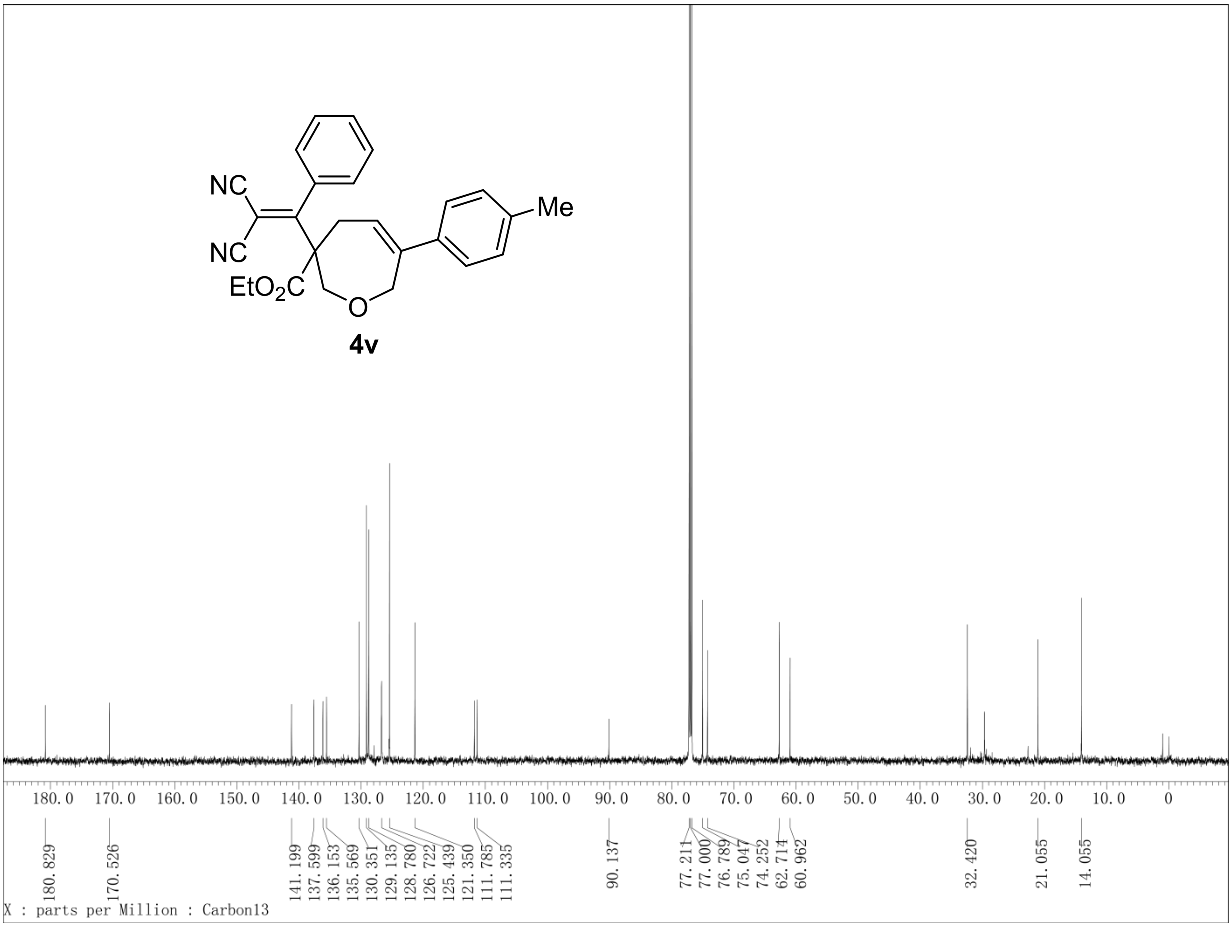

NC
NC
EtO2C
O
Me
4v
X : parts per Million : Carbon13

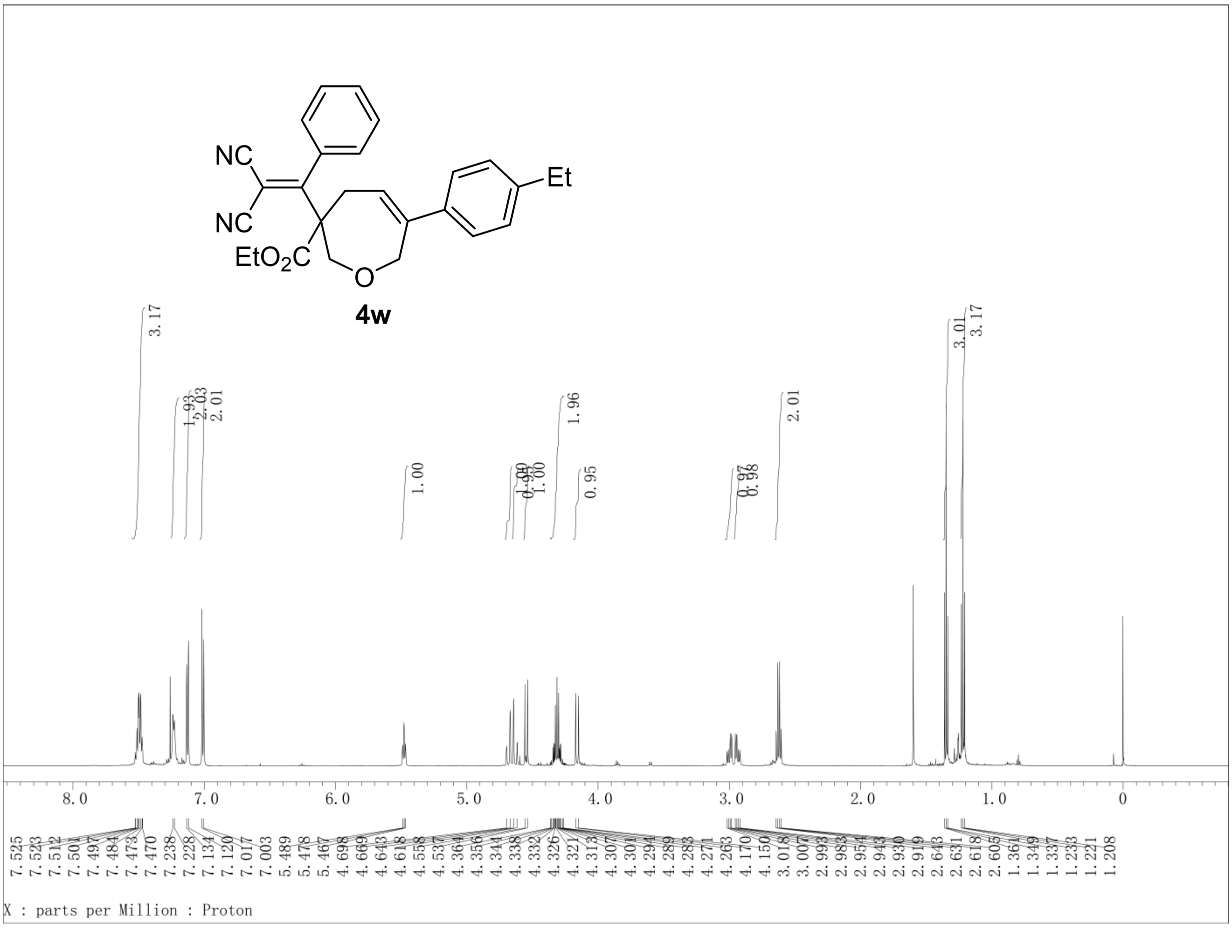
NC
NC
EtO2C
O
Et
4w
3.17
1.93
2.03
2.01
1.00
1.00
0.95
1.00
1.96
0.95
0.97
0.98
2.01
3.01
3.17
8.0
7.0
6.0
5.0
4.0
3.0
2.0
1.0
0
7.525
7.523
7.512
7.501
7.497
7.484
7.473
7.470
7.238
7.228
7.134
7.120
7.017
7.003
5.489
5.478
5.467
4.698
4.669
4.643
4.618
4.558
4.537
4.364
4.356
4.344
4.338
4.332
4.326
4.321
4.313
4.307
4.301
4.294
4.289
4.283
4.271
4.263
4.170
4.150
3.018
3.007
2.993
2.983
2.954
2.943
2.930
2.919
2.643
2.631
2.618
2.605
1.361
1.349
1.337
1.233
1.221
1.208
X : parts per Million : Proton

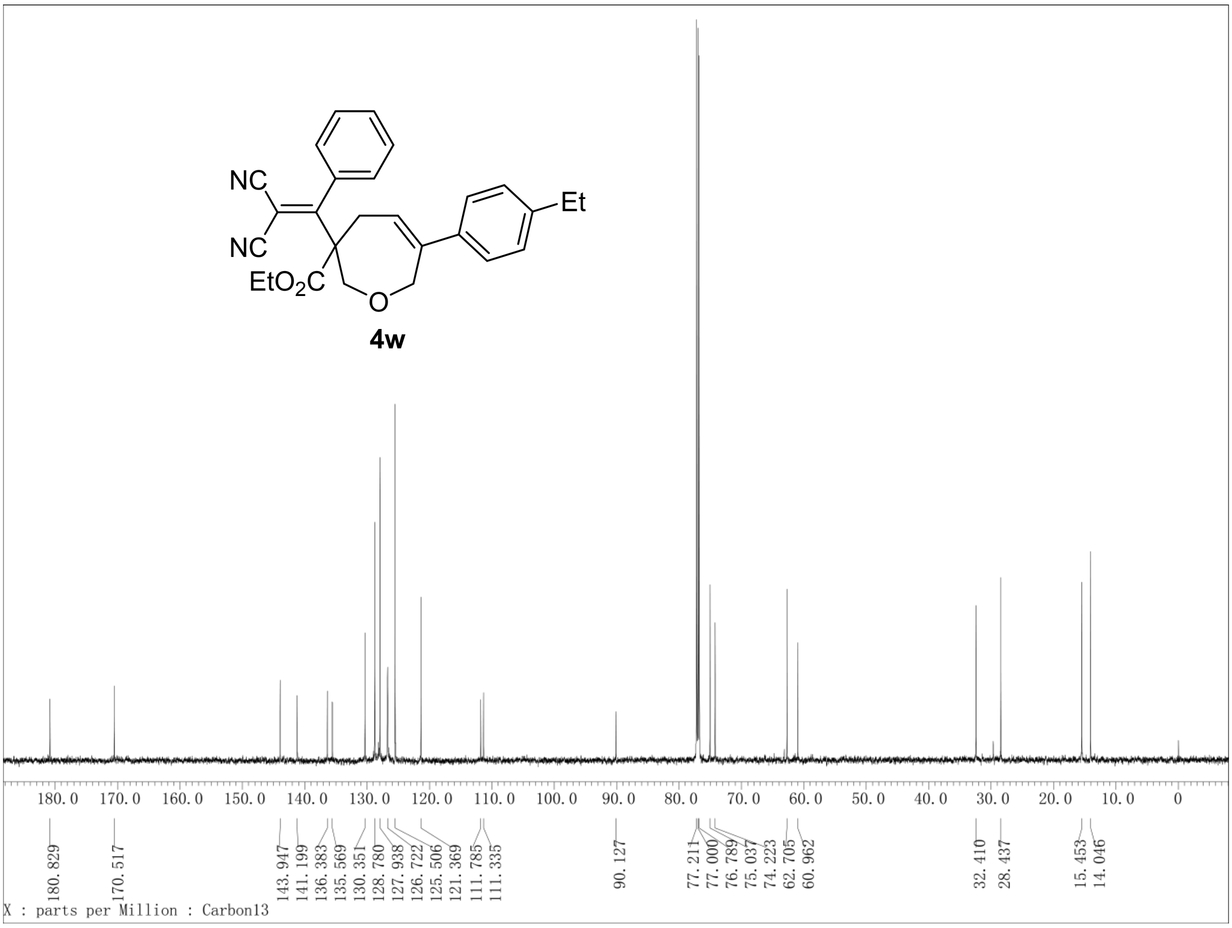
NC
NC
EtO2C
O
Et
4w
180.0
170.0
160.0
150.0
140.0
130.0
120.0
110.0
100.0
90.0
80.0
70.0
60.0
50.0
40.0
30.0
20.0
10.0
0
180.829
170.517
143.947
141.199
136.383
135.569
130.351
128.780
127.938
126.722
125.506
121.369
111.785
111.335
90.127
77.211
77.000
76.789
75.037
74.223
62.705
60.962
32.410
28.437
15.453
14.046
X : parts per Million : Carbon13

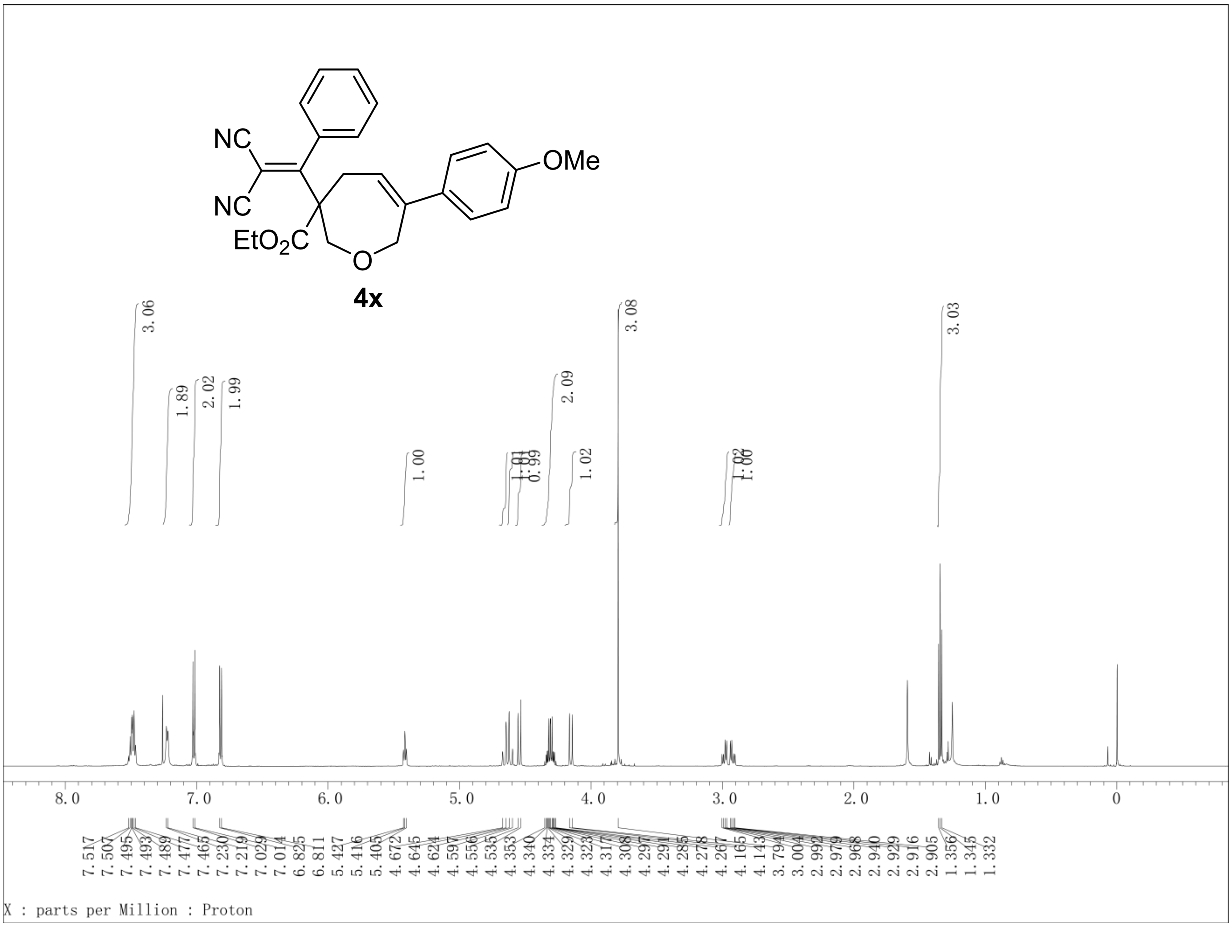

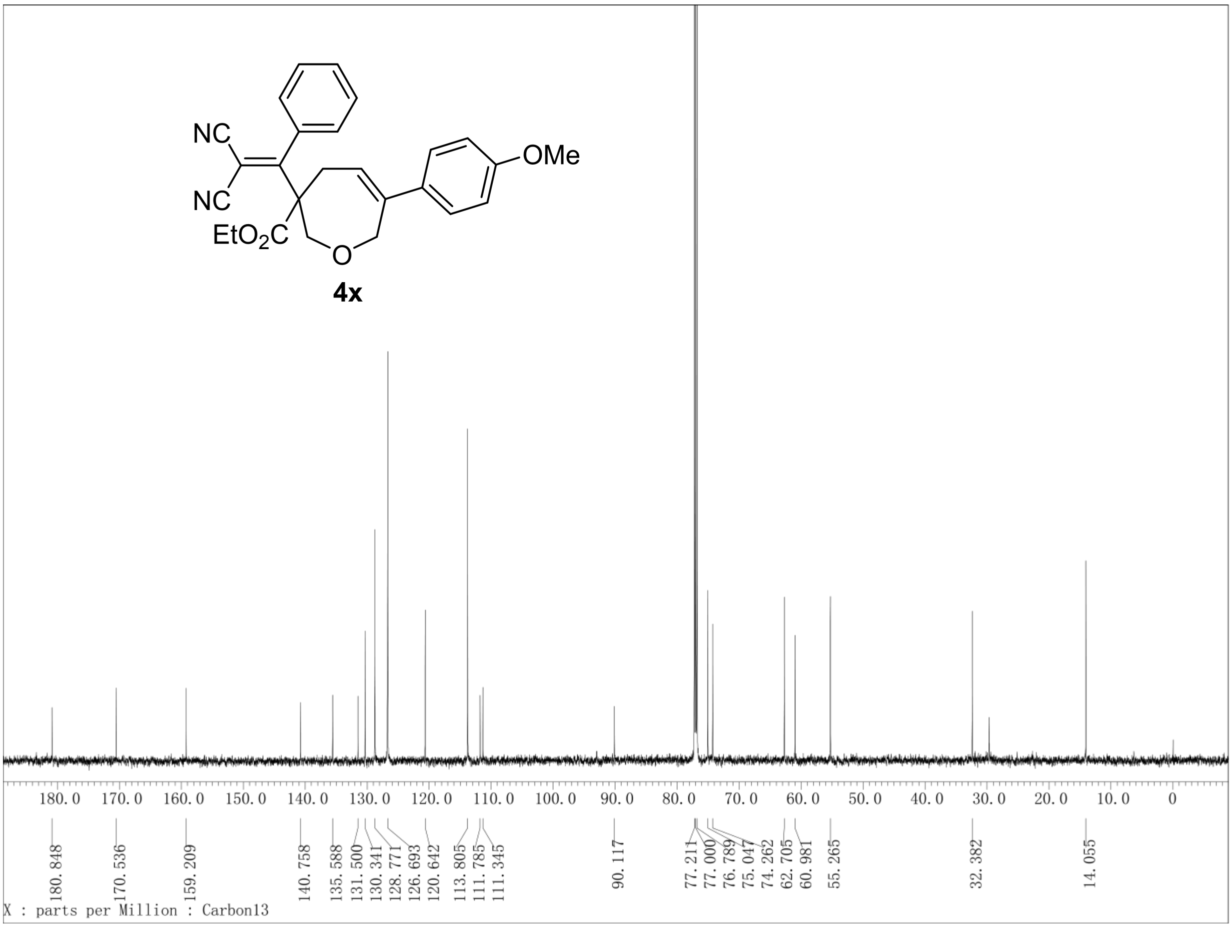

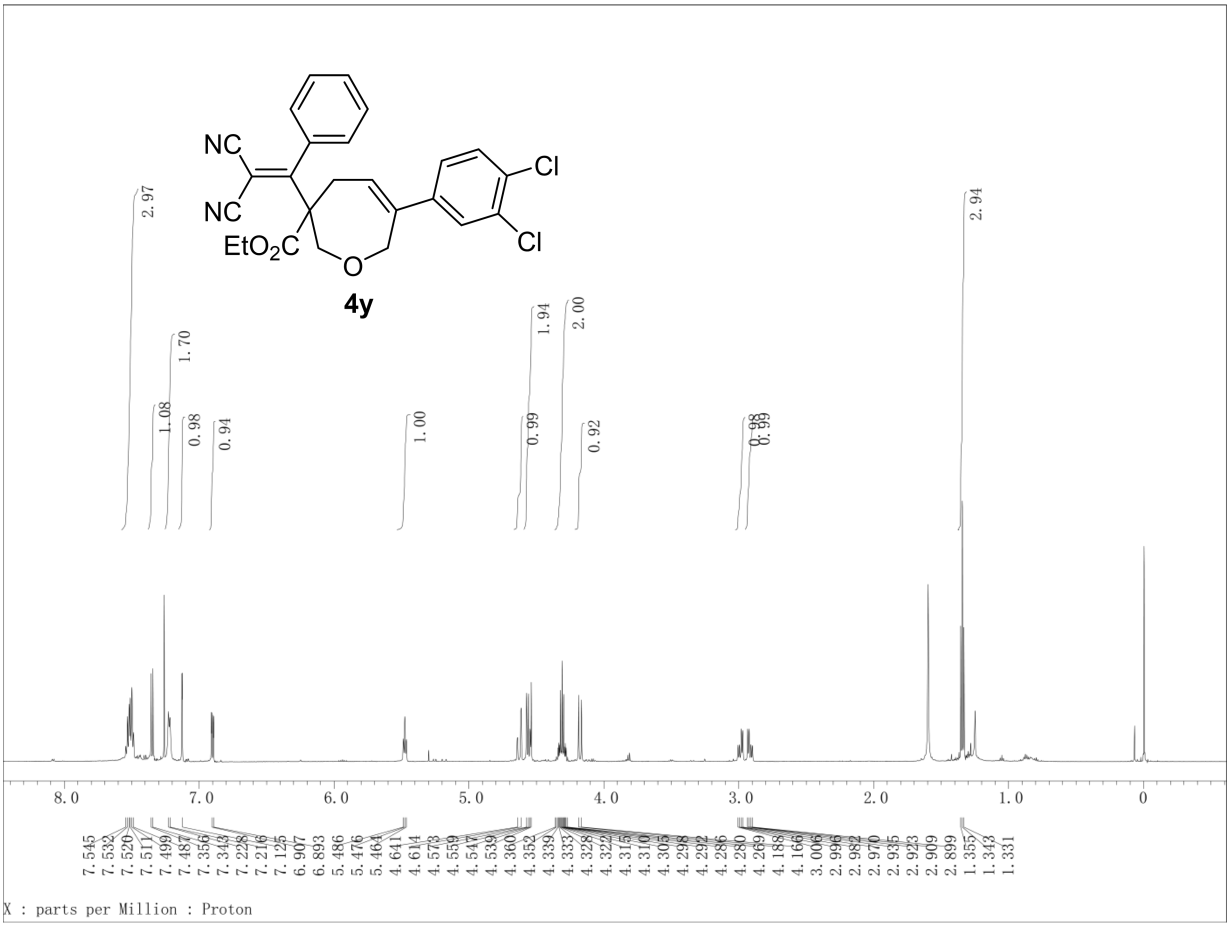
NC
NC
EtO2C
O
Cl
Cl
4y
2.97
1.08
1.70
0.98
0.94
1.00
0.99
1.94
2.00
0.92
0.98
0.99
2.94
8.0
7.0
6.0
5.0
4.0
3.0
2.0
1.0
0
7.545
7.532
7.520
7.511
7.499
7.487
7.356
7.343
7.228
7.216
7.125
6.907
6.893
5.486
5.476
5.464
4.641
4.614
4.573
4.559
4.547
4.539
4.360
4.352
4.339
4.333
4.328
4.322
4.315
4.310
4.305
4.298
4.292
4.286
4.280
4.269
4.188
4.166
3.006
2.996
2.982
2.970
2.935
2.923
2.909
2.899
1.355
1.343
1.331
X : parts per Million : Proton

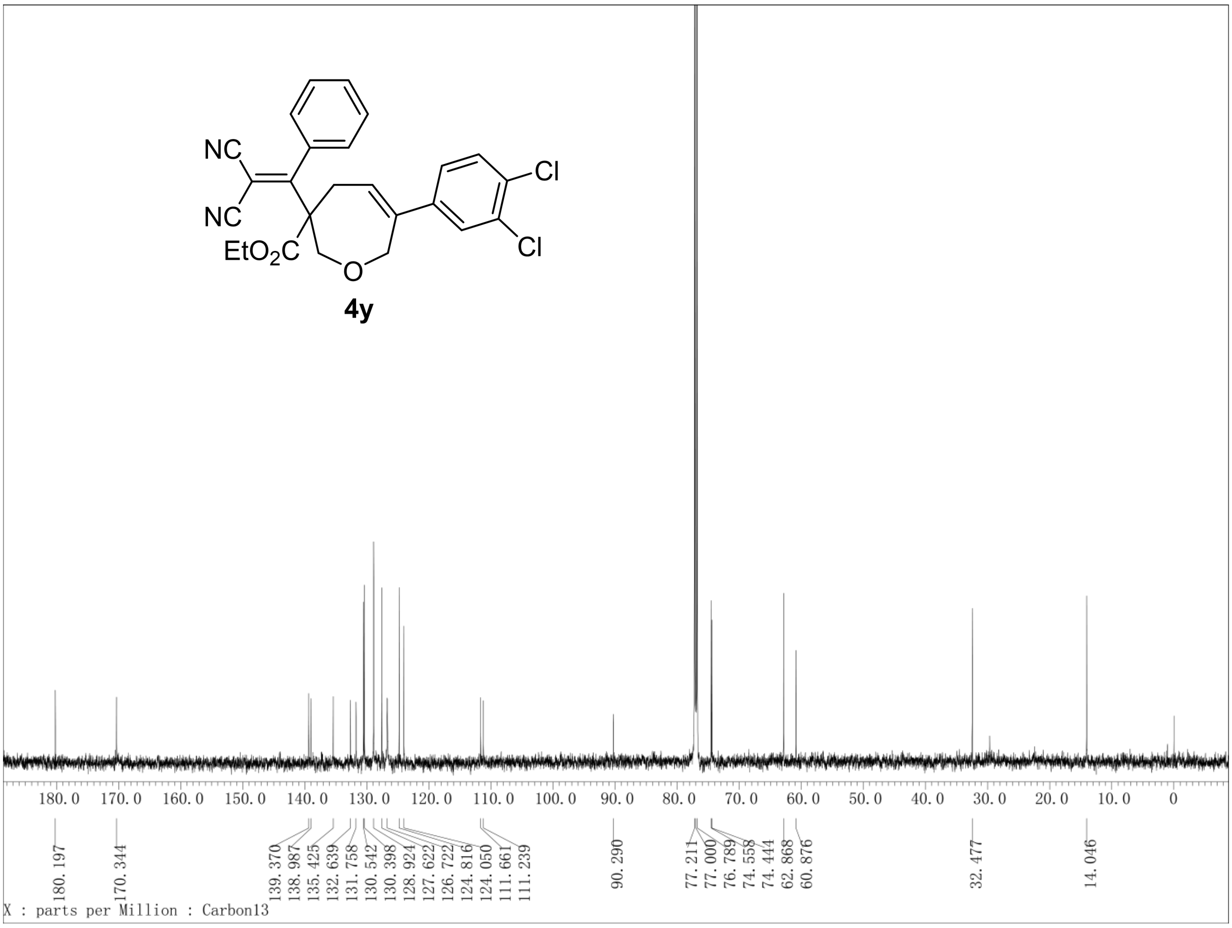
NC
NC
EtO2C
O
Cl
Cl
4y
180.0
170.0
160.0
150.0
140.0
130.0
120.0
110.0
100.0
90.0
80.0
70.0
60.0
50.0
40.0
30.0
20.0
10.0
0
180.197
170.344
139.370
138.987
135.425
132.639
131.758
130.542
130.398
128.924
127.622
126.722
124.816
124.050
111.661
111.239
90.290
77.211
77.000
76.789
74.558
74.444
62.868
60.876
32.477
14.046
X : parts per Million : Carbon13

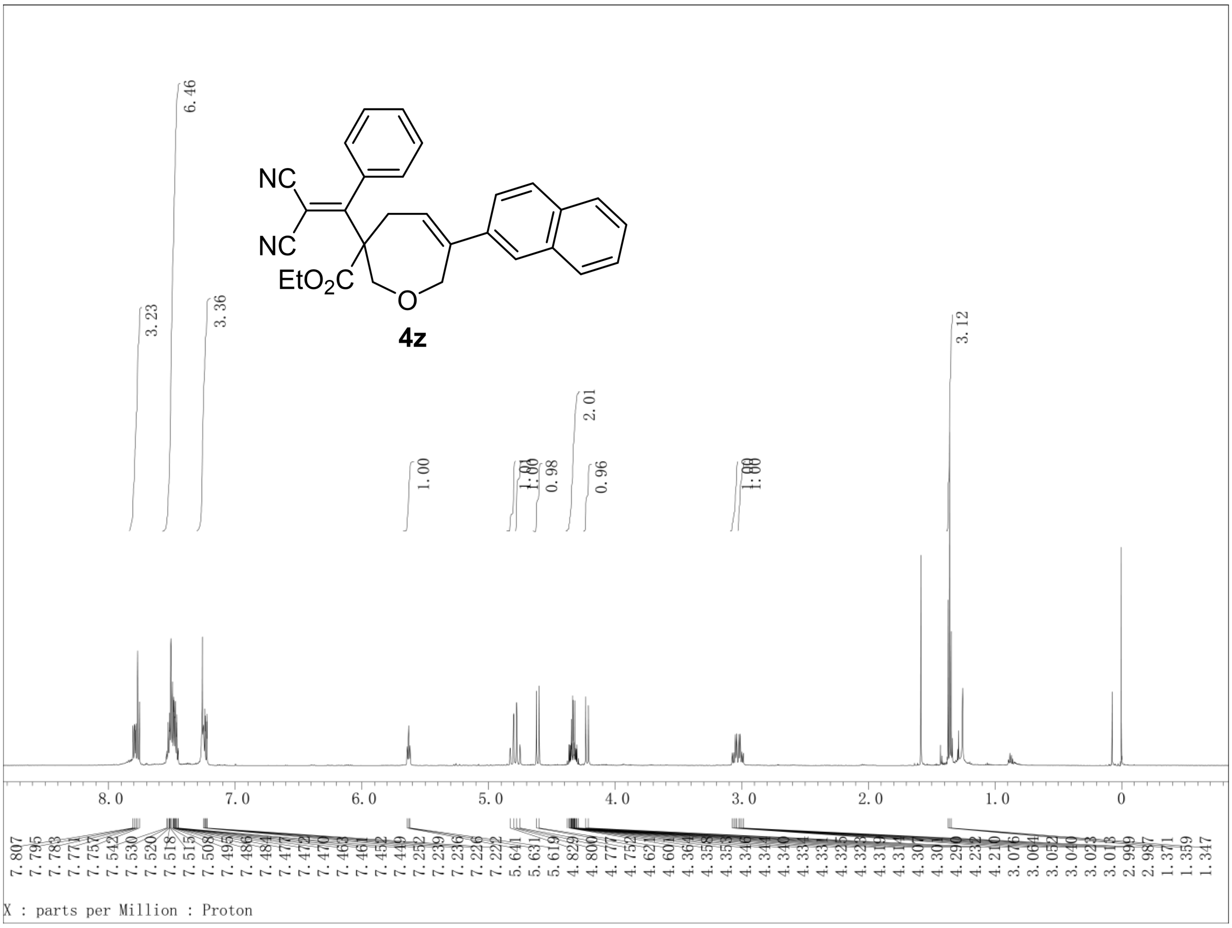
NC
NC
EtO2C
O
4z
3.23
6.46
3.36
1.00
0.98
2.01
0.96
3.12
8.0 7.0 6.0 5.0 4.0 3.0 2.0 1.0 0
7.807 7.795 7.783 7.771 7.757 7.542 7.530 7.520 7.518 7.515 7.508 7.495 7.486 7.484 7.477 7.472 7.470 7.463 7.461 7.452 7.449 7.252 7.239 7.236 7.226 7.222 5.641 5.631 5.619 4.829 4.800 4.777 4.752 4.621 4.601 4.364 4.358 4.355 4.346 4.340 4.232 4.210 3.076 3.064 3.052 3.040 3.023 3.013 2.999 2.987 1.371 1.359 1.347
X : parts per Million : Proton

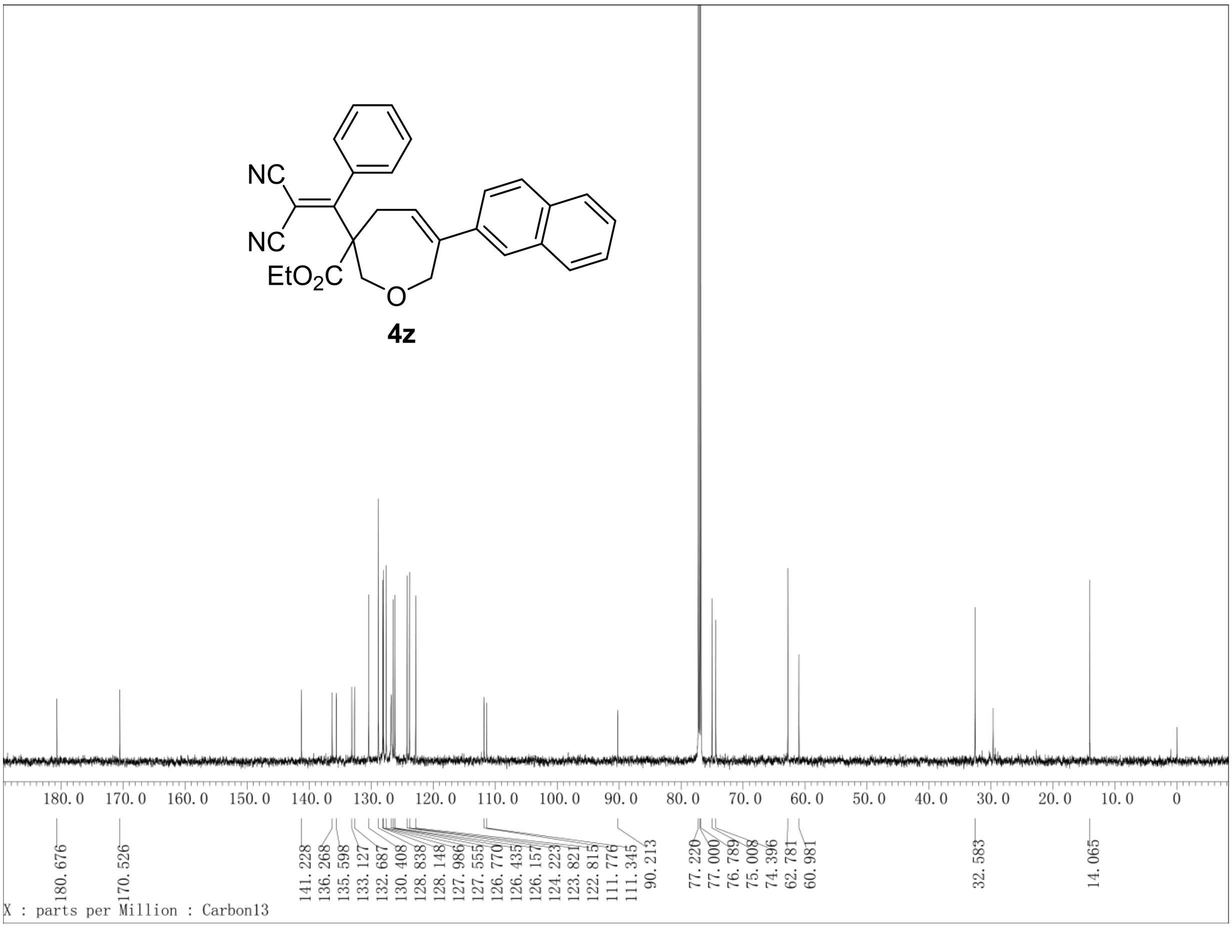
NC
NC
EtO2C
O
4z
180.0 170.0 160.0 150.0 140.0 130.0 120.0 110.0 100.0 90.0 80.0 70.0 60.0 50.0 40.0 30.0 20.0 10.0 0
180.676 170.526 141.228 136.268 135.598 133.127 132.687 130.408 128.838 128.148 127.986 127.555 126.770 126.435 126.157 124.223 123.821 122.815 111.776 111.345 90.213 77.220 77.000 76.789 75.008 74.396 62.781 60.981 32.583 14.065
X : parts per Million : Carbon13

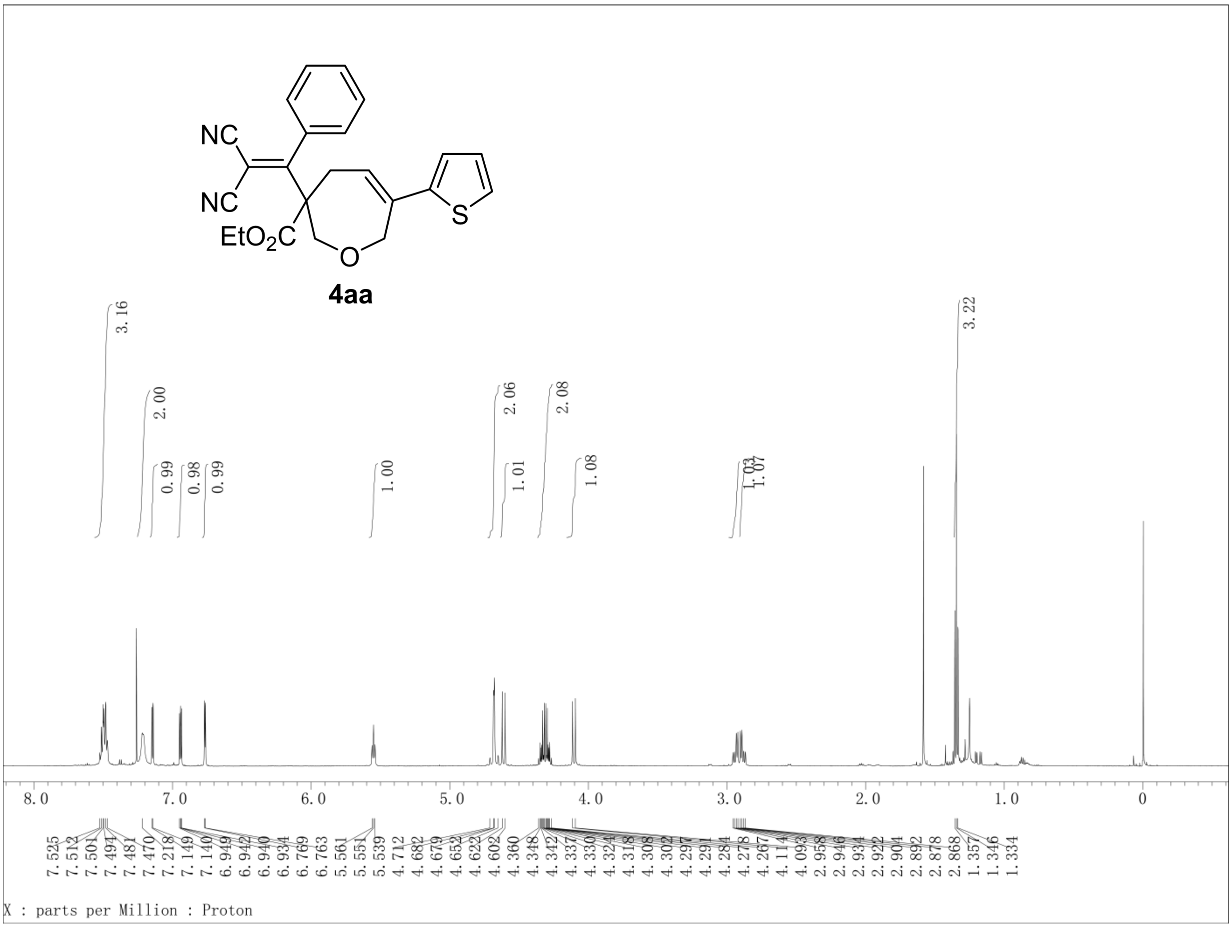
NC
NC
EtO2C
S
O
4aa
3.16
2.00
0.99
0.98
0.99
1.00
2.06
1.01
2.08
1.08
1.03
1.07
3.22
8.0 7.0 6.0 5.0 4.0 3.0 2.0 1.0 0
7.525 7.512 7.501 7.494 7.481 7.470 7.218 7.149 7.140 6.949 6.942 6.940 6.934 6.769 6.763 5.561 5.551 5.539 4.712 4.682 4.679 4.652 4.622 4.602 4.360 4.348 4.342 4.337 4.330 4.324 4.318 4.308 4.302 4.297 4.291 4.284 4.278 4.267 4.114 4.093 2.958 2.946 2.934 2.922 2.904 2.892 2.878 2.868 1.357 1.346 1.334
X : parts per Million : Proton

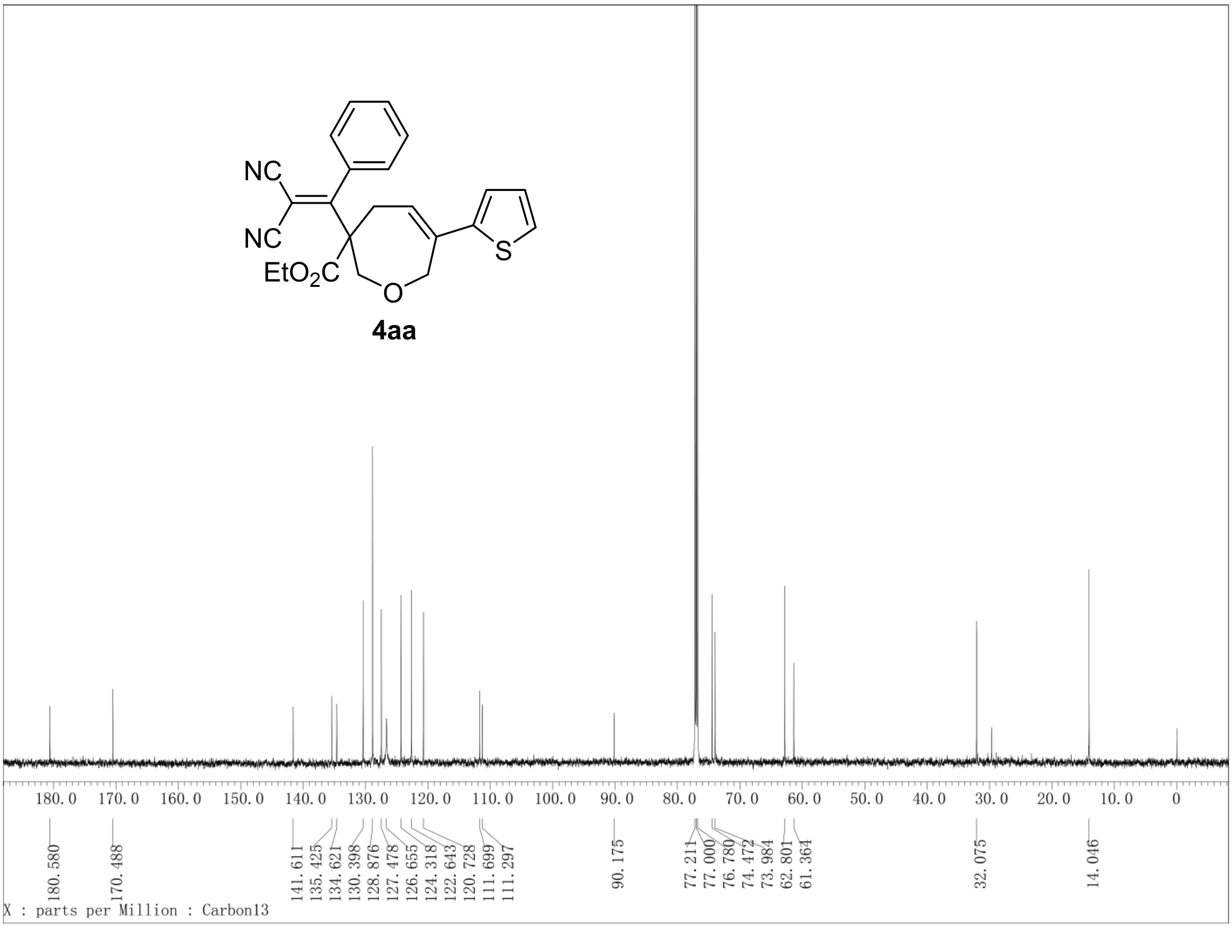
NC
NC
EtO2C
S
O
4aa
180.0 170.0 160.0 150.0 140.0 130.0 120.0 110.0 100.0 90.0 80.0 70.0 60.0 50.0 40.0 30.0 20.0 10.0 0
180.580 170.488 141.611 135.425 134.621 130.398 128.876 127.478 126.655 124.318 122.643 120.728 111.699 111.297 90.175 77.211 77.000 76.780 74.472 73.984 62.801 61.364 32.075 14.046
X : parts per Million : Carbon13

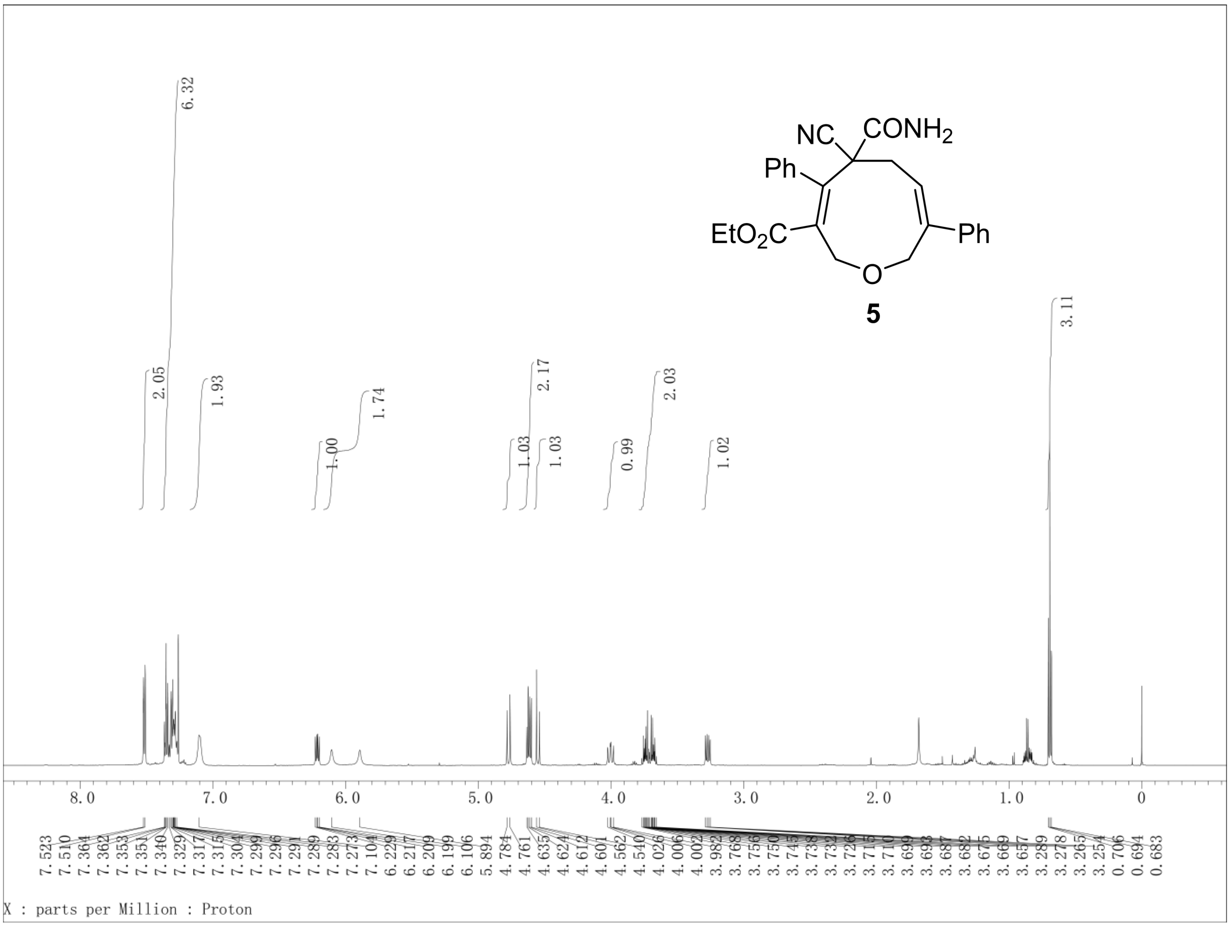
NC CONH2
Ph
EtO2C
Ph
O
5
2.05
6.32
1.93
1.00
1.74
1.03
2.17
1.03
0.99
2.03
1.02
3.11
8.0 7.0 6.0 5.0 4.0 3.0 2.0 1.0 0
7.523 7.510 7.364 7.362 7.353 7.351 7.340 7.329 7.317 7.315 7.304 7.299 7.296 7.291 7.289 7.283 7.273 7.104 6.229 6.217 6.209 6.199 6.106 5.894 4.784 4.761 4.635 4.624 4.612 4.601 4.562 4.540 4.026 4.006 4.002 3.982 3.768 3.756 3.750 3.745 3.738 3.733 3.726 3.715 3.710 3.699 3.693 3.687 3.682 3.675 3.669 3.657 3.289 3.278 3.265 3.254 0.706 0.694 0.683
X : parts per Million : Proton

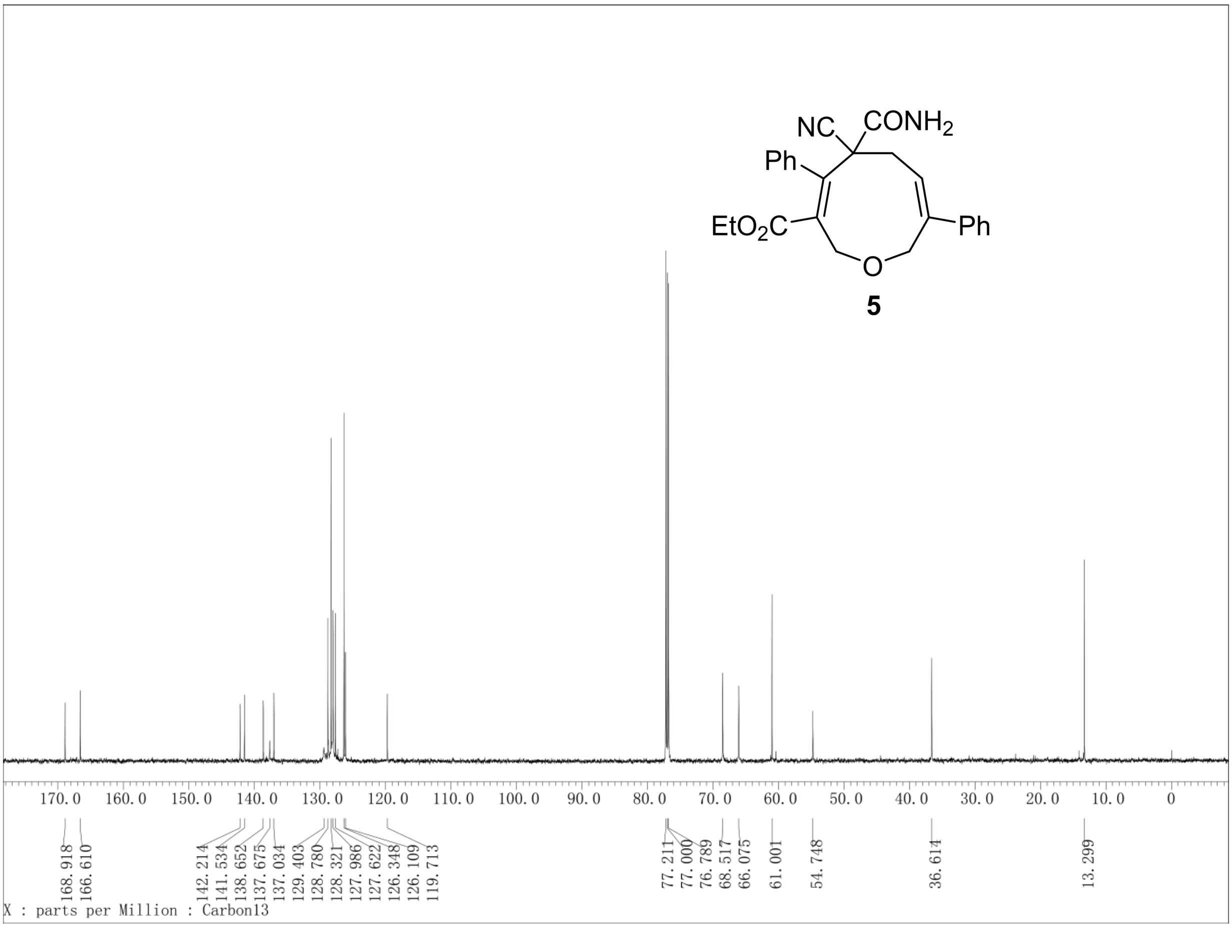
NC CONH2
Ph
EtO2C
Ph
O
5
170.0 160.0 150.0 140.0 130.0 120.0 110.0 100.0 90.0 80.0 70.0 60.0 50.0 40.0 30.0 20.0 10.0 0
168.918 166.610 142.214 141.534 138.652 137.675 137.034 129.403 128.780 128.321 127.986 127.622 126.348 126.109 119.713 77.211 77.000 76.789 68.517 66.075 61.001 54.748 36.614 13.299
X : parts per Million : Carbon13

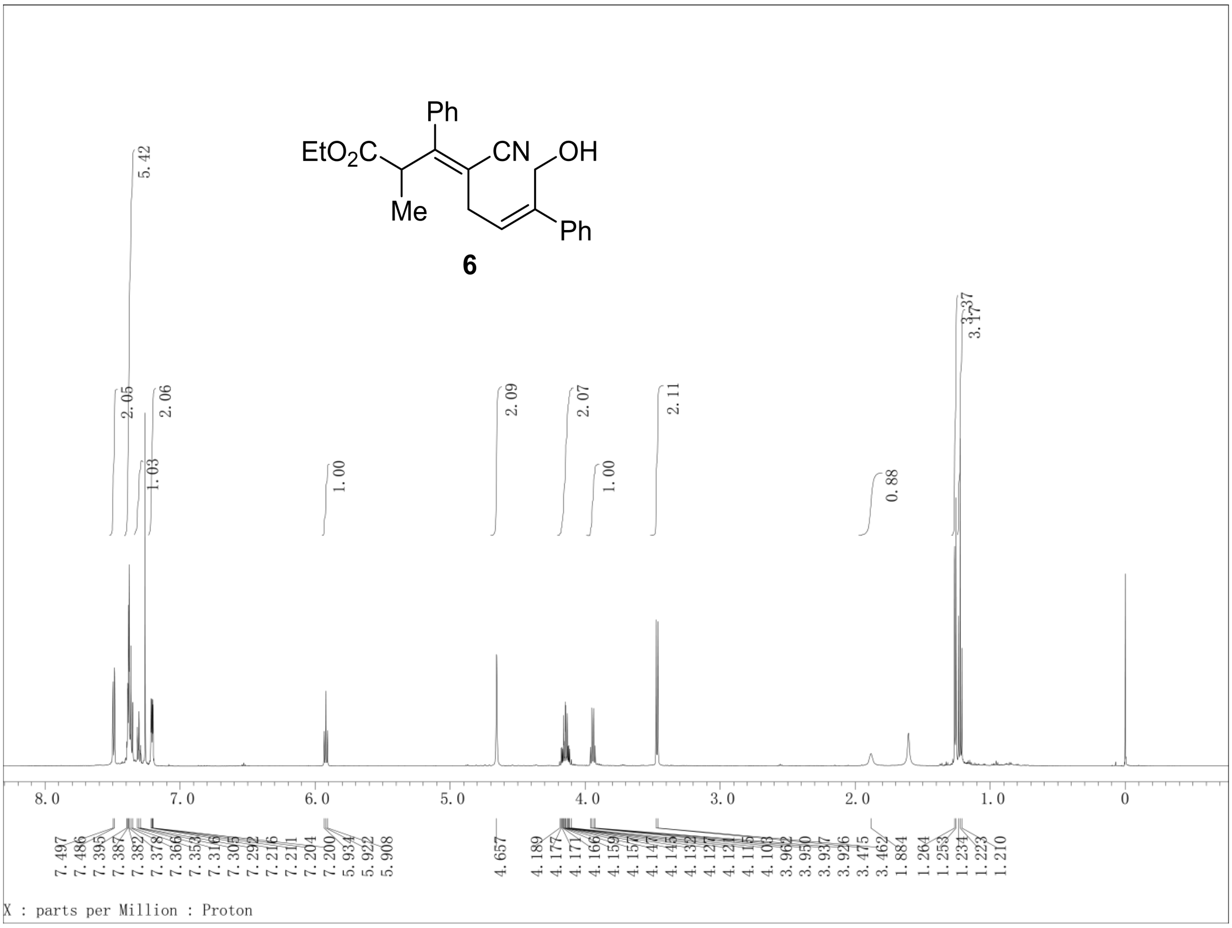
Ph
EtO2C
CN
OH
Me
Ph
6
5.42
2.05
2.06
1.03
1.00
2.09
2.07
1.00
2.11
0.88
3.37
3.17
8.0 7.0 6.0 5.0 4.0 3.0 2.0 1.0 0
7.497 7.486 7.395 7.387 7.382 7.378 7.366 7.353 7.316 7.305 7.292 7.216 7.211 7.204 7.200 5.934 5.922 5.908
4.657
4.189 4.177 4.171 4.166 4.159 4.157 4.147 4.145 4.132 4.127 4.121 4.115 4.103 3.962 3.950 3.937 3.926 3.475 3.462 1.884 1.264 1.253 1.234 1.223 1.210
X : parts per Million : Proton

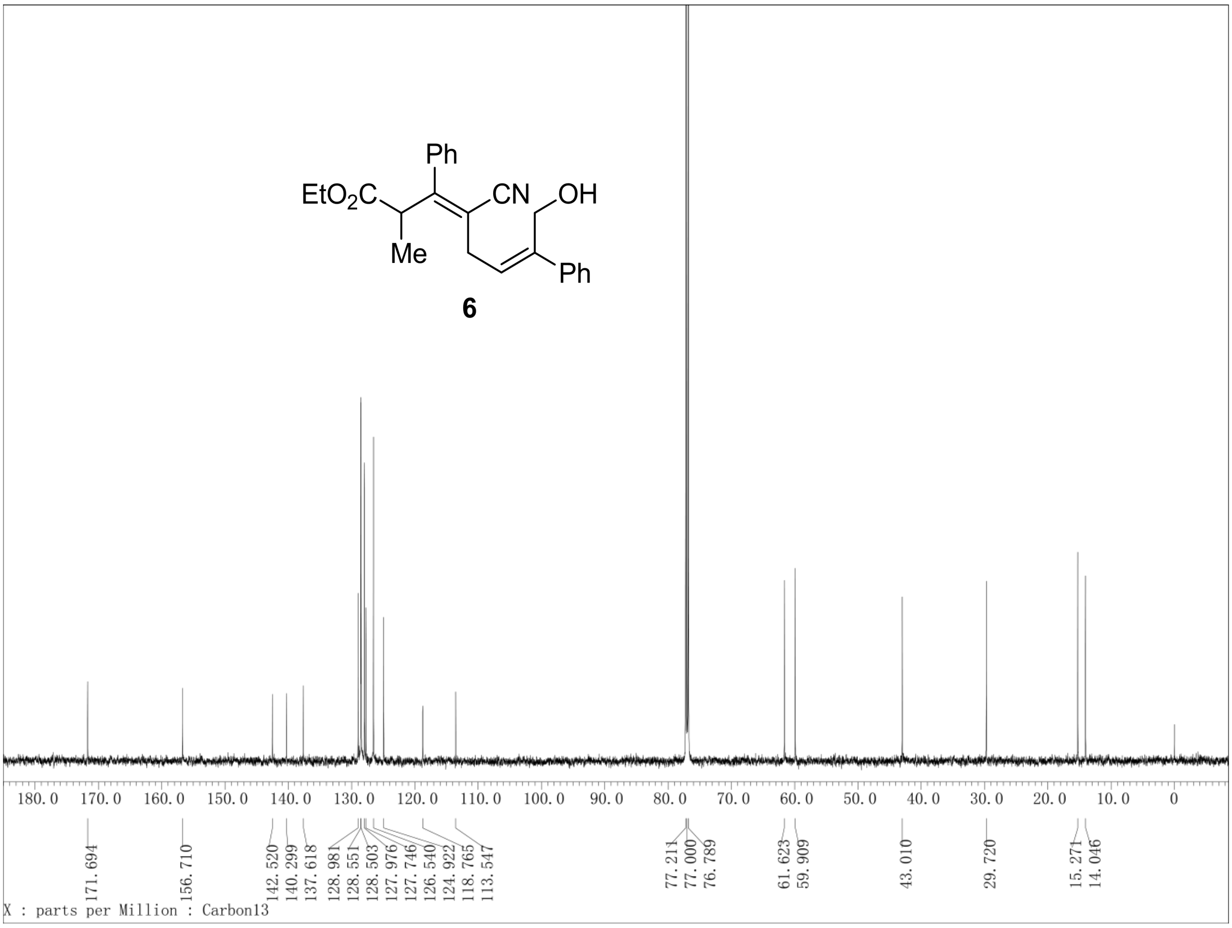
Ph
EtO2C
CN
OH
Me
Ph
6
180.0 170.0 160.0 150.0 140.0 130.0 120.0 110.0 100.0 90.0 80.0 70.0 60.0 50.0 40.0 30.0 20.0 10.0 0
171.694
156.710
142.520 140.299 137.618 128.981 128.551 128.503 127.976 127.746 126.540 124.922 118.765 113.547
77.211 77.000 76.789
61.623 59.909
43.010
29.720
15.271 14.046
X : parts per Million : Carbon13

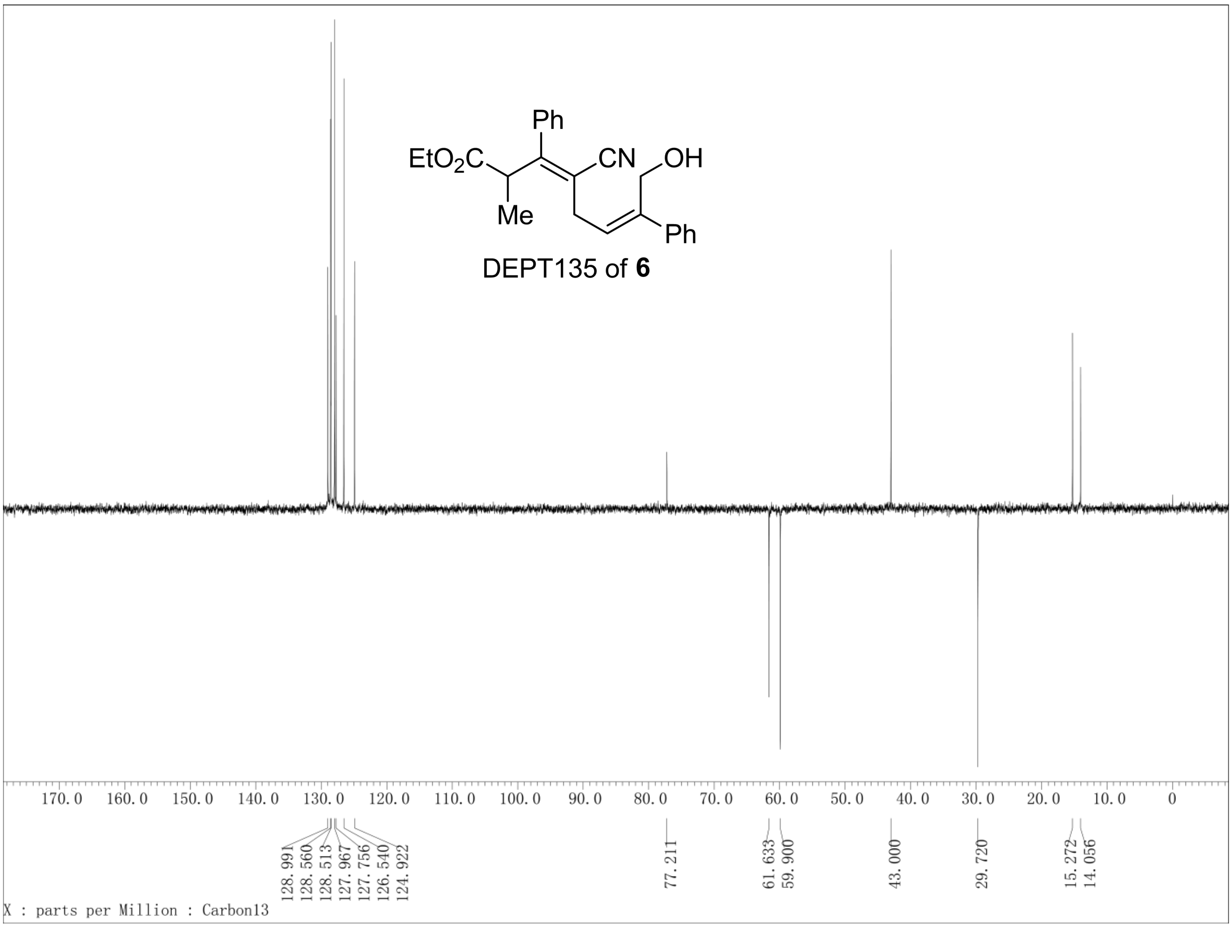
Ph
EtO2C
CN
OH
Me
Ph
DEPT135 of 6
170.0 160.0 150.0 140.0 130.0 120.0 110.0 100.0 90.0 80.0 70.0 60.0 50.0 40.0 30.0 20.0 10.0 0
128.991
128.560
128.513
127.967
127.756
126.540
124.922
77.211
61.633
59.900
43.000
29.720
15.272
14.056
X : parts per Million : Carbon13

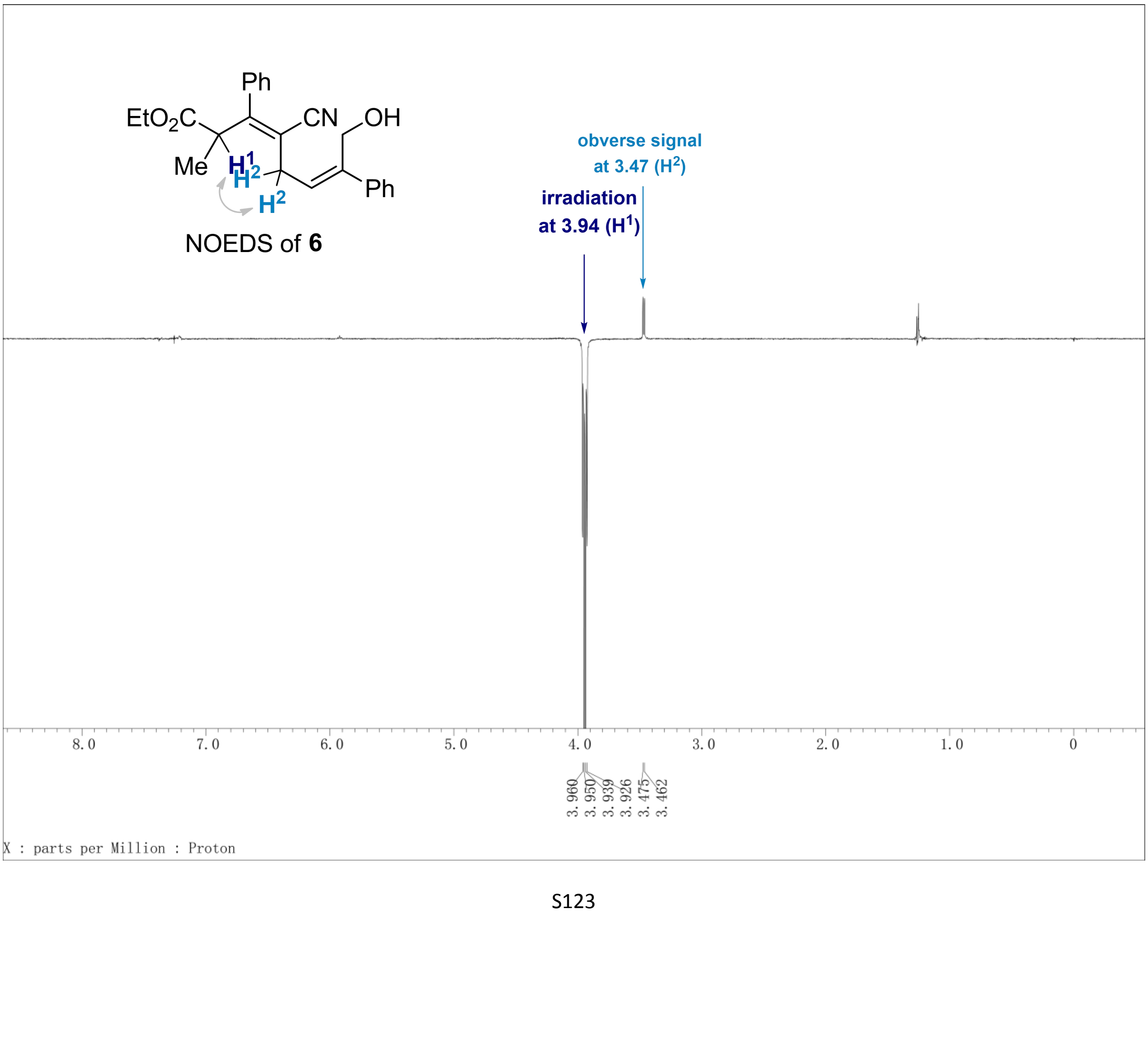
Ph
EtO2C
CN
OH
Me
H1
H2
H2
Ph
NOEDS of 6
obverse signal
at 3.47 (H2)
irradiation
at 3.94 (H1)
8.0 7.0 6.0 5.0 4.0 3.0 2.0 1.0 0
3.960
3.950
3.939
3.926
3.475
3.462
X : parts per Million : Proton

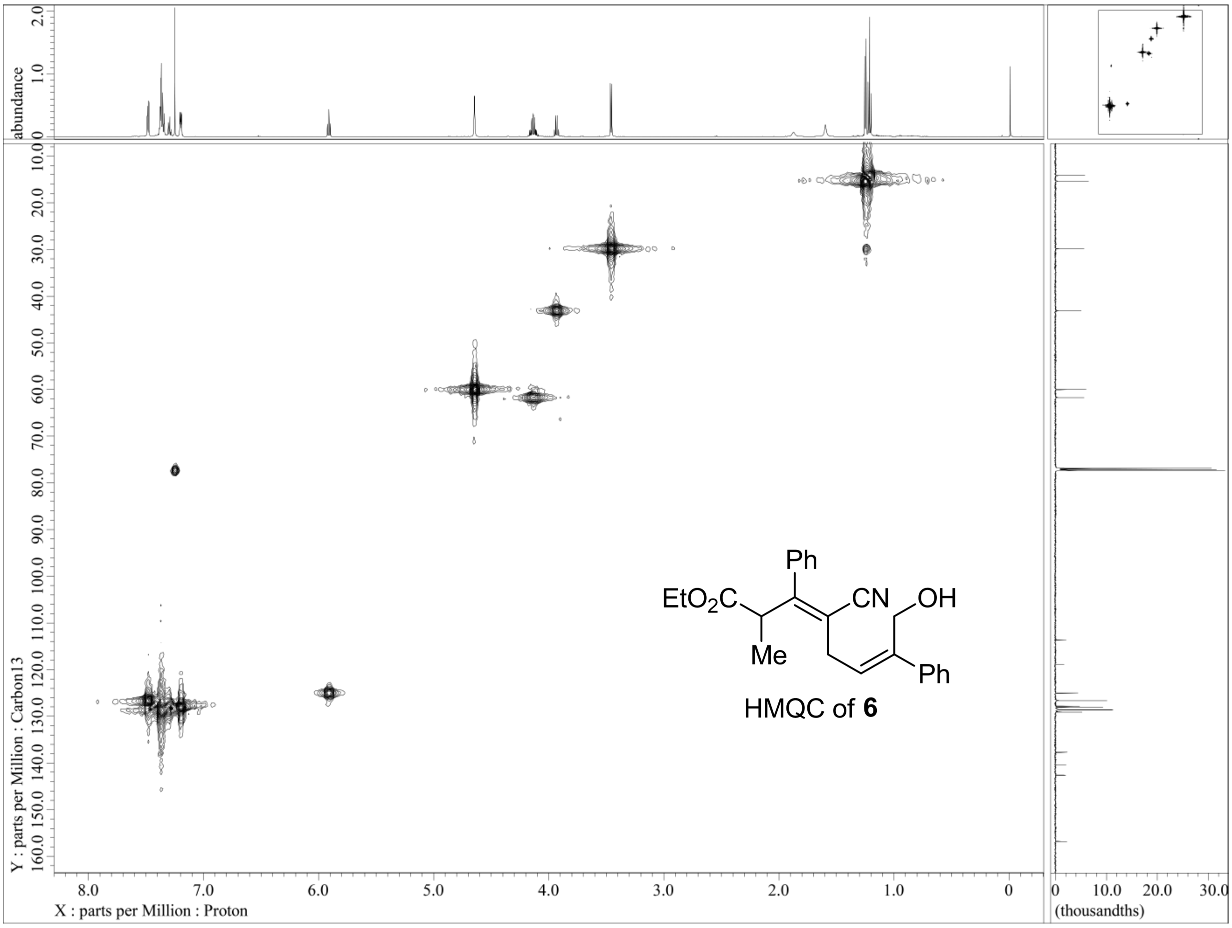
abundance
Y : parts per Million : Carbon13
X : parts per Million : Proton
(thousandths)
Ph
EtO2C
CN
OH
Me
Ph
HMQC of 6

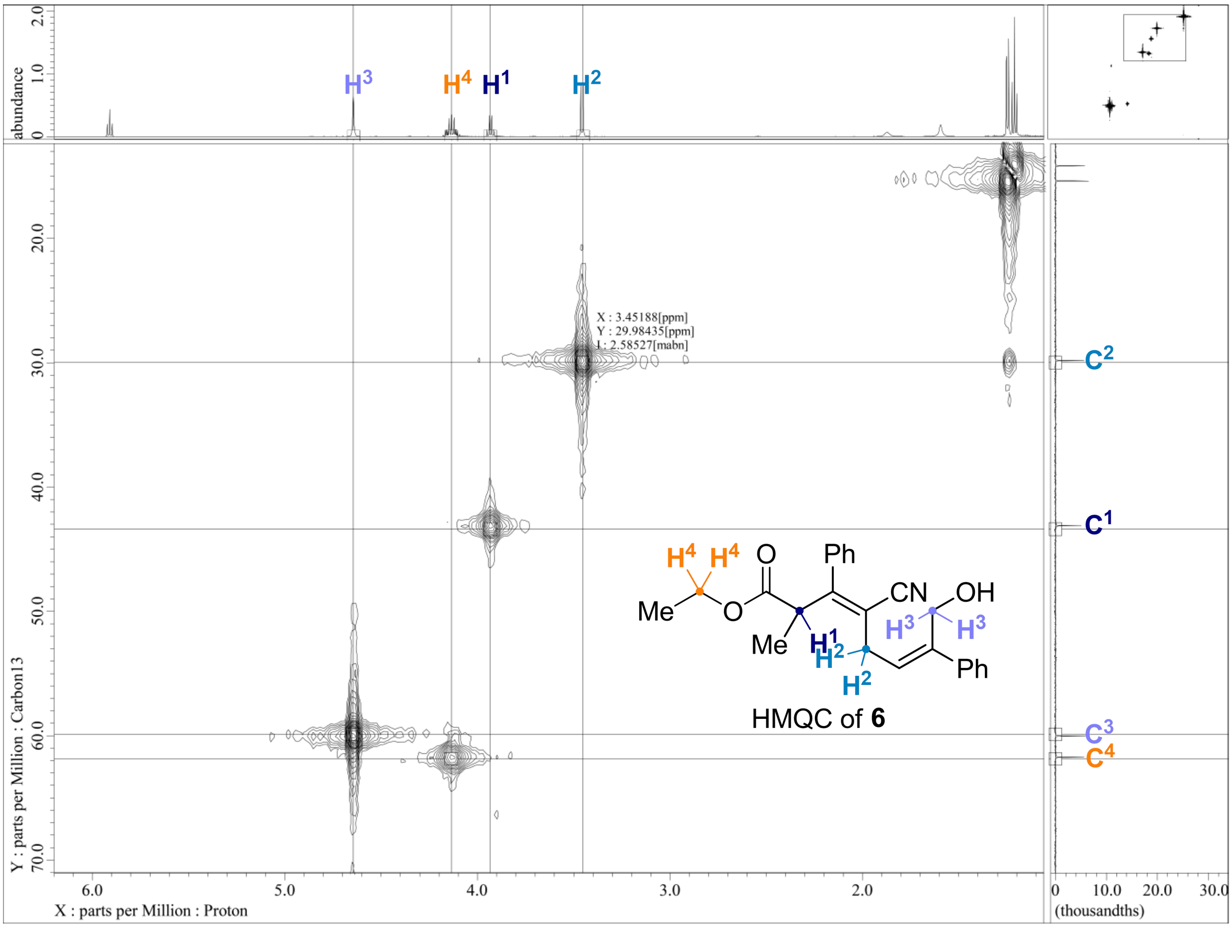
abundance
H3
H4
H1
H2
X : 3.45188[ppm]
Y : 29.98435[ppm]
I : 2.58527[mabn]
C2
C1
C3
C4
H4
H4
O
Ph
CN
OH
Me
O
H3
H3
Me
H1
H2
H2
Ph
HMQC of 6
Y : parts per Million : Carbon13
X : parts per Million : Proton
(thousandths)

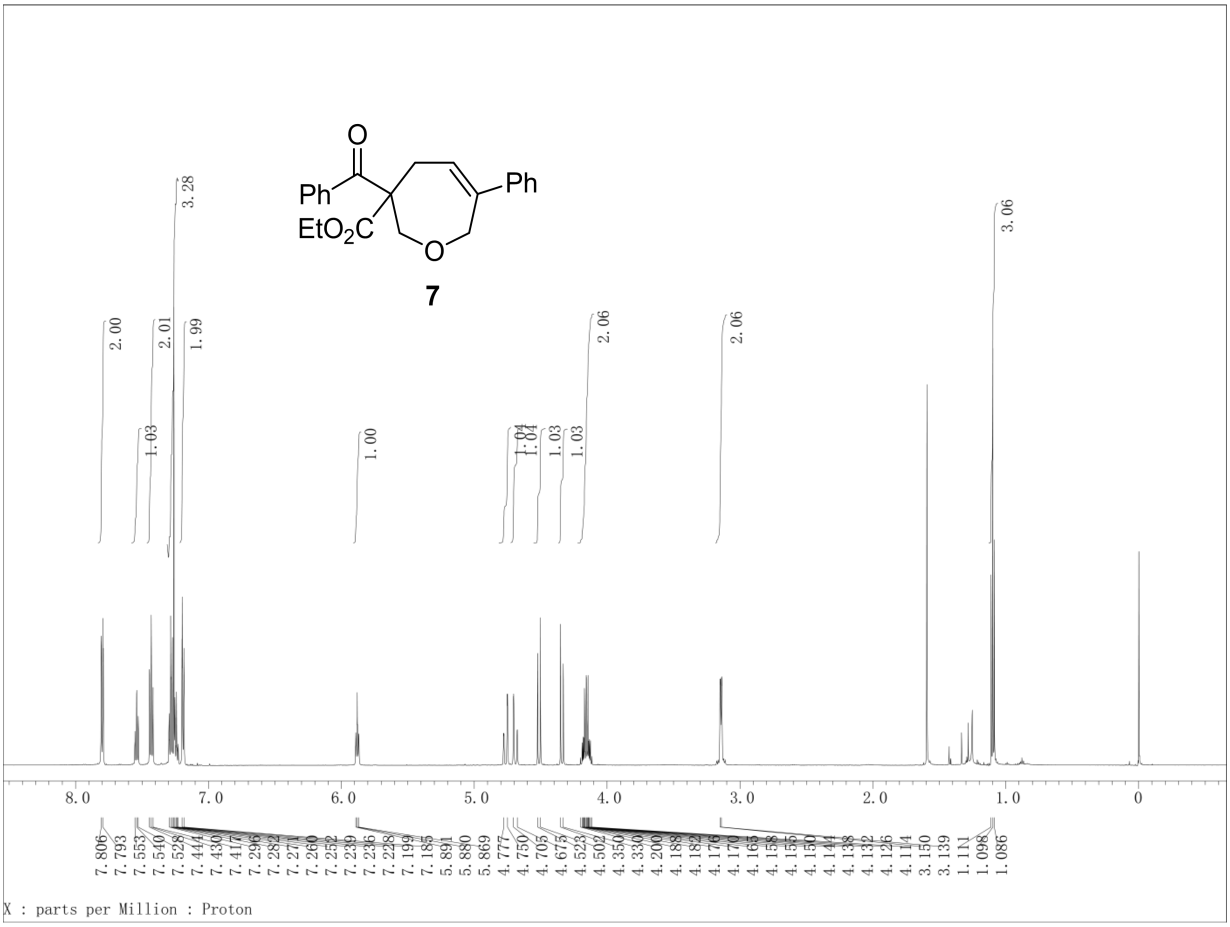
Ph
EtO2C
O
Ph
7
2.00
1.03
2.01
3.28
1.99
1.00
1.04
1.04
1.03
1.03
2.06
2.06
3.06
8.0
7.0
6.0
5.0
4.0
3.0
2.0
1.0
0
7.806
7.793
7.553
7.540
7.528
7.444
7.430
7.417
7.296
7.282
7.271
7.260
7.252
7.239
7.236
7.228
7.199
7.185
5.891
5.880
5.869
4.777
4.750
4.705
4.675
4.523
4.502
4.350
4.330
4.200
4.188
4.182
4.176
4.170
4.165
4.158
4.155
4.150
4.144
4.138
4.132
4.126
4.114
3.150
3.139
1.111
1.098
1.086
X : parts per Million : Proton

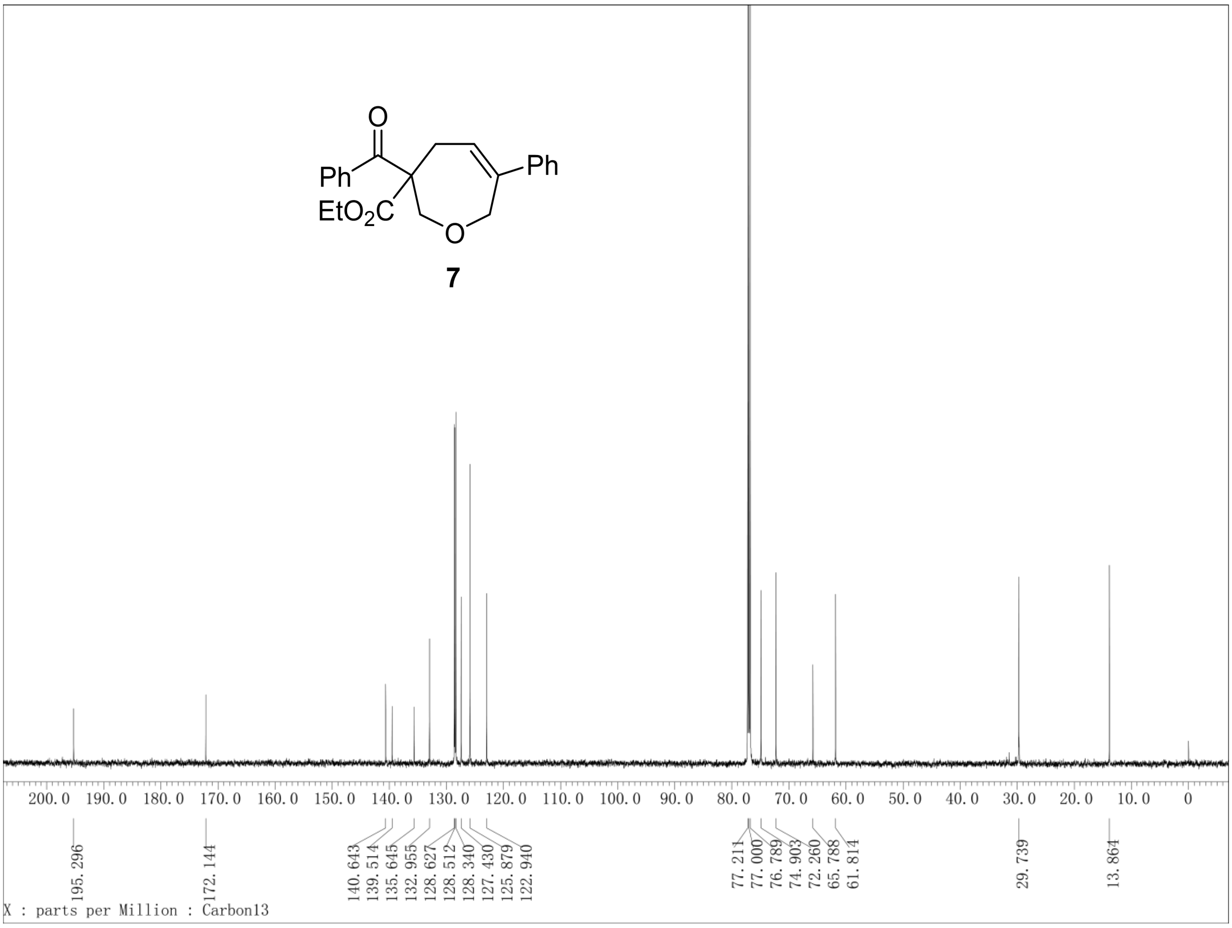
Ph
EtO2C
O
Ph
7
200.0
190.0
180.0
170.0
160.0
150.0
140.0
130.0
120.0
110.0
100.0
90.0
80.0
70.0
60.0
50.0
40.0
30.0
20.0
10.0
0
195.296
172.144
140.643
139.514
135.645
132.955
128.627
128.512
128.340
127.430
125.879
122.940
77.211
77.000
76.789
74.903
72.260
65.788
61.814
29.739
13.864
X : parts per Million : Carbon13

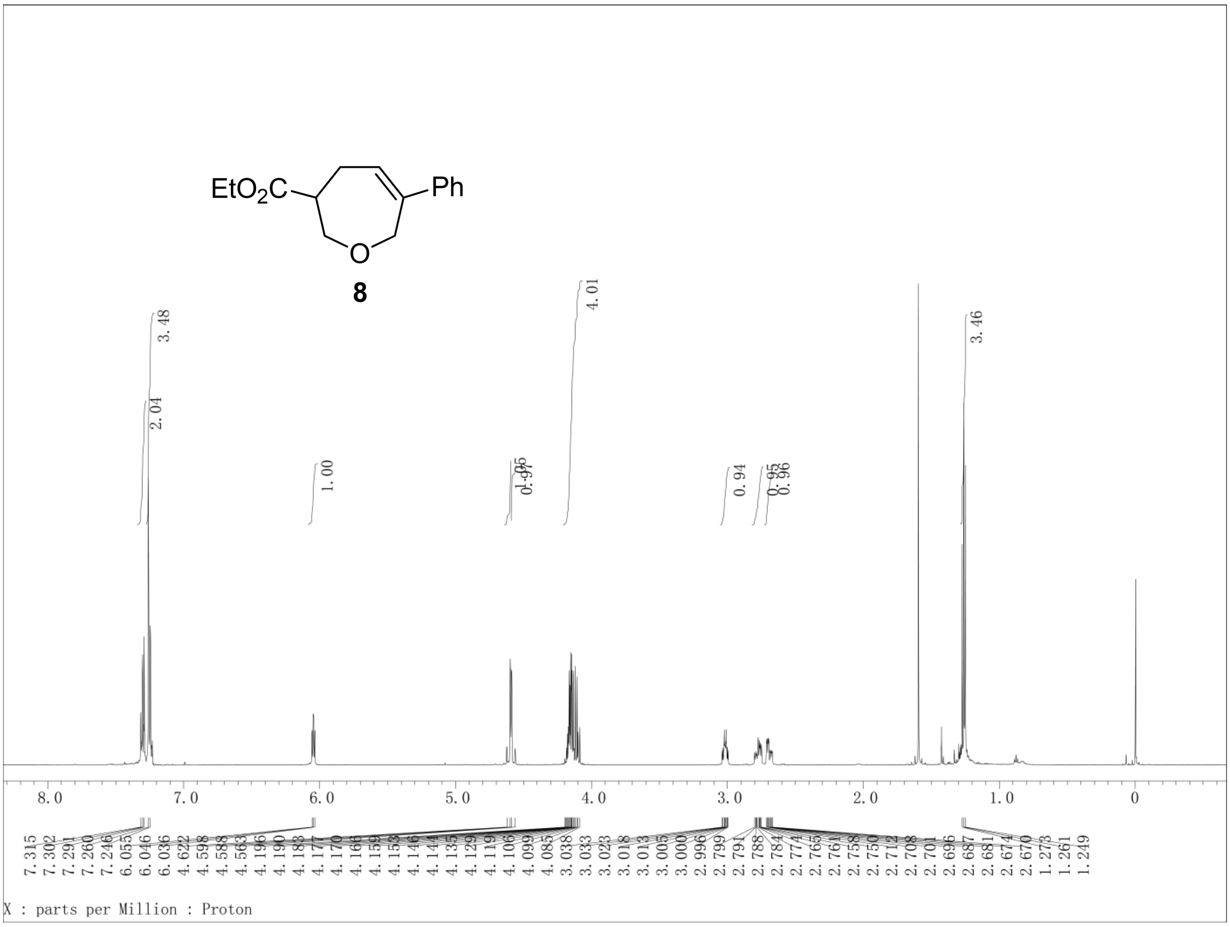
EtO2C
Ph
O
8
2.04
3.48
1.00
4.01
0.94
0.95
0.96
3.46
8.0
7.0
6.0
5.0
4.0
3.0
2.0
1.0
0
7.315
7.302
7.291
7.260
7.246
6.055
6.046
6.036
4.622
4.598
4.588
4.563
4.196
4.190
4.183
4.177
4.170
4.165
4.159
4.153
4.146
4.144
4.135
4.129
4.119
4.106
4.099
4.085
3.038
3.033
3.023
3.018
3.013
3.005
3.000
2.996
2.799
2.791
2.788
2.784
2.774
2.765
2.761
2.758
2.750
2.712
2.708
2.701
2.696
2.687
2.681
2.674
2.670
1.273
1.261
1.249
X : parts per Million : Proton

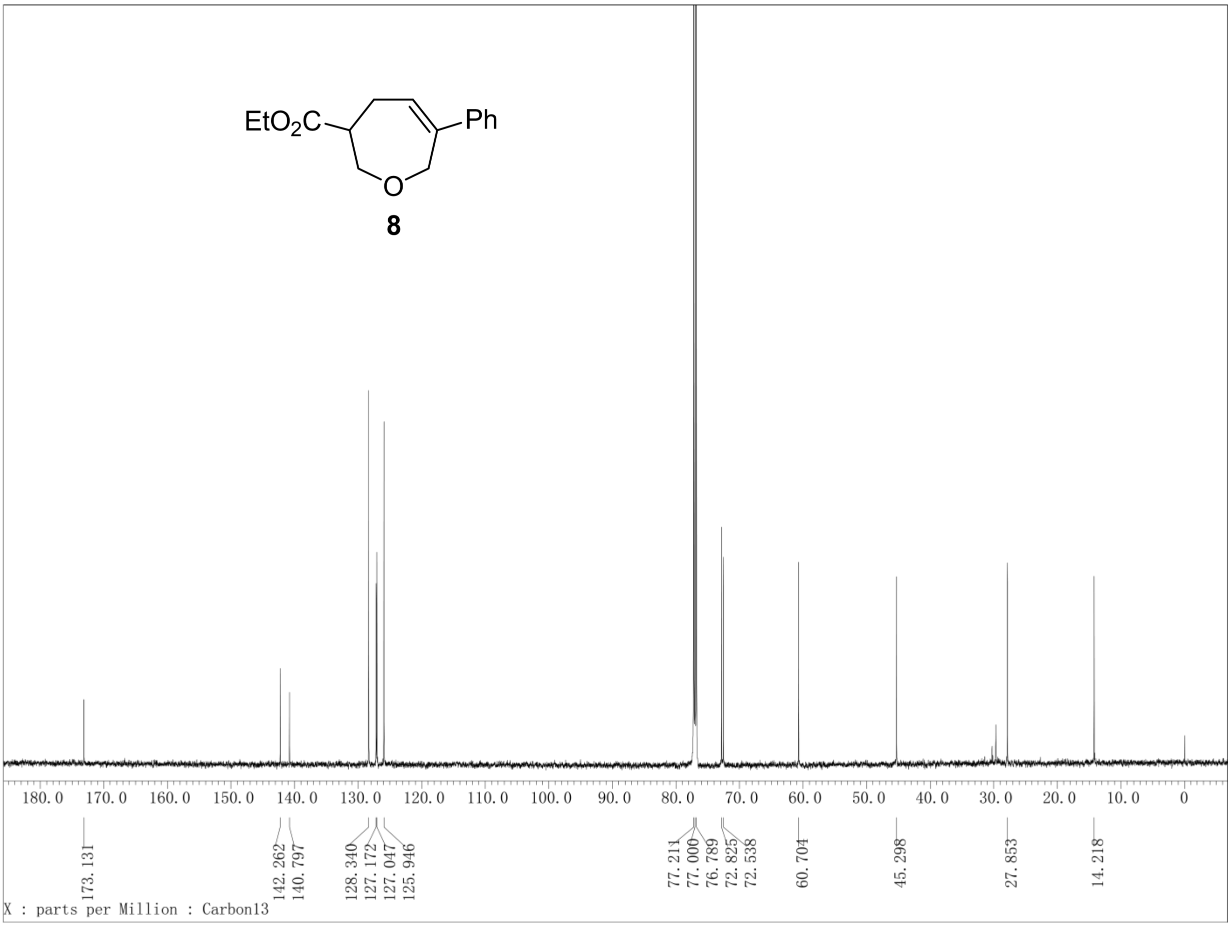
EtO2C
Ph
O
8
180.0
170.0
160.0
150.0
140.0
130.0
120.0
110.0
100.0
90.0
80.0
70.0
60.0
50.0
40.0
30.0
20.0
10.0
0
173.131
142.262
140.797
128.340
127.172
127.047
125.946
77.211
77.000
76.789
72.825
72.538
60.704
45.298
27.853
14.218
X : parts per Million : Carbon13

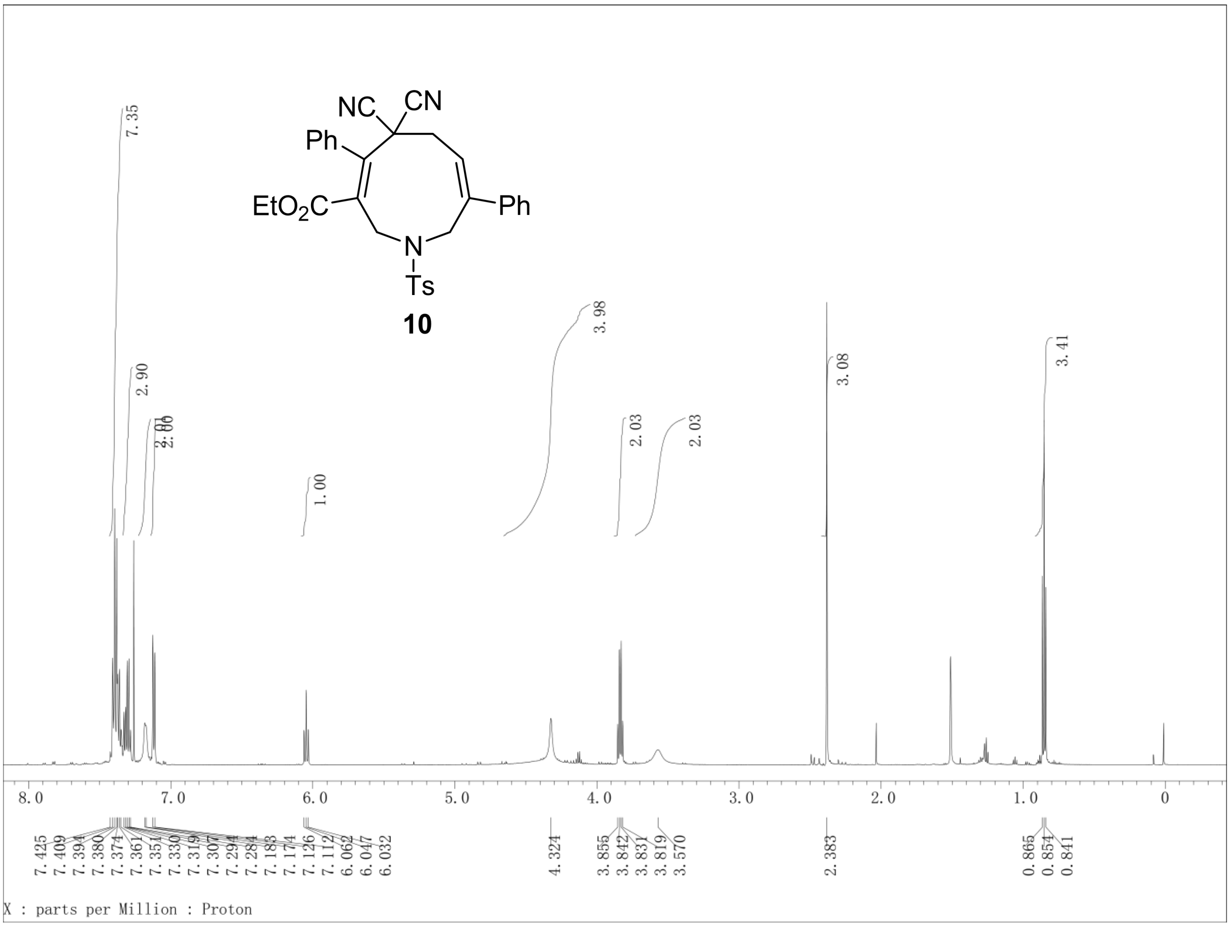
NC
CN
Ph
EtO2C
Ph
N
Ts
10
7.35
2.90
2.01
2.00
1.00
3.98
2.03
2.03
3.08
3.41
8.0
7.0
6.0
5.0
4.0
3.0
2.0
1.0
0
7.425
7.409
7.394
7.380
7.374
7.361
7.351
7.330
7.319
7.307
7.294
7.284
7.183
7.174
7.126
7.112
6.062
6.047
6.032
4.324
3.855
3.842
3.831
3.819
3.570
2.383
0.865
0.854
0.841
X : parts per Million : Proton

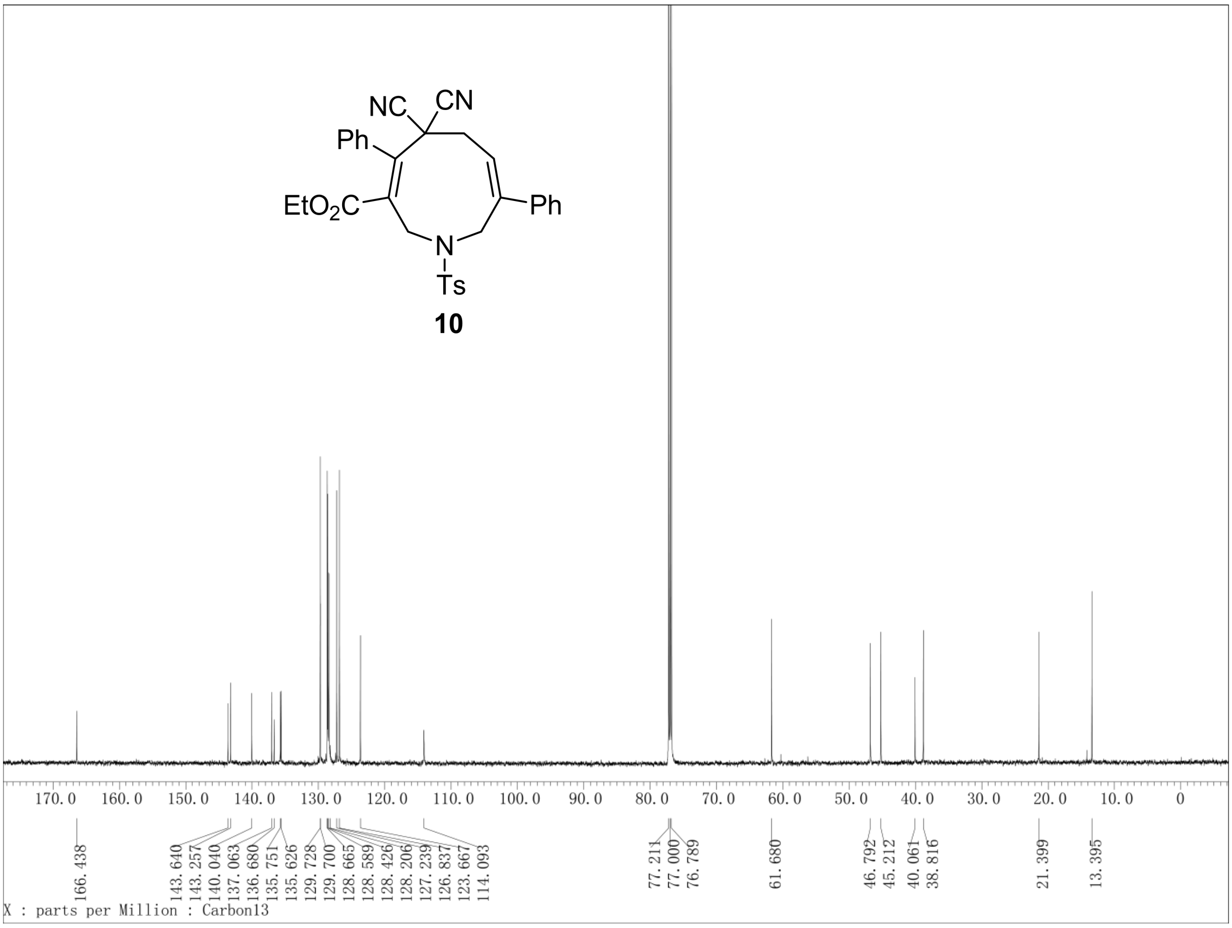
NC
CN
Ph
EtO2C
Ph
N
Ts
10
170.0
160.0
150.0
140.0
130.0
120.0
110.0
100.0
90.0
80.0
70.0
60.0
50.0
40.0
30.0
20.0
10.0
0
166.438
143.640
143.257
140.040
137.063
136.680
135.751
135.626
129.728
129.700
128.665
128.589
128.426
128.206
127.239
126.837
123.667
114.093
77.211
77.000
76.789
61.680
46.792
45.212
40.061
38.816
21.399
13.395
X : parts per Million : Carbon13

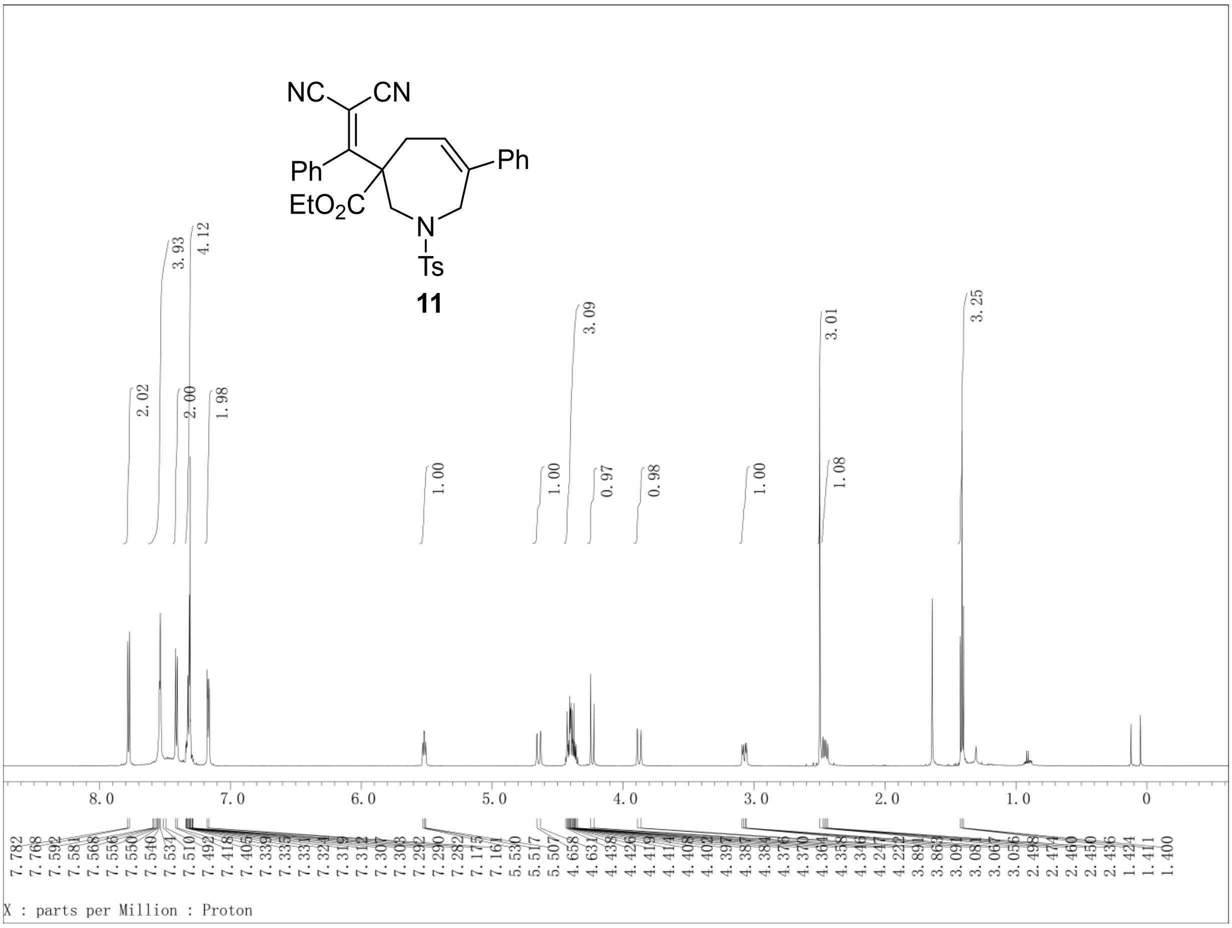
NC
CN
Ph
Ph
EtO2C
N
Ts
11
X : parts per Million : Proton

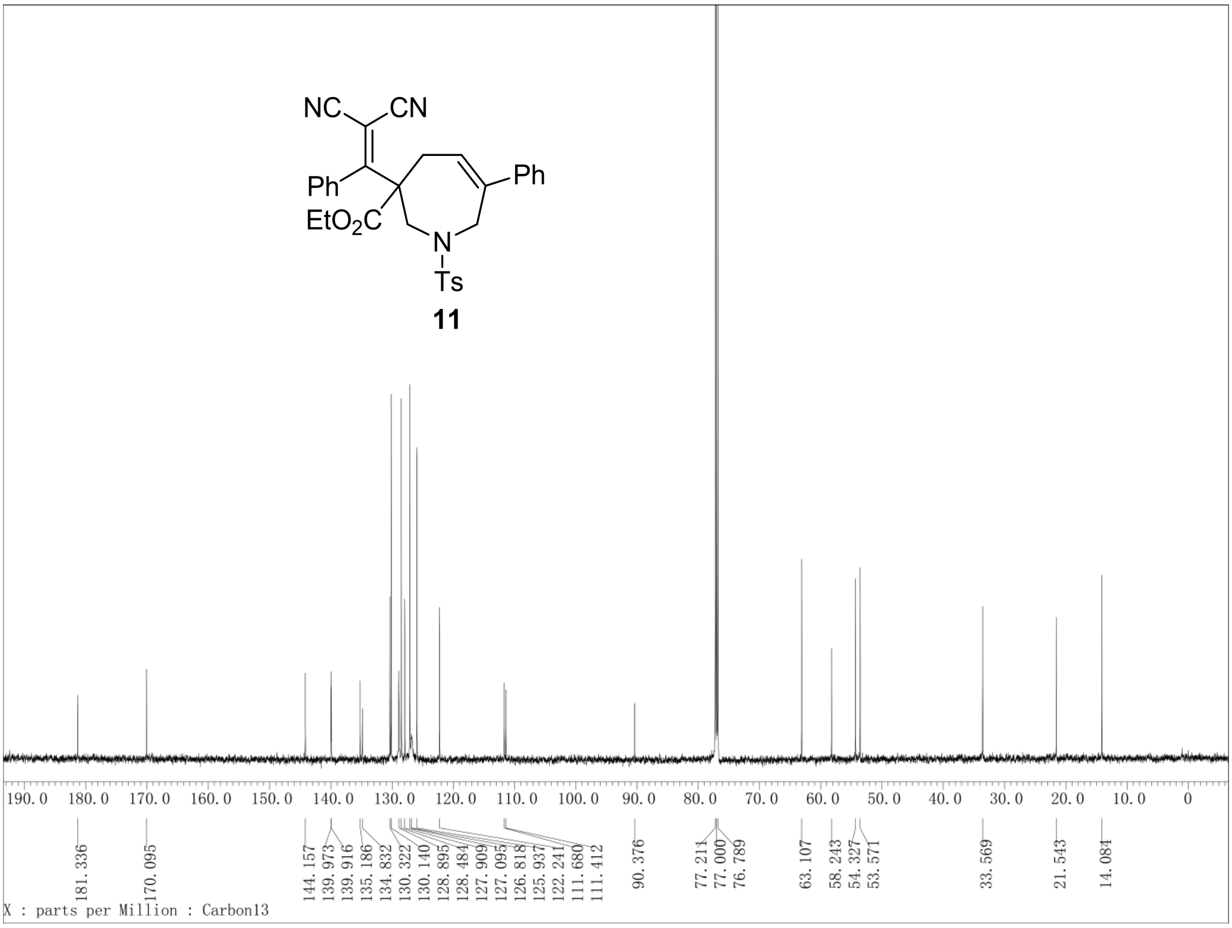
NC
CN
Ph
Ph
EtO2C
N
Ts
11
X : parts per Million : Carbon13

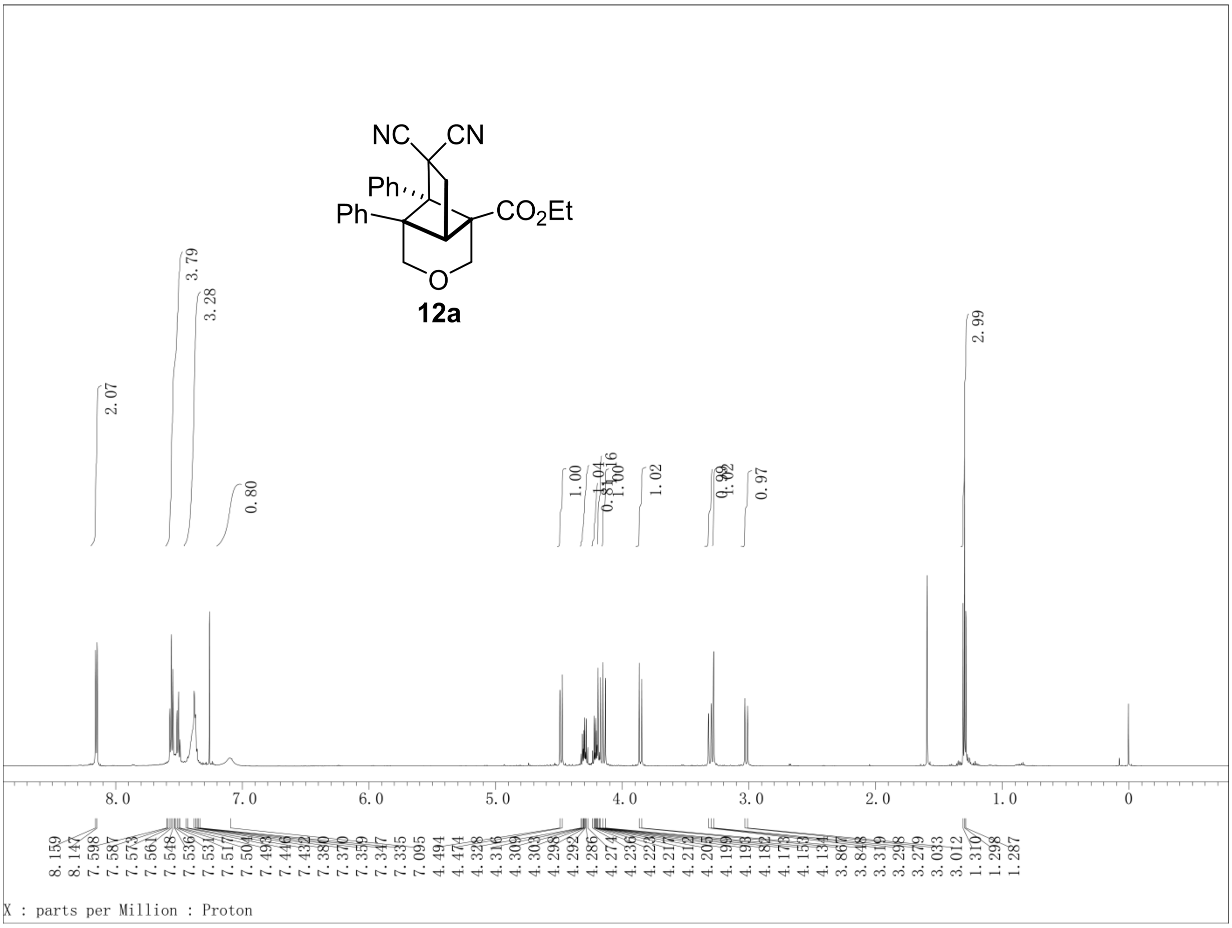
NC
CN
Ph
Ph
CO2Et
O
12a
2.07
3.79
3.28
0.80
1.00
1.02
0.97
2.99
8.0
7.0
6.0
5.0
4.0
3.0
2.0
1.0
0
8.159
8.147
7.598
7.587
7.573
7.561
7.548
7.536
7.531
7.517
7.504
7.493
7.446
7.432
7.380
7.370
7.359
7.347
7.335
7.095
4.494
4.474
4.328
4.316
4.309
4.303
4.298
4.292
4.286
4.274
4.236
4.223
4.217
4.212
4.205
4.199
4.193
4.182
4.173
4.153
4.134
3.867
3.848
3.319
3.298
3.279
3.033
3.012
1.310
1.298
1.287
X : parts per Million : Proton

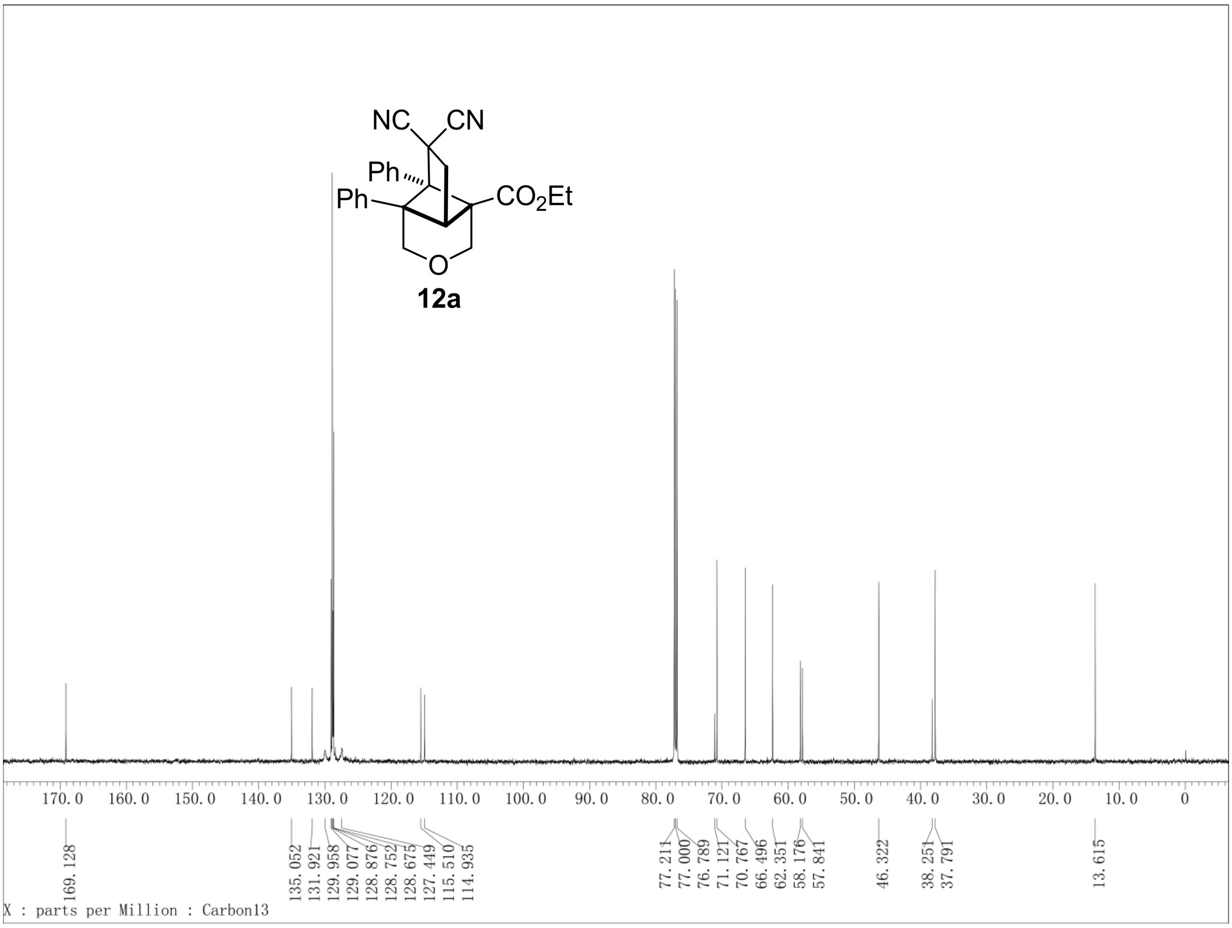
NC
CN
Ph
Ph
CO2Et
O
12a
170.0
160.0
150.0
140.0
130.0
120.0
110.0
100.0
90.0
80.0
70.0
60.0
50.0
40.0
30.0
20.0
10.0
0
169.128
135.052
131.921
129.958
129.077
128.876
128.752
128.675
127.449
115.510
114.935
77.211
77.000
76.789
71.121
70.767
66.496
62.351
58.176
57.841
46.322
38.251
37.791
13.615
X : parts per Million : Carbon13

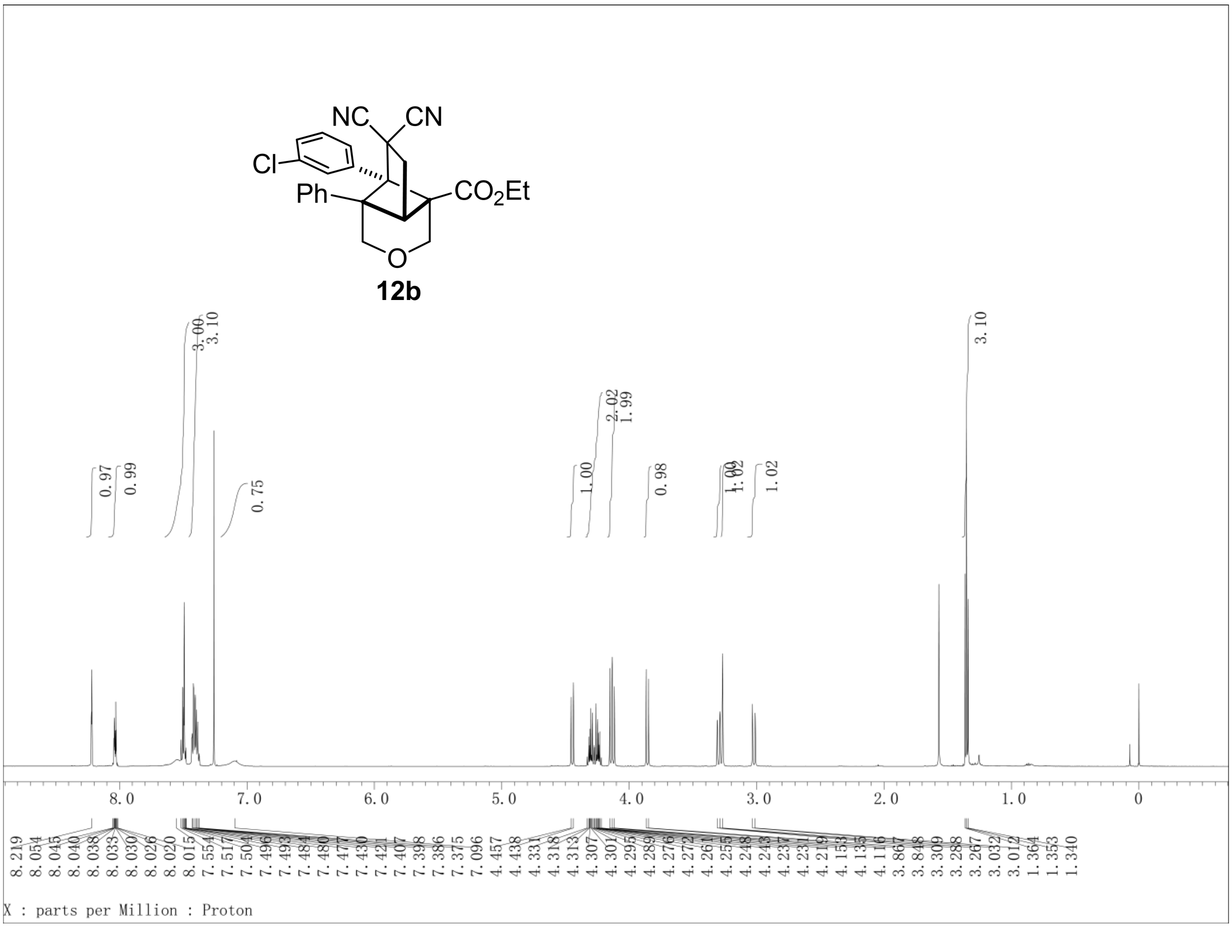
NC CN
Cl
Ph
CO2Et
O
12b
0.97 0.99 3.00 3.10 0.75 1.00 2.02 1.99 0.98 1.00 1.02 1.02 3.10
8.0 7.0 6.0 5.0 4.0 3.0 2.0 1.0 0
8.219 8.054 8.045 8.040 8.038 8.033 8.030 8.026 8.020 8.015 7.554 7.517 7.504 7.496 7.493 7.484 7.480 7.477 7.430 7.421 7.407 7.398 7.386 7.375 7.096 4.457 4.438 4.331 4.318 4.313 4.307 4.301 4.295 4.289 4.276 4.272 4.261 4.255 4.248 4.243 4.237 4.231 4.219 4.153 4.135 4.116 3.867 3.848 3.309 3.288 3.267 3.032 3.012 1.364 1.353 1.340
X : parts per Million : Proton

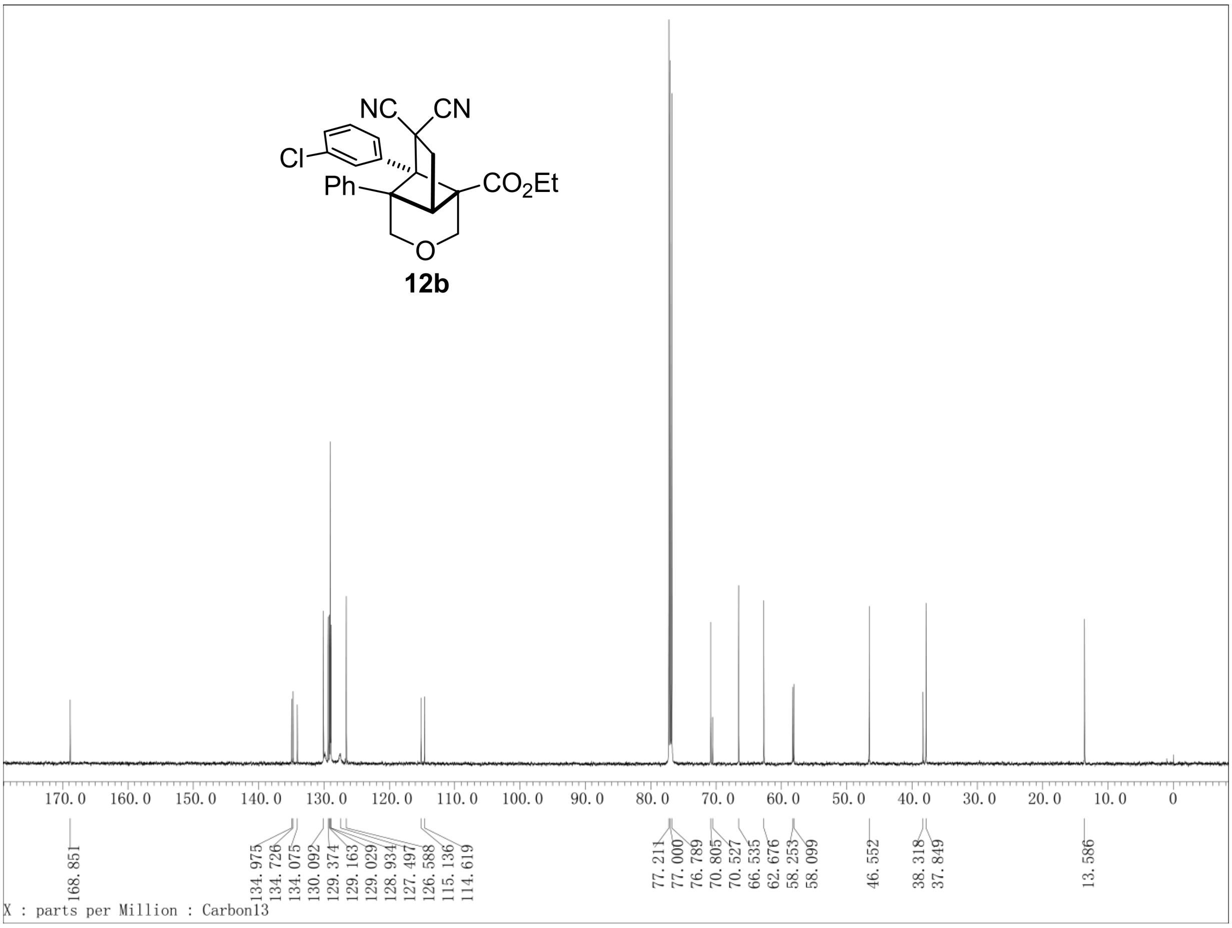
NC CN
Cl
Ph
CO2Et
O
12b
170.0 160.0 150.0 140.0 130.0 120.0 110.0 100.0 90.0 80.0 70.0 60.0 50.0 40.0 30.0 20.0 10.0 0
168.851 134.975 134.726 134.075 130.092 129.374 129.163 129.029 128.934 127.497 126.588 115.136 114.619 77.211 77.000 76.789 70.805 70.527 66.535 62.676 58.253 58.099 46.552 38.318 37.849 13.586
X : parts per Million : Carbon13

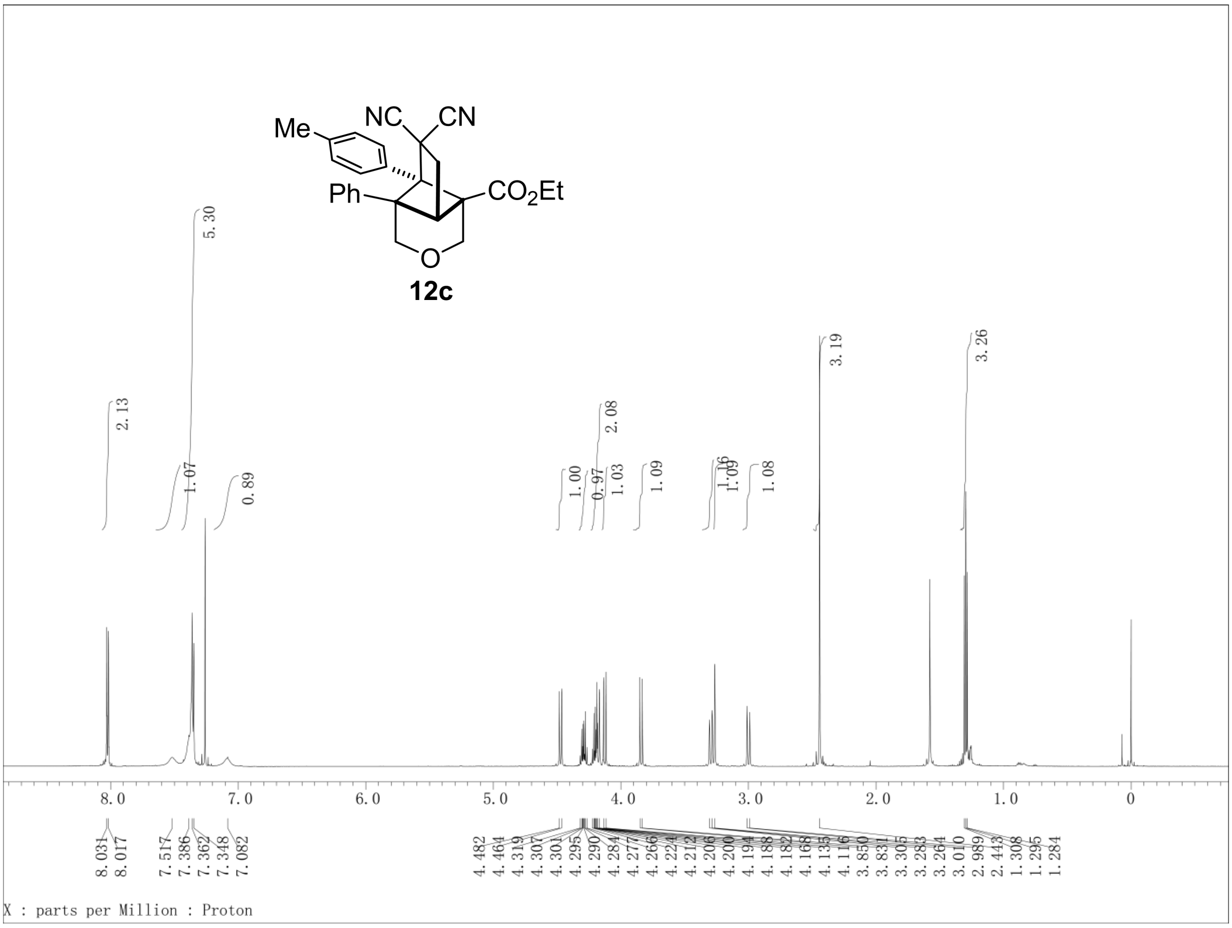

Me
NC
CN
Ph
CO_2Et
O
12c
5.30
2.13
1.07
0.89
1.00
0.97
2.08
1.03
1.09
1.16
1.09
1.08
3.19
3.26
8.0
7.0
6.0
5.0
4.0
3.0
2.0
1.0
0
8.031
8.017
7.517
7.386
7.362
7.348
7.082
4.482
4.464
4.319
4.307
4.301
4.295
4.290
4.284
4.277
4.266
4.224
4.212
4.206
4.200
4.194
4.188
4.182
4.168
4.135
4.116
3.850
3.831
3.305
3.283
3.264
3.010
2.989
2.443
1.308
1.295
1.284
X : parts per Million : Proton

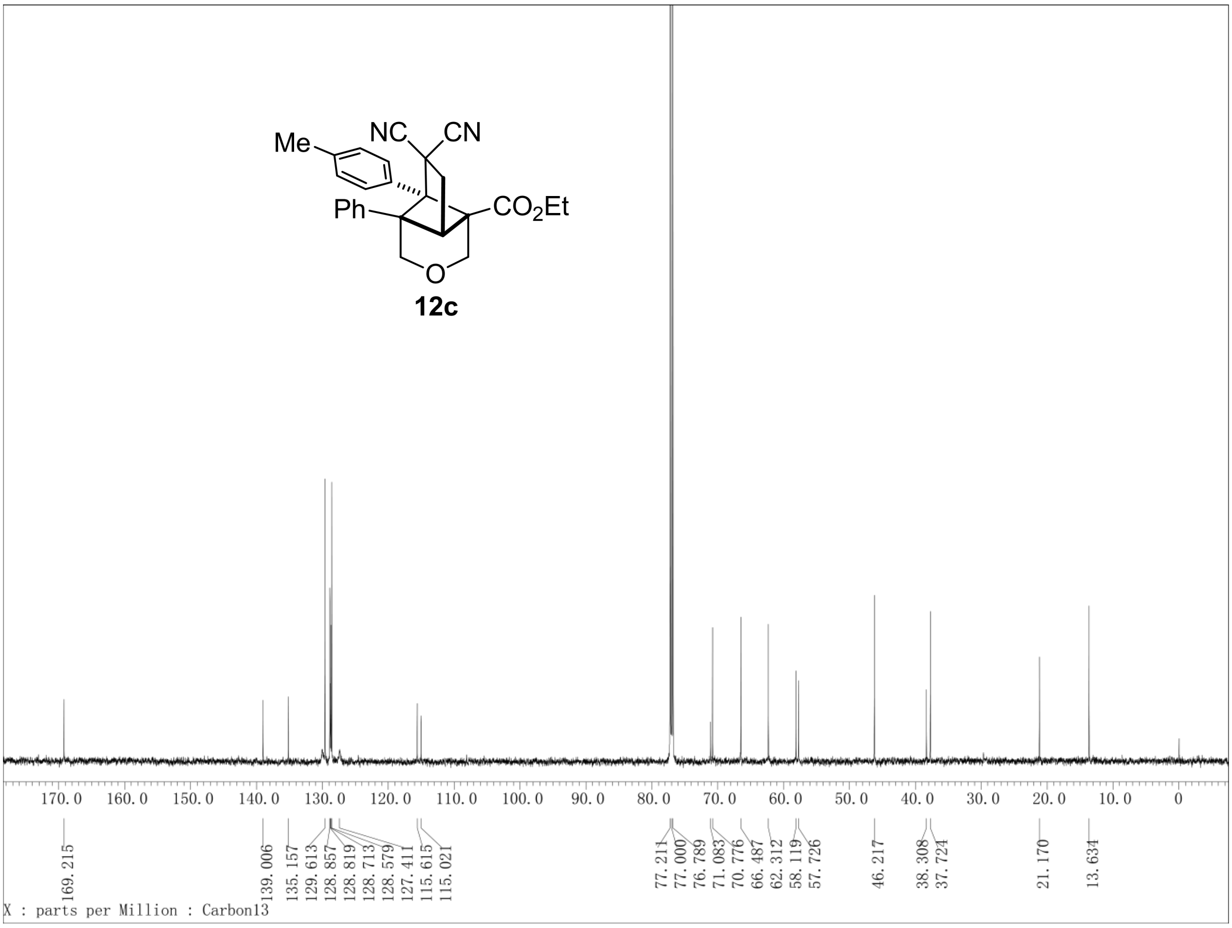

Me
NC
CN
Ph
CO_2Et
O
12c
170.0
160.0
150.0
140.0
130.0
120.0
110.0
100.0
90.0
80.0
70.0
60.0
50.0
40.0
30.0
20.0
10.0
0
169.215
139.006
135.157
129.613
128.857
128.819
128.713
128.579
127.411
115.615
115.021
77.211
77.000
76.789
71.083
70.776
66.487
62.312
58.119
57.726
46.217
38.308
37.724
21.170
13.634
X : parts per Million : Carbon13

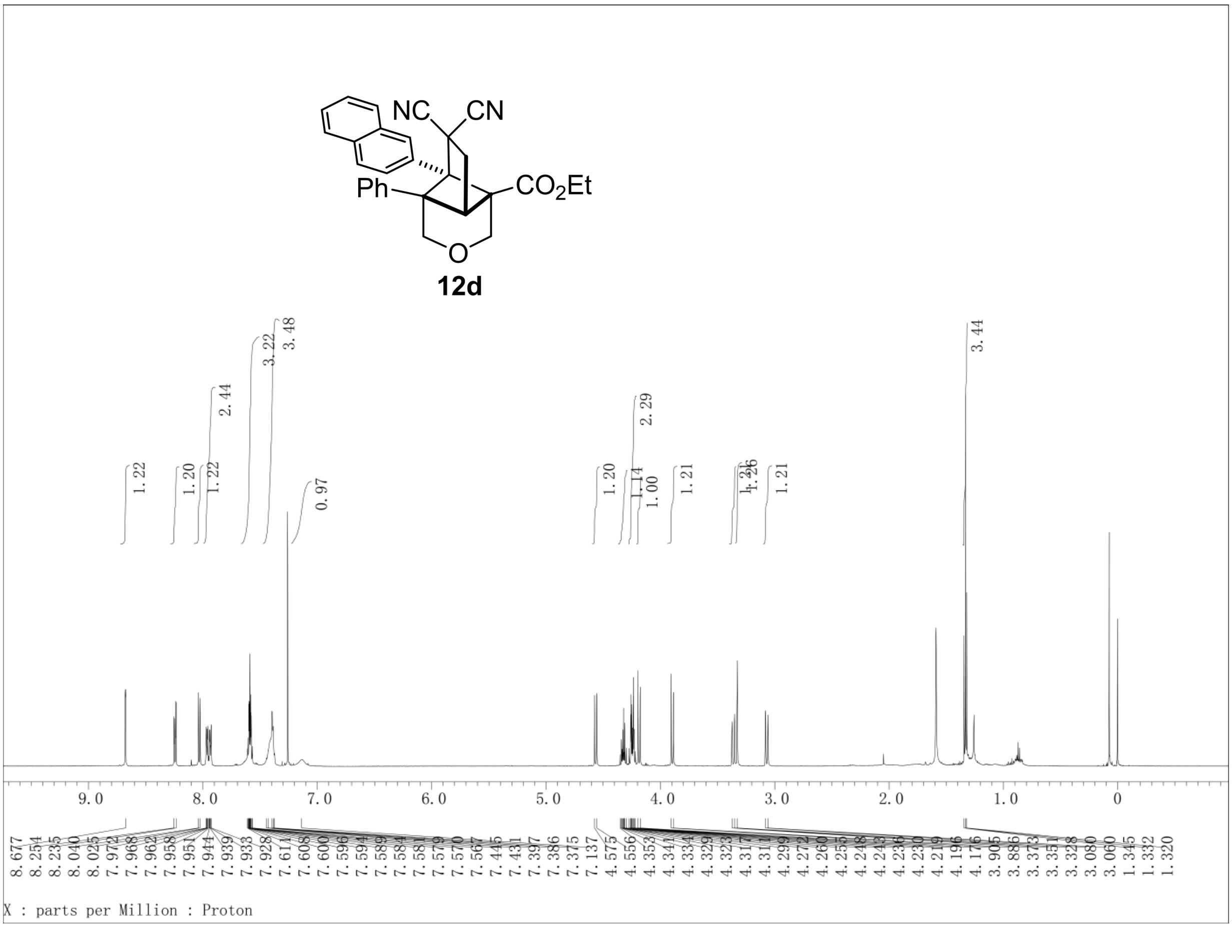
NC
CN
Ph
CO2Et
O
12d
1.22
1.20
1.22
2.44
3.22
3.48
0.97
1.20
1.14
2.29
1.00
1.21
1.26
1.21
3.44
9.0
8.0
7.0
6.0
5.0
4.0
3.0
2.0
1.0
0
8.677 8.254 8.235 8.040 8.025 7.972 7.968 7.962 7.958 7.951 7.944 7.939 7.933 7.928 7.611 7.608 7.600 7.596 7.594 7.589 7.584 7.581 7.579 7.570 7.567 7.445 7.431 7.397 7.386 7.375 7.137 4.575 4.556 4.353 4.347 4.334 4.329 4.323 4.317 4.311 4.299 4.272 4.260 4.255 4.248 4.243 4.236 4.230 4.219 4.196 4.176 3.905 3.886 3.373 3.351 3.328 3.080 3.060 1.345 1.332 1.320
X : parts per Million : Proton

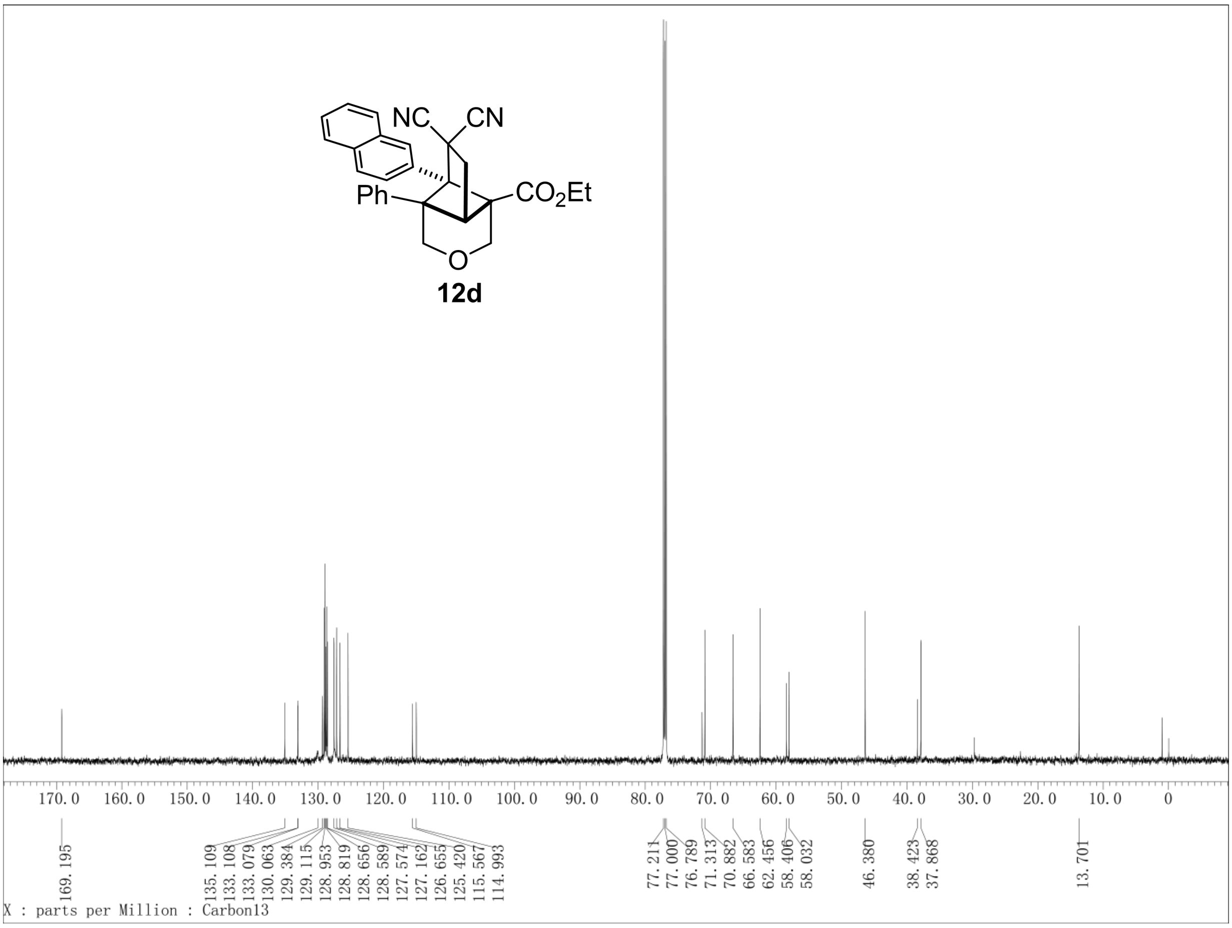
NC
CN
Ph
CO2Et
O
12d
170.0
160.0
150.0
140.0
130.0
120.0
110.0
100.0
90.0
80.0
70.0
60.0
50.0
40.0
30.0
20.0
10.0
0
169.195
135.109 133.108 133.079 130.063 129.384 129.115 128.953 128.819 128.656 128.589 127.574 127.162 126.655 125.420 115.567 114.993
77.211 77.000 76.789 71.313 70.882 66.583 62.456 58.406 58.032
46.380
38.423 37.868
13.701
X : parts per Million : Carbon13

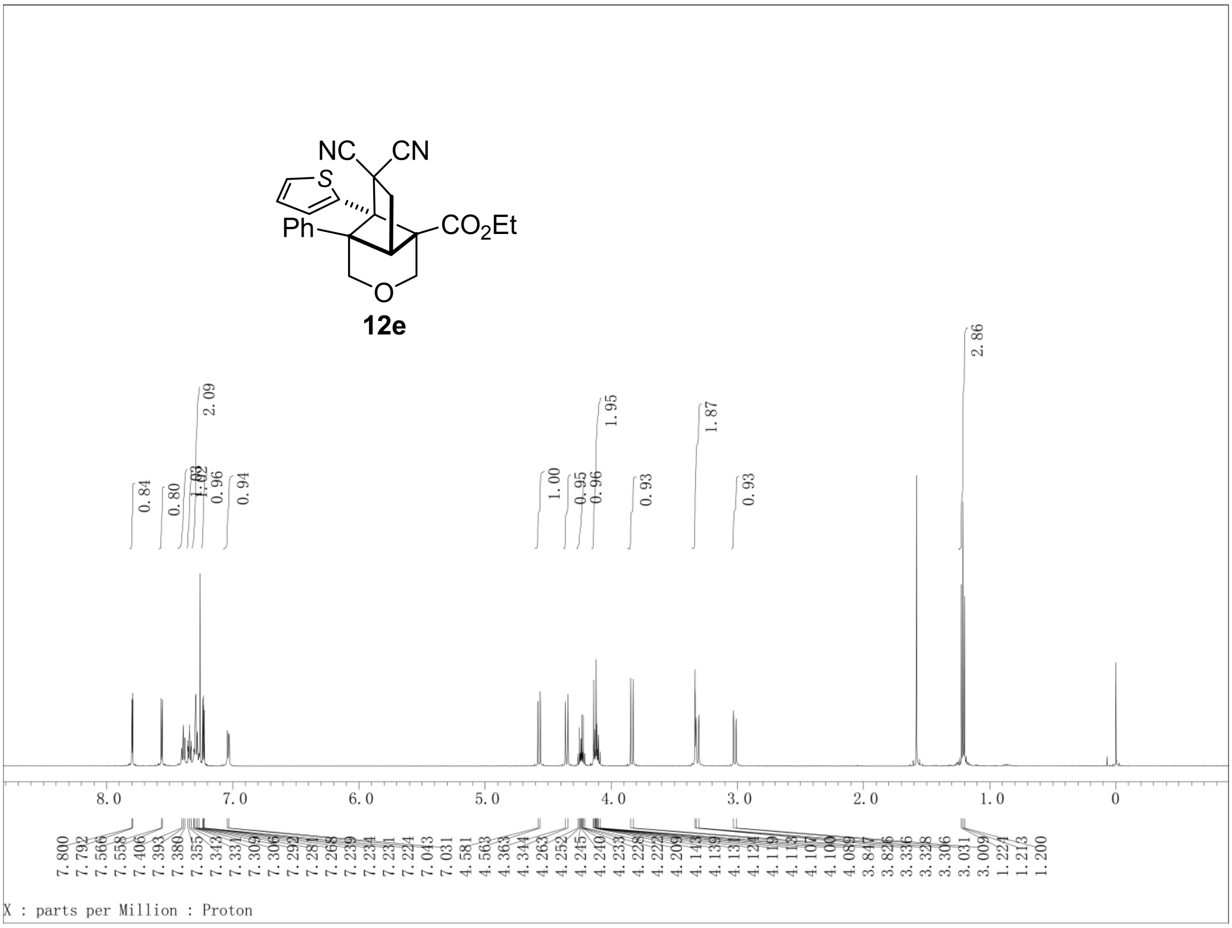
NC
CN
S
Ph
CO2Et
O
12e
0.84
0.80
1.02
1.02
2.09
0.96
0.94
1.00
0.95
0.96
1.95
0.93
1.87
0.93
2.86
8.0
7.0
6.0
5.0
4.0
3.0
2.0
1.0
0
7.800 7.792 7.566 7.558 7.406 7.393 7.380 7.355 7.342 7.331 7.309 7.306 7.292 7.281 7.268 7.239 7.234 7.231 7.224 7.043 7.031 4.581 4.563 4.363 4.344 4.263 4.252 4.245 4.240 4.233 4.228 4.222 4.209 4.143 4.139 4.131 4.124 4.119 4.113 4.107 4.100 4.089 3.847 3.826 3.336 3.328 3.306 3.031 3.009 1.224 1.213 1.200
X : parts per Million : Proton

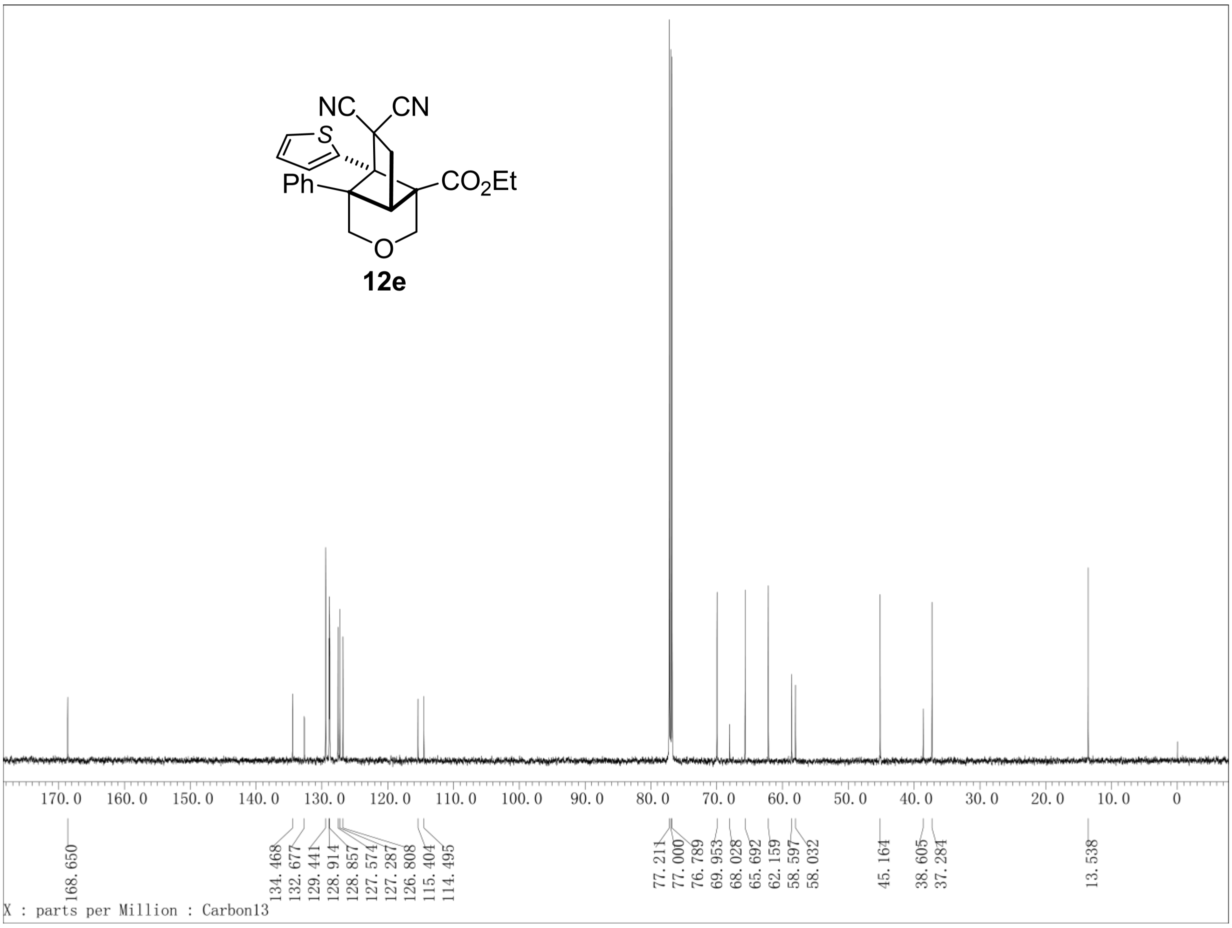
NC
CN
S
Ph
CO2Et
O
12e
170.0
160.0
150.0
140.0
130.0
120.0
110.0
100.0
90.0
80.0
70.0
60.0
50.0
40.0
30.0
20.0
10.0
0
168.650
134.468 132.677 129.441 128.914 128.857 127.574 127.287 126.808 115.404 114.495
77.211 77.000 76.789 69.953 68.028 65.692 62.159 58.597 58.032
45.164
38.605 37.284
13.538
X : parts per Million : Carbon13

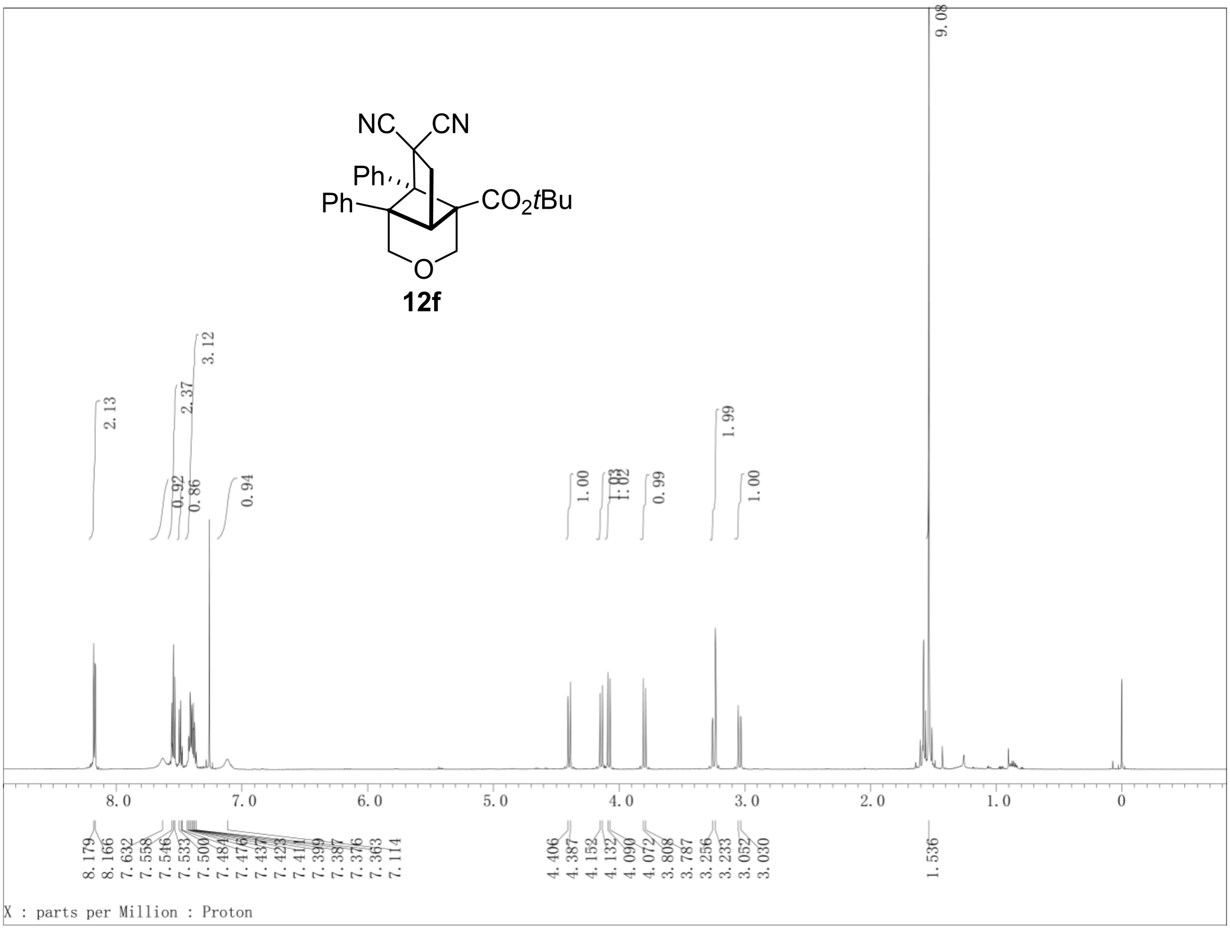
NC
CN
Ph
Ph
CO2tBu
O
12f
9.08
2.13
2.37
3.12
0.92
0.86
0.94
1.00
1.03
1.02
0.99
1.99
1.00
8.0
7.0
6.0
5.0
4.0
3.0
2.0
1.0
0
8.179
8.166
7.632
7.558
7.546
7.533
7.500
7.484
7.476
7.437
7.423
7.411
7.399
7.387
7.376
7.363
7.114
4.406
4.387
4.152
4.132
4.090
4.072
3.808
3.787
3.256
3.233
3.052
3.030
1.536
X : parts per Million : Proton

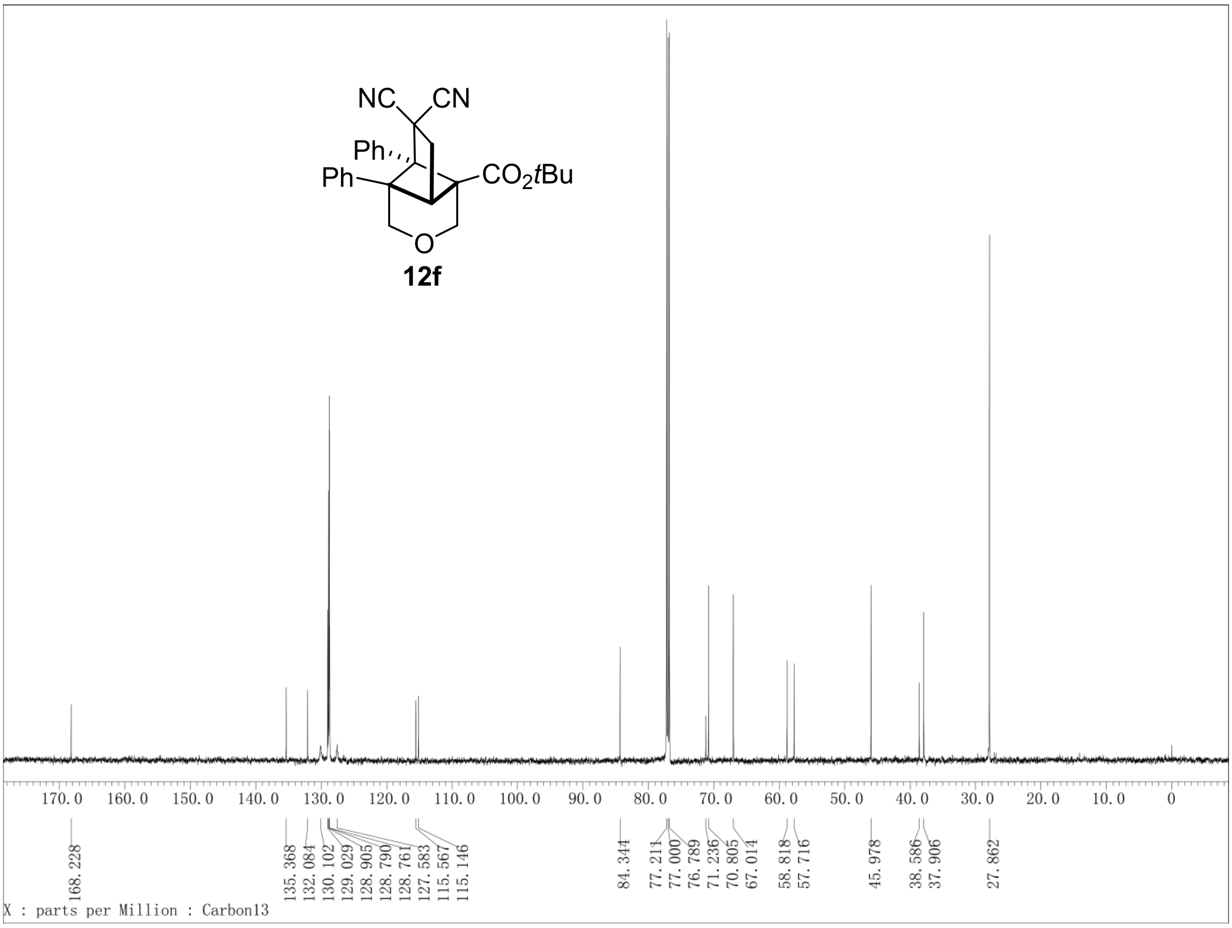
NC
CN
Ph
Ph
CO2tBu
O
12f
170.0
160.0
150.0
140.0
130.0
120.0
110.0
100.0
90.0
80.0
70.0
60.0
50.0
40.0
30.0
20.0
10.0
0
168.228
135.368
132.084
130.102
129.029
128.905
128.790
128.761
127.583
115.567
115.146
84.344
77.211
77.000
76.789
71.236
70.805
67.014
58.818
57.716
45.978
38.586
37.906
27.862
X : parts per Million : Carbon13

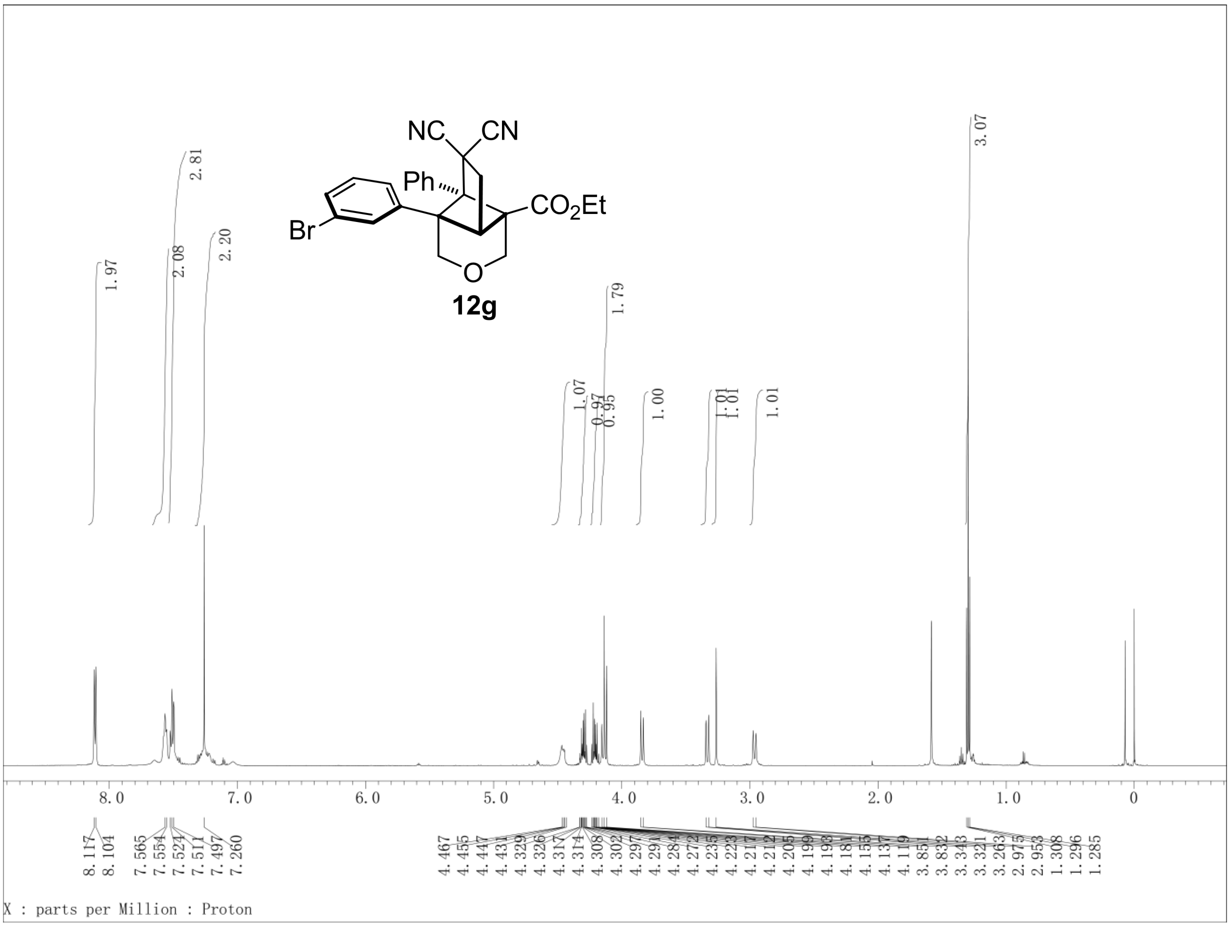
NC
CN
Ph
CO2Et
Br
O
12g
8.0
7.0
6.0
5.0
4.0
3.0
2.0
1.0
0
X : parts per Million : Proton

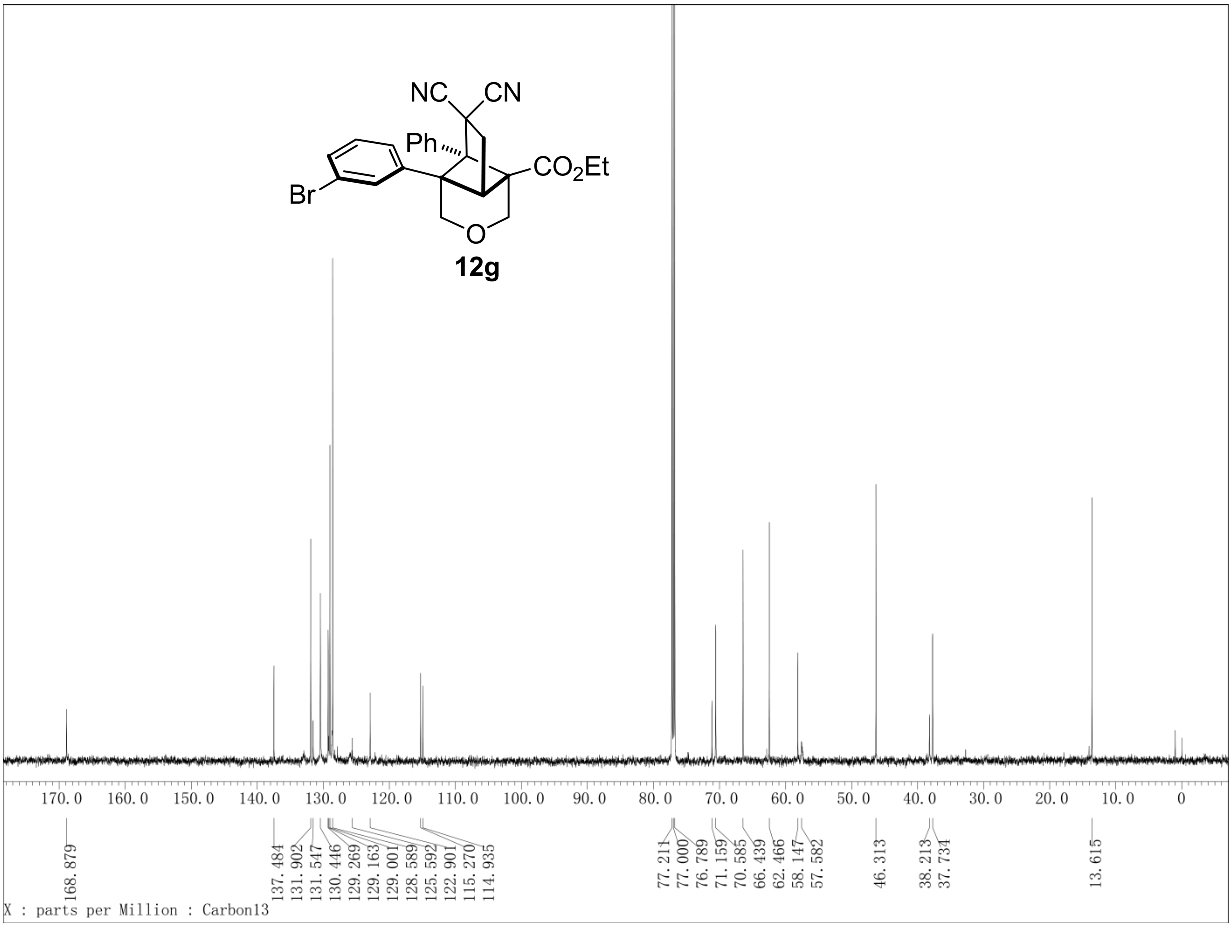
NC
CN
Ph
CO2Et
Br
O
12g
170.0
160.0
150.0
140.0
130.0
120.0
110.0
100.0
90.0
80.0
70.0
60.0
50.0
40.0
30.0
20.0
10.0
0
X : parts per Million : Carbon13

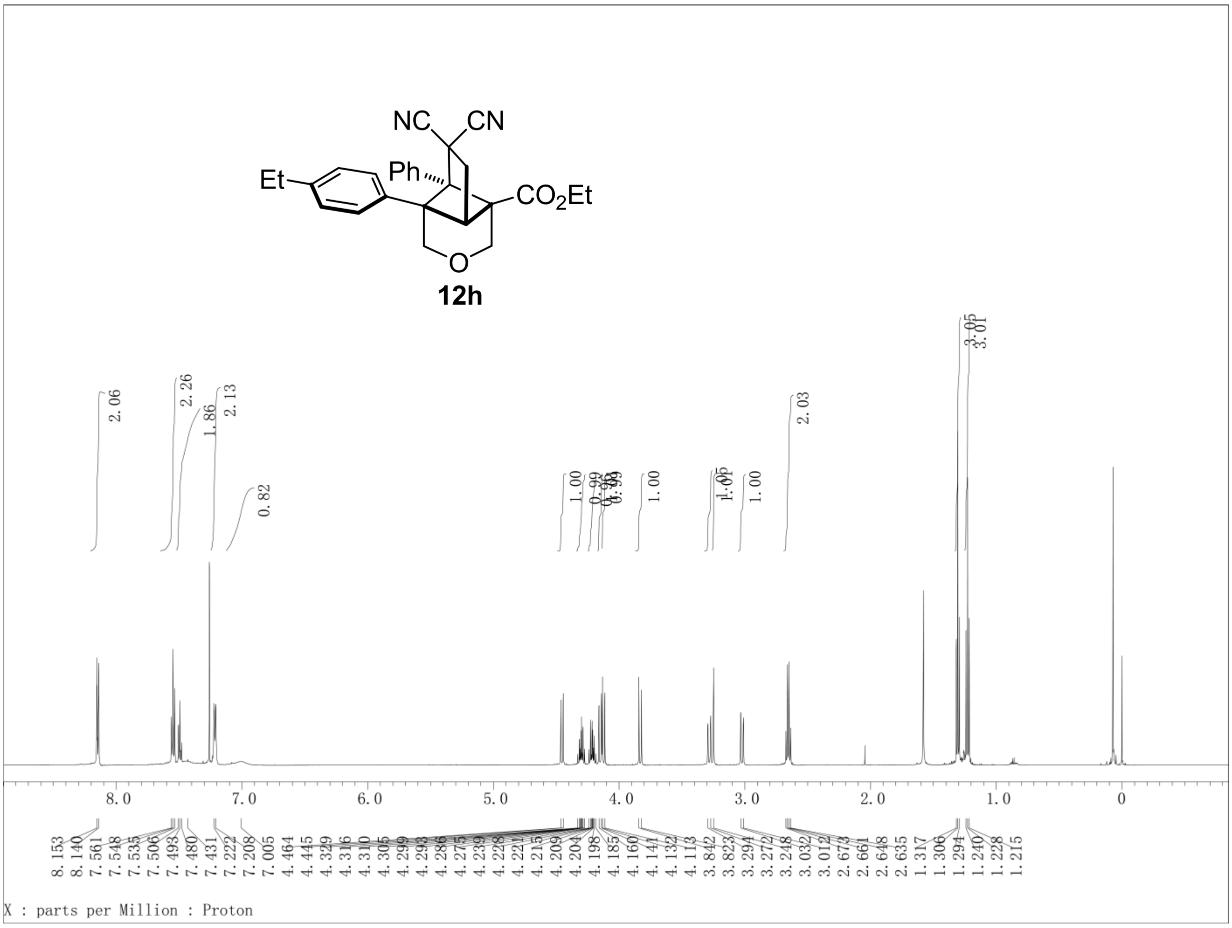
NC CN
Et
Ph
CO2Et
O
12h
2.06 2.26 1.86 2.13 0.82 1.00 1.00 1.00 1.05 1.01 1.00 2.03 3.05 3.01
8.153 8.140 7.561 7.548 7.535 7.506 7.493 7.480 7.431 7.222 7.208 7.005 4.464 4.445 4.329 4.316 4.310 4.305 4.299 4.293 4.286 4.275 4.239 4.228 4.221 4.215 4.209 4.204 4.198 4.185 4.160 4.141 4.132 4.113 3.842 3.823 3.294 3.272 3.248 3.032 3.012 2.673 2.661 2.648 2.635 1.317 1.306 1.294 1.240 1.228 1.215
X : parts per Million : Proton

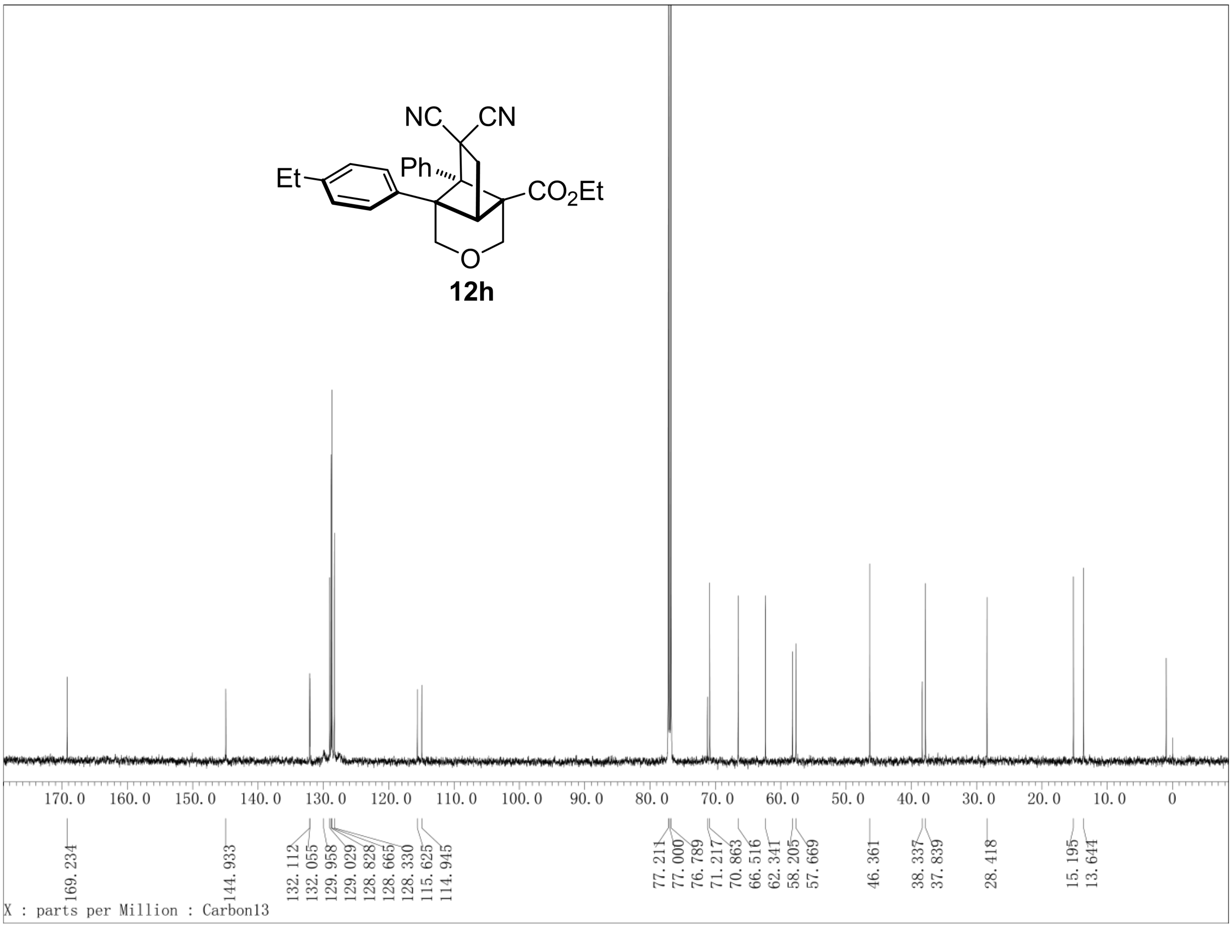
NC CN
Et
Ph
CO2Et
O
12h
169.234 144.933 132.112 132.055 129.958 129.029 128.828 128.665 128.330 115.625 114.945 77.211 77.000 76.789 71.217 70.863 66.516 62.341 58.205 57.669 46.361 38.337 37.839 28.418 15.195 13.644
X : parts per Million : Carbon13

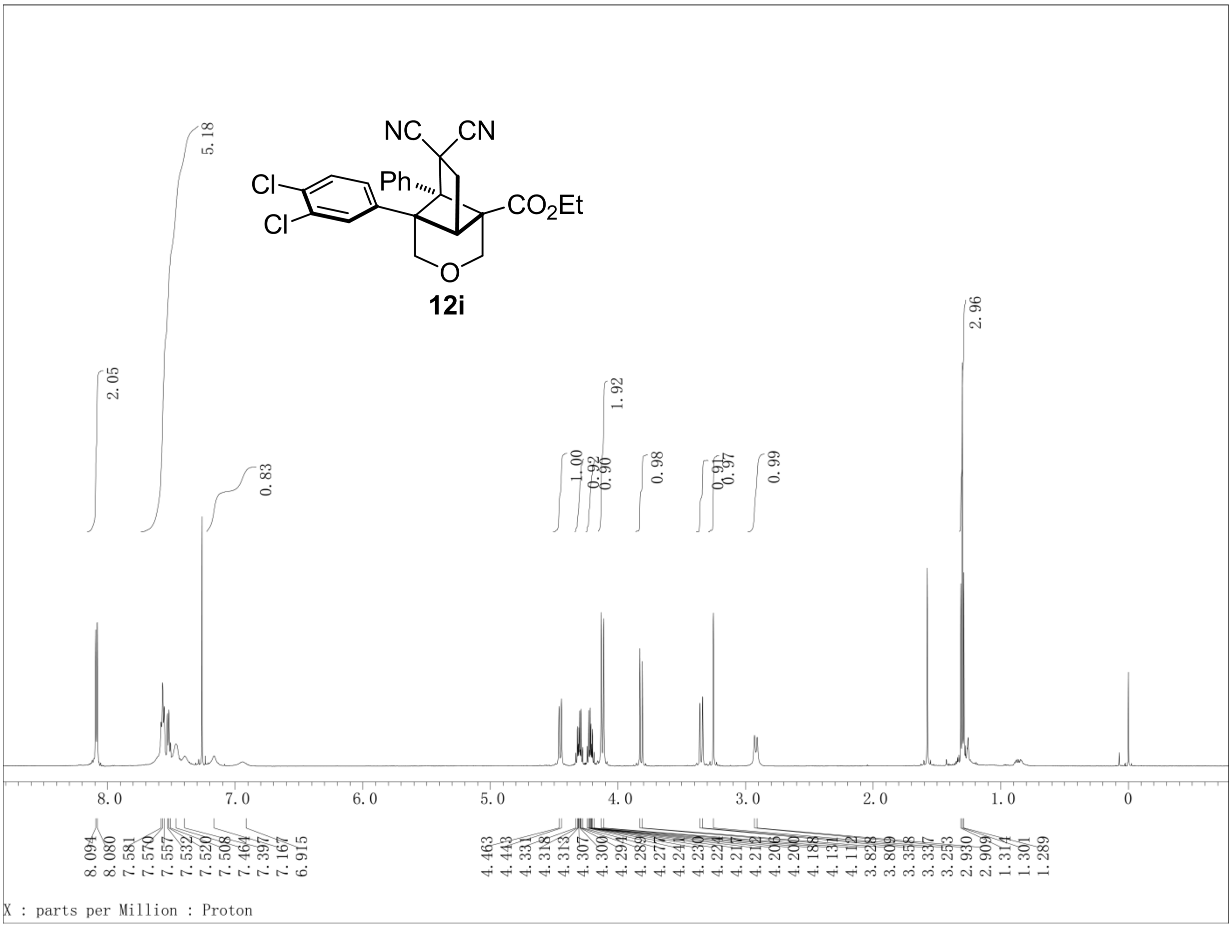
NC CN
Ph
Cl
Cl
CO2Et
O
12i
5.18
2.05
0.83
1.00
0.92
0.90
1.92
0.98
0.91
0.97
0.99
2.96
8.0
7.0
6.0
5.0
4.0
3.0
2.0
1.0
0
8.094
8.080
7.581
7.570
7.557
7.532
7.520
7.508
7.464
7.397
7.167
6.915
4.463
4.443
4.331
4.318
4.313
4.307
4.300
4.294
4.289
4.277
4.241
4.230
4.224
4.217
4.212
4.206
4.200
4.188
4.131
4.112
3.828
3.809
3.358
3.337
3.253
2.930
2.909
1.314
1.301
1.289
X : parts per Million : Proton

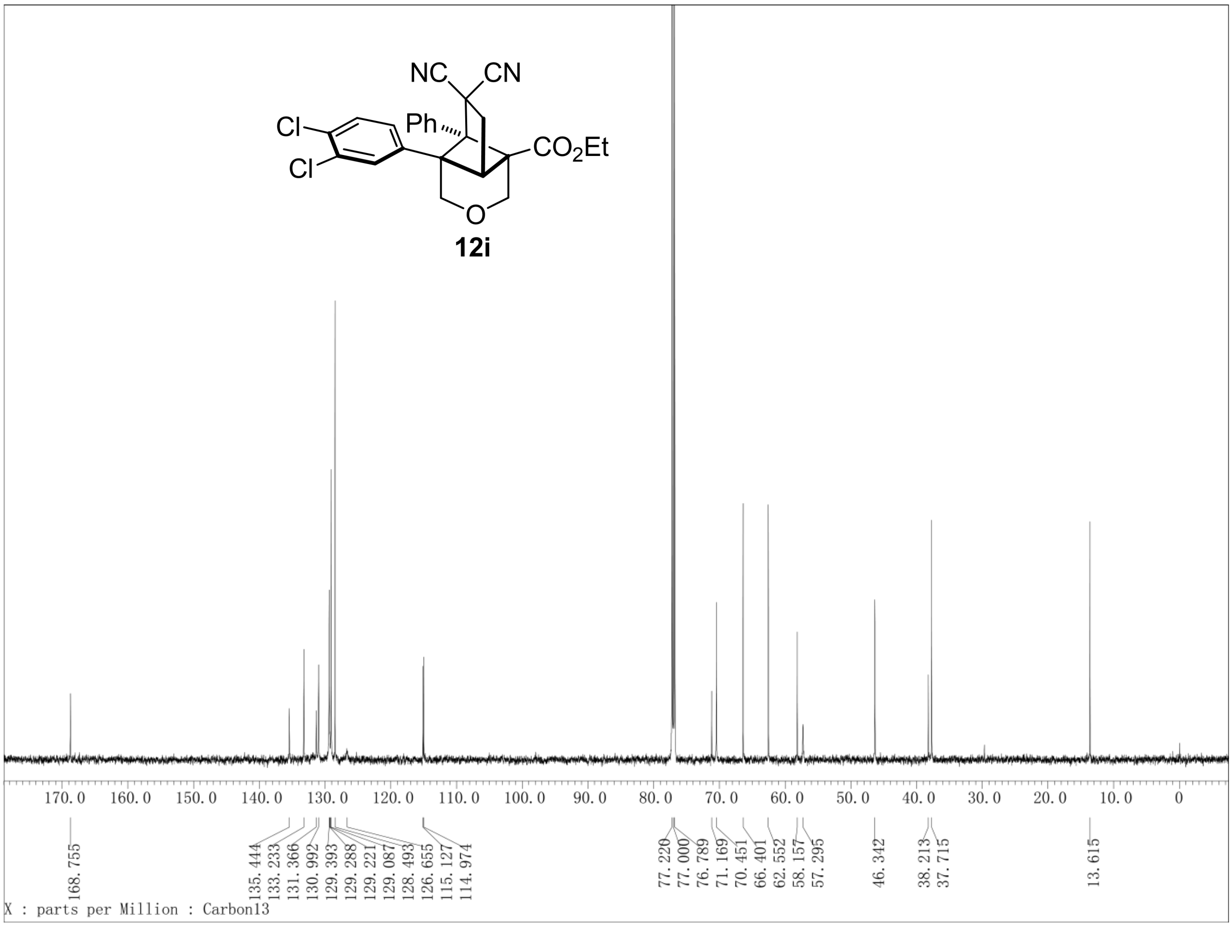
NC CN
Ph
Cl
Cl
CO2Et
O
12i
170.0
160.0
150.0
140.0
130.0
120.0
110.0
100.0
90.0
80.0
70.0
60.0
50.0
40.0
30.0
20.0
10.0
0
168.755
135.444
133.233
131.366
130.992
129.393
129.288
129.221
129.087
128.493
126.655
115.127
114.974
77.220
77.000
76.789
71.169
70.451
66.401
62.552
58.157
57.295
46.342
38.213
37.715
13.615
X : parts per Million : Carbon13

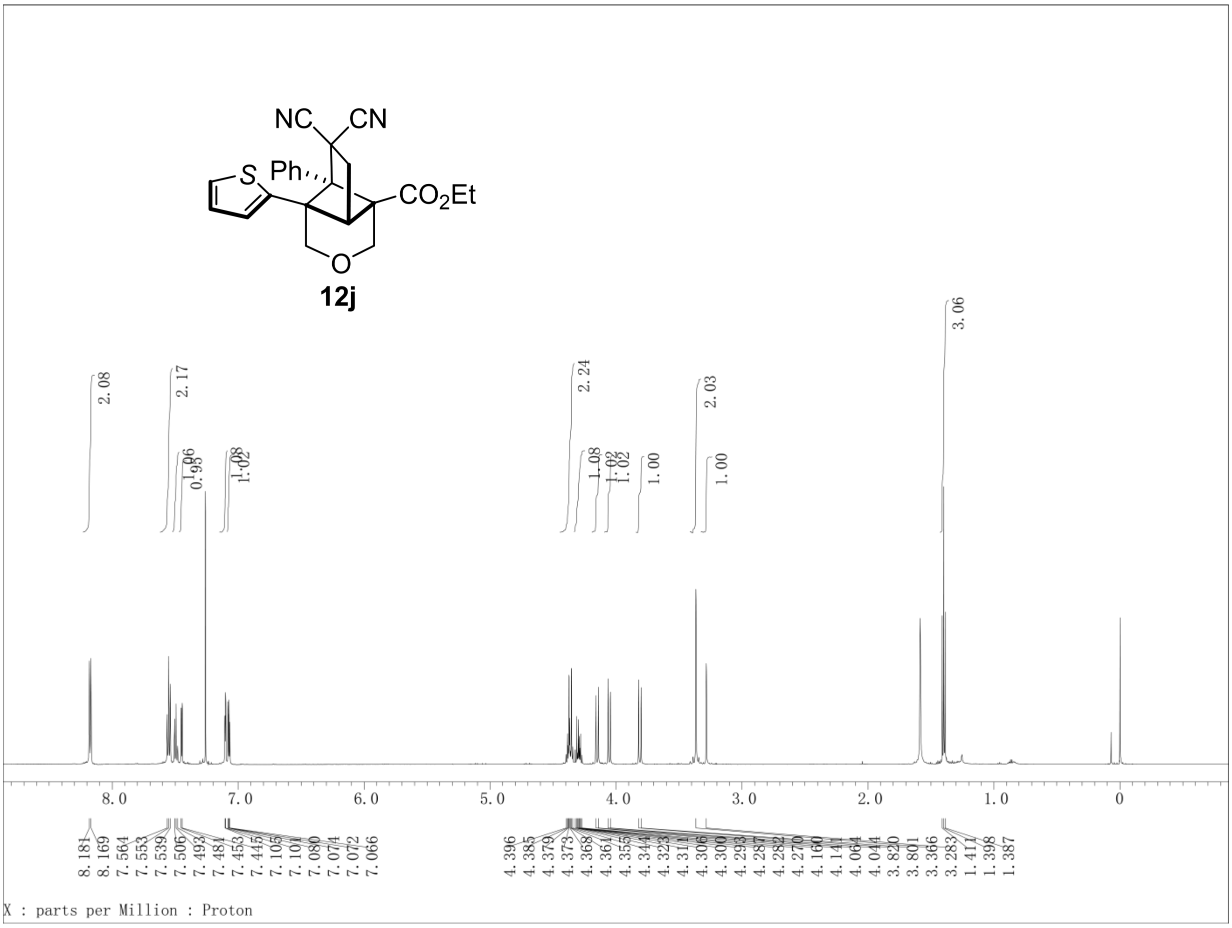
NC
CN
Ph
S
CO_2Et
O
12j
X : parts per Million : Proton

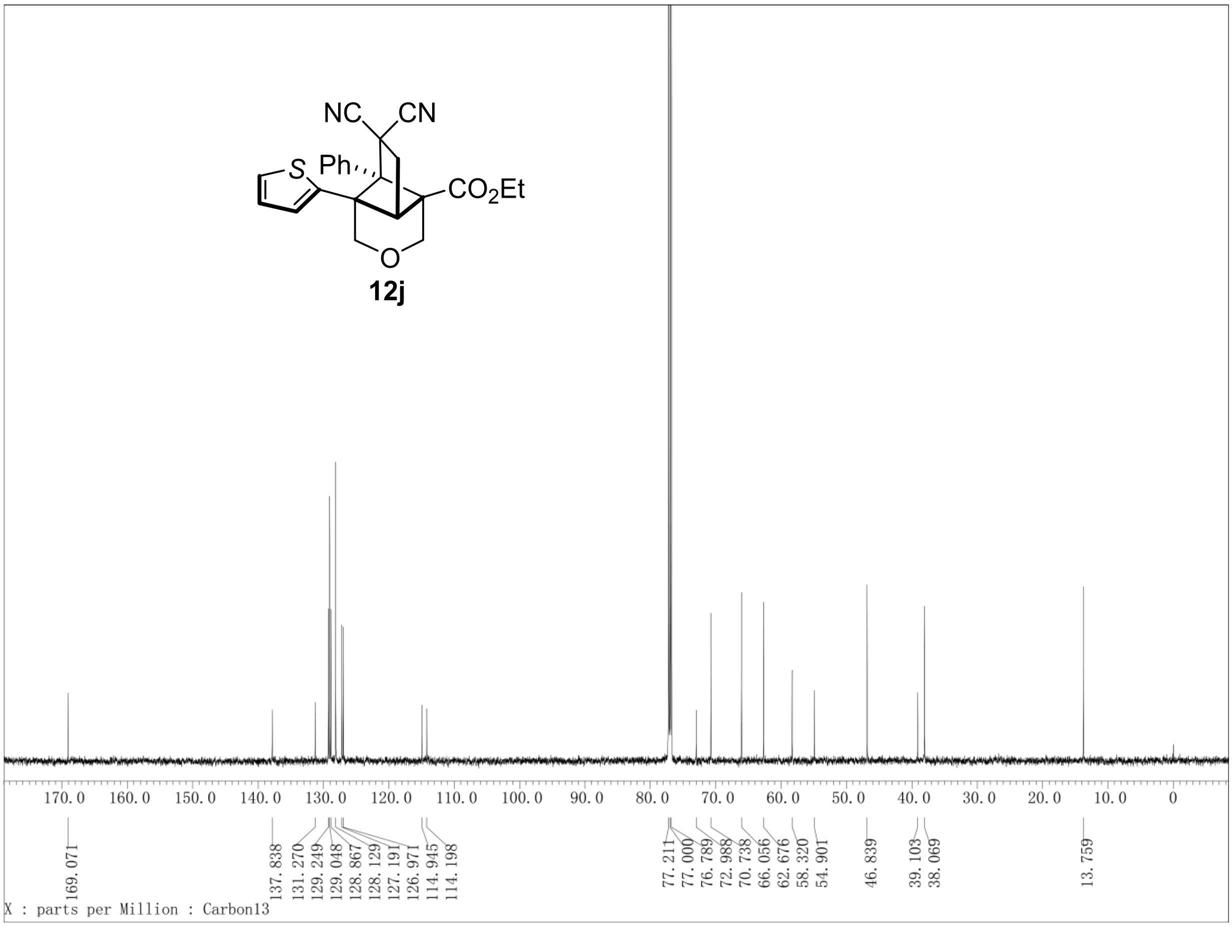
NC
CN
Ph
S
CO_2Et
O
12j
X : parts per Million : Carbon13

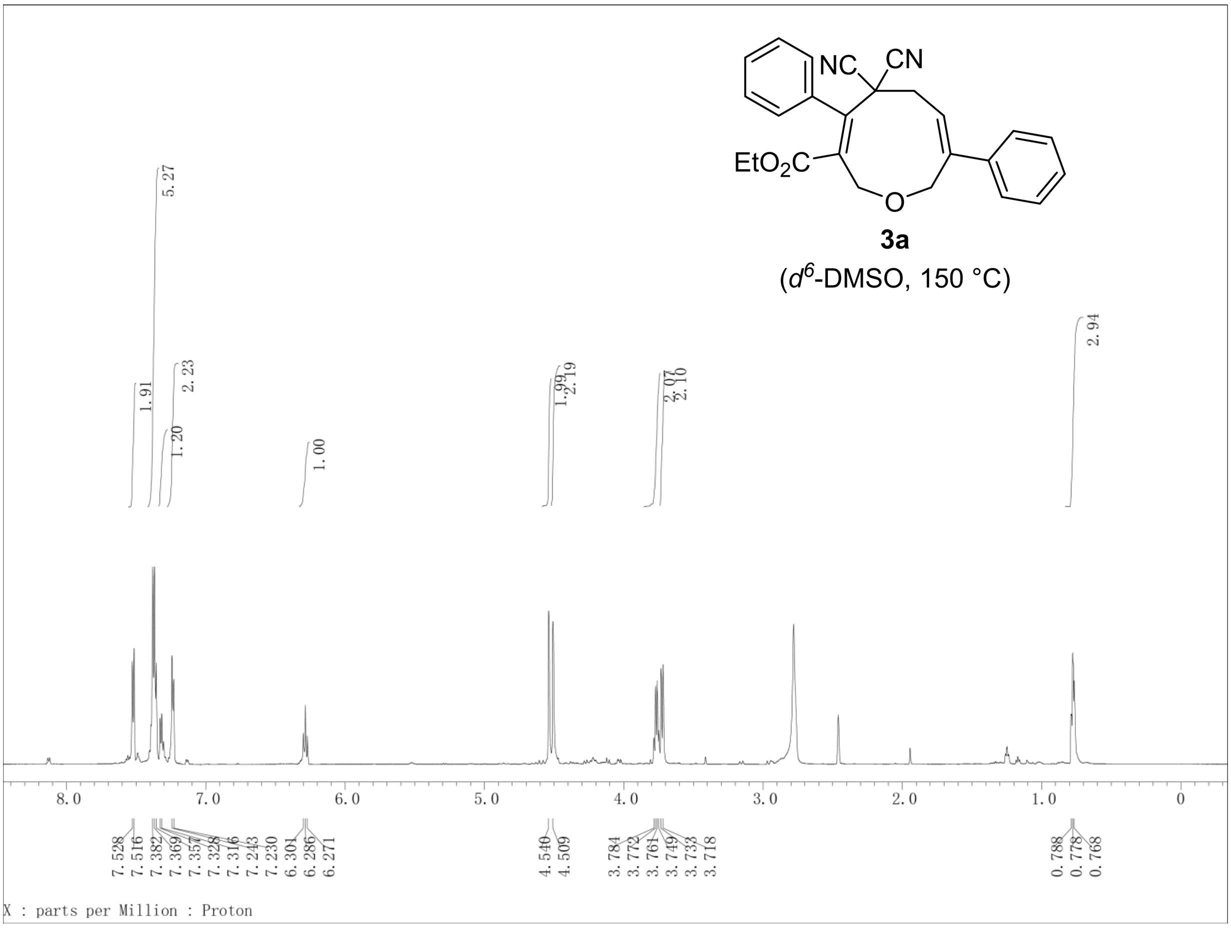
NC
CN
EtO2C
O
3a
(d6-DMSO, 150 °C)
5.27
1.91
1.20
2.23
1.00
1.99
2.19
2.07
2.10
2.94
8.0
7.0
6.0
5.0
4.0
3.0
2.0
1.0
0
7.528
7.516
7.382
7.369
7.357
7.328
7.316
7.243
7.230
6.301
6.286
6.271
4.540
4.509
3.784
3.772
3.761
3.749
3.733
3.718
0.788
0.778
0.768
X : parts per Million : Proton

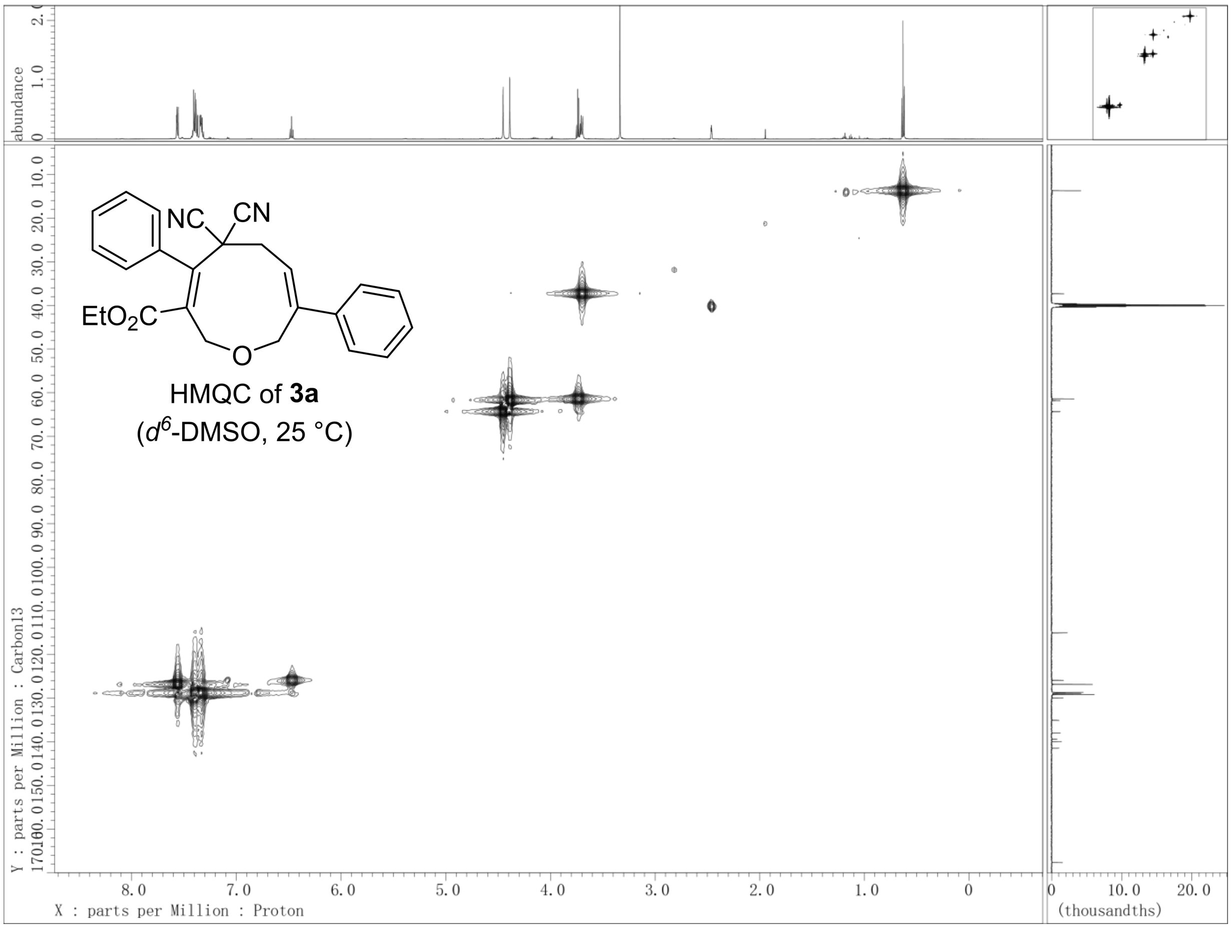
abundance
NC
CN
EtO2C
O
HMQC of 3a
(d6-DMSO, 25 °C)
Y : parts per Million : Carbon13
X : parts per Million : Proton
(thousandths)

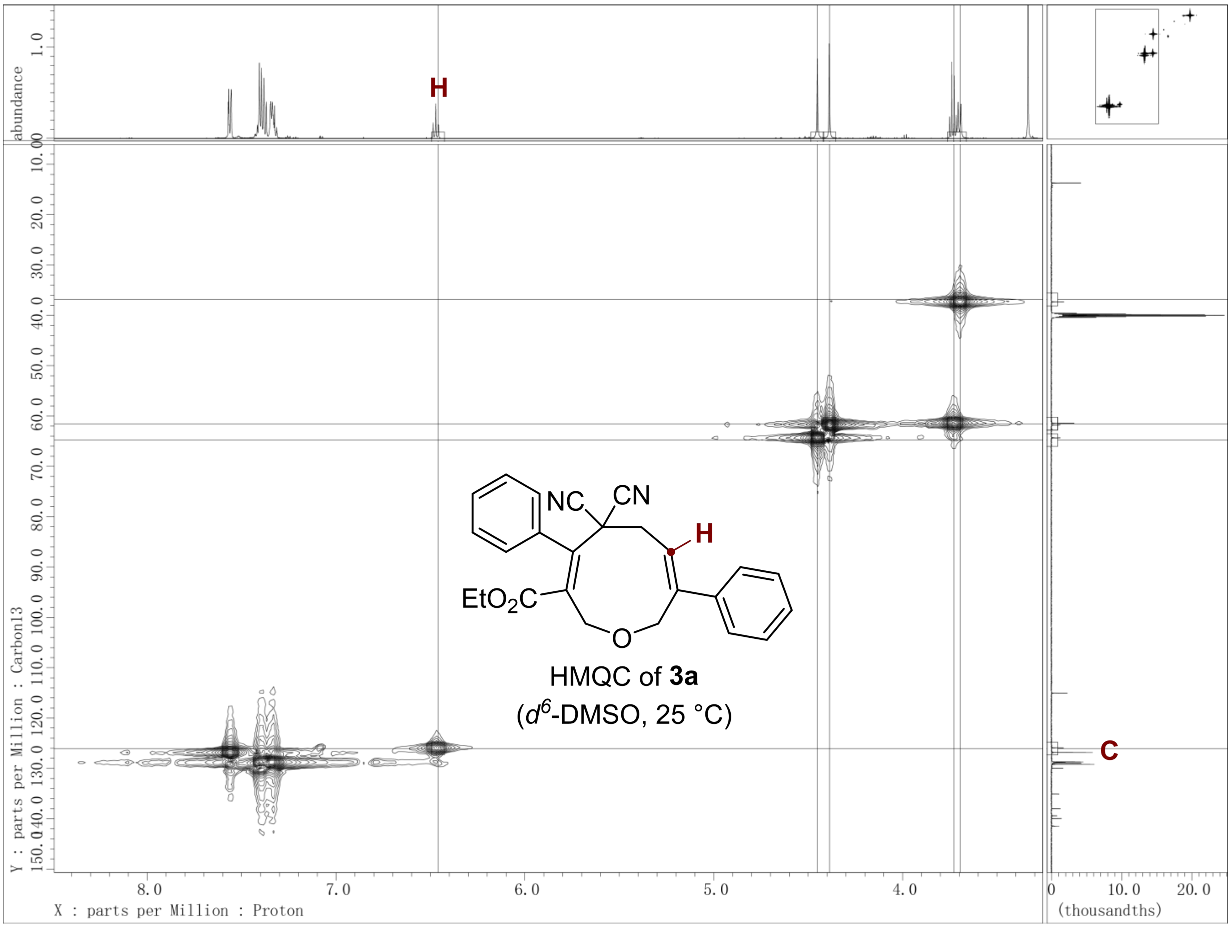

abundance
H
NC
CN
EtO2C
O
HMQC of 3a
(d6-DMSO, 25 °C)
C
Y : parts per Million : Carbon13
X : parts per Million : Proton
(thousandths)

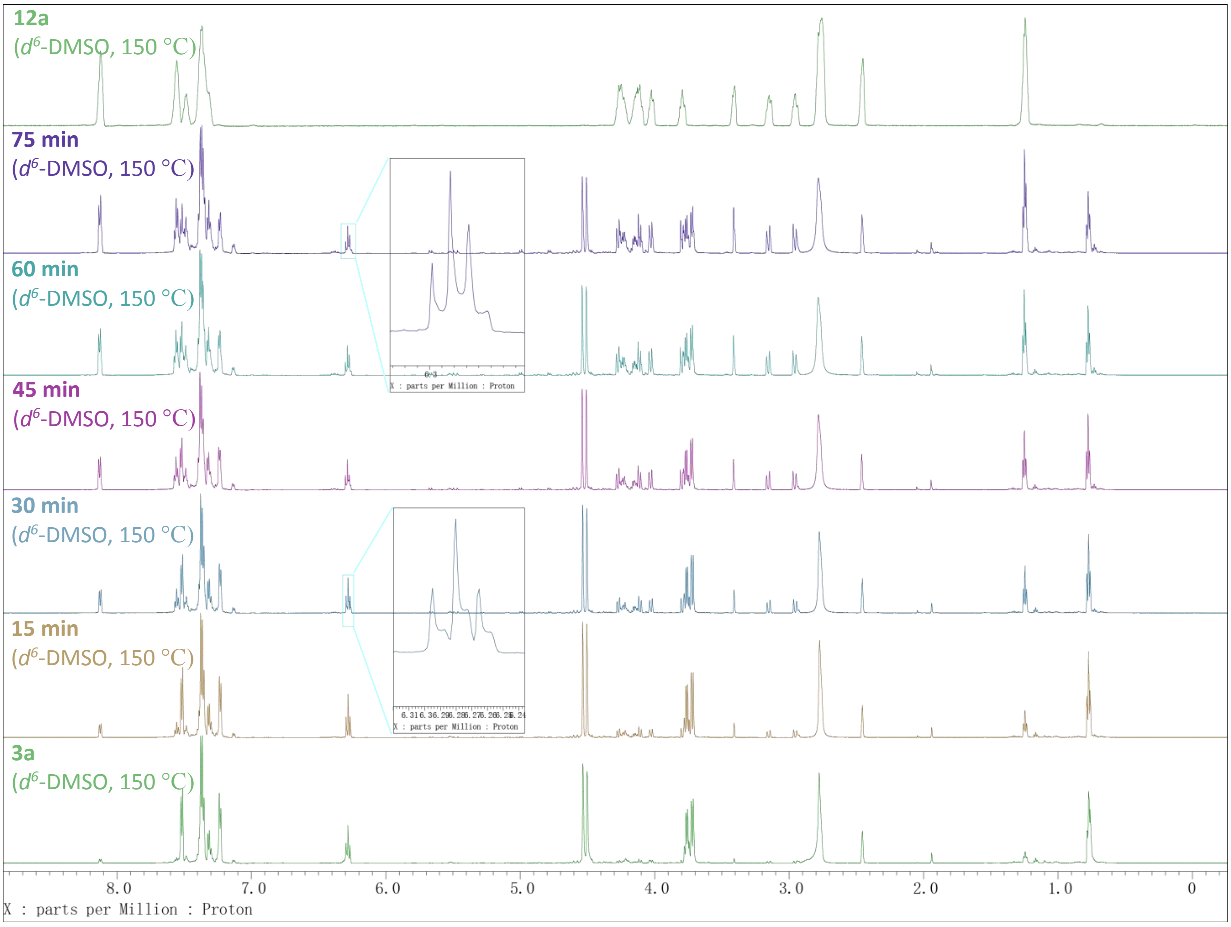

12a
(d6-DMSO, 150 °C)
75 min
(d6-DMSO, 150 °C)
60 min
(d6-DMSO, 150 °C)
45 min
(d6-DMSO, 150 °C)
30 min
(d6-DMSO, 150 °C)
15 min
(d6-DMSO, 150 °C)
3a
(d6-DMSO, 150 °C)
X : parts per Million : Proton
